# Airborne Ragweed (*Ambrosia artemisiifolia*) Allergen Exposure and Sensitization Pattern in Western Romania: A 5-Year Retrospective Cross-Sectional Observational Analysis of Sensitization Prevalence, Complemented by a Parallel Temporal Analysis of Aerobiological Data and Symptom-Driven Healthcare Presentation Patterns Study

**DOI:** 10.3390/life16030526

**Published:** 2026-03-22

**Authors:** Valentin-Cristian Iovin, Carmen Neamtu, Roxana Buzan, Corina Porr, Alina-Daniela Totorean, Ana-Adina Iovin, Andreea-Adriana Neamtu, Diana Luisa Lighezan, Carmen Panaitescu

**Affiliations:** 1 Doctoral School Department, “Victor Babes” University of Medicine and Pharmacy of Timisoara, 300041 Timisoara, Romania; valentin.iovin@umft.ro; 2 Department III Functional Sciences, Physiology Discipline, “Victor Babes” University of Medicine and Pharmacy, 300041 Timisoara, Romania; cbunu@umft.ro; 3 Centre of Immuno-Physiology and Biotechnologies (CIFBIOTEH), Department of Functional Sciences, Physiology, “Victor Babes” University of Medicine and Pharmacy of Timisoara, 300041 Timisoara, Romania; 4 Faculty of Dentistry, “Vasile Goldis” Western University of Arad, 310045 Arad, Romania; neamtu.carmen@uvvg.ro; 5 Department of Surgery I, Clinical County Emergency Hospital of Arad, 310037 Arad, Romania; 6 OncoGen Center, Pius Brinzeu County Clinical Emergency Hospital, 300723 Timisoara, Romania; buzan.roxana@umft.ro; 7 Allergology Department, Faculty of Medicine, Lucian Blaga University, 550169 Sibiu, Romania; corina_sibiu@yahoo.com; 8 Department of Balneology, Medical Rehabilitation and Rheumatology, “Victor Babes” University of Medicine and Pharmacy of Timisoara, Eftimie Murgu Square, No. 2, 300041 Timisoara, Romania; totorean.alina@umft.ro; 9 Department of Physical and Rehabilitation Medicine, “Pius Brinzeu” Clinical County Emergency Hospital Timisoara, Liviu Rebreanu Boulevard, No. 156, 300723 Timisoara, Romania; 10 Department of Pathology, Clinical County Emergency Hospital of Arad, Andrenyi Karoly Str., No. 2–4, 310037 Arad, Romania; adina.iovin@gmail.com; 11 Department of Pathology, “Pius Brinzeu” Clinical County Emergency Hospital Timisoara, Liviu Rebreanu Boulevard, No. 156, 300723 Timisoara, Romania; 12 Department of Toxicology, “Victor Babes” University of Medicine and Pharmacy of Timisoara, Eftimie Murgu Square, No. 2, 300041 Timisoara, Romania; 13 Research Centre for Pharmaco-Toxicological Evaluation, “Victor Babes” University of Medicine and Pharmacy of Timisoara, Eftimie Murgu Square, No. 2, 300041 Timisoara, Romania; 14 Department of Hematology, “Victor Babes” University of Medicine and Pharmacy of Timisoara, Eftimie Murgu Square, No. 2, 300041 Timisoara, Romania

**Keywords:** sensitization, skin prick test, *Ambrosia artemisiifolia*, ragweed, aeroallergens, polysensitization, monosensitization, epidemiology, Romania

## Abstract

Ragweed (*Ambrosia artemisiifolia*) represents a major and expanding source of aeroallergen exposure in Europe, with rising sensitization rates and substantial clinical impact. However, population-level data integrating airborne pollen exposure with detailed clinical sensitization patterns remain limited. We conducted a 5-year retrospective cross-sectional observational analysis of sensitization prevalence, complemented by a parallel temporal analysis of aerobiological data and symptom-driven healthcare presentation patterns (2020–2024) in Timisoara, Romania, including all patients undergoing first-time sensitization evaluation at a tertiary referral hospital. Sensitization was assessed using standardized skin prick testing to common aeroallergens and other allergen categories, while airborne ragweed pollen concentrations were monitored through a peri-urban network of real-time bio-particle analyzers. Statistical analyses included descriptive statistics, multivariable logistic regression, χ^2^ tests for co-sensitization patterns, and comparative analyses of clinical manifestations across sensitization profiles. Among 4404 eligible patients, 50.7% were sensitized to at least one allergen. Ragweed sensitization was identified in 24.1% of patients, with a mean age of 31.1 years at diagnosis and no significant sex-related difference. Most ragweed-sensitized patients were polysensitized (71.5%), predominantly to other aeroallergens. Increasing age was independently associated with lower odds of polysensitization to other aeroallergens (adjusted OR = 0.97 per year, 95% CI: 0.96–0.98), while sex showed no independent association. Patients with ragweed sensitization alone and those cosensitized with aeroallergens exhibited similar prevalence of respiratory manifestations, whereas individuals with additional non-aeroallergen sensitization showed lower prevalence of rhinitis, conjunctivitis, and asthma but slightly higher rates of asthma exacerbations. Weekly diagnoses of ragweed sensitization demonstrated a pronounced seasonal peak between calendar weeks 33 and 38 (mid-August to late September), coinciding with peak airborne ragweed pollen concentrations. Ragweed sensitization therefore represents a substantial and seasonally driven healthcare burden in western Romania, characterized by frequent polysensitization, distinct clinical manifestation patterns across sensitization profiles, and close temporal alignment between airborne pollen exposure and clinical presentation. Integrating aerobiological monitoring with clinical surveillance may support targeted prevention strategies and improved patient management.

## 1. Introduction

The epidemiological significance of ragweed sensitization has been documented through multiple national and regional monitoring programs, which report rising sensitization rates and increasing patient volumes over recent decades [1,2]. In Germany, marked increases in ragweed sensitization have been observed over a 20-year period (1998–2017), with the most pronounced rises occurring in younger patient cohorts [1]. Similarly, large clinical studies from Asia have reported remarkably high sensitization rates, with researchers from Beijing documenting a 45.5% positive skin-prick test rate to ragweed among 9727 symptomatic patients, reaching 65.5% in adolescents [3]. In the Black Sea region, ragweed has been identified as the most important seasonal allergen, reflecting both its biological invasiveness and allergenic potency [4]. These epidemiological trends underscore the growing clinical relevance of ragweed sensitization and subsequent potential development of allergic disease and highlight the need for studies to quantify disease prevalence and progression.

Ragweed (*Ambrosia artemisiifolia*) has emerged as one of the most significant aeroallergen sources in Europe, with its invasive spread and expanding geographic range posing increasing public health challenges across the continent [5,6]. As a highly allergenic plant species, ragweed produces large amounts of pollen, which is capable of triggering severe allergic rhinoconjunctivitis, asthma exacerbations, and other respiratory manifestations in sensitized individuals [7,8]. The clinical burden of ragweed allergy is substantial, with recent studies showing that nearly all ragweed-sensitized patients experience rhinoconjunctivitis, approximately 60% report asthma-like symptoms, and a significant proportion develop skin manifestations [9]. High-resolution exposure mapping across Europe has identified large regions with substantial seasonal ragweed pollen exposure, enabling continent-scale risk assessment and revealing the expanding geographical footprint of this invasive allergen [5].

Environmental and climate factors play a critical role in modulating ragweed pollen production, dispersal, and allergenic potency, thereby influencing population exposure and sensitization risk [10,11,12]. Elevated atmospheric CO_2_ concentrations have been experimentally shown to increase both pollen production and allergenic potency. In this context, controlled chamber studies demonstrate stronger lung inflammation and higher IgE responses to pollen from plants grown under elevated CO_2_ conditions [11]. Temperature increase and shifting phenological patterns associated with climate change have resulted in earlier pollen season onset, prolonged blooming periods, and higher seasonal pollen integrals [12]. Additionally, air pollutants such as carbon monoxide and sulfur dioxide have been correlated with increased airborne allergen concentrations and enhanced pollen allergenic potential [10]. Long-distance pollen transport events, facilitated by wind patterns, have extended ragweed pollen seasons even in regions where the plant is not locally established, as documented in Finland and other northern European countries [13]. These environmental drivers operate synergistically at local, regional, and continental scales, creating a dynamic and intensifying the exposure landscape that requires comprehensive epidemiological surveillance.

Despite the growing body of evidence on ragweed sensitization and allergy, significant knowledge gaps remain, justifying prospective epidemiological investigations [5,9,10]. Current literature is characterized by a paucity of cohort data quantifying the incidence of new sensitizations and the natural history of disease progression from initial sensitization to established allergic rhinitis or asthma [9]. There is insufficient linkage between ambient allergen exposure metrics—including Amb a 1 concentrations (the most studied therapeutic target) and pollen allergenic potential (PAP)—and individual clinical outcomes [10]. CRD approaches have rarely been integrated into large-scale epidemiological studies, limiting our understanding of the clinical relevance of non-Amb a 1 sensitization at the population level [9,14]. Furthermore, the effects of environmental modifiers such as CO_2_, temperature, precipitation, and air pollutants on sensitization risk and clinical severity remain inadequately characterized through longitudinal population-based research [11,12]. Real-world effectiveness data for environmental control measures, plant eradication policies, and allergen immunotherapy interventions are also limited [4].

The western side of Romania represents a particularly relevant geographical setting for ragweed epidemiological research, given the documented expansion of *Ambrosia artemisiifolia* in the region and the increasing clinical burden observed in allergology clinics [8,9]. The present study addresses critical gaps in the scientific literature by conducting a 5-year retrospective cross-sectional observational analysis of sensitization prevalence, complemented by a parallel temporal analysis of aerobiological data and symptom-driven healthcare presentation patterns, regarding ragweed sensitization. By integrating aerobiological monitoring and clinical phenotyping, this research aims to elucidate the temporal dynamics of ragweed sensitization, identify patterns of allergen exposure, and quantify the clinical impact of ragweed sensitization in a well-defined population. The findings will contribute to evidence-based public health strategies for ragweed allergy prevention and patient management.

## 2. Materials and Methods

### 2.1. Study Design and Setting

This is a retrospective cross-sectional observational analysis of sensitization prevalence, complemented by a parallel temporal analysis of aerobiological data and symptom-driven healthcare presentation patterns study conducted under the coordination of the Allergology Department of the OncoGen Center, part of the “Pius Brinzeu” Clinical County Emergency Hospital, Timisoara, Romania. The study period covers five years, from 1 January 2020 to 31 December 2024. The hospital serves a population of approximately 650,000 people [15].

### 2.2. Patient Selection

This 5-year retrospective study included all patients evaluated at the study centers during the study period.

Inclusion criteria:

• Patients undergoing diagnostic procedures for allergy evaluation, including skin prick testing for sensitization;
• Patients not previously diagnosed with sensitization to any allergen;
• Patients of any age.

Exclusion criteria:

• Patients with sensitizations diagnosed prior to consultation at the study center.

### 2.3. Ethics Approval

The retrospective observational study protocol was approved by the Ethics Committees of “Pius Brinzeu” Clinical County Emergency Hospital and “Victor Babes” University of Medicine and Pharmacy, Timisoara (Approval number: No. 316 issued on 3 August 2022, and No. 70 issued on 28 May 2025, respectively). The study was conducted in accordance with the Declaration of Helsinki. Due to the retrospective nature of the study, the requirement for specific informed consent was waived. Nevertheless, as patients of the OncoGen Center, patients agreed for their data to be used for research and educational purposes. All patient data were anonymized prior to analysis to ensure confidentiality and protect patient privacy. Unique identifiers were removed or coded, and all personal information that could directly or indirectly identify patients was excluded. Data handling and storage complied with applicable data protection regulations, including the General Data Protection Regulation (GDPR).

### 2.4. Diagnostic Procedures

All patients underwent evaluation, including sensitization prick tests performed by trained personnel following standardized protocols. The prick tests included tree pollen, grass pollen, ragweed, mugwort, house dust mites, animal dander, molds, and food allergens. The test was performed on the volar aspect of the forearm using standard lancets and allergen extract (Lofarma S.p.A, Milan, Italy) according to the manufacturer’s instructions for use. Positive and negative controls were included with histamine and saline solutions, respectively. A wheal diameter of ≥3 mm was considered positive. Additionally, based on clinical context, patients were tested for drug and hymenoptera venom sensitization. In the present analysis, sensitization was defined based on skin prick test positivity. Because detailed clinical confirmation of allergic disease was not systematically evaluated for all patients, the results primarily reflect patterns of sensitization rather than clinically confirmed allergic disease.

Additional diagnostic tests performed included serum-specific IgE testing, pulmonary function tests, etc. Nevertheless, this data was not included in the current analysis, as it was not consistently assessed for all patients.

### 2.5. Data Collection

Clinical variables were derived from physician documentation and patient-reported symptoms recorded at the time of consultation. Asthma exacerbations were defined in accordance with the Global Initiative for Asthma (GINA) [16] as episodes of acute worsening of respiratory symptoms compared with the patient’s baseline condition requiring clinical evaluation. These were recorded as a binary variable (0 = absence of exacerbation, 1 = presence of exacerbation). The severity of allergic rhinitis symptoms was classified according to the Allergic Rhinitis and its Impact on Asthma (ARIA) recommendations [17,18], based on the documented clinical assessment of symptom burden. Symptom severity was recorded as an ordinal variable with three levels (0 = no symptoms, 1 = mild symptoms, 2 = moderate-to-severe symptoms). Because some patients presented outside the peak symptomatic period, variables reflect the subjective perception leading to healthcare presentation.

Data were anonymized before analysis.

### 2.6. Environmental Sensors for the Detection of Airborne Ragweed Pollen Concentration

Airborne bio-particle data were collected using real-time analyzers designed for continuous environmental monitoring of airborne biological particles (Lumbara Edge, Luxembourg, Luxembourg). The analyzers were supported by an energy module containing a 60 Ah battery, enabling autonomous operation. Depending on site-specific infrastructure, the devices were installed either within protective enclosures or connected directly to a 220 V external power supply to ensure uninterrupted data acquisition.

The bio-particle analyzers operate based on a machine vision algorithm that enables real-time capture and classification of airborne particles. Ambient air is continuously drawn into the system, and particulate matter, including pollen and dust, is collected using a specialized adhesive tape. Captured particles are then imaged and analyzed in real time, allowing automated detection and classification of airborne bio-particles.

Airborne ragweed pollen concentrations were recorded continuously and aggregated to daily mean values. For analyses involving clinical data, daily measurements were further aggregated to weekly mean concentrations to ensure temporal alignment with patient-based datasets.

All monitoring equipment was installed at 4 predefined locations in Timisoara (Romania) and the surrounding areas, forming a peri-urban airborne monitoring network, predominantly covering the northern sector of the study area, allowing representative assessment of airborne ragweed pollen exposure in clinically and environmentally relevant zones.

### 2.7. Statistical Analysis

Statistical analysis was performed using GraphPad Prism (version 10.5.0 (673)), R (version 4.5.0), RStudio (version 4.5.0), and Microsoft Excel (version 2025.05.0+496). Descriptive statistics were calculated for demographic and clinical characteristics. Continuous variables were expressed as means with 95% confidence intervals and categorical variables were presented as frequencies and percentages. Normality of continuous variables was assessed using the Shapiro–Wilk test. Group comparisons were conducted using appropriate statistical tests:

• The chi-square test for categorical variables;
• Student’s *t*-test for normally distributed continuous variables;
• The Mann–Whitney U test for non-normally distributed variables.

Multivariable logistic regression analysis was performed to evaluate factors associated with polysensitization, including age and sex as independent variables.

Associations between weekly ragweed burden and clinical variables were assessed using Spearman’s rank correlation. Correction for multiple testing was performed using the Holm method where appropriate. A *p*-value < 0.05 was considered statistically significant.

## 3. Results

### 3.1. Study Population and Baseline Characteristics

**Figure 1:**
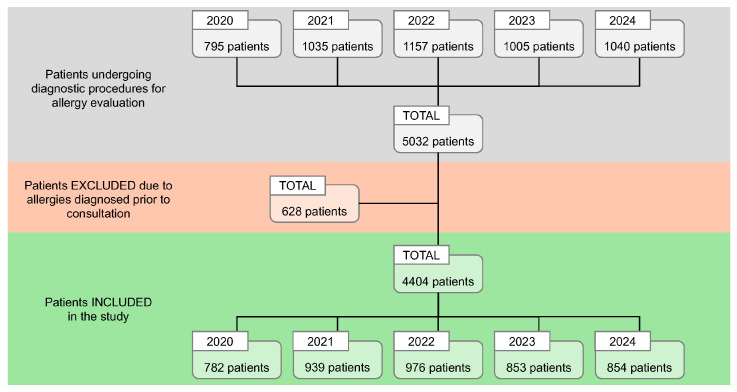
Flow diagram of patient selection, inclusion, and exclusion. Overview of the patient recruitment process for the 5-year study period (2020–2024) at the Allergology Department of the OncoGen Center, part of the “Pius Brinzeu” Clinical County Emergency Hospital.

During the 5-year study period (2020–2024), a total of 5032 patients were evaluated at the study center. Of these, 628 individuals were excluded due to a prior diagnosis of sensitizations, resulting in 4404 patients meeting the inclusion criteria and being retained for analysis (Figure 1). The annual distribution of included patients was relatively consistent, with: 782 patients in 2020, 939 patients in 2021, 976 patients in 2022, 853 patients in 2023, and 854 patients in 2024, reflecting a stable patient flow over the study period, despite the COVID-19 pandemic.

**Figure 2:**
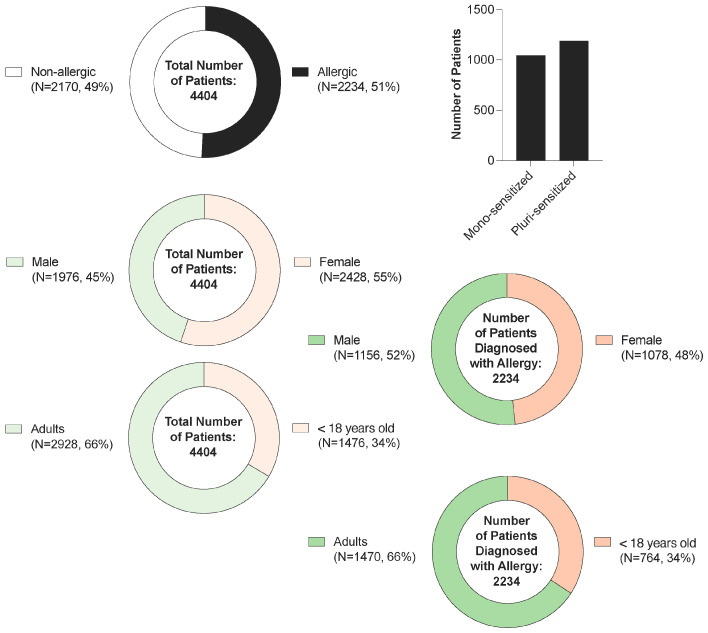
Demographic and sensitization profile of the study cohort. Distribution of patients by gender and allergen sensitization status for the patients diagnosed at the Allergology Department of the OncoGen Center, part of the “Pius Brinzeu” Clinical County Emergency Hospital.

The study cohort included a total of 4404 patients (see Supplementary Materials, Table S1), of whom 55.1% were female and 44.9% male (Figure 2). Sensitization to at least one allergen (tree pollen, grass pollen, ragweed, mugwort, house dust mites, animal dander, molds, food allergens, drugs, and hymenoptera) was identified in 50.7% of patients, indicating a nearly equal distribution between sensitized and non-sensitized individuals. Among sensitized subjects, males were slightly more represented (51.7%) compared to females (48.3%), suggesting a modest gender-related difference in sensitization prevalence. Regarding age, 33.5% of participants were under 18 years old and 66.5% were adults, with similar proportions observed within the sensitized group (34.2% vs. 65.8%, respectively), indicating no substantial age-related variation. Among those exhibiting sensitization, 46.7% were monosensitized while 53.3% displayed polysensitization, highlighting a slight predominance of multiple allergen reactivity in this population.

At the initial presentation, a statistically significant age difference (Table 1) was observed between male and female patients, with females being older than males (mean age 31.98 vs. 25.51 years, *p* < 0.0001). This sex-related age disparity persisted among sensitized patients, maintaining the same level of statistical significance (*p* < 0.0001). In contrast, among ragweed-sensitized patients, the age at diagnosis did not differ significantly between sexes (mean age 31.99 vs. 30.25 years; *p* = 0.0554), indicating a lack of the sex-dependent age difference within this specific sensitization subgroup.

### 3.2. Ragweed Sensitization

**Figure 3:**
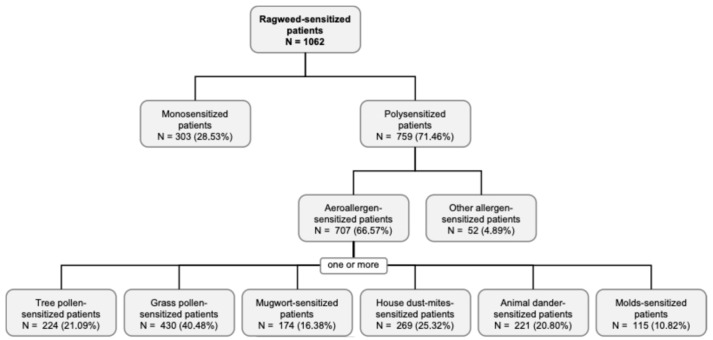
Flow diagram illustrating the distribution of mono- and polysensitization patterns among ragweed-sensitized patients (N = 1062). Patients were categorized according to the presence of single or multiple sensitizations, including aeroallergens and other allergen sources, with further subdivision by specific aeroallergen types. Percentages are calculated relative to the total ragweed-sensitized cohort or relevant subgroup. Patients belong to the cohort diagnosed at the Allergology Department of the OncoGen Center, part of the “Pius Brinzeu” Clinical County Emergency Hospital.

Among all patients included in this retrospective observational study, the ragweed sensitization has a prevalence of 24.11%. From the ragweed-sensitized patients, 303 individuals (28.53%) were monosensitized, while the majority, 759 patients (71.46%), exhibited polysensitization. Within the polysensitized group, most patients were sensitized to aeroallergens (*N* = 707; 66.57%), whereas a smaller proportion showed sensitization to other allergen categories (*N* = 52; 4.89%). Among aeroallergen-sensitized patients, sensitization to grass pollen (40.48%) and tree pollen (21.09%) was most frequent, followed by house dust mites (25.32%), animal dander (20.80%), mugwort (16.38%), and molds (10.82%), with several patients presenting sensitization to more than one aeroallergen source (Figure 3). These findings indicate a predominance of pollen-related co-sensitization, with a substantial contribution from indoor aeroallergens.

**Figure 4:**
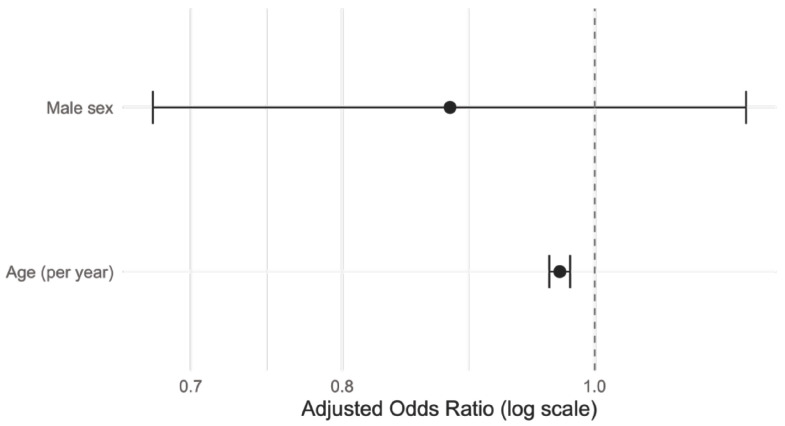
Adjusted odds ratios for polysensitization to other aeroallergens among ragweed-sensitized patients. Forest plot showing adjusted odds ratios (ORs) with 95% confidence intervals derived from multivariable logistic regression analysis including age and sex. The dashed vertical line indicates the null value (OR = 1). Patients belong to the cohort diagnosed at the Allergology Department of the OncoGen Center, part of the “Pius Brinzeu” Clinical County Emergency Hospital.

In multivariable logistic regression analysis (Figure 4) restricted to ragweed-sensitized patients (*N* = 1062), increasing age was independently associated with lower odds of polysensitization to other aeroallergens (adjusted OR = 0.97 per year, 95% CI: 0.96–0.98, β = −0.031, *p* < 0.001). Sex was not independently associated with polysensitization after adjustment for age (male vs. female: adjusted OR = 0.88, 95% CI: 0.68–1.14, *p* = 0.339).

Pairwise χ^2^ analyses revealed significant, non-random co-sensitization patterns among aeroallergens in ragweed-sensitized patients. Strong associations were observed within outdoor pollen allergens, particularly between tree pollen and grass pollen (χ^2^ = 145.8, *p* < 0.001), as well as between grass pollen and mugwort pollen (χ^2^ = 43.5, *p* < 0.001). Indoor aeroallergens also formed a distinct cluster, with significant associations between house dust mites and animal dander (χ^2^ = 30.0, *p* < 0.001) and between animal dander and molds (χ^2^ = 27.5, *p* < 0.001). Several cross-category associations were identified, notably between grass pollen and indoor allergens, suggesting a broader atopic sensitization profile in a subset of patients. All reported associations remained significant after Holm correction for multiple testing, except for tree pollen–house dust mites and mugwort–animal dander pairs, which showed no significant association (Table 2).

### 3.3. Seasonal Distribution of Ragweed-Sensitized Patients, Clinical Phenotype, and Airborne Ragweed Pollen Concentration

The weekly number of patients newly diagnosed with ragweed sensitization initially showed a moderate positive correlation with asthma exacerbation rates (Spearman ρ = 0.30, unadjusted *p* = 0.029); however, this association did not remain statistically significant after adjustment for multiple testing with Holm correction (Table 3). No significant correlations were observed between weekly ragweed burden and other aeroallergen sensitizations, total sensitization load, or nasal symptom severity scores.

**Figure 5:**
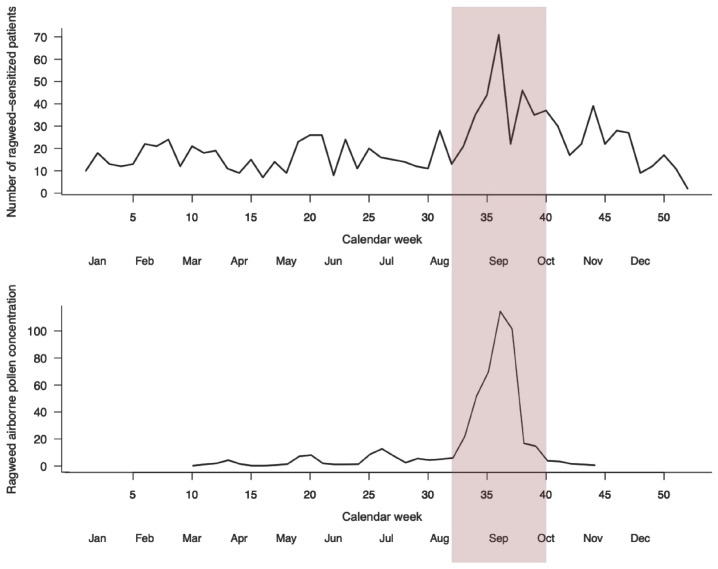
Weekly distribution of ragweed-sensitized patients and airborne ragweed pollen concentration [ppm^3^] across the study period. The upper panel shows the number of ragweed-sensitized patients newly diagnosed per calendar week, while the lower panel depicts weekly airborne ragweed pollen concentrations measured by environmental sensors. Calendar weeks (1–52) are shown on the x-axis, with the corresponding calendar months indicated below to facilitate interpretation of seasonal trends throughout the 5-year analysis. The shaded area highlights the peak ragweed pollen season (calendar weeks 33–38).

Weekly aggregation of clinical and environmental data revealed a seasonal pattern for ragweed sensitization (Figure 5). The number of ragweed-sensitized patients remained relatively stable at low levels during winter and spring, followed by a progressive increase starting in late summer and reaching a pronounced peak between calendar weeks 33 and 38 (mid-August to late-September). This peak coincided temporally with the highest airborne ragweed pollen concentrations detected by environmental sensors, which showed a sharp rise during the same interval and rapid decline thereafter.

Outside the ragweed pollen season, airborne pollen concentrations were negligible, and only modest fluctuations in the number of newly diagnosed ragweed-sensitized patients were observed. In contrast, during the peak pollen period, both clinical case counts and pollen concentrations increased markedly, with the highest weekly values occurring in early September. Following the end of the pollen season, a parallel decline was observed in both parameters, returning toward baseline levels by late autumn.

Overall, the temporal alignment between airborne ragweed pollen levels and the weekly number of newly diagnosed ragweed-sensitized patients supports a seasonal relationship, with the highest clinical burden occurring during periods of intense environmental exposure. Nevertheless, these findings represent a descriptive seasonal alignment between clinical presentation and airborne pollen exposure, whereas the statistical analyses presented in Table 3 did not demonstrate robust associations after correction for multiple testing.

### 3.4. Clinical Manifestation According to Sensitization Profile

**Figure 6:**
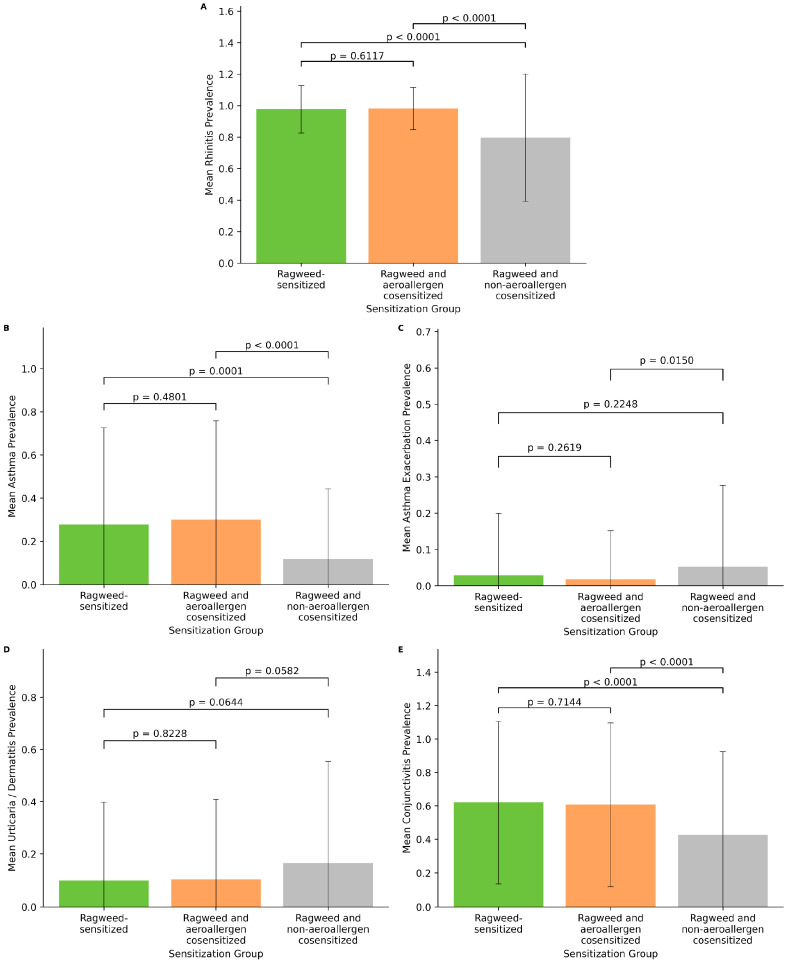
Clinical manifestations across different sensitization profiles in ragweed-sensitized patients. Mean prevalence of rhinitis (**A**), asthma (**B**), asthma exacerbation (**C**), urticaria/dermatitis (**D**), and conjunctivitis (**E**) among patients with ragweed sensitization alone, ragweed with aeroallergen cosensitization, and ragweed with non-aeroallergen cosensitization. Bars represent mean values, and error bars indicate standard deviation. Pairwise comparisons were conducted using the Mann–Whitney U test, with *p*-values indicated above each comparison.

The prevalence of major allergic clinical manifestations differed across sensitization profiles (Figure 6). Rhinitis was highly prevalent among all groups, with no significant difference between ragweed-sensitized patients and those cosensitized with aeroallergens (*p* = 0.6117). However, patients with ragweed and non-aeroallergen cosensitization showed a significantly lower prevalence of rhinitis compared with both ragweed-only and ragweed–aeroallergen cosensitized individuals (both *p* < 0.0001).

A similar pattern was observed for conjunctivitis, where no significant difference was identified between ragweed-only and ragweed–aeroallergen cosensitized patients (*p* = 0.7144), while the prevalence was significantly lower among patients with non-aeroallergen cosensitization (both *p* < 0.0001).

For asthma, no statistically significant difference was observed between ragweed-sensitized patients and those cosensitized with aeroallergens (*p* = 0.4801). In contrast, asthma prevalence was significantly lower in patients with ragweed and non-aeroallergen cosensitization compared with the other two groups (*p* = 0.0001 and *p* < 0.0001, respectively).

In contrast to these respiratory manifestations, asthma exacerbations were slightly more frequent among patients with ragweed and non-aeroallergen cosensitization. Although no significant differences were observed between ragweed-only and ragweed–aeroallergen cosensitized groups (*p* = 0.2619) or between ragweed-only and non-aeroallergen cosensitized patients (*p* = 0.2248), a significant difference was detected between ragweed–aeroallergen and ragweed–non-aeroallergen cosensitized groups (*p* = 0.0150).

Finally, urticaria/dermatitis showed a tendency toward higher prevalence among patients with ragweed and non-aeroallergen cosensitization; however, none of the pairwise comparisons reached statistical significance (*p* = 0.8228, *p* = 0.0644, and *p* = 0.0582).

## 4. Discussion

In this 5-year retrospective observational study, we provide a comprehensive epidemiological characterization of ragweed sensitization in western Romania by integrating large-scale clinical data with real-time aerobiological monitoring. Our findings confirm that ragweed sensitization represents a substantial and seasonally driven healthcare burden in this region, with nearly one quarter of all newly evaluated patients exhibiting sensitization to *Ambrosia artemisiifolia*. This prevalence is consistent with reports from other highly affected European regions and underscores the growing public health relevance of ragweed allergy in Central and Eastern Europe [1,4]. In the Black Sea region, a large retrospective study of 3000 patients identified ragweed as the most important seasonal allergen, with the strongest statistical association with asthma development (*p* = 6.69 × 10^−52^) [4]. Similarly, pediatric studies from Croatia reported remarkably high sensitization rates of 62.87% by skin prick test among children aged 4–17 years with allergic rhinitis [19], while Ukrainian cohorts documented 42.5% specific IgE positivity to ragweed among sensitized children and adolescents [20]. These regional variations in sensitization rates reflect differences in study populations, diagnostic methods, and local pollen exposure intensities, yet collectively demonstrate the substantial allergenic impact of ragweed across this European territory.

At the population level, a significant sex-related age difference was observed, with female patients presenting at older ages than male patients. This pattern persisted among sensitized individuals but was no longer evident in the ragweed-sensitized subgroup, suggesting that ragweed sensitization may occur within a narrower and more uniform age window across sexes. The older age at first specialist consultation observed among female patients in the overall cohort may reflect a combination of biological and behavioral factors rather than a delayed onset of sensitization. From a biological perspective, experimental and clinical evidence suggests that female sex hormones—particularly estrogens and progesterone—may exert immunomodulatory effects that can attenuate early inflammatory responses in allergic disease, potentially resulting in milder or less disruptive early clinical manifestations [21]. Estrogen has been shown to enhance epithelial barrier integrity and modulate mast cell and eosinophil activation, while progesterone may suppress excessive Th2-driven inflammation under certain conditions, collectively contributing to a more gradual symptom evolution, with a more severe exacerbation in women [21,22,23,24]. Such hormonal modulation could delay the perceived need for specialist evaluation despite ongoing sensitization [22,24]. This observation also aligns with previous reports indicating that allergen-specific IgE production to inhalant allergens tends to peak in childhood and young adulthood and decline with increasing age, albeit in an allergen-specific manner [25]. Tosca et al. showed that allergen-specific IgE responses to molecular components decrease with aging, although the magnitude and timing of this decline differ between allergens, highlighting the importance of age as a modifier of sensitization patterns and clinical expression [25]. Consistent with recent European pediatric literature, we observed substantial ragweed sensitization among school-age children. A Croatian study of 412 children aged 4–17 years reported 62.9% skin prick test positivity and 66.0% specific IgE positivity to ragweed [19], while retrospective Ukrainian data from 892 children identified weed pollen as the dominant allergen group (86.6% of cases), with ragweed among the leading species [9]. These high pediatric sensitization rates are concerning, as early-life sensitization to potent aeroallergens like ragweed may influence the trajectory of allergic disease and increase the risk of asthma development [20]. Our multivariable analysis further supports this concept, as increasing age was independently associated with lower odds of polysensitization to other aeroallergens among ragweed-sensitized patients. This finding suggests that younger individuals may exhibit a broader atopic profile, whereas sensitization patterns in older patients may become more restricted, potentially reflecting immunosenescence, cumulative exposure history, or changes in immune regulation over time.

The high prevalence of polysensitization observed among ragweed-sensitized patients in our cohort was lower than the values reported by previous clinical and molecular studies [7]. Ragweed allergy is rarely an isolated phenomenon and is frequently accompanied by sensitization to other seasonal pollens and indoor aeroallergens, with some studies even reporting polysensitization rates exceeding 99% among ragweed-sensitized patients [7]. Pairwise χ^2^ analyses revealed distinct and biologically plausible co-sensitization clusters, with strong associations among outdoor pollen allergens (tree, grass, and mugwort) and among indoor allergens (house dust mites, animal dander, and molds). These findings support the concept of shared exposure environments and overlapping sensitization pathways, rather than random co-occurrence. The key finding of our study, quoting polysensitization among ragweed-sensitized patients at 62.65%, aligns closely with the results of the Po Valley longitudinal cohort, where 66% of pollen-positive patients exhibited polysensitization to two or more pollen types [2], while pediatric series across Eastern Europe consistently report frequent co-sensitization to ragweed alongside other weed and grass pollens [8,9]. The clinical implications of polysensitization are substantial, as complex sensitization profiles can complicate diagnosis, influence disease severity, and affect immunotherapy selection and efficacy [14,20].

From a molecular perspective, ragweed sensitization is characterized by complex IgE profiles with important diagnostic and therapeutic implications [9,14]. While Amb a 1 remains the dominant allergen and accounts for the majority of ragweed-specific IgE responses, a substantial proportion of patients exhibit sensitization to additional ragweed components, including profilin and calcium-binding proteins such as Amb a 9 and Amb a 10 [3,9,14,26]. CRD studies in Romanian cohorts have demonstrated IgE reactivity to Amb a 9 and Amb a 10 in approximately 25–35% of ragweed-allergic patients, with distinct clinical phenotypes associated with polcalcin sensitization [14]. Polcalcins are pan-allergens that cross-react with homologous proteins from other plant sources, particularly Art v 15 derived from mugwort (*Artemisia vulgaris*), and may explain some patterns of polysensitization observed in the region [8,9]. Although molecular diagnostics were not included in the present analysis, the high degree of polysensitization observed at the extract level suggests underlying molecular complexity that may influence symptom severity, cross-reactivity, and treatment response.

Beyond sensitization patterns, our analysis also explored how different sensitization profiles were associated with clinical manifestations. Patients with ragweed sensitization alone and those cosensitized with other aeroallergens showed very similar prevalence of rhinitis, conjunctivitis, and asthma, suggesting that additional aeroallergen sensitization does not necessarily translate into a substantially different clinical phenotype at the population level. In contrast, patients with ragweed sensitization combined with non-aeroallergen sensitization exhibited a lower prevalence of upper and lower respiratory manifestations, including rhinitis, conjunctivitis, and asthma. This observation may reflect differences in exposure pathways and immunologic mechanisms between inhalant and non-inhalant allergens. Aeroallergen cosensitization typically results from shared environmental exposure and overlapping seasonal triggers, whereas non-aeroallergen sensitization may reflect independent atopic pathways or food-related sensitization patterns that do not directly contribute to respiratory symptom burden. Interestingly, asthma exacerbations were slightly more frequent in the group with non-aeroallergen cosensitization, although the differences were modest. While these findings should be interpreted cautiously due to the cross-sectional design, they suggest that the clinical expression of ragweed sensitization may vary depending on the broader atopic profile of the patient. Future studies incorporating molecular allergen diagnostics and longitudinal clinical assessment could further clarify how specific sensitization patterns influence symptom severity and disease trajectory.

By integrating clinical data with continuous aerobiological monitoring, our study demonstrates a pronounced seasonal pattern of ragweed sensitization, with a clear peak between calendar weeks 33 and 38, corresponding approximately to mid-August through late September. The temporal trends observed in our study parallel longitudinal data from other European regions. A landmark 33-year study from the Po Valley, Italy (1986–2019), documented a progressive increase in ragweed pollen load, extension of the pollen season, and rising sensitization rates among respiratory-allergic patients, concomitant with a regional temperature increase of 1.4 °C [1]. This long-term evidence supports the hypothesis that climate change is facilitating ragweed expansion and intensifying allergen exposure across Europe [11]. Eight-year pollen monitoring data from Bucharest similarly documented rapid local spread and increasing biological pollution from ragweed, with pollen concentrations correlated to both air pollutant levels and clinical disease burden [10]. These findings underscore the need for sustained aerobiologic surveillance and integrated public health responses to address the expanding ragweed threat.

Weekly ragweed burden showed a moderate positive correlation with asthma exacerbation rates. Although this association did not remain statistically significant after correction for multiple testing, overall, the absence of statistically significant associations after correction indicates that weekly fluctuations in ragweed pollen burden alone were insufficient to explain variability in clinical outcomes at the population level. It is biologically plausible and consistent with the established role of ragweed pollen as a potent trigger of lower airway inflammation and asthma instability [27]. Notably, no significant associations were observed between weekly ragweed burden and nasal symptom severity or overall polysensitization, suggesting a more selective impact of ragweed exposure on lower airway manifestations rather than a generalized amplification of allergic symptoms [7]. This selective association may reflect differential tissue susceptibility, exposure thresholds, or lagged effects that are not fully captured by weekly data aggregation.

Several limitations should be acknowledged when interpreting the findings of the present study. First, the present analysis was restricted to ragweed-sensitized patients, precluding direct comparison with non–ragweed-sensitized individuals. Inclusion of appropriate control groups in future investigations would allow more precise identification of factors independently associated with ragweed sensitization and the progression of allergic disease. Second, the retrospective cross-sectional design limits causal inference and does not allow determination of the temporal sequence between environmental exposure, sensitization, and clinical symptom development. In addition, hospital-based datasets may be influenced by referral patterns and documentation variability. Because the study was conducted in a tertiary referral allergology center, the cohort likely represents a selected symptomatic population rather than the general population, as patients were typically referred from primary or secondary care settings for specialized evaluation. This may result in an overrepresentation of individuals with more severe or persistent symptoms, and therefore the observed sensitization patterns should be interpreted within the context of a clinically referred population rather than as population-level estimates. Third, the temporal analysis linking weekly diagnoses to airborne pollen concentrations reflects patterns of symptom-driven healthcare presentation rather than the true onset of immunologic sensitization, which may precede clinical evaluation by months or years. Consequently, the observed seasonal patterns should be interpreted as indicators of healthcare utilization during periods of increased allergen exposure rather than direct measures of disease incidence. Finally, molecular allergen diagnostics and component-resolved diagnosis were not incorporated into the present analysis, which could have provided a more refined characterization of sensitization profiles and cross-reactivity patterns. In addition, detailed environmental covariates—including meteorological parameters, air pollution levels, and viral respiratory infections—were not included in the statistical models. These factors may contribute substantially to variability in allergic symptoms and asthma exacerbations and should be considered in future longitudinal studies integrating environmental, molecular, and clinical datasets.

Despite these limitations, this study provides robust epidemiological evidence that ragweed sensitization in western Romania is common, frequently polysensitized, and strongly seasonally driven, with close temporal alignment between airborne pollen exposure and clinical presentation. The integration of clinical surveillance with aerobiological monitoring represents a valuable framework for improving risk stratification, guiding public health interventions, and informing patient management strategies in regions increasingly affected by ragweed sensitization and allergy.

## 5. Conclusions

Ragweed sensitization represents a substantial and strongly seasonal healthcare burden in western Romania, affecting nearly one quarter of newly evaluated patients. Sensitization to ragweed was frequently accompanied by polysensitization to other aeroallergens, particularly pollens, with younger age independently associated with broader sensitization profiles. Distinct clinical manifestation patterns were observed according to sensitization profile, with similar prevalence of rhinitis, conjunctivitis, and asthma among ragweed-only and ragweed–aeroallergen cosensitized patients, whereas individuals with additional non-aeroallergen sensitization showed lower prevalence of respiratory manifestations but a tendency toward more frequent asthma exacerbations. The pronounced late-summer peak in ragweed-sensitized cases closely overlapped with airborne pollen concentrations, supporting a clear exposure–disease relationship. Although associations with asthma exacerbations did not remain statistically significant after correction for multiple testing, the observed temporal patterns were biologically plausible. Integrating clinical surveillance with continuous aerobiological monitoring provides a valuable framework for risk assessment and supports targeted public health and clinical interventions in regions increasingly impacted by ragweed sensitization and allergy.

## Figures and Tables

**Table 1:** Age distribution of patients according to sensitization status and sex for the patients diagnosed at the Allergology Department of the OncoGen Center, part of the “Pius Brinzeu” Clinical County Emergency Hospital.

Cohort	Age [95% CI] [Years] (Total Number of Patients)	Number of Male Patients Age [95% CI] [Years]	Number of Female Patients Age [95% CI] [Years]
All patients	29.08 [95% CI (28.51–29.64)]	25.51 [95% CI (24.70–26.32)]	31.98 [95% CI (31.22–32.74)]
	(N = 4404)	(N = 1976)	(N = 2428)
Non-sensitized patients	30.06 [95% CI (29.19–30.93)]	25.57 [95% CI (24.17–26.98)]	32.79 [95% CI (31.70–33.87)]
	(N = 2170)	(N = 820)	(N = 1350)
Sensitized patients	28.12 [95% CI (27.40–28.84)]	25.46 [95% CI (24.49–26.43)]	30.97 [95% CI (29.93–32.01)]
	(N = 2235)	(N = 1157)	(N = 1078)
Monosensitized patients	29.02 [95% CI (27.90–30.14)]	25.36 [95% CI (23.84–26.87)]	32.98 [95% CI (31.38–34.59)]
	(N = 1041)	(N = 541)	(N = 500)
Polysensitized patients	27.41 [95% CI (26.49–28.32)]	25.69 [95% CI (24.45–26.93)]	29.23 [95% CI (27.89–30.57)]
	(N = 1194)	(N = 616)	(N = 578)
All aeroallergen sensitized patients	27.73 [95% CI (27.00–28.47)]	25.56 [95% CI (24.57–26.54)]	30.22 [95% CI (29.14–31.30)]
	(N = 1907)	(N = 1017)	(N = 890)
Aeroallergen monosensitized patients	28.42 [95% CI (27.29–29.56)]	25.37 [95% CI (23.87–26.87)]	32.14 [95% CI (30.47–33.81)]
	(N = 856)	(N = 470)	(N = 386)
Aeroallergen polysensitized patients	27.17 [95% CI (26.21–28.13)]	25.71 [95% CI (24.42–27.01)]	28.75 [95% CI (27.34–30.16)]
	(N = 1051)	(N = 547)	(N = 504)
Ragweed sensitized patients	31.07 [95% CI (30.18–31.96)]	30.25 [95% CI (29.03–31.46)]	31.99 [95% CI (30.68–33.31)]
	(N = 1062)	(N = 562)	(N = 500)

**Table 2:** Pairwise associations between aeroallergen co-sensitizations among ragweed-sensitized patients, through Pearson’s χ^2^ test with Yates’ continuity correction and Holm adjustment for multiple testing. Patients belong to the cohort diagnosed at the Allergology Department of the OncoGen Center, part of the “Pius Brinzeu” Clinical County Emergency Hospital.

Aeroallergen 1	Aeroallergen 2	χ^2^	*p*-Value	Adjusted *p*-Value (Holm)
Tree pollen	Grass pollen	145.81	<0.001	<0.001
Tree pollen	Mugwort pollen	21.47	<0.001	<0.001
Tree pollen	House dust mites	0.42	0.515	0.515
Tree pollen	Animal dander	17.98	<0.001	<0.001
Tree pollen	Molds	17.42	<0.001	<0.001
Grass pollen	Mugwort pollen	43.49	<0.001	<0.001
Grass pollen	House dust mites	20.61	<0.001	<0.001
Grass pollen	Animal dander	22.82	<0.001	<0.001
Grass pollen	Molds	27.23	<0.001	<0.001
Mugwort pollen	House dust mites	6.55	0.010	0.042
Mugwort pollen	Animal dander	2.87	0.090	0.181
Mugwort pollen	Molds	5.34	0.021	0.063
House dust mites	Animal dander	30.02	<0.001	<0.001
House dust mites	Molds	10.65	0.001	0.006
Animal dander	Molds	27.53	<0.001	<0.001

**Table 3:** Correlation between weekly ragweed-sensitization burden and clinical variables derived from patient-reported symptoms and physician documentation. Spearman rank correlation analysis of weekly aggregated data (5-year overlap). Patients belong to the cohort diagnosed at the Allergology Department of the OncoGen Center, part of the “Pius Brinzeu” Clinical County Emergency Hospital.

Variable	Spearman’s ρ	*p*-Value	Adjusted *p*-Value (Holm)
Asthma exacerbation	0.303	0.029	0.549
Tree pollen sensitization	−0.188	0.181	1.000
Grass pollen sensitization	−0.152	0.281	1.000
Mugwort pollen sensitization	0.035	0.804	1.000
House dust mite sensitization	−0.035	0.804	1.000
Animal dander sensitization	−0.117	0.409	1.000
Mold sensitization	0.170	0.227	1.000
Number of aeroallergens	−0.114	0.421	1.000
Total sensitization burden	−0.085	0.549	1.000
Rhinitis	−0.143	0.310	1.000
Conjunctivitis	0.234	0.095	1.000
Asthma (overall diagnosis)	0.067	0.639	1.000
Urticaria/dermatitis	−0.235	0.093	1.000
Symptom severity score	−0.117	0.407	1.000
Number of symptoms	0.139	0.327	1.000
Pruritus severity	0.199	0.158	1.000
Nasal obstruction severity	−0.009	0.950	1.000
Rhinorrhea severity	0.025	0.859	1.000
Sneezing severity	0.001	0.992	1.000

Note: Weekly ragweed burden was defined as the number of ragweed-sensitized patients diagnosed per calendar week, aggregated across all study years. Binary variables are expressed as weekly proportions and continuous variables as weekly means. Associations were assessed using Spearman’s rank correlation. Holm correction was applied to account for multiple testing.

## Data Availability

The original contributions presented in this study are included in the article and Supplementary Materials. Further inquiries can be directed to the corresponding authors.

## Supplementary Materials

The following supporting information can be downloaded at: https://www.mdpi.com/article/10.3390/life16030526/s1, Supplementary Materials Table S1: Anonymized patient data.
