# Supplementary material for: Airborne Ragweed (Ambrosia artemisiifolia) Allergen Exposure and Sensitization Pattern in Western Romania: A 5-Year Retrospective Cross-Sectional Observational Analysis of Sensitization Prevalence, Complemented by a Parallel Temporal Analysis of Aerobiological Data and Symptom-Driven Healthcare Presentation Patterns Study
Source: Life (Basel). 2026 Mar 22;16(3):526. doi: 10.3390/life16030526 (PMC13028212; doi:10.3390/life16030526)
Supplement: Supplementary file 1 [file life-16-00526-s001.zip › Table S1. Anonymized patient data..pdf]

Table S1. Anonymized patient data.

Date of presentation	Sex	Age	Tree pollen	Grass pollen	Ragweed pollen	Mugwort pollen	House dust mites	Animal dander	Molds	Food allergens	Drugs	Insect venom	Rhinitis	Conjuncti-vitis	Asthma	Asthma exacer-bation	Urticaria / Dermatitis
08/01/2020	M	0	0	0	0	0	0	0	0	0	0	0	0	0	0	0	0
23/01/2020	M	0	0	0	0	0	0	0	0	0	0	0	0	0	0	0	0
18/02/2020	F	0	0	0	0	0	0	0	0	0	0	0	0	0	0	0	1
09/03/2020	M	0	0	0	0	0	0	0	0	0	0	0	0	0	0	0	1
09/03/2020	M	0	0	0	0	0	0	0	0	0	0	0	0	0	0	0	1
31/07/2020	M	0	0	0	0	0	0	0	0	0	0	0	1	0	0	0	0
17/09/2020	M	0	0	0	0	0	0	0	0	0	0	0	0	0	0	0	0
25/09/2020	M	0	0	0	0	0	0	0	0	0	0	0	0	0	0	0	0
30/09/2020	M	0	0	0	0	0	0	0	0	0	0	0	0	0	0	0	0
12/10/2020	M	0	0	0	0	0	0	0	0	0	0	0	0	0	0	0	1
17.03.2021	M	0	0	0	0	0	0	0	0	0	0	0	1	0	0	0	0
21.04.2021	M	0	0	0	0	0	0	0	0	0	0	0	0	0	0	1	0
24.03.2021	F	0	0	0	0	0	0	0	0	0	0	0	0	0	1	0	1
26/05/2022	F	0	0	0	0	0	0	0	0	0	0	0	0	0	0	0	0
01/08/2022	F	0	0	0	0	0	0	0	0	0	0	0	0	0	0	0	0
21/09/2022	F	0	0	0	0	0	0	0	0	0	0	0	0	0	0	0	1
06/10/2022	M	0	0	0	0	0	0	0	0	0	0	0	0	0	0	0	0
06/12/2022	M	0	0	0	0	0	0	0	0	0	0	0	0	0	0	0	0
03/02/2023	F	0	0	0	0	0	0	0	0	0	0	0	0	0	0	0	1
08/02/2023	F	0	0	0	0	0	0	0	0	0	0	0	0	0	0	0	1
17/02/2023	M	0	0	0	0	0	0	0	0	0	0	0	0	0	0	0	1
24/02/2023	M	0	0	0	0	0	0	0	0	0	0	0	1	0	0	0	0
22/03/2023	M	0	0	0	0	0	0	0	0	0	0	0	0	0	0	0	1
29/03/2023	F	0	0	0	0	0	0	0	0	0	0	0	0	0	0	0	1
05/04/2023	F	0	0	0	0	0	0	0	0	0	0	0	0	0	0	0	1

10/05/2023	M	0	0	0	0	0	0	0	0	0	0	0	1	0	0	0	0
19/05/2023	M	0	0	0	0	0	0	0	0	0	0	0	0	0	0	0	1
28/06/2023	M	0	0	0	0	0	0	0	0	0	0	0	0	0	0	0	1
19/07/2023	M	0	0	0	0	0	0	0	0	0	0	0	1	0	0	0	0
24/07/2023	F	0	0	0	0	0	0	0	0	0	0	0	0	0	0	0	1
28/07/2023	M	0	0	0	0	0	0	0	0	0	0	0	0	0	0	0	1
30/08/2023	F	0	0	0	0	0	0	0	0	0	0	0	0	0	0	0	1
08/02/2024	M	0	0	0	0	0	0	0	0	0	0	0	0	0	0	0	1
20/03/2024	F	0	0	0	0	0	0	0	0	0	0	0	0	0	0	0	1
02/04/2024	F	0	0	0	0	0	0	0	0	0	0	0	0	0	0	0	0
19/04/2024	M	0	0	0	0	0	0	0	0	0	0	0	0	0	0	0	1
25/06/2024	M	0	0	0	0	0	0	0	0	0	0	0	0	0	0	0	1
26/06/2024	F	0	0	0	0	0	0	0	0	0	0	0	0	0	0	0	1
03/07/2024	F	0	0	0	0	0	0	0	0	0	0	0	0	0	0	0	1
09/07/2024	F	0	0	0	0	0	0	0	0	0	0	0	0	0	0	0	1
23/07/2024	M	0	0	0	0	0	0	0	0	0	0	0	0	0	0	0	1
30/07/2024	F	0	0	0	0	0	0	0	0	0	0	0	0	0	0	0	1
02/08/2024	M	0	0	0	0	0	0	0	0	0	0	0	0	0	0	0	1
16/10/2024	M	0	0	0	0	0	0	0	0	0	0	0	1	0	0	0	0
30/10/2024	M	0	0	0	0	0	0	0	0	0	0	0	0	0	0	0	1
15/11/2024	M	0	0	0	0	0	0	0	0	0	0	0	0	0	0	0	1
19/11/2024	M	0	0	0	0	0	0	0	0	0	0	0	0	0	0	0	0
31/08/2020	M	0	0	0	0	0	0	0	0	1	0	0	0	0	0	0	0
12/10/2020	F	0	0	0	0	0	0	0	0	1	0	0	0	0	0	0	0
26.11.2021	F	0	0	0	0	0	0	0	0	0	0	1	0	0	0	0	0
27.01.2021	F	0	0	0	0	0	0	0	0	1	0	0	0	0	0	0	0
07/06/2023	M	0	0	0	0	0	0	0	0	1	0	0	0	0	0	0	1
11/01/2024	M	0	0	0	0	0	0	0	0	1	0	0	0	0	0	0	1
12/04/2024	M	0	0	0	0	0	0	0	0	1	0	0	0	0	0	0	1

04/02/2020	M	1	0	0	0	0	0	0	0	0	0	0	1	0	0	0	1
11/02/2020	M	1	0	0	0	0	0	0	0	0	0	0	0	0	0	0	0
19/02/2020	M	1	0	0	0	0	0	0	0	0	0	0	0	0	0	0	0
26/05/2020	M	1	0	0	0	0	0	0	0	0	0	0	0	0	0	0	0
01/07/2020	M	1	0	0	0	0	0	0	0	0	0	0	0	0	0	0	0
11/08/2020	M	1	0	0	0	0	0	0	0	0	0	0	0	0	0	0	0
14/10/2020	M	1	0	0	0	0	0	0	0	0	0	0	0	0	0	0	0
06/11/2020	M	1	0	0	0	0	0	0	0	0	0	0	0	0	0	0	0
23/11/2020	F	1	0	0	0	0	0	0	0	0	0	0	0	0	0	0	0
07/12/2020	F	1	0	0	0	0	0	0	0	0	0	0	0	0	0	0	0
08/04/2021	M	1	0	0	0	0	0	0	0	0	0	0	0	0	0	0	1
18/05/2021	M	1	0	0	0	0	0	0	0	0	0	0	1	0	0	0	0
08/06/2021	M	1	0	0	0	0	0	0	0	0	0	0	0	0	0	0	1
22/09/2021	F	1	0	0	0	0	0	0	0	0	0	0	0	0	0	0	0
05/10/2021	F	1	0	0	0	0	0	0	0	0	0	0	0	0	0	0	1
03.12.2021	M	1	0	0	0	0	0	0	0	0	0	0	0	0	0	1	0
10.02.2021	F	1	0	0	0	0	0	0	0	0	0	0	0	0	1	0	1
15.10.2021	M	1	0	0	0	0	0	0	0	0	0	0	0	0	0	1	0
17.11.2021	M	1	0	0	0	0	0	0	0	0	0	0	0	0	0	0	0
21.12.2021	M	1	0	0	0	0	0	0	0	0	0	0	0	0	0	1	0
24.02.2021	M	1	0	0	0	0	0	0	0	0	0	0	1	0	1	0	1
26.05.2021	M	1	0	0	0	0	0	0	0	0	0	0	0	0	0	1	0
26/01/2022	M	1	0	0	0	0	0	0	0	0	0	0	1	0	0	0	1
26/01/2022	F	1	0	0	0	0	0	0	0	0	0	0	0	0	0	0	0
09/02/2022	M	1	0	0	0	0	0	0	0	0	0	0	0	0	0	0	1
15/04/2022	M	1	0	0	0	0	0	0	0	0	0	0	0	0	0	0	0
10/05/2022	M	1	0	0	0	0	0	0	0	0	0	0	0	0	0	0	0
11/05/2022	F	1	0	0	0	0	0	0	0	0	0	0	1	0	0	0	0
13/05/2022	M	1	0	0	0	0	0	0	0	0	0	0	0	0	0	0	0

27/05/2022	F	1	0	0	0	0	0	0	0	0	0	0	0	0	0	0	1
08/06/2022	F	1	0	0	0	0	0	0	0	0	0	0	0	0	0	0	0
27/06/2022	F	1	0	0	0	0	0	0	0	0	0	0	0	0	0	0	0
01/08/2022	M	1	0	0	0	0	0	0	0	0	0	0	0	0	0	0	1
29/09/2022	M	1	0	0	0	0	0	0	0	0	0	0	0	0	0	0	0
30/09/2022	F	1	0	0	0	0	0	0	0	0	0	0	1	0	0	0	0
30/09/2022	F	1	0	0	0	0	0	0	0	0	0	0	0	0	0	0	0
17/10/2022	F	1	0	0	0	0	0	0	0	0	0	0	1	0	0	0	0
07/11/2022	F	1	0	0	0	0	0	0	0	0	0	0	0	0	0	0	0
21/11/2022	F	1	0	0	0	0	0	0	0	0	0	0	0	0	0	0	0
25/11/2022	M	1	0	0	0	0	0	0	0	0	0	0	0	0	0	0	1
16/12/2022	M	1	0	0	0	0	0	0	0	0	0	0	0	0	0	0	1
16/12/2022	M	1	0	0	0	0	0	0	0	0	0	0	0	0	0	0	1
16/12/2022	M	1	0	0	0	0	0	0	0	0	0	0	0	0	0	0	0
21/12/2022	M	1	0	0	0	0	0	0	0	0	0	0	0	0	0	0	0
10/02/2023	M	1	0	0	0	0	0	0	0	0	0	0	0	0	0	0	1
01/03/2023	M	1	0	0	0	0	0	0	0	0	0	0	1	0	0	0	1
13/04/2023	F	1	0	0	0	0	0	0	0	0	0	0	0	0	0	0	1
08/05/2023	F	1	0	0	0	0	0	0	0	0	0	0	1	0	0	0	0
09/05/2023	F	1	0	0	0	0	0	0	0	0	0	0	0	0	0	0	1
16/05/2023	F	1	0	0	0	0	0	0	0	0	0	0	1	0	0	0	0
31/05/2023	F	1	0	0	0	0	0	0	0	0	0	0	0	0	0	0	1
15/06/2023	M	1	0	0	0	0	0	0	0	0	0	0	1	0	0	0	0
10/08/2023	M	1	0	0	0	0	0	0	0	0	0	0	1	0	0	0	0
30/08/2023	M	1	0	0	0	0	0	0	0	0	0	0	0	0	0	0	1
27/11/2023	M	1	0	0	0	0	0	0	0	0	0	0	1	0	0	0	0
11/12/2023	M	1	0	0	0	0	0	0	0	0	0	0	1	0	0	0	1
08/01/2024	M	1	0	0	0	0	0	0	0	0	0	0	0	0	0	1	0
15/02/2024	F	1	0	0	0	0	0	0	0	0	0	0	0	0	0	0	0

21/02/2024	F	1	0	0	0	0	0	0	0	0	0	0	0	0	0	0	1
22/03/2024	M	1	0	0	0	0	0	0	0	0	0	0	0	0	0	0	0
13/05/2024	M	1	0	0	0	0	0	0	0	0	0	0	0	0	0	0	0
22/05/2024	M	1	0	0	0	0	0	0	0	0	0	0	0	0	0	0	1
24/05/2024	M	1	0	0	0	0	0	0	0	0	0	0	0	0	0	0	1
20/08/2024	M	1	0	0	0	0	0	0	0	0	0	0	0	0	0	0	1
17/09/2024	M	1	0	0	0	0	0	0	0	0	0	0	0	0	0	0	0
18/12/2024	M	1	0	0	0	0	0	0	0	0	0	0	0	0	0	0	1
19/12/2024	F	1	0	0	0	0	0	0	0	0	0	0	0	0	0	0	0
08/10/2020	F	1	0	0	0	0	0	0	0	1	0	0	0	0	0	0	0
01/09/2021	F	1	0	0	0	0	0	0	0	1	0	0	0	0	0	1	0
23/09/2021	M	1	0	0	0	0	0	0	0	1	0	0	0	0	0	0	1
08.10.2021	F	1	0	0	0	0	0	0	0	1	0	0	0	0	0	1	0
12.05.2021	M	1	0	0	0	0	0	0	0	0	0	1	0	0	0	1	0
14.05.2021	F	1	0	0	0	0	0	0	0	1	0	0	0	0	0	1	0
15.12.2021	F	1	0	0	0	0	0	0	0	1	0	0	0	0	0	1	0
15.12.2021	M	1	0	0	0	0	0	0	0	0	0	1	0	0	0	0	0
27.08.2021	F	1	0	0	0	0	0	0	0	0	0	1	0	0	0	0	0
09/02/2022	M	1	0	0	0	0	0	0	0	1	0	0	1	0	0	0	1
18/11/2022	F	1	0	0	0	0	0	0	0	1	0	0	0	0	0	0	0
25/11/2022	M	1	0	0	0	0	0	0	0	1	0	0	0	0	0	0	0
09/01/2023	M	1	0	0	0	0	0	0	0	1	0	0	0	0	0	0	1
01/02/2023	M	1	0	0	0	0	0	0	0	1	0	0	0	0	0	0	1
28/03/2023	F	1	0	0	0	0	0	0	0	1	0	0	0	0	0	0	1
06/04/2023	M	1	0	0	0	0	0	0	0	1	0	0	0	0	0	0	0
20/04/2023	M	1	0	0	0	0	0	0	0	1	0	0	1	0	0	0	1
30/10/2023	F	1	0	0	0	0	0	0	0	1	0	0	0	0	0	0	1
28/11/2023	F	1	0	0	0	0	0	0	0	1	0	0	1	0	0	0	1
15/02/2024	M	1	0	0	0	0	0	0	0	1	0	0	0	0	0	0	0

19/02/2024	M	1	0	0	0	0	0	0	0	0	1	0	0	0	0	0	0
15/11/2024	M	1	0	0	0	0	0	0	0	1	0	0	0	0	0	0	0
08/12/2020	M	1	0	0	0	0	1	0	0	0	0	0	1	0	0	0	0
26/09/2023	M	1	0	0	1	0	0	0	0	0	0	0	1	0	0	0	0
21/04/2023	M	1	0	1	0	0	0	0	0	0	0	0	1	0	0	0	0
28/06/2023	M	1	0	0	0	0	0	0	1	1	0	0	0	0	0	0	1
29/10/2020	F	1	0	1	1	0	0	0	0	0	0	0	1	0	0	0	0
18/01/2021	M	1	0	0	0	0	1	1	0	1	0	0	0	0	0	0	1
13/01/2020	F	2	0	0	0	0	0	0	0	0	0	0	0	0	0	0	0
22/01/2020	M	2	0	0	0	0	0	0	0	0	0	0	0	0	0	0	1
29/01/2020	F	2	0	0	0	0	0	0	0	0	0	0	0	0	0	0	0
31/01/2020	M	2	0	0	0	0	0	0	0	0	0	0	1	0	0	0	0
10/08/2020	M	2	0	0	0	0	0	0	0	0	0	0	0	0	0	0	0
31/08/2020	M	2	0	0	0	0	0	0	0	0	0	0	1	1	0	0	0
07/09/2020	F	2	0	0	0	0	0	0	0	0	0	0	0	0	0	0	1
17/09/2020	M	2	0	0	0	0	0	0	0	0	0	0	1	0	0	0	0
30/09/2020	F	2	0	0	0	0	0	0	0	0	0	0	0	0	0	0	0
28/06/2021	F	2	0	0	0	0	0	0	0	0	0	0	1	0	0	0	0
29/07/2021	F	2	0	0	0	0	0	0	0	0	0	0	0	0	0	0	0
13/09/2021	F	2	0	0	0	0	0	0	0	0	0	0	1	1	0	0	0
22/09/2021	M	2	0	0	0	0	0	0	0	0	0	0	0	0	0	1	0
27/09/2021	M	2	0	0	0	0	0	0	0	0	0	0	0	0	0	0	1
18/10/2021	M	2	0	0	0	0	0	0	0	0	0	0	0	0	0	0	1
20/10/2021	F	2	0	0	0	0	0	0	0	0	0	0	0	0	0	0	1
29/11/2021	F	2	0	0	0	0	0	0	0	0	0	0	0	0	0	0	1
20/12/2021	M	2	0	0	0	0	0	0	0	0	0	0	0	0	0	0	1
04.06.2021	F	2	0	0	0	0	0	0	0	0	0	0	0	0	0	1	0
10.02.2021	F	2	0	0	0	0	0	0	0	0	0	0	0	0	0	0	0
15.01.2021	M	2	0	0	0	0	0	0	0	0	0	0	0	0	0	0	1

15.12.2021	F	2	0	0	0	0	0	0	0	0	0	0	0	0	0	1	0
23.13.2020	M	2	0	0	0	0	0	0	0	0	0	0	1	0	0	0	1
29.09.2021	F	2	0	0	0	0	0	0	0	0	0	0	0	0	0	0	0
14/01/2022	M	2	0	0	0	0	0	0	0	0	0	0	0	0	0	0	0
19/01/2022	F	2	0	0	0	0	0	0	0	0	0	0	0	0	0	0	1
22/02/2022	M	2	0	0	0	0	0	0	0	0	0	0	1	0	0	0	0
11/03/2022	F	2	0	0	0	0	0	0	0	0	0	0	0	0	0	0	0
17/03/2022	F	2	0	0	0	0	0	0	0	0	0	0	1	0	0	0	0
28/03/2022	M	2	0	0	0	0	0	0	0	0	0	0	1	0	0	0	0
05/04/2022	F	2	0	0	0	0	0	0	0	0	0	0	0	0	0	0	0
28/04/2022	M	2	0	0	0	0	0	0	0	0	0	0	1	0	0	0	0
23/08/2022	M	2	0	0	0	0	0	0	0	0	0	0	0	0	0	0	0
28/09/2022	M	2	0	0	0	0	0	0	0	0	0	0	1	0	0	0	1
06/10/2022	M	2	0	0	0	0	0	0	0	0	0	0	1	0	0	0	0
14/10/2022	M	2	0	0	0	0	0	0	0	0	0	0	1	0	0	0	0
02/11/2022	F	2	0	0	0	0	0	0	0	0	0	0	0	0	0	0	0
16/11/2022	F	2	0	0	0	0	0	0	0	0	0	0	0	0	0	0	0
07/12/2022	F	2	0	0	0	0	0	0	0	0	0	0	0	0	0	0	0
14/12/2022	M	2	0	0	0	0	0	0	0	0	0	0	0	0	0	0	1
09/01/2023	F	2	0	0	0	0	0	0	0	0	0	0	0	0	0	0	0
10/01/2023	F	2	0	0	0	0	0	0	0	0	0	0	0	0	0	0	1
03/02/2023	F	2	0	0	0	0	0	0	0	0	0	0	0	0	0	0	1
02/03/2023	M	2	0	0	0	0	0	0	0	0	0	0	1	0	0	0	0
09/03/2023	M	2	0	0	0	0	0	0	0	0	0	0	0	0	0	0	0
10/04/2023	M	2	0	0	0	0	0	0	0	0	0	0	1	0	0	0	0
24/04/2023	M	2	0	0	0	0	0	0	0	0	0	0	1	1	0	0	0
24/04/2023	F	2	0	0	0	0	0	0	0	0	0	0	1	1	0	0	0
27/04/2023	M	2	0	0	0	0	0	0	0	0	0	0	1	0	0	0	0
27/04/2023	F	2	0	0	0	0	0	0	0	0	0	0	0	0	0	0	0

19/05/2023	M	2	0	0	0	0	0	0	0	0	0	0	1	0	0	0	0
31/05/2023	M	2	0	0	0	0	0	0	0	0	0	0	1	0	0	0	1
13/06/2023	M	2	0	0	0	0	0	0	0	0	0	0	0	0	0	0	0
27/09/2023	M	2	0	0	0	0	0	0	0	0	0	0	1	0	0	0	0
21/11/2023	F	2	0	0	0	0	0	0	0	0	0	0	0	0	0	0	1
05/01/2024	F	2	0	0	0	0	0	0	0	0	0	0	0	0	0	0	1
11/01/2024	F	2	0	0	0	0	0	0	0	0	0	0	0	0	0	0	1
21/02/2024	M	2	0	0	0	0	0	0	0	0	0	0	0	0	0	0	1
04/03/2024	F	2	0	0	0	0	0	0	0	0	0	0	0	0	0	0	1
22/03/2024	F	2	0	0	0	0	0	0	0	0	0	0	0	0	0	0	1
29/03/2024	M	2	0	0	0	0	0	0	0	0	0	0	0	0	0	0	0
15/04/2024	M	2	0	0	0	0	0	0	0	0	0	0	0	1	0	0	0
22/04/2024	M	2	0	0	0	0	0	0	0	0	0	0	0	0	0	0	0
26/04/2024	M	2	0	0	0	0	0	0	0	0	0	0	0	0	0	0	1
21/05/2024	M	2	0	0	0	0	0	0	0	0	0	0	1	0	0	0	1
19/06/2024	M	2	0	0	0	0	0	0	0	0	0	0	0	0	0	0	0
26/06/2024	M	2	0	0	0	0	0	0	0	0	0	0	0	0	0	0	0
09/07/2024	M	2	0	0	0	0	0	0	0	0	0	0	0	0	0	0	0
30/08/2024	F	2	0	0	0	0	0	0	0	0	0	0	1	1	0	0	0
25/09/2024	F	2	0	0	0	0	0	0	0	0	0	0	0	0	0	0	0
09/10/2024	M	2	0	0	0	0	0	0	0	0	0	0	0	0	0	0	1
29/10/2024	M	2	0	0	0	0	0	0	0	0	0	0	0	0	0	0	1
08/04/2021	M	2	0	0	0	0	0	0	0	1	0	0	1	0	0	0	0
22/09/2021	F	2	0	0	0	0	0	0	0	0	0	1	0	0	0	0	0
03.11.2021	F	2	0	0	0	0	0	0	0	1	0	0	0	0	0	0	0
12.03.2021	F	2	0	0	0	0	0	0	0	1	0	0	0	0	1	0	1
20.10.2021	M	2	0	0	0	0	0	0	0	0	0	1	0	0	0	0	0
27.08.2021	F	2	0	0	0	0	0	0	0	1	0	0	0	0	0	1	1
20/03/2023	M	2	0	0	0	0	0	0	0	1	0	0	0	0	0	0	1

10/10/2023	F	2	0	0	0	0	0	0	0	1	0	0	0	0	0	0	0
16/05/2024	M	2	0	0	0	0	0	0	0	1	0	0	1	0	0	0	0
23/07/2024	M	2	0	0	0	0	0	0	0	1	0	0	0	0	0	0	1
08.12.2021	F	2	0	0	0	0	0	0	0	1	0	1	0	0	0	1	0
07/01/2020	M	2	0	0	0	0	1	0	0	0	0	0	1	0	0	0	1
07/09/2021	F	2	0	0	0	0	1	0	0	0	0	0	0	0	0	0	1
14/10/2020	F	2	0	0	0	0	1	0	0	1	0	0	1	0	1	0	1
08/09/2021	M	2	0	0	0	0	1	0	0	0	0	1	0	0	0	0	0
12.11.2021	F	2	0	0	1	0	0	0	0	0	0	1	0	0	0	0	0
30/08/2023	M	2	0	1	0	0	0	0	0	1	0	0	1	0	0	0	0
24/09/2024	M	2	0	0	0	0	1	0	0	1	0	0	1	0	0	0	0
28/10/2024	M	2	0	0	0	0	1	0	0	1	0	0	0	0	0	0	1
14/09/2020	M	2	0	0	1	0	0	1	0	0	0	0	1	1	0	0	0
29/11/2022	M	2	0	1	1	0	0	0	0	0	0	0	1	0	0	0	0
01.10.2021	M	2	1	1	1	0	0	0	0	0	0	1	0	0	0	0	0
10/11/2023	M	2	0	1	0	0	1	1	0	1	0	0	1	0	0	0	0
29/03/2021	F	2	0	0	0	0	0	0	0	0	0	0	0	0	0	0	0
31/01/2020	M	3	0	0	0	0	0	0	0	0	0	0	0	0	0	0	0
02/03/2020	M	3	0	0	0	0	0	0	0	0	0	0	0	0	0	0	0
16/03/2020	F	3	0	0	0	0	0	0	0	0	0	0	0	0	0	0	0
08/07/2020	M	3	0	0	0	0	0	0	0	0	0	0	0	0	0	0	0
29/07/2020	M	3	0	0	0	0	0	0	0	0	0	0	0	0	0	0	0
16/09/2020	M	3	0	0	0	0	0	0	0	0	0	0	1	1	0	0	0
24/09/2020	F	3	0	0	0	0	0	0	0	0	0	0	0	0	0	0	0
06/11/2020	M	3	0	0	0	0	0	0	0	0	0	0	0	0	0	0	1
25/11/2020	M	3	0	0	0	0	0	0	0	0	0	0	0	0	0	0	0
01/04/2021	M	3	0	0	0	0	0	0	0	0	0	0	1	0	0	0	0
24/05/2021	M	3	0	0	0	0	0	0	0	0	0	0	1	0	0	0	1
06/07/2021	M	3	0	0	0	0	0	0	0	0	0	0	1	0	0	0	0

26/08/2021	M	3	0	0	0	0	0	0	0	0	0	0	0	0	0	0	0
27/09/2021	M	3	0	0	0	0	0	0	0	0	0	0	0	0	0	0	1
28/09/2021	F	3	0	0	0	0	0	0	0	0	0	0	0	0	0	0	0
02/12/2021	F	3	0	0	0	0	0	0	0	0	0	0	1	0	0	0	1
20/12/2021	M	3	0	0	0	0	0	0	0	0	0	0	0	0	0	0	1
04.06.2021	F	3	0	0	0	0	0	0	0	0	0	0	0	0	0	1	0
07.05.2021	F	3	0	0	0	0	0	0	0	0	0	0	0	0	0	1	0
07/01/2022	M	3	0	0	0	0	0	0	0	0	0	0	0	0	0	0	0
18/01/2022	F	3	0	0	0	0	0	0	0	0	0	0	1	0	0	0	0
25/01/2022	F	3	0	0	0	0	0	0	0	0	0	0	1	0	0	0	0
02/02/2022	F	3	0	0	0	0	0	0	0	0	0	0	0	0	0	0	0
10/02/2022	F	3	0	0	0	0	0	0	0	0	0	0	1	0	0	0	0
16/02/2022	F	3	0	0	0	0	0	0	0	0	0	0	0	0	0	0	0
17/02/2022	M	3	0	0	0	0	0	0	0	0	0	0	1	0	0	0	0
25/02/2022	F	3	0	0	0	0	0	0	0	0	0	0	0	0	0	0	0
17/03/2022	F	3	0	0	0	0	0	0	0	0	0	0	0	0	0	0	1
30/03/2022	F	3	0	0	0	0	0	0	0	0	0	0	0	0	0	0	1
11/04/2022	F	3	0	0	0	0	0	0	0	0	0	0	1	0	0	0	0
13/04/2022	M	3	0	0	0	0	0	0	0	0	0	0	1	0	0	0	0
20/04/2022	F	3	0	0	0	0	0	0	0	0	0	0	0	0	0	0	1
29/04/2022	F	3	0	0	0	0	0	0	0	0	0	0	1	0	0	0	0
24/05/2022	F	3	0	0	0	0	0	0	0	0	0	0	0	0	0	0	0
30/05/2022	M	3	0	0	0	0	0	0	0	0	0	0	0	0	0	0	0
08/06/2022	F	3	0	0	0	0	0	0	0	0	0	0	1	1	0	0	0
17/06/2022	M	3	0	0	0	0	0	0	0	0	0	0	1	0	0	0	0
13/07/2022	F	3	0	0	0	0	0	0	0	0	0	0	0	0	0	0	0
21/07/2022	F	3	0	0	0	0	0	0	0	0	0	0	0	0	0	0	0
27/09/2022	M	3	0	0	0	0	0	0	0	0	0	0	0	0	0	0	1
28/09/2022	F	3	0	0	0	0	0	0	0	0	0	0	1	0	0	0	1

04/10/2022	F	3	0	0	0	0	0	0	0	0	0	0	1	0	1	0	0
04/10/2022	M	3	0	0	0	0	0	0	0	0	0	0	1	1	0	0	0
04/11/2022	M	3	0	0	0	0	0	0	0	0	0	0	0	0	0	0	0
16/11/2022	M	3	0	0	0	0	0	0	0	0	0	0	0	0	0	0	0
17/11/2022	F	3	0	0	0	0	0	0	0	0	0	0	0	0	0	0	0
06/12/2022	M	3	0	0	0	0	0	0	0	0	0	0	1	0	0	0	0
03/03/2023	M	3	0	0	0	0	0	0	0	0	0	0	1	0	0	0	1
02/05/2023	F	3	0	0	0	0	0	0	0	0	0	0	0	0	0	0	1
08/05/2023	M	3	0	0	0	0	0	0	0	0	0	0	0	0	0	0	0
08/05/2023	F	3	0	0	0	0	0	0	0	0	0	0	0	0	0	0	0
19/05/2023	F	3	0	0	0	0	0	0	0	0	0	0	1	0	0	0	0
26/05/2023	M	3	0	0	0	0	0	0	0	0	0	0	0	0	0	0	1
20/06/2023	F	3	0	0	0	0	0	0	0	0	0	0	0	0	0	0	0
26/07/2023	M	3	0	0	0	0	0	0	0	0	0	0	1	0	0	0	0
04/03/2024	F	3	0	0	0	0	0	0	0	0	0	0	0	0	0	0	0
13/03/2024	M	3	0	0	0	0	0	0	0	0	0	0	0	0	0	0	0
09/04/2024	F	3	0	0	0	0	0	0	0	0	0	0	1	0	0	0	0
21/08/2024	F	3	0	0	0	0	0	0	0	0	0	0	0	0	0	0	0
05/09/2024	M	3	0	0	0	0	0	0	0	0	0	0	1	1	0	0	0
08/10/2024	F	3	0	0	0	0	0	0	0	0	0	0	0	0	0	0	1
09/12/2024	F	3	0	0	0	0	0	0	0	0	0	0	0	0	0	0	0
22/04/2021	M	3	0	0	0	0	0	0	0	1	0	0	0	0	0	0	1
13/09/2021	M	3	0	0	0	0	0	0	0	1	0	0	1	1	0	0	0
21/09/2021	M	3	0	0	0	0	0	0	0	1	0	0	1	0	0	0	0
27/09/2021	F	3	0	0	0	0	0	0	0	1	0	0	0	0	0	0	1
26.03.2021	M	3	0	0	0	0	0	0	0	1	0	0	1	0	0	0	0
27.08.2021	M	3	0	0	0	0	0	0	0	0	0	1	1	0	0	0	0
21/09/2022	M	3	0	0	0	0	0	0	0	1	0	0	0	0	1	0	0
14/11/2022	M	3	0	0	0	0	0	0	0	1	0	0	0	0	0	0	1

07/04/2023	M	3	0	0	0	0	0	0	0	1	0	0	0	0	0	0	1
19/09/2024	M	3	0	0	0	0	0	0	0	0	1	0	0	0	0	0	0
31/08/2021	M	3	0	1	0	0	0	0	0	0	0	0	1	0	0	0	1
09/06/2022	M	3	0	0	0	0	1	0	0	0	0	0	1	1	0	0	0
12/07/2022	M	3	0	0	0	0	0	0	1	0	0	0	1	0	0	0	0
01/09/2022	M	3	0	0	0	0	1	0	0	0	0	0	1	0	0	0	0
15/12/2022	M	3	0	0	0	0	1	0	0	0	0	0	1	0	1	0	0
17/03/2023	F	3	0	0	0	0	1	0	0	0	0	0	1	0	0	0	0
29.09.2021	F	3	0	0	0	0	1	0	0	0	0	1	1	0	0	0	0
12/05/2023	M	3	0	0	0	0	0	0	1	1	0	0	1	0	0	0	0
06/11/2020	M	3	1	0	1	0	0	0	0	0	0	0	0	0	0	0	0
13/05/2022	M	3	0	1	0	0	0	0	1	0	0	0	1	0	0	0	0
14/12/2022	M	3	0	1	0	0	0	0	1	0	0	0	1	0	0	0	0
16/08/2023	F	3	1	0	0	1	0	0	0	0	0	0	1	0	0	0	0
06/11/2023	F	3	0	0	0	0	1	1	0	0	0	0	1	0	1	0	0
05/08/2021	F	3	1	1	0	0	0	0	0	1	0	0	1	1	0	0	0
07/04/2022	M	3	0	1	1	0	0	0	0	1	0	1	1	1	0	0	0
21/08/2023	F	3	0	0	1	1	1	0	0	0	0	0	1	1	0	0	0
31/10/2023	M	3	1	1	1	0	0	1	1	1	0	0	1	0	0	0	1
14/01/2020	M	4	0	0	0	0	0	0	0	0	0	0	0	0	0	0	1
11/02/2020	M	4	0	0	0	0	0	0	0	0	0	0	0	0	0	0	1
19/02/2020	F	4	0	0	0	0	0	0	0	0	0	0	0	0	0	0	1
26/02/2020	M	4	0	0	0	0	0	0	0	0	0	0	1	1	0	0	0
07/09/2020	F	4	0	0	0	0	0	0	0	0	0	0	1	0	1	0	0
14/09/2020	M	4	0	0	0	0	0	0	0	0	0	0	1	1	1	0	0
11/01/2021	M	4	0	0	0	0	0	0	0	0	0	0	0	0	0	0	1
10/05/2021	M	4	0	0	0	0	0	0	0	0	0	0	0	0	0	0	1
14/06/2021	M	4	0	0	0	0	0	0	0	0	0	0	1	0	0	0	0
23/08/2021	F	4	0	0	0	0	0	0	0	0	0	0	0	0	0	0	1

23/08/2021	F	4	0	0	0	0	0	0	0	0	0	0	0	0	0	0	0
03/09/2021	M	4	0	0	0	0	0	0	0	0	0	0	0	0	0	0	0
23/09/2021	M	4	0	0	0	0	0	0	0	0	0	0	1	0	0	0	0
12/10/2021	M	4	0	0	0	0	0	0	0	0	0	0	1	0	0	0	0
22.10.2021	F	4	0	0	0	0	0	0	0	0	0	0	0	0	0	0	0
27.08.2021	F	4	0	0	0	0	0	0	0	0	0	0	0	0	0	1	0
27.10.2021	F	4	0	0	0	0	0	0	0	0	0	0	0	0	0	1	0
06/01/2022	M	4	0	0	0	0	0	0	0	0	0	0	1	0	0	0	0
21/01/2022	F	4	0	0	0	0	0	0	0	0	0	0	0	0	0	0	0
28/01/2022	M	4	0	0	0	0	0	0	0	0	0	0	0	0	0	0	0
02/02/2022	M	4	0	0	0	0	0	0	0	0	0	0	0	0	0	0	0
09/02/2022	F	4	0	0	0	0	0	0	0	0	0	0	0	0	0	0	0
17/02/2022	M	4	0	0	0	0	0	0	0	0	0	0	0	0	0	0	0
25/02/2022	F	4	0	0	0	0	0	0	0	0	0	0	0	0	0	0	1
17/03/2022	F	4	0	0	0	0	0	0	0	0	0	0	1	0	0	0	0
21/03/2022	M	4	0	0	0	0	0	0	0	0	0	0	1	0	0	0	0
23/03/2022	M	4	0	0	0	0	0	0	0	0	0	0	0	0	0	0	0
07/04/2022	F	4	0	0	0	0	0	0	0	0	0	0	0	0	0	0	0
11/05/2022	M	4	0	0	0	0	0	0	0	0	0	0	0	0	0	0	0
24/05/2022	F	4	0	0	0	0	0	0	0	0	0	0	1	0	0	0	0
27/05/2022	M	4	0	0	0	0	0	0	0	0	0	0	0	0	0	0	1
21/06/2022	M	4	0	0	0	0	0	0	0	0	0	0	0	0	0	0	1
29/06/2022	M	4	0	0	0	0	0	0	0	0	0	0	0	0	1	0	0
26/07/2022	M	4	0	0	0	0	0	0	0	0	0	0	0	0	0	0	1
07/09/2022	M	4	0	0	0	0	0	0	0	0	0	0	0	0	0	0	0
28/09/2022	M	4	0	0	0	0	0	0	0	0	0	0	0	0	0	0	0
17/10/2022	F	4	0	0	0	0	0	0	0	0	0	0	1	0	0	0	0
04/11/2022	M	4	0	0	0	0	0	0	0	0	0	0	1	0	0	0	0
15/12/2022	F	4	0	0	0	0	0	0	0	0	0	0	0	0	0	0	1

06/01/2023	F	4	0	0	0	0	0	0	0	0	0	0	1	0	0	0	1
30/01/2023	M	4	0	0	0	0	0	0	0	0	0	0	1	0	0	0	0
22/02/2023	F	4	0	0	0	0	0	0	0	0	0	0	1	0	0	0	0
30/03/2023	F	4	0	0	0	0	0	0	0	0	0	0	0	0	0	0	0
03/04/2023	M	4	0	0	0	0	0	0	0	0	0	0	0	0	1	0	0
10/05/2023	F	4	0	0	0	0	0	0	0	0	0	0	1	0	0	0	0
11/05/2023	M	4	0	0	0	0	0	0	0	0	0	0	0	0	0	0	0
11/05/2023	M	4	0	0	0	0	0	0	0	0	0	0	0	0	0	0	0
19/05/2023	M	4	0	0	0	0	0	0	0	0	0	0	0	0	0	0	1
13/06/2023	F	4	0	0	0	0	0	0	0	0	0	0	0	0	0	0	0
27/07/2023	M	4	0	0	0	0	0	0	0	0	0	0	1	0	0	0	0
13/10/2023	F	4	0	0	0	0	0	0	0	0	0	0	0	0	0	0	1
08/11/2023	F	4	0	0	0	0	0	0	0	0	0	0	0	0	0	0	1
21/11/2023	M	4	0	0	0	0	0	0	0	0	0	0	0	0	0	0	0
27/11/2023	F	4	0	0	0	0	0	0	0	0	0	0	0	0	1	0	0
10/01/2024	M	4	0	0	0	0	0	0	0	0	0	0	0	0	0	0	1
19/01/2024	F	4	0	0	0	0	0	0	0	0	0	0	0	0	0	0	1
07/02/2024	F	4	0	0	0	0	0	0	0	0	0	0	0	0	0	0	1
07/02/2024	F	4	0	0	0	0	0	0	0	0	0	0	0	0	0	0	1
09/02/2024	F	4	0	0	0	0	0	0	0	0	0	0	0	0	0	0	1
13/03/2024	F	4	0	0	0	0	0	0	0	0	0	0	0	0	0	0	0
23/04/2024	F	4	0	0	0	0	0	0	0	0	0	0	0	0	0	0	0
24/04/2024	F	4	0	0	0	0	0	0	0	0	0	0	0	0	0	0	0
14/05/2024	F	4	0	0	0	0	0	0	0	0	0	0	1	0	0	0	0
01/07/2024	M	4	0	0	0	0	0	0	0	0	0	0	0	0	1	0	0
22/08/2024	M	4	0	0	0	0	0	0	0	0	0	0	1	0	0	0	0
09/10/2024	F	4	0	0	0	0	0	0	0	0	0	0	0	0	0	0	0
11/10/2024	F	4	0	0	0	0	0	0	0	0	0	0	1	0	1	0	0
22/01/2020	M	4	0	0	0	0	0	0	0	0	1	0	0	0	0	0	0

10/09/2021	M	4	0	0	0	0	0	0	0	0	0	1	1	0	0	0	0
20/12/2021	F	4	0	0	0	0	0	0	0	1	0	0	0	0	0	0	0
08/06/2022	M	4	0	0	0	0	0	0	0	1	0	0	0	0	0	0	1
17/05/2023	M	4	0	0	0	0	0	0	0	1	0	0	0	0	0	0	0
24/07/2023	F	4	0	0	0	0	0	0	0	0	0	1	0	0	0	0	1
01/11/2023	F	4	0	0	0	0	0	0	0	0	1	0	0	0	0	0	0
09/05/2024	M	4	0	0	0	0	0	0	0	0	0	1	0	0	0	0	0
24/09/2024	M	4	0	0	0	0	0	0	0	1	0	0	0	0	0	0	0
08.12.2021	M	4	0	0	0	0	0	0	0	1	0	1	0	0	0	0	0
07/01/2020	F	4	0	0	0	0	1	0	0	0	0	0	1	0	0	0	0
04/12/2020	F	4	0	0	0	0	1	0	0	0	0	0	1	0	0	0	1
13.01.2021	M	4	0	0	0	0	1	0	0	0	0	0	1	0	0	0	0
06/01/2023	M	4	0	0	1	0	0	0	0	0	0	0	1	0	0	0	0
26/06/2023	F	4	0	0	1	0	0	0	0	0	0	0	1	0	0	0	0
19/04/2022	M	4	0	0	0	0	0	1	0	0	0	0	1	0	0	0	0
11/05/2022	F	4	0	0	0	0	1	0	0	0	0	0	1	1	0	0	0
11/05/2022	M	4	0	0	0	0	1	0	0	0	0	0	1	0	0	0	0
05/10/2022	F	4	0	0	0	0	1	0	0	0	0	0	1	1	1	0	0
24/10/2022	M	4	0	0	0	0	1	0	0	0	0	0	1	1	0	0	0
31/10/2022	M	4	0	0	0	0	1	0	0	0	0	0	1	0	0	0	0
03/11/2022	F	4	0	1	0	0	0	0	0	0	0	0	1	1	0	0	0
04/11/2022	M	4	0	0	0	0	1	0	0	0	0	0	1	0	1	0	0
22/11/2022	M	4	0	0	0	0	1	0	0	0	0	0	1	0	0	0	0
14/12/2022	M	4	0	0	0	1	0	0	0	0	0	0	1	0	1	0	0
19/12/2022	M	4	0	0	0	0	1	0	0	0	0	0	1	0	0	0	0
22/03/2023	M	4	0	0	0	0	1	0	0	0	0	0	0	0	0	0	1
15/05/2023	M	4	0	1	0	0	0	0	0	0	0	0	1	1	0	0	0
23/08/2023	M	4	0	0	0	0	0	1	0	0	0	0	1	0	0	0	0
06/09/2023	M	4	0	0	0	0	1	0	0	0	0	0	1	0	1	0	0

15/11/2023	M	4	0	0	0	0	0	0	1	0	0	0	1	0	0	0	0
12/02/2024	M	4	0	1	0	0	0	0	0	0	0	0	1	1	0	0	1
18/06/2024	M	4	0	0	0	0	0	1	0	0	0	0	1	0	0	0	0
19/09/2024	F	4	0	0	0	0	1	0	0	0	0	0	1	1	1	0	0
10.09.2021	M	4	0	0	0	0	1	0	0	0	0	1	1	0	0	1	0
11/01/2023	M	4	1	0	0	0	0	0	0	1	0	0	1	0	0	0	1
12/02/2020	M	4	0	0	0	1	0	0	1	0	0	0	1	0	0	0	0
15/06/2020	M	4	0	1	0	0	0	0	1	0	0	0	1	0	0	0	0
15/07/2020	F	4	0	1	0	0	1	0	0	0	0	0	1	0	1	0	0
10.02.2021	F	4	1	0	0	0	1	0	0	0	0	0	1	1	0	0	0
03/11/2020	F	4	0	1	1	0	0	0	0	0	0	0	1	1	0	0	0
09/03/2021	M	4	0	0	1	0	1	0	0	0	0	0	1	1	0	0	0
09/11/2021	M	4	0	1	1	0	0	0	0	0	0	0	1	0	0	0	0
18/11/2021	F	4	0	0	1	0	0	0	1	0	0	0	1	0	1	0	0
21/03/2022	M	4	0	0	1	1	0	0	0	0	0	0	1	0	1	0	0
15/02/2023	F	4	0	0	1	0	1	0	0	0	0	0	1	0	0	0	1
11/02/2022	M	4	0	0	0	0	1	0	1	0	0	0	1	0	0	0	0
24/05/2022	M	4	0	1	0	0	1	0	0	0	0	0	1	1	0	0	0
23/04/2024	M	4	0	0	0	0	1	0	1	0	0	0	1	0	0	0	0
19/11/2024	M	4	0	0	0	0	1	0	1	0	0	0	1	1	1	0	0
26.11.2021	F	4	0	0	1	0	0	0	1	0	0	1	0	1	0	0	0
21/10/2021	M	4	1	0	1	0	0	1	0	0	0	0	1	1	0	0	0
01/10/2024	F	4	1	0	1	0	0	1	0	0	0	0	1	1	1	0	0
03/11/2022	M	4	0	1	0	0	1	1	0	0	0	0	1	0	0	0	0
26/04/2024	F	4	0	1	0	0	1	0	1	0	0	0	1	1	1	0	0
02/06/2020	F	4	1	1	1	0	0	0	0	1	0	0	1	1	0	0	0
07/03/2022	M	4	1	0	1	0	0	1	0	1	0	0	1	0	0	0	0
13/11/2024	M	4	0	1	1	0	0	0	1	1	0	0	1	1	0	0	0
14/10/2020	M	4	1	1	0	1	0	0	1	0	0	0	1	0	0	0	0

14/09/2020	F	4	0	0	1	0	1	1	1	0	0	0	1	0	1	1	0
01/10/2020	M	4	1	1	1	0	0	1	0	1	0	0	1	1	0	0	0
15.09.2021	M	4	1	1	1	0	0	0	1	0	0	1	1	0	0	0	0
27/01/2020	M	5	0	0	0	0	0	0	0	0	0	0	0	0	0	0	0
19/02/2020	F	5	0	0	0	0	0	0	0	0	0	0	0	0	0	0	0
25/02/2020	F	5	0	0	0	0	0	0	0	0	0	0	0	0	0	0	1
09/06/2020	F	5	0	0	0	0	0	0	0	0	0	0	1	0	1	0	0
10/08/2020	F	5	0	0	0	0	0	0	0	0	0	0	0	0	0	0	1
11/08/2020	F	5	0	0	0	0	0	0	0	0	0	0	1	0	0	0	1
21/09/2020	F	5	0	0	0	0	0	0	0	0	0	0	1	0	0	0	0
29/09/2020	M	5	0	0	0	0	0	0	0	0	0	0	1	0	0	0	0
12/10/2020	F	5	0	0	0	0	0	0	0	0	0	0	0	0	1	0	0
08/02/2021	M	5	0	0	0	0	0	0	0	0	0	0	0	0	0	0	0
29/04/2021	F	5	0	0	0	0	0	0	0	0	0	0	0	0	0	0	1
28/06/2021	F	5	0	0	0	0	0	0	0	0	0	0	0	0	0	0	1
06/09/2021	M	5	0	0	0	0	0	0	0	0	0	0	0	0	0	0	0
21/09/2021	F	5	0	0	0	0	0	0	0	0	0	0	1	0	0	0	0
11/11/2021	F	5	0	0	0	0	0	0	0	0	0	0	0	0	0	0	1
29/11/2021	F	5	0	0	0	0	0	0	0	0	0	0	0	0	1	0	0
02/12/2021	F	5	0	0	0	0	0	0	0	0	0	0	0	0	0	0	1
09.06.2021	F	5	0	0	0	0	0	0	0	0	0	0	0	0	0	1	0
12.03.2021	F	5	0	0	0	0	0	0	0	0	0	0	0	0	1	0	1
12.05.2021	F	5	0	0	0	0	0	0	0	0	0	0	0	0	0	1	0
19.05.2021	F	5	0	0	0	0	0	0	0	0	0	0	0	0	0	1	0
24.02.2021	F	5	0	0	0	0	0	0	0	0	0	0	0	0	1	0	1
21/01/2022	M	5	0	0	0	0	0	0	0	0	0	0	0	0	0	0	0
28/01/2022	M	5	0	0	0	0	0	0	0	0	0	0	0	0	0	0	0
17/03/2022	M	5	0	0	0	0	0	0	0	0	0	0	1	0	0	0	0
22/03/2022	F	5	0	0	0	0	0	0	0	0	0	0	0	0	0	0	1

24/03/2022	F	5	0	0	0	0	0	0	0	0	0	0	1	0	0	0	0
04/04/2022	M	5	0	0	0	0	0	0	0	0	0	0	1	0	0	0	0
10/05/2022	M	5	0	0	0	0	0	0	0	0	0	0	0	0	0	0	0
25/05/2022	M	5	0	0	0	0	0	0	0	0	0	0	0	0	0	0	0
02/08/2022	M	5	0	0	0	0	0	0	0	0	0	0	1	0	0	0	1
22/08/2022	F	5	0	0	0	0	0	0	0	0	0	0	0	0	1	0	0
30/08/2022	F	5	0	0	0	0	0	0	0	0	0	0	0	0	0	0	1
16/09/2022	M	5	0	0	0	0	0	0	0	0	0	0	1	0	0	0	0
20/09/2022	M	5	0	0	0	0	0	0	0	0	0	0	0	0	1	0	0
27/09/2022	F	5	0	0	0	0	0	0	0	0	0	0	1	0	1	0	1
14/10/2022	M	5	0	0	0	0	0	0	0	0	0	0	1	0	0	0	0
04/11/2022	F	5	0	0	0	0	0	0	0	0	0	0	1	0	0	0	0
07/11/2022	M	5	0	0	0	0	0	0	0	0	0	0	0	0	0	0	0
14/11/2022	F	5	0	0	0	0	0	0	0	0	0	0	1	0	0	0	0
14/11/2022	F	5	0	0	0	0	0	0	0	0	0	0	0	0	1	0	0
17/11/2022	F	5	0	0	0	0	0	0	0	0	0	0	0	0	0	0	0
22/11/2022	F	5	0	0	0	0	0	0	0	0	0	0	1	0	1	0	1
06/01/2023	M	5	0	0	0	0	0	0	0	0	0	0	1	0	0	0	0
27/01/2023	F	5	0	0	0	0	0	0	0	0	0	0	0	0	1	0	0
15/02/2023	M	5	0	0	0	0	0	0	0	0	0	0	1	0	0	0	0
20/03/2023	F	5	0	0	0	0	0	0	0	0	0	0	1	0	1	0	0
28/03/2023	F	5	0	0	0	0	0	0	0	0	0	0	1	0	0	0	0
18/09/2023	F	5	0	0	0	0	0	0	0	0	0	0	0	0	0	0	1
09/10/2023	M	5	0	0	0	0	0	0	0	0	0	0	0	0	0	0	0
30/10/2023	F	5	0	0	0	0	0	0	0	0	0	0	0	0	0	0	1
16/11/2023	M	5	0	0	0	0	0	0	0	0	0	0	1	0	0	0	0
27/11/2023	F	5	0	0	0	0	0	0	0	0	0	0	0	0	1	0	0
22/01/2024	F	5	0	0	0	0	0	0	0	0	0	0	0	0	1	0	0
26/01/2024	M	5	0	0	0	0	0	0	0	0	0	0	1	0	0	0	0

05/02/2024	M	5	0	0	0	0	0	0	0	0	0	0	1	1	1	0	1
23/02/2024	F	5	0	0	0	0	0	0	0	0	0	0	0	0	1	0	0
27/02/2024	M	5	0	0	0	0	0	0	0	0	0	0	0	0	1	0	0
01/03/2024	F	5	0	0	0	0	0	0	0	0	0	0	1	0	0	0	0
01/03/2024	F	5	0	0	0	0	0	0	0	0	0	0	1	0	1	0	0
12/04/2024	F	5	0	0	0	0	0	0	0	0	0	0	0	0	0	0	0
25/06/2024	F	5	0	0	0	0	0	0	0	0	0	0	0	0	0	0	1
03/07/2024	F	5	0	0	0	0	0	0	0	0	0	0	0	0	0	0	1
09/07/2024	F	5	0	0	0	0	0	0	0	0	0	0	0	0	0	0	0
23/07/2024	M	5	0	0	0	0	0	0	0	0	0	0	0	0	0	0	1
27/08/2024	F	5	0	0	0	0	0	0	0	0	0	0	0	0	0	0	0
16/10/2024	F	5	0	0	0	0	0	0	0	0	0	0	1	0	0	0	0
20/01/2020	F	5	0	0	0	0	0	0	0	1	0	0	0	0	0	0	1
07/10/2020	M	5	0	0	0	0	0	0	0	1	0	0	1	0	0	0	0
15.10.2021	M	5	0	0	0	0	0	0	0	0	0	1	0	0	0	0	0
15.12.2021	M	5	0	0	0	0	0	0	0	0	0	1	0	1	0	1	0
26.11.2021	M	5	0	0	0	0	0	0	0	1	0	0	0	0	0	0	0
17/01/2022	F	5	0	0	0	0	0	0	0	0	1	0	1	0	1	0	0
27/09/2022	M	5	0	0	0	0	0	0	0	1	0	0	0	0	0	0	1
29/03/2023	M	5	0	0	0	0	0	0	0	1	0	0	0	0	0	0	1
28/06/2023	F	5	0	0	0	0	0	0	0	0	1	0	0	0	0	0	1
26/04/2024	M	5	0	0	0	0	0	0	0	1	0	0	0	0	0	0	0
29/10/2024	M	5	0	0	0	0	0	0	0	1	0	0	0	0	0	0	1
03/02/2020	M	5	0	1	0	0	0	0	0	0	0	0	1	0	0	0	0
15/09/2020	M	5	0	0	0	0	1	0	0	0	0	0	1	0	1	0	0
29/09/2020	M	5	0	0	0	0	0	0	1	0	0	0	1	0	0	0	0
26/01/2021	F	5	0	0	0	0	1	0	0	0	0	0	1	1	0	0	1
15/06/2021	M	5	0	0	0	0	1	0	0	0	0	0	0	0	1	0	0
18/10/2021	M	5	0	0	0	0	1	0	0	0	0	0	1	0	1	0	0

17/03/2022	M	5	0	1	0	0	0	0	0	0	0	0	1	0	1	0	0
19/04/2022	M	5	0	0	0	0	0	1	0	0	0	0	1	0	0	0	0
10/05/2022	M	5	0	0	0	0	1	0	0	0	0	0	1	0	1	0	0
08/06/2022	M	5	0	0	0	0	1	0	0	0	0	0	1	0	0	0	0
28/08/2022	M	5	0	0	0	0	1	0	0	0	0	0	1	0	1	0	0
08/11/2022	M	5	1	0	0	0	0	0	0	0	0	0	0	0	1	0	0
26/06/2023	F	5	0	0	0	0	1	0	0	0	0	0	1	0	1	0	0
20.10.2021	F	5	0	0	0	0	1	0	0	0	0	1	1	0	0	0	0
25/05/2022	F	5	0	0	0	0	1	0	0	1	0	0	1	0	0	0	0
24/04/2024	M	5	0	0	0	0	1	0	0	0	1	0	1	1	0	0	0
30/07/2024	M	5	0	0	0	0	1	0	0	0	1	0	1	1	0	0	0
30/07/2024	M	5	1	0	0	0	0	0	0	1	0	0	1	0	1	0	1
08/10/2024	F	5	0	0	0	0	0	1	0	1	0	0	1	0	0	0	0
09/06/2020	M	5	1	1	0	0	0	0	0	0	0	0	1	1	0	0	0
17/07/2020	M	5	0	0	0	0	1	0	1	0	0	0	1	0	0	0	0
20/07/2020	M	5	0	0	0	0	1	1	0	0	0	0	1	0	0	0	0
11/11/2021	F	5	0	0	1	0	1	0	0	0	0	0	1	0	0	0	0
27/06/2022	M	5	0	1	1	0	0	0	0	0	0	0	1	1	0	0	0
04/04/2022	M	5	0	1	0	0	0	0	1	0	0	0	1	0	1	0	0
18/07/2022	M	5	0	1	0	0	1	0	0	0	0	0	1	1	0	0	0
10/05/2023	F	5	0	0	0	0	1	0	1	0	0	0	1	0	0	0	0
26/06/2023	M	5	1	1	0	0	0	0	0	0	0	0	1	1	0	0	0
03/11/2023	M	5	0	0	0	0	1	1	0	0	0	0	1	0	1	0	1
08/04/2021	M	5	0	0	0	1	1	0	0	1	0	0	1	0	1	0	1
26.11.2021	F	5	0	0	1	0	0	0	1	0	0	1	0	0	0	1	0
26/10/2020	M	5	0	0	0	0	1	1	1	0	0	0	1	0	0	0	0
09/09/2021	M	5	0	1	0	1	0	1	0	0	0	0	1	1	1	0	0
23/12/2020	M	5	0	1	1	0	1	0	0	0	0	0	1	0	0	0	1
07/09/2021	F	5	0	0	1	1	0	1	0	0	0	0	1	1	0	0	0

21/12/2022	M	5	1	0	1	0	0	1	0	0	0	0	1	1	1	0	0
06/06/2023	F	5	1	1	1	0	0	0	0	0	0	0	1	1	0	0	0
24/07/2023	M	5	0	1	1	0	0	0	1	0	0	0	1	0	1	0	0
01/11/2023	F	5	0	1	1	0	0	1	0	0	0	0	1	0	1	0	0
20/06/2022	M	5	1	1	0	0	1	0	0	0	0	0	1	0	1	0	0
27/07/2020	M	5	1	1	0	0	1	0	0	1	0	0	1	1	0	0	0
09/08/2023	M	5	0	1	0	1	1	0	0	1	0	0	1	0	0	0	1
08.12.2021	M	5	0	0	1	1	1	0	0	1	0	1	0	0	0	1	0
04/04/2022	M	5	1	1	0	0	0	0	1	1	1	0	1	1	0	0	0
21/09/2020	M	5	1	1	0	1	0	0	1	0	0	0	1	1	1	0	0
09/11/2020	F	5	0	1	1	0	0	1	1	0	0	0	1	1	0	0	0
27/10/2022	F	5	0	1	1	0	1	1	0	0	0	0	1	0	1	0	0
20/11/2020	M	5	1	1	0	1	1	1	1	0	0	0	1	0	0	0	0
22/02/2020	M	6	0	0	0	0	0	0	0	0	0	0	0	0	1	0	0
11/03/2020	M	6	0	0	0	0	0	0	0	0	0	0	0	0	0	0	0
13/03/2020	F	6	0	0	0	0	0	0	0	0	0	0	0	0	0	0	1
06/07/2020	M	6	0	0	0	0	0	0	0	0	0	0	1	0	1	0	0
20/07/2020	M	6	0	0	0	0	0	0	0	0	0	0	0	0	0	0	0
10/09/2020	M	6	0	0	0	0	0	0	0	0	0	0	0	0	1	0	0
24/09/2020	F	6	0	0	0	0	0	0	0	0	0	0	1	0	1	0	0
18/01/2021	M	6	0	0	0	0	0	0	0	0	0	0	0	0	0	0	0
29/06/2021	M	6	0	0	0	0	0	0	0	0	0	0	1	0	1	0	0
22/07/2021	F	6	0	0	0	0	0	0	0	0	0	0	0	0	1	0	1
03/08/2021	M	6	0	0	0	0	0	0	0	0	0	0	0	0	0	0	0
31/08/2021	M	6	0	0	0	0	0	0	0	0	0	0	0	0	0	0	0
05/10/2021	M	6	0	0	0	0	0	0	0	0	0	0	0	0	0	0	0
26/10/2021	F	6	0	0	0	0	0	0	0	0	0	0	0	0	0	0	0
06/12/2021	M	6	0	0	0	0	0	0	0	0	0	0	0	0	0	0	0
07/12/2021	M	6	0	0	0	0	0	0	0	0	0	0	0	0	0	0	0

08.12.2021	F	6	0	0	0	0	0	0	0	0	0	0	0	1	0	0	0
20.10.2021	M	6	0	0	0	0	0	0	0	0	0	0	0	0	0	0	1
27.10.2021	M	6	0	0	0	0	0	0	0	0	0	0	0	1	0	0	0
28.05.2021	M	6	0	0	0	0	0	0	0	0	0	0	0	0	0	1	0
23/02/2022	M	6	0	0	0	0	0	0	0	0	0	0	0	0	0	0	0
28/03/2022	F	6	0	0	0	0	0	0	0	0	0	0	1	0	0	0	0
13/04/2022	F	6	0	0	0	0	0	0	0	0	0	0	1	0	0	0	0
19/04/2022	F	6	0	0	0	0	0	0	0	0	0	0	1	0	0	0	0
24/05/2022	M	6	0	0	0	0	0	0	0	0	0	0	0	0	1	0	0
22/08/2022	M	6	0	0	0	0	0	0	0	0	0	0	1	0	1	0	0
07/09/2022	F	6	0	0	0	0	0	0	0	0	0	0	1	1	0	0	0
09/09/2022	M	6	0	0	0	0	0	0	0	0	0	0	0	0	0	0	1
20/02/2023	F	6	0	0	0	0	0	0	0	0	0	0	0	0	0	0	0
15/03/2023	F	6	0	0	0	0	0	0	0	0	0	0	1	0	0	0	1
07/04/2023	M	6	0	0	0	0	0	0	0	0	0	0	1	0	0	0	0
29/05/2023	F	6	0	0	0	0	0	0	0	0	0	0	1	0	0	0	0
06/06/2023	M	6	0	0	0	0	0	0	0	0	0	0	1	1	0	0	0
16/06/2023	F	6	0	0	0	0	0	0	0	0	0	0	0	0	0	0	1
16/08/2023	M	6	0	0	0	0	0	0	0	0	0	0	0	0	0	0	1
18/08/2023	F	6	0	0	0	0	0	0	0	0	0	0	0	0	0	0	1
25/08/2023	M	6	0	0	0	0	0	0	0	0	0	0	1	0	0	0	0
10/11/2023	F	6	0	0	0	0	0	0	0	0	0	0	1	0	0	0	0
15/12/2023	M	6	0	0	0	0	0	0	0	0	0	0	1	0	0	0	0
25/03/2024	M	6	0	0	0	0	0	0	0	0	0	0	0	0	0	1	0
02/04/2024	F	6	0	0	0	0	0	0	0	0	0	0	0	0	0	0	0
12/04/2024	M	6	0	0	0	0	0	0	0	0	0	0	0	0	0	0	0
08/05/2024	M	6	0	0	0	0	0	0	0	0	0	0	0	0	0	0	1
28/05/2024	F	6	0	0	0	0	0	0	0	0	0	0	0	0	0	0	0
03/07/2024	M	6	0	0	0	0	0	0	0	0	0	0	0	0	0	0	1

23/07/2024	M	6	0	0	0	0	0	0	0	0	0	0	0	0	0	0	1
02/08/2024	F	6	0	0	0	0	0	0	0	0	0	0	0	0	0	0	1
30/08/2024	F	6	0	0	0	0	0	0	0	0	0	0	0	0	0	0	0
04/09/2024	M	6	0	0	0	0	0	0	0	0	0	0	0	0	0	0	1
27/09/2024	M	6	0	0	0	0	0	0	0	0	0	0	0	1	0	0	0
07/10/2024	M	6	0	0	0	0	0	0	0	0	0	0	0	0	0	0	0
06/11/2024	M	6	0	0	0	0	0	0	0	0	0	0	0	0	1	0	0
22/01/2020	M	6	0	0	0	0	0	0	0	1	0	0	0	0	0	0	0
07/09/2020	M	6	0	0	0	0	0	0	0	0	0	1	1	0	0	0	0
20.10.2021	F	6	0	0	0	0	0	0	0	0	0	1	0	0	0	0	0
11/04/2022	M	6	0	0	0	0	0	0	0	1	0	0	0	0	1	0	0
20/09/2023	M	6	0	0	0	0	0	0	0	0	0	1	0	0	0	0	0
24/10/2023	M	6	0	0	0	0	0	0	0	0	1	0	0	0	0	0	0
08/01/2020	M	6	0	0	0	0	1	0	0	0	0	0	1	0	1	0	1
22/01/2020	M	6	0	0	0	0	1	0	0	0	0	0	1	0	1	0	0
02/06/2020	M	6	0	0	0	0	1	0	0	0	0	0	1	0	1	0	0
26/06/2020	F	6	0	0	0	0	1	0	0	0	0	0	1	0	1	0	0
29/03/2021	M	6	0	0	0	0	1	0	0	0	0	0	1	0	0	0	0
11/05/2021	M	6	0	1	0	0	0	0	0	0	0	0	1	1	0	0	0
07/06/2021	M	6	0	1	0	0	0	0	0	0	0	0	1	1	0	0	0
20/09/2021	M	6	0	0	0	0	1	0	0	0	0	0	1	0	1	0	0
22/10/2020	M	6	0	0	1	0	0	0	0	0	0	0	1	0	1	0	0
22/11/2022	F	6	0	0	1	0	0	0	0	0	0	0	1	0	1	0	0
11/04/2022	F	6	0	0	0	0	1	0	0	0	0	0	1	1	0	0	0
10/06/2022	M	6	0	0	0	0	1	0	0	0	0	0	1	0	1	0	0
10/04/2023	F	6	0	0	0	0	1	0	0	0	0	0	1	0	1	0	0
07/06/2023	M	6	0	0	0	0	1	0	0	0	0	0	1	0	1	0	1
22/06/2023	M	6	0	0	0	0	1	0	0	0	0	0	1	0	1	0	0
29/06/2023	M	6	0	0	0	0	1	0	0	0	0	0	1	1	0	0	0

16/10/2023	M	6	0	1	0	0	0	0	0	0	0	0	1	0	1	0	0
15/11/2023	F	6	0	0	0	0	0	0	1	0	0	0	1	0	1	0	1
29/03/2024	M	6	0	1	0	0	0	0	0	0	0	0	1	0	1	0	1
09/04/2024	M	6	0	0	0	0	1	0	0	0	0	0	1	1	0	0	0
01/08/2024	M	6	0	0	0	0	0	0	1	0	0	0	1	0	1	0	0
25/10/2024	F	6	0	0	0	0	1	0	0	0	0	0	1	1	1	0	0
29/04/2021	F	6	1	0	0	0	0	0	0	1	0	0	1	1	0	0	1
12.05.2021	M	6	0	0	0	0	1	0	0	0	0	1	0	0	0	0	0
13/01/2023	F	6	0	0	0	0	1	0	0	1	1	0	1	0	1	0	1
14/01/2020	F	6	0	0	0	0	1	1	0	0	0	0	1	0	1	0	0
21/01/2020	F	6	0	0	0	0	1	1	0	0	0	0	1	0	1	0	0
31/01/2020	M	6	0	1	0	0	0	0	1	0	0	0	1	0	0	0	1
08/09/2020	M	6	0	1	0	0	0	1	0	0	0	0	1	1	0	0	0
24.02.2021	M	6	1	1	0	0	0	0	0	0	0	0	1	1	1	0	0
16/05/2022	M	6	0	0	1	0	1	0	0	0	0	0	1	0	1	0	0
13/12/2022	F	6	0	0	1	0	0	1	0	0	0	0	1	0	1	1	0
11/10/2024	M	6	0	1	1	0	0	0	0	0	0	0	1	0	1	0	0
20/04/2022	M	6	0	1	0	0	1	0	0	0	0	0	1	0	0	0	0
05/07/2022	M	6	0	0	0	0	1	1	0	0	0	0	0	0	1	1	1
21/09/2022	F	6	0	1	0	0	0	1	0	0	0	0	1	0	1	1	0
24/05/2023	F	6	0	0	0	0	1	0	1	0	0	0	1	0	1	0	0
26/07/2023	M	6	0	0	0	0	1	1	0	0	0	0	1	0	1	0	0
24/11/2023	M	6	1	0	0	0	1	0	0	0	0	0	1	0	0	0	0
06/03/2024	F	6	1	1	0	0	0	0	0	0	0	0	1	1	0	0	0
10/04/2024	M	6	0	1	0	0	1	0	0	0	0	0	1	1	1	0	0
20/08/2024	F	6	0	1	0	0	0	1	0	0	0	0	1	1	0	0	1
19.05.2021	M	6	0	1	0	0	1	0	0	0	0	1	0	0	0	0	0
03.11.2021	M	6	0	0	1	0	1	0	0	1	0	1	0	1	0	0	0
30/09/2020	M	6	1	1	0	1	0	0	0	0	0	0	1	1	0	0	0

24.02.2021	M	6	0	1	0	0	1	0	1	0	0	0	1	1	0	0	0
17/08/2020	M	6	0	0	1	1	0	1	0	0	0	0	1	1	0	0	0
19/09/2022	F	6	1	0	1	1	0	0	0	0	0	0	1	0	1	0	0
20/12/2024	F	6	0	0	1	0	0	1	1	0	0	0	1	0	0	0	0
25/05/2023	F	6	0	1	0	0	1	1	0	0	0	0	1	0	1	0	1
15/11/2023	M	6	0	1	0	0	1	0	1	0	0	0	1	0	1	0	0
19/01/2024	M	6	0	1	0	0	1	1	0	1	0	0	1	0	0	0	0
27/01/2020	M	7	0	0	0	0	0	0	0	0	0	0	0	0	1	0	0
28/01/2020	M	7	0	0	0	0	0	0	0	0	0	0	0	0	0	0	1
13/03/2020	M	7	0	0	0	0	0	0	0	0	0	0	0	0	0	0	1
28/05/2020	F	7	0	0	0	0	0	0	0	0	0	0	1	0	0	0	0
04/08/2020	M	7	0	0	0	0	0	0	0	0	0	0	1	0	0	0	0
20/05/2021	M	7	0	0	0	0	0	0	0	0	0	0	1	1	0	0	0
13/10/2021	M	7	0	0	0	0	0	0	0	0	0	0	0	1	0	1	0
12.02.2021	F	7	0	0	0	0	0	0	0	0	0	0	0	0	0	0	0
13/01/2022	F	7	0	0	0	0	0	0	0	0	0	0	0	0	0	0	0
09/02/2022	M	7	0	0	0	0	0	0	0	0	0	0	0	0	0	0	0
07/04/2022	M	7	0	0	0	0	0	0	0	0	0	0	1	1	0	0	0
13/04/2022	M	7	0	0	0	0	0	0	0	0	0	0	1	0	0	0	0
02/05/2022	F	7	0	0	0	0	0	0	0	0	0	0	0	0	0	0	0
27/05/2022	F	7	0	0	0	0	0	0	0	0	0	0	1	0	0	0	0
22/06/2022	M	7	0	0	0	0	0	0	0	0	0	0	0	1	0	0	0
07/09/2022	F	7	0	0	0	0	0	0	0	0	0	0	1	0	0	0	0
11/10/2022	F	7	0	0	0	0	0	0	0	0	0	0	0	0	0	0	0
27/10/2022	F	7	0	0	0	0	0	0	0	0	0	0	0	0	0	0	0
08/11/2022	M	7	0	0	0	0	0	0	0	0	0	0	0	0	0	0	0
10/02/2023	F	7	0	0	0	0	0	0	0	0	0	0	0	0	0	0	1
21/03/2023	F	7	0	0	0	0	0	0	0	0	0	0	1	0	1	0	0
22/05/2023	F	7	0	0	0	0	0	0	0	0	0	0	0	0	0	0	1

28/07/2023	F	7	0	0	0	0	0	0	0	0	0	0	1	0	0	0	0
12/10/2023	M	7	0	0	0	0	0	0	0	0	0	0	0	0	0	0	0
29/01/2024	F	7	0	0	0	0	0	0	0	0	0	0	0	0	0	0	0
07/02/2024	M	7	0	0	0	0	0	0	0	0	0	0	1	0	1	0	0
02/04/2024	M	7	0	0	0	0	0	0	0	0	0	0	0	0	0	0	1
07/05/2024	F	7	0	0	0	0	0	0	0	0	0	0	0	0	1	0	0
22/05/2024	F	7	0	0	0	0	0	0	0	0	0	0	0	0	0	0	1
02/08/2024	M	7	0	0	0	0	0	0	0	0	0	0	0	0	0	0	0
20/08/2024	M	7	0	0	0	0	0	0	0	0	0	0	0	0	0	0	1
18/09/2024	M	7	0	0	0	0	0	0	0	0	0	0	0	0	0	0	0
09/10/2024	F	7	0	0	0	0	0	0	0	0	0	0	0	0	0	0	0
12/12/2024	F	7	0	0	0	0	0	0	0	0	0	0	0	0	1	0	0
29/07/2021	M	7	0	0	0	0	0	0	0	0	1	0	0	0	1	0	1
15.10.2021	F	7	0	0	0	0	0	0	0	0	0	1	0	0	0	0	0
02/08/2024	M	7	0	0	0	0	0	0	0	0	0	1	0	0	0	0	0
16/01/2020	M	7	0	0	0	0	1	0	0	0	0	0	1	0	1	0	0
22/01/2020	F	7	0	0	0	0	1	0	0	0	0	0	1	0	0	0	1
18/02/2020	M	7	0	0	0	0	1	0	0	0	0	0	1	0	1	0	0
09/07/2020	M	7	0	0	0	0	1	0	0	0	0	0	1	0	1	0	0
24/07/2020	M	7	0	0	0	0	1	0	0	0	0	0	1	0	1	0	0
09/09/2020	F	7	0	0	0	0	0	1	0	0	0	0	1	0	1	0	0
05/11/2020	F	7	0	0	0	0	1	0	0	0	0	0	1	0	1	0	0
07/06/2021	M	7	0	1	0	0	0	0	0	0	0	0	1	1	0	0	0
08/06/2021	F	7	1	0	0	0	0	0	0	0	0	0	1	0	0	0	0
23/11/2021	F	7	0	0	0	0	1	0	0	0	0	0	1	0	1	0	0
23/10/2020	M	7	0	0	1	0	0	0	0	0	0	0	0	0	0	0	0
15/11/2021	M	7	0	0	1	0	0	0	0	0	0	0	1	0	1	0	0
12/10/2022	M	7	0	0	1	0	0	0	0	0	0	0	1	1	0	0	0
19/09/2023	M	7	0	0	1	0	0	0	0	0	0	0	1	0	0	0	1

23/02/2024	M	7	0	0	1	0	0	0	0	0	0	0	1	1	0	0	1
08/02/2022	F	7	0	0	0	0	1	0	0	0	0	0	1	1	0	0	0
20/09/2022	M	7	0	0	0	0	1	0	0	0	0	0	1	0	1	0	0
08/02/2023	F	7	0	0	0	0	1	0	0	0	0	0	1	0	0	0	0
16/05/2023	M	7	0	1	0	0	0	0	0	0	0	0	1	1	0	0	0
16/06/2023	M	7	0	1	0	0	0	0	0	0	0	0	1	0	0	0	0
18/09/2023	M	7	0	0	0	0	1	0	0	0	0	0	1	0	0	0	0
20/09/2023	M	7	0	0	0	0	1	0	0	0	0	0	1	0	1	0	0
29/03/2024	M	7	0	0	0	0	1	0	0	0	0	0	1	0	1	0	0
27/06/2024	M	7	0	0	0	0	1	0	0	0	0	0	1	0	1	0	0
05/07/2024	M	7	0	1	0	0	0	0	0	0	0	0	1	0	1	0	0
06/11/2024	F	7	0	1	0	0	0	0	0	0	0	0	0	0	1	0	0
03/09/2021	M	7	0	0	0	0	1	0	0	0	0	1	0	0	0	0	0
05.11.2021	F	7	0	0	0	0	1	0	0	0	0	1	0	0	0	1	0
15/04/2022	F	7	0	0	0	0	1	0	0	0	1	0	1	0	0	0	0
12/12/2023	M	7	0	0	0	0	1	0	0	1	0	0	1	0	0	0	0
28.04.2021	M	7	0	0	0	0	0	0	1	1	0	1	0	1	0	1	0
05/10/2022	M	7	0	1	1	0	0	0	0	0	0	0	1	1	0	0	0
08/02/2024	F	7	1	0	1	0	0	0	0	0	0	0	1	0	0	0	1
08/03/2024	M	7	0	0	1	0	0	0	1	0	0	0	1	1	0	0	1
31/05/2022	M	7	1	1	0	0	0	0	0	0	0	0	1	1	0	0	0
05/10/2022	F	7	0	1	0	1	0	0	0	0	0	0	1	0	0	0	1
26/05/2023	M	7	0	1	0	0	1	0	0	0	0	0	1	0	0	0	1
07/10/2024	M	7	0	1	0	0	0	1	0	0	0	0	1	1	1	0	0
14/09/2021	M	7	1	1	0	1	0	0	0	0	0	0	1	0	1	0	0
30/01/2020	F	7	0	1	1	0	0	1	0	0	0	0	1	0	1	0	0
31/07/2020	F	7	0	1	1	1	0	0	0	0	0	0	1	1	0	0	0
08/09/2020	F	7	1	1	1	0	0	0	0	0	0	0	0	0	0	0	0
05/10/2022	M	7	0	1	1	1	0	0	0	0	0	0	1	1	0	0	0

27/10/2022	M	7	0	1	1	0	1	0	0	0	0	0	1	0	1	0	0
06/02/2023	M	7	1	1	1	0	0	0	0	0	0	0	0	0	0	0	1
07/01/2022	M	7	0	1	0	0	1	1	0	0	0	0	1	0	0	0	0
06/03/2023	M	7	0	0	0	0	1	1	1	0	0	0	1	0	0	0	0
09/02/2024	F	7	0	1	0	0	1	1	0	0	0	0	1	0	0	0	1
04/10/2022	M	7	0	0	1	0	0	1	1	1	0	0	1	0	0	0	1
22/10/2020	M	7	0	1	1	0	1	1	0	0	0	0	1	1	1	0	0
20/09/2021	F	7	0	1	1	0	0	1	1	0	0	0	1	0	0	0	0
27/07/2023	F	7	1	1	1	0	0	1	0	0	0	0	1	0	0	0	0
09/08/2023	M	7	1	1	0	1	0	1	0	0	0	0	1	0	1	0	1
13/11/2024	M	7	1	1	0	1	0	1	0	0	0	0	1	0	1	0	0
25/11/2022	M	7	0	1	1	0	1	1	0	1	0	1	1	0	0	0	0
18/10/2021	M	7	1	1	1	1	0	1	1	0	0	0	1	1	0	0	0
08/01/2020	M	8	0	0	0	0	0	0	0	0	0	0	0	0	0	0	1
09/01/2020	M	8	0	0	0	0	0	0	0	0	0	0	1	0	1	0	0
09/03/2020	M	8	0	0	0	0	0	0	0	0	0	0	0	0	0	0	0
22/06/2020	M	8	0	0	0	0	0	0	0	0	0	0	1	0	1	0	1
17/07/2020	M	8	0	0	0	0	0	0	0	0	0	0	1	0	0	0	0
27/07/2020	M	8	0	0	0	0	0	0	0	0	0	0	1	0	0	0	0
05/08/2020	M	8	0	0	0	0	0	0	0	0	0	0	0	0	0	0	1
03/09/2020	F	8	0	0	0	0	0	0	0	0	0	0	1	1	0	0	0
14/09/2020	F	8	0	0	0	0	0	0	0	0	0	0	1	0	1	0	0
30/09/2020	F	8	0	0	0	0	0	0	0	0	0	0	0	0	0	0	0
07/12/2020	F	8	0	0	0	0	0	0	0	0	0	0	0	0	0	0	1
11/12/2020	M	8	0	0	0	0	0	0	0	0	0	0	1	0	0	0	0
25/01/2021	M	8	0	0	0	0	0	0	0	0	0	0	0	0	0	0	0
07/06/2021	F	8	0	0	0	0	0	0	0	0	0	0	0	0	0	0	1
15/06/2021	F	8	0	0	0	0	0	0	0	0	0	0	0	0	0	0	0
06/07/2021	F	8	0	0	0	0	0	0	0	0	0	0	0	0	0	0	0

11/11/2021	F	8	0	0	0	0	0	0	0	0	0	0	0	0	0	0	0
29/04/2022	M	8	0	0	0	0	0	0	0	0	0	0	0	0	0	0	1
30/05/2022	M	8	0	0	0	0	0	0	0	0	0	0	1	1	0	0	0
05/07/2022	F	8	0	0	0	0	0	0	0	0	0	0	1	0	0	0	0
05/07/2022	F	8	0	0	0	0	0	0	0	0	0	0	0	0	0	0	1
31/08/2022	F	8	0	0	0	0	0	0	0	0	0	0	1	1	1	1	0
07/09/2022	F	8	0	0	0	0	0	0	0	0	0	0	1	1	0	0	0
19/09/2022	F	8	0	0	0	0	0	0	0	0	0	0	1	0	1	0	0
07/12/2022	M	8	0	0	0	0	0	0	0	0	0	0	0	0	0	0	1
07/12/2022	M	8	0	0	0	0	0	0	0	0	0	0	0	0	0	0	0
03/02/2023	F	8	0	0	0	0	0	0	0	0	0	0	0	0	0	0	1
03/04/2023	M	8	0	0	0	0	0	0	0	0	0	0	0	0	0	0	1
03/10/2023	M	8	0	0	0	0	0	0	0	0	0	0	0	0	0	0	1
10/11/2023	F	8	0	0	0	0	0	0	0	0	0	0	0	0	1	0	0
24/11/2023	M	8	0	0	0	0	0	0	0	0	0	0	0	0	1	0	0
04/01/2024	M	8	0	0	0	0	0	0	0	0	0	0	0	0	0	0	1
15/01/2024	F	8	0	0	0	0	0	0	0	0	0	0	0	0	0	0	0
14/02/2024	F	8	0	0	0	0	0	0	0	0	0	0	1	1	0	0	1
04/03/2024	F	8	0	0	0	0	0	0	0	0	0	0	0	0	0	0	1
25/06/2024	F	8	0	0	0	0	0	0	0	0	0	0	0	0	0	0	0
17/07/2024	F	8	0	0	0	0	0	0	0	0	0	0	0	0	0	0	0
16/10/2024	F	8	0	0	0	0	0	0	0	0	0	0	0	0	0	0	1
13/11/2024	M	8	0	0	0	0	0	0	0	0	0	0	0	0	1	0	0
26/11/2024	M	8	0	0	0	0	0	0	0	0	0	0	0	0	1	0	1
19/05/2020	F	8	0	0	0	0	0	0	0	0	1	0	1	0	1	0	0
01/10/2021	M	8	0	0	0	0	0	0	0	0	0	1	0	0	0	0	0
26.05.2021	F	8	0	0	0	0	0	0	0	0	0	1	0	0	0	0	0
29.10.2021	M	8	0	0	0	0	0	0	0	0	0	1	0	1	0	1	0
02/03/2023	M	8	0	0	0	0	0	0	0	0	1	0	0	0	0	0	0

27/02/2020	M	8	0	0	0	0	1	0	0	0	0	0	0	0	1	0	0
11/08/2020	M	8	0	1	0	0	0	0	0	0	0	0	1	1	0	0	0
09/12/2020	M	8	1	0	0	0	0	0	0	0	0	0	1	0	0	0	0
23/08/2021	M	8	0	0	0	0	1	0	0	0	0	0	1	0	1	0	1
13/12/2021	F	8	0	0	1	0	0	0	0	0	0	0	1	1	0	0	0
22/06/2022	M	8	0	0	1	0	0	0	0	0	0	0	1	1	0	0	0
13/07/2022	M	8	0	0	1	0	0	0	0	0	0	0	1	1	0	0	0
06/09/2023	M	8	0	0	1	0	0	0	0	0	0	0	1	0	1	1	0
21/01/2022	M	8	0	1	0	0	0	0	0	0	0	0	1	1	1	0	1
31/08/2022	M	8	0	0	0	0	1	0	0	0	0	0	1	1	1	0	0
09/09/2022	F	8	0	1	0	0	0	0	0	0	0	0	1	0	1	0	0
29/03/2023	M	8	0	0	0	0	0	1	0	0	0	0	1	0	1	0	0
07/08/2023	M	8	0	0	0	0	1	0	0	0	0	0	1	0	1	0	0
29/09/2023	M	8	0	0	0	0	0	1	0	0	0	0	1	0	1	0	0
18/10/2023	M	8	0	0	0	0	1	0	0	0	0	0	1	0	0	0	0
27/10/2023	F	8	0	1	0	0	0	0	0	0	0	0	1	0	0	0	0
19/03/2024	M	8	0	0	0	0	1	0	0	0	0	0	1	0	0	0	1
04/09/2024	M	8	0	1	0	0	0	0	0	0	0	0	1	0	1	0	0
29/11/2024	F	8	0	0	0	0	1	0	0	0	0	0	1	0	1	0	0
10/01/2024	M	8	0	0	0	0	0	0	1	1	0	0	1	0	1	0	1
05.11.2021	M	8	0	0	1	0	0	0	0	1	0	1	1	1	0	0	0
13/01/2020	F	8	0	0	1	0	0	0	1	0	0	0	1	0	0	0	0
30/08/2022	M	8	0	0	1	0	0	0	1	0	0	0	1	1	0	0	0
26/11/2024	M	8	0	0	1	0	1	0	0	0	0	0	1	1	0	0	0
08/11/2023	F	8	0	0	0	0	1	1	0	0	0	0	1	0	1	1	0
30/04/2024	F	8	0	1	0	0	0	1	0	0	0	0	1	0	1	0	1
03/09/2024	F	8	0	1	0	0	1	0	0	0	0	0	1	0	0	0	0
05/09/2024	M	8	0	1	0	1	0	0	0	0	0	0	0	0	1	0	0
20.10.2021	M	8	0	1	1	0	0	0	0	0	0	1	1	1	0	0	0

17/06/2022	M	8	1	0	0	0	0	0	1	1	0	0	1	1	0	0	0
22/07/2020	F	8	0	1	0	0	1	0	1	0	0	0	1	1	0	0	0
15/01/2020	F	8	0	1	1	0	1	0	0	0	0	0	1	0	0	0	0
08/07/2020	M	8	1	1	1	0	0	0	0	0	0	0	1	1	0	0	0
03/10/2022	M	8	0	1	1	1	0	0	0	0	0	0	1	1	0	0	0
12/10/2022	M	8	1	0	1	1	0	0	0	0	0	0	1	1	0	0	1
21/11/2022	F	8	0	1	1	0	0	0	1	0	0	0	1	0	0	0	0
09/08/2023	M	8	0	1	1	0	1	0	0	0	0	0	1	0	0	0	1
13/01/2022	F	8	0	1	0	0	1	1	0	0	0	0	1	0	1	0	0
29/11/2024	F	8	1	1	0	0	0	1	0	0	0	0	1	0	0	0	0
29/09/2021	F	8	1	0	0	1	0	1	0	0	0	1	1	0	0	0	0
26.05.2021	M	8	1	1	0	1	0	0	0	0	0	1	1	0	0	0	0
30/08/2021	M	8	0	1	1	1	0	0	1	0	0	0	1	1	0	0	0
04/09/2024	M	8	0	1	1	0	0	1	1	0	0	0	1	1	0	0	0
06/08/2020	M	8	1	1	1	0	1	0	1	0	0	0	1	1	0	0	0
25/07/2023	M	8	1	1	0	0	1	1	1	0	0	0	1	0	0	0	1
08/09/2021	M	8	1	1	1	1	0	1	0	0	0	1	1	1	0	0	0
21/12/2022	F	8	1	1	1	1	0	0	1	1	0	0	1	0	0	0	0
21/11/2023	F	8	1	1	0	0	1	1	1	1	0	0	1	1	1	0	0
10/02/2020	F	9	0	0	0	0	0	0	0	0	0	0	0	0	0	0	1
24/02/2020	M	9	0	0	0	0	0	0	0	0	0	0	0	0	0	0	1
04/03/2020	M	9	0	0	0	0	0	0	0	0	0	0	1	0	0	0	0
21/09/2020	M	9	0	0	0	0	0	0	0	0	0	0	0	0	1	0	0
25/09/2020	F	9	0	0	0	0	0	0	0	0	0	0	0	0	1	0	0
13/10/2020	F	9	0	0	0	0	0	0	0	0	0	0	1	0	0	0	1
18/11/2020	F	9	0	0	0	0	0	0	0	0	0	0	0	0	0	0	0
11/01/2021	M	9	0	0	0	0	0	0	0	0	0	0	0	0	0	0	0
04/02/2021	F	9	0	0	0	0	0	0	0	0	0	0	1	0	0	0	0
21/09/2021	F	9	0	0	0	0	0	0	0	0	0	0	1	0	0	0	0

13.10.2021	M	9	0	0	0	0	0	0	0	0	0	0	0	1	0	0	0
13/01/2022	F	9	0	0	0	0	0	0	0	0	0	0	0	0	0	0	0
14/03/2022	M	9	0	0	0	0	0	0	0	0	0	0	1	1	1	0	0
11/05/2022	M	9	0	0	0	0	0	0	0	0	0	0	1	0	1	0	0
25/05/2022	F	9	0	0	0	0	0	0	0	0	0	0	0	0	0	0	0
13/07/2022	F	9	0	0	0	0	0	0	0	0	0	0	0	0	0	0	0
02/08/2022	M	9	0	0	0	0	0	0	0	0	0	0	1	0	1	0	0
21/09/2022	M	9	0	0	0	0	0	0	0	0	0	0	1	1	0	0	0
06/12/2022	M	9	0	0	0	0	0	0	0	0	0	0	1	0	1	0	0
26/01/2023	F	9	0	0	0	0	0	0	0	0	0	0	0	0	1	0	0
28/04/2023	F	9	0	0	0	0	0	0	0	0	0	0	0	0	0	0	1
15/05/2023	F	9	0	0	0	0	0	0	0	0	0	0	1	0	0	0	0
26/06/2023	F	9	0	0	0	0	0	0	0	0	0	0	0	0	0	0	0
12/10/2023	M	9	0	0	0	0	0	0	0	0	0	0	1	0	1	0	0
27/10/2023	M	9	0	0	0	0	0	0	0	0	0	0	1	0	0	0	0
05/01/2024	M	9	0	0	0	0	0	0	0	0	0	0	1	0	0	0	1
01/03/2024	M	9	0	0	0	0	0	0	0	0	0	0	0	0	0	0	0
26/03/2024	F	9	0	0	0	0	0	0	0	0	0	0	0	0	0	0	0
15/04/2024	F	9	0	0	0	0	0	0	0	0	0	0	0	0	0	0	1
07/05/2024	M	9	0	0	0	0	0	0	0	0	0	0	0	0	0	0	1
08/05/2024	M	9	0	0	0	0	0	0	0	0	0	0	0	0	0	0	0
16/05/2024	M	9	0	0	0	0	0	0	0	0	0	0	1	1	0	0	0
23/08/2024	F	9	0	0	0	0	0	0	0	0	0	0	0	0	0	0	0
04/10/2021	F	9	0	0	0	0	0	0	0	0	1	0	0	0	0	0	0
13/05/2022	F	9	0	0	0	0	0	0	0	0	1	0	1	0	0	0	1
24/05/2023	F	9	0	0	0	0	0	0	0	1	0	0	1	0	0	0	0
31/07/2023	M	9	0	0	0	0	0	0	0	1	0	0	0	0	0	0	1
29/07/2020	F	9	0	0	0	0	1	0	0	0	0	0	1	0	0	0	0
26/01/2021	M	9	0	0	0	0	1	0	0	0	0	0	1	0	0	0	0

18/06/2020	M	9	0	0	1	0	0	0	0	0	0	0	1	1	1	0	0
08/04/2021	F	9	0	0	1	0	0	0	0	0	0	0	1	1	0	0	1
13/01/2022	M	9	0	0	0	0	1	0	0	0	0	0	1	0	0	0	0
19/04/2023	M	9	0	1	0	0	0	0	0	0	0	0	1	0	0	0	0
09/08/2023	F	9	0	0	0	0	0	0	1	0	0	0	1	0	1	0	0
20/09/2023	M	9	0	1	0	0	0	0	0	0	0	0	1	0	0	0	0
06/10/2023	M	9	0	0	0	0	1	0	0	0	0	0	1	0	0	0	0
09/04/2024	F	9	0	0	0	0	1	0	0	0	0	0	1	0	1	1	0
15/04/2024	M	9	0	1	0	0	0	0	0	0	0	0	1	0	0	0	0
25/06/2024	M	9	0	0	0	0	1	0	0	0	0	0	1	0	1	0	0
02/10/2024	F	9	0	0	0	0	1	0	0	0	0	0	1	0	1	0	0
25/10/2024	M	9	0	0	0	0	1	0	0	0	0	0	1	1	0	0	0
13/10/2021	F	9	0	0	0	0	1	0	0	0	0	1	1	0	0	0	0
17/01/2020	M	9	0	1	0	0	1	0	0	0	0	0	1	1	1	0	1
29/07/2020	F	9	0	1	0	0	1	0	0	0	0	0	1	1	0	0	0
02/03/2021	M	9	0	0	0	0	1	1	0	0	0	0	1	0	1	1	0
29.01.2021	F	9	0	0	0	0	1	1	0	0	0	0	1	1	0	0	0
06/07/2020	M	9	0	1	1	0	0	0	0	0	0	0	1	1	0	0	0
12/08/2020	F	9	1	0	1	0	0	0	0	0	0	0	0	1	0	0	0
22/06/2022	F	9	0	1	1	0	0	0	0	0	0	0	1	1	1	0	0
17/10/2022	F	9	0	0	1	0	1	0	0	0	0	0	1	0	1	0	0
02/05/2023	M	9	0	1	1	0	0	0	0	0	0	0	1	1	0	0	0
07/09/2023	F	9	0	0	1	0	1	0	0	0	0	0	1	1	0	0	0
04/01/2023	M	9	0	1	0	0	1	0	0	0	0	0	1	0	1	0	0
28/07/2023	F	9	0	0	0	0	1	1	0	0	0	0	1	0	1	0	0
20/06/2024	F	9	0	0	0	0	0	1	1	0	0	0	1	0	1	1	0
17/09/2024	F	9	0	0	0	0	0	1	1	0	0	0	1	0	1	0	0
05/06/2020	F	9	1	1	1	0	0	0	0	0	0	0	1	1	0	0	0
31/05/2022	F	9	0	1	0	0	1	0	1	0	0	0	1	1	0	0	0

09/06/2022	F	9	0	1	0	0	1	0	1	0	0	0	1	1	0	0	0
29.10.2021	M	9	0	1	0	0	1	1	0	0	0	1	0	1	1	0	0
15/09/2020	F	9	1	1	1	0	0	1	0	0	0	0	1	1	0	0	0
20/03/2024	M	9	1	1	1	1	0	0	0	0	0	0	1	1	0	0	0
26/08/2021	M	9	1	1	0	0	1	1	1	0	0	0	1	1	0	0	0
21/01/2020	F	10	0	0	0	0	0	0	0	0	0	0	0	0	0	0	1
28/02/2020	M	10	0	0	0	0	0	0	0	0	0	0	0	0	0	0	0
16/06/2020	F	10	0	0	0	0	0	0	0	0	0	0	1	0	0	0	0
19/06/2020	F	10	0	0	0	0	0	0	0	0	0	0	1	1	0	0	0
23/09/2020	M	10	0	0	0	0	0	0	0	0	0	0	1	0	0	0	0
13/10/2020	M	10	0	0	0	0	0	0	0	0	0	0	1	0	1	0	0
08/12/2020	F	10	0	0	0	0	0	0	0	0	0	0	1	0	1	0	0
18/01/2021	F	10	0	0	0	0	0	0	0	0	0	0	0	0	0	0	1
08/06/2021	F	10	0	0	0	0	0	0	0	0	0	0	1	1	0	0	0
13/09/2021	M	10	0	0	0	0	0	0	0	0	0	0	1	1	0	0	0
24.02.2021	F	10	0	0	0	0	0	0	0	0	0	0	0	0	1	0	1
20/01/2022	M	10	0	0	0	0	0	0	0	0	0	0	0	0	0	0	0
19/04/2022	F	10	0	0	0	0	0	0	0	0	0	0	0	0	0	0	1
20/05/2022	M	10	0	0	0	0	0	0	0	0	0	0	0	0	0	0	1
23/08/2022	F	10	0	0	0	0	0	0	0	0	0	0	0	0	0	0	1
25/08/2022	M	10	0	0	0	0	0	0	0	0	0	0	1	0	0	0	1
28/09/2022	F	10	0	0	0	0	0	0	0	0	0	0	0	0	0	0	1
30/09/2022	M	10	0	0	0	0	0	0	0	0	0	0	1	0	0	0	0
24/10/2022	F	10	0	0	0	0	0	0	0	0	0	0	0	0	1	0	0
15/11/2022	M	10	0	0	0	0	0	0	0	0	0	0	0	0	1	0	0
17/01/2023	F	10	0	0	0	0	0	0	0	0	0	0	0	0	0	0	0
24/04/2023	F	10	0	0	0	0	0	0	0	0	0	0	1	0	0	0	0
02/08/2023	M	10	0	0	0	0	0	0	0	0	0	0	1	0	1	0	1
01/02/2024	M	10	0	0	0	0	0	0	0	0	0	0	0	0	0	0	1

07/02/2024	F	10	0	0	0	0	0	0	0	0	0	0	0	0	0	0	1
19/03/2024	M	10	0	0	0	0	0	0	0	0	0	0	1	0	0	0	0
28/08/2024	M	10	0	0	0	0	0	0	0	0	0	0	0	0	0	0	0
22/06/2020	F	10	0	0	0	0	0	0	0	1	0	0	1	0	1	0	0
02/07/2020	M	10	0	0	0	0	0	0	0	1	0	0	0	0	0	0	1
25/11/2022	F	10	0	0	0	0	0	0	0	0	1	0	0	0	0	0	0
13/01/2020	F	10	0	0	0	0	1	0	0	0	0	0	1	0	1	0	0
14/01/2020	M	10	0	0	0	0	1	0	0	0	0	0	1	0	0	0	0
22/04/2021	M	10	0	0	0	0	1	0	0	0	0	0	1	0	1	0	0
20/12/2021	F	10	0	0	0	0	1	0	0	0	0	0	1	0	1	0	0
23/08/2023	F	10	0	0	1	0	0	0	0	0	0	0	1	0	0	0	0
23/09/2024	M	10	0	0	1	0	0	0	0	0	0	0	1	1	0	0	0
01/03/2022	M	10	0	0	0	0	1	0	0	0	0	0	0	0	0	0	1
06/09/2022	F	10	1	0	0	0	0	0	0	0	0	0	1	0	0	0	1
06/11/2023	M	10	0	0	0	0	1	0	0	0	0	0	0	0	1	0	0
18/04/2024	M	10	0	0	0	0	1	0	0	0	0	0	0	0	1	0	0
26/04/2024	M	10	0	0	0	0	1	0	0	0	0	0	1	0	0	0	0
19/07/2024	M	10	0	0	0	0	1	0	0	0	0	0	1	0	0	0	0
30/10/2024	M	10	0	0	0	0	1	0	0	0	0	0	1	0	0	0	0
05/11/2024	F	10	0	0	0	0	1	0	0	0	0	0	1	0	0	0	0
29/11/2024	F	10	0	0	0	0	1	0	0	0	0	0	1	0	1	0	0
12/12/2024	M	10	0	1	0	0	0	0	0	0	0	0	1	0	0	0	0
15.12.2021	M	10	0	0	0	0	1	0	0	0	0	1	0	1	0	0	0
20/07/2020	M	10	0	1	0	0	0	0	1	0	0	0	1	0	1	0	0
07/06/2021	M	10	1	1	0	0	0	0	0	0	0	0	1	1	0	0	1
16/11/2022	M	10	0	0	1	0	0	0	1	0	0	0	1	0	1	0	0
30/04/2024	M	10	0	0	1	0	0	1	0	0	0	0	1	1	1	0	0
22/10/2024	F	10	0	0	1	0	1	0	0	0	0	0	1	1	0	0	0
03.12.2021	F	10	0	0	1	0	0	1	0	0	0	0	1	0	0	1	0

15/03/2023	M	10	0	0	0	0	1	0	1	0	0	0	1	0	1	0	0
22/09/2021	F	10	0	1	0	0	1	0	0	0	0	1	0	1	0	1	1
21.12.2021	M	10	0	0	0	0	1	1	0	0	0	1	0	1	0	0	0
25/08/2022	M	10	0	0	0	0	1	1	0	1	0	0	1	0	1	0	1
09/02/2024	F	10	0	1	0	0	1	0	0	1	0	0	1	0	1	0	0
27/05/2021	F	10	0	1	0	0	1	0	1	0	0	0	1	1	0	0	0
29.01.2021	M	10	0	1	0	0	1	1	0	0	0	0	1	0	1	0	0
14/09/2020	M	10	1	1	1	0	0	0	0	0	0	0	1	1	0	0	0
03/11/2020	F	10	0	1	1	1	0	0	0	0	0	0	1	1	0	0	0
27/09/2021	M	10	0	1	1	0	0	0	1	0	0	0	1	1	1	0	0
24/05/2022	M	10	1	1	1	0	0	0	0	0	0	0	1	1	0	0	0
09/09/2022	M	10	1	1	1	0	0	0	0	0	0	0	1	0	0	0	0
12/01/2023	M	10	0	1	1	0	1	0	0	0	0	0	1	0	0	0	0
16/08/2023	F	10	0	1	1	0	0	0	1	0	0	0	1	0	1	0	0
05/09/2023	M	10	0	0	1	0	1	1	0	0	0	0	1	1	0	0	0
19/02/2024	F	10	0	1	1	0	1	0	0	0	0	0	1	1	0	0	1
18/09/2024	M	10	0	1	1	0	1	0	0	0	0	0	1	1	0	0	1
16/10/2024	F	10	0	0	1	0	1	1	0	0	0	0	1	0	0	0	1
03/04/2023	F	10	0	0	0	1	1	1	0	0	0	0	1	0	1	0	0
24.11.2021	M	10	1	1	0	0	1	0	0	0	0	1	0	0	0	0	0
26/05/2020	M	10	1	1	1	0	0	0	1	0	0	0	1	1	0	0	0
21/06/2023	M	10	1	1	1	0	1	0	0	0	0	0	1	0	0	0	0
10/11/2023	M	10	1	1	1	0	1	0	0	0	0	0	1	1	0	0	0
28/07/2023	F	10	0	1	0	0	1	1	1	0	0	0	1	0	0	0	0
12/10/2020	F	10	1	0	1	0	1	1	1	0	0	0	1	0	1	0	0
27/09/2021	M	10	0	1	1	1	1	1	0	0	0	0	1	1	0	0	0
01/11/2023	M	10	1	1	1	0	1	1	0	0	0	0	1	0	1	0	0
02/06/2020	M	10	1	1	1	1	1	1	0	0	0	0	1	1	0	0	0
22/11/2021	F	10	1	1	1	1	1	0	1	0	0	0	1	1	0	0	1

24/07/2020	F	11	0	0	0	0	0	0	0	0	0	0	0	0	0	0	1
08/09/2020	F	11	0	0	0	0	0	0	0	0	0	0	1	0	1	0	0
09/09/2020	M	11	0	0	0	0	0	0	0	0	0	0	1	0	1	0	0
11/03/2021	M	11	0	0	0	0	0	0	0	0	0	0	0	0	0	0	0
06/09/2021	M	11	0	0	0	0	0	0	0	0	0	0	0	0	0	0	0
14/09/2021	M	11	0	0	0	0	0	0	0	0	0	0	0	0	0	0	1
18/11/2021	F	11	0	0	0	0	0	0	0	0	0	0	0	0	0	0	1
19/01/2022	F	11	0	0	0	0	0	0	0	0	0	0	0	0	0	0	1
20/01/2022	M	11	0	0	0	0	0	0	0	0	0	0	0	0	0	0	0
29/04/2022	F	11	0	0	0	0	0	0	0	0	0	0	1	0	1	0	0
07/09/2022	M	11	0	0	0	0	0	0	0	0	0	0	0	0	0	0	0
12/10/2022	M	11	0	0	0	0	0	0	0	0	0	0	1	0	1	0	0
02/11/2022	F	11	0	0	0	0	0	0	0	0	0	0	1	0	0	0	0
29/11/2022	F	11	0	0	0	0	0	0	0	0	0	0	0	0	0	0	1
19/12/2022	F	11	0	0	0	0	0	0	0	0	0	0	0	0	0	0	1
02/05/2023	M	11	0	0	0	0	0	0	0	0	0	0	1	0	1	0	0
11/05/2023	M	11	0	0	0	0	0	0	0	0	0	0	1	0	1	0	0
03/06/2023	F	11	0	0	0	0	0	0	0	0	0	0	0	0	0	0	0
14/06/2023	M	11	0	0	0	0	0	0	0	0	0	0	1	0	0	0	0
22/06/2023	F	11	0	0	0	0	0	0	0	0	0	0	1	0	1	0	0
16/11/2023	F	11	0	0	0	0	0	0	0	0	0	0	1	0	1	0	0
27/02/2024	F	11	0	0	0	0	0	0	0	0	0	0	1	0	1	0	0
20/08/2024	F	11	0	0	0	0	0	0	0	0	0	0	0	0	0	0	1
06/11/2024	F	11	0	0	0	0	0	0	0	0	0	0	0	0	1	0	0
10.11.2021	M	11	0	0	0	0	0	0	0	1	0	0	0	0	0	0	0
22.09.2021	F	11	0	0	0	0	0	0	0	1	0	0	0	1	0	1	0
05/01/2022	M	11	0	0	0	0	0	0	0	1	0	0	1	1	0	0	0
12/05/2022	M	11	0	0	0	0	0	0	0	1	0	0	0	0	0	0	1
19/09/2022	M	11	0	0	0	0	0	0	0	1	0	0	0	1	0	0	1

02/07/2020	F	11	0	1	0	0	0	0	0	0	0	0	1	1	0	0	0
02/09/2020	M	11	0	0	0	0	0	1	0	0	0	0	1	0	0	0	0
08/09/2020	M	11	0	0	0	0	0	1	0	0	0	0	1	1	0	0	0
09/09/2020	M	11	0	0	0	0	1	0	0	0	0	0	1	0	1	0	0
23/11/2020	F	11	0	0	0	0	1	0	0	0	0	0	1	1	1	0	0
15/06/2021	F	11	0	1	0	0	0	0	0	0	0	0	0	0	1	0	0
12.03.2021	M	11	0	0	0	0	1	0	0	0	0	0	1	1	0	0	0
30/10/2020	M	11	0	0	1	0	0	0	0	0	0	0	1	0	0	0	0
09/08/2023	M	11	0	0	1	0	0	0	0	0	0	0	1	0	1	0	0
27.01.2021	M	11	0	0	1	0	0	0	0	0	0	0	1	1	0	0	1
15/04/2022	M	11	0	0	0	0	1	0	0	0	0	0	1	0	0	0	0
20/05/2022	M	11	0	0	0	0	1	0	0	0	0	0	1	0	0	0	0
24/05/2022	M	11	0	1	0	0	0	0	0	0	0	0	1	1	0	0	0
01/08/2022	M	11	0	0	0	0	1	0	0	0	0	0	1	0	0	0	0
02/11/2022	M	11	0	0	0	0	1	0	0	0	0	0	1	0	1	0	0
24/11/2023	M	11	0	0	0	0	1	0	0	0	0	0	0	0	1	1	0
24/04/2024	M	11	0	0	0	0	0	0	1	0	0	0	1	0	1	0	0
21/08/2024	M	11	0	0	0	0	1	0	0	0	0	0	1	0	1	0	0
03/09/2021	M	11	0	1	0	0	0	0	0	0	0	1	1	0	0	0	0
10.09.2021	F	11	0	0	1	0	0	0	0	0	0	1	1	0	0	0	0
11/02/2020	M	11	0	0	0	0	1	0	1	0	0	0	1	0	0	0	0
24/02/2020	F	11	0	0	0	0	1	0	1	0	0	0	1	1	1	0	0
10/07/2020	M	11	0	0	0	0	1	1	0	0	0	0	1	1	1	0	0
24/07/2020	M	11	0	1	0	0	0	0	1	0	0	0	1	1	0	0	0
22/09/2020	F	11	0	0	0	0	1	1	0	0	0	0	1	1	1	0	0
30/09/2020	M	11	0	0	0	0	1	1	0	0	0	0	1	0	1	0	0
07/06/2021	M	11	0	1	0	0	1	0	0	0	0	0	1	0	0	0	0
05.05.2021	F	11	0	0	0	0	1	1	0	0	0	0	0	0	0	1	0
16/07/2020	M	11	0	1	1	0	0	0	0	0	0	0	1	1	1	0	0

02/05/2022	F	11	0	0	1	0	0	1	0	0	0	0	1	1	0	0	0
20/07/2022	M	11	0	1	1	0	0	0	0	0	0	0	1	1	0	0	1
21/12/2022	M	11	0	0	0	0	1	1	0	0	0	0	1	0	1	0	0
30/07/2024	M	11	0	0	0	0	1	1	0	0	0	0	1	0	0	0	0
30/08/2024	F	11	0	1	0	0	0	0	1	0	0	0	1	0	0	0	0
07/10/2020	M	11	0	1	1	0	0	0	0	0	0	1	1	1	0	0	0
02/09/2024	M	11	0	1	1	0	0	0	0	0	0	1	0	0	0	0	2
15/04/2021	M	11	0	0	0	0	1	1	1	0	0	0	1	0	0	0	0
03/09/2020	F	11	0	1	1	0	1	0	0	0	0	0	1	0	1	0	0
21/03/2022	M	11	0	1	1	1	0	0	0	0	0	0	1	1	0	0	0
20/05/2022	M	11	0	0	1	0	1	1	0	0	0	0	1	1	0	0	1
03/11/2022	M	11	1	1	0	0	0	1	0	0	0	0	1	0	0	0	0
28/05/2024	M	11	1	1	0	0	1	0	0	0	0	0	1	0	0	0	0
05.11.2021	M	11	0	0	1	0	1	0	1	0	0	1	0	0	0	0	0
02/06/2020	M	11	0	1	1	1	0	0	1	0	0	0	1	1	1	0	0
22/11/2021	F	11	0	1	1	1	1	0	0	0	0	0	1	1	0	0	1
02/08/2024	F	11	1	0	1	0	0	1	1	0	0	0	1	0	0	0	0
27/10/2023	F	11	1	1	0	0	1	1	0	0	0	0	1	0	1	0	1
06/10/2023	M	11	0	1	1	0	0	1	1	1	0	0	1	0	0	0	0
06/09/2024	M	11	1	1	0	1	0	0	1	1	0	0	1	0	0	0	0
11/02/2020	F	12	0	0	0	0	0	0	0	0	0	0	0	0	0	0	0
11/01/2021	M	12	0	0	0	0	0	0	0	0	0	0	0	0	0	0	1
07/06/2021	F	12	0	0	0	0	0	0	0	0	0	0	0	0	0	0	1
10/06/2021	F	12	0	0	0	0	0	0	0	0	0	0	1	0	1	0	0
26/08/2021	M	12	0	0	0	0	0	0	0	0	0	0	1	0	0	0	0
30/08/2021	M	12	0	0	0	0	0	0	0	0	0	0	0	0	0	0	0
30/08/2021	M	12	0	0	0	0	0	0	0	0	0	0	1	0	0	0	0
06.01.2021	F	12	0	0	0	0	0	0	0	0	0	0	0	0	0	0	1
28.05.2021	M	12	0	0	0	0	0	0	0	0	0	0	0	0	0	1	0

29/06/2022	M	12	0	0	0	0	0	0	0	0	0	0	1	0	1	0	0
15/12/2022	F	12	0	0	0	0	0	0	0	0	0	0	0	0	1	0	0
12/01/2023	M	12	0	0	0	0	0	0	0	0	0	0	0	0	0	0	0
20/03/2023	F	12	0	0	0	0	0	0	0	0	0	0	0	0	0	0	1
13/06/2023	F	12	0	0	0	0	0	0	0	0	0	0	0	0	0	0	1
14/06/2023	F	12	0	0	0	0	0	0	0	0	0	0	0	0	0	0	1
22/06/2023	M	12	0	0	0	0	0	0	0	0	0	0	0	0	0	0	0
29/06/2023	F	12	0	0	0	0	0	0	0	0	0	0	0	0	0	0	0
06/09/2023	F	12	0	0	0	0	0	0	0	0	0	0	0	0	0	0	1
13/10/2023	M	12	0	0	0	0	0	0	0	0	0	0	1	0	1	0	0
03/11/2023	M	12	0	0	0	0	0	0	0	0	0	0	1	0	1	0	0
05/01/2024	M	12	0	0	0	0	0	0	0	0	0	0	1	0	0	0	0
15/01/2024	M	12	0	0	0	0	0	0	0	0	0	0	0	0	0	0	1
29/01/2024	M	12	0	0	0	0	0	0	0	0	0	0	0	0	0	0	0
01/02/2024	F	12	0	0	0	0	0	0	0	0	0	0	0	0	0	0	0
08/03/2024	F	12	0	0	0	0	0	0	0	0	0	0	0	0	0	0	0
24/04/2024	F	12	0	0	0	0	0	0	0	0	0	0	0	0	0	0	1
01/07/2024	M	12	0	0	0	0	0	0	0	0	0	0	0	0	1	1	0
21/08/2024	M	12	0	0	0	0	0	0	0	0	0	0	1	0	0	0	1
29/10/2024	F	12	0	0	0	0	0	0	0	0	0	0	1	0	0	0	0
05.05.2021	F	12	0	0	0	0	0	0	0	0	0	1	0	0	0	0	0
19/01/2022	F	12	0	0	0	0	0	0	0	0	0	1	0	0	0	0	0
12/04/2023	F	12	0	0	0	0	0	0	0	0	1	0	0	0	0	0	1
16/01/2020	M	12	0	0	0	0	1	0	0	0	0	0	1	1	1	0	0
20/07/2020	M	12	0	1	0	0	0	0	0	0	0	0	1	1	1	0	0
15/10/2020	M	12	0	0	0	0	0	0	1	0	0	0	1	0	1	0	0
07/09/2021	F	12	0	0	1	0	0	0	0	0	0	0	1	1	0	0	0
03/02/2023	M	12	0	0	1	0	0	0	0	0	0	0	1	0	0	0	0
24/04/2023	M	12	0	0	1	0	0	0	0	0	0	0	1	1	0	0	0

07/11/2023	M	12	0	0	1	0	0	0	0	0	0	0	1	1	0	0	0
13/11/2023	M	12	0	0	1	0	0	0	0	0	0	0	1	0	1	0	0
18/09/2024	M	12	0	0	1	0	0	0	0	0	0	0	1	1	0	0	0
13/11/2024	M	12	0	0	1	0	0	0	0	0	0	0	1	1	0	0	0
06/06/2022	M	12	0	0	0	0	1	0	0	0	0	0	1	0	0	0	0
03/02/2023	M	12	0	0	0	0	1	0	0	0	0	0	1	0	0	0	0
21/02/2023	M	12	0	0	0	0	1	0	0	0	0	0	1	0	1	0	0
03/11/2023	F	12	0	0	0	0	1	0	0	0	0	0	1	0	0	0	0
20/05/2024	F	12	0	0	0	0	0	0	1	0	0	0	1	0	1	0	0
01/08/2024	F	12	0	0	0	0	1	0	0	0	0	0	1	0	1	0	0
22/01/2020	F	12	0	0	0	0	1	1	0	0	0	0	1	1	0	0	0
22/04/2021	F	12	0	0	0	0	1	0	1	0	0	0	1	0	1	0	0
10/06/2021	F	12	0	1	0	0	1	0	0	0	0	0	1	0	1	0	0
31/07/2020	F	12	0	1	1	0	0	0	0	0	0	0	1	1	0	0	0
25/05/2022	F	12	0	0	1	0	1	0	0	0	0	0	1	1	0	0	0
10/05/2023	F	12	0	1	1	0	0	0	0	0	0	0	1	0	0	0	0
07/05/2024	F	12	0	0	1	0	0	1	0	0	0	0	1	1	1	0	0
20/09/2022	M	12	0	0	0	0	1	1	0	0	0	0	1	1	1	0	0
10/10/2022	M	12	0	0	0	0	1	1	0	0	0	0	1	0	1	0	0
08/03/2023	F	12	1	0	0	0	1	0	0	0	0	0	1	0	0	0	1
12.05.2021	M	12	1	1	0	0	0	0	0	0	0	1	1	0	0	0	0
21.05.2021	M	12	0	1	1	0	0	0	0	0	0	1	1	1	0	0	1
09/11/2020	F	12	0	1	1	0	0	1	0	0	0	0	1	0	0	0	0
13/07/2021	M	12	1	1	1	0	0	0	0	0	0	0	1	1	0	0	1
23/08/2021	F	12	0	0	1	1	1	0	0	0	0	0	1	1	1	0	1
02/11/2021	M	12	1	1	1	0	0	0	0	0	0	0	1	1	0	0	0
25/09/2023	M	12	0	1	1	1	0	0	0	0	0	0	1	1	1	0	0
07/02/2024	M	12	0	1	1	0	1	0	0	0	0	0	1	1	0	0	0
01/10/2024	F	12	0	1	1	0	1	0	0	0	0	0	1	1	0	0	0

22/10/2024	F	12	0	1	1	0	1	0	0	0	0	0	1	1	0	0	1
29/10/2024	M	12	1	1	0	0	1	0	0	0	0	0	1	0	0	0	0
06/09/2021	M	12	0	1	1	0	1	1	0	0	0	0	1	1	0	0	0
31/03/2022	M	12	1	1	1	0	1	0	0	0	0	0	1	1	0	0	0
24/06/2022	M	12	1	1	1	0	1	0	0	0	0	0	1	1	1	0	0
30/01/2023	M	12	1	1	1	0	0	0	1	0	0	0	1	1	0	0	0
26/06/2024	M	12	1	1	1	0	1	0	0	0	0	0	1	0	1	0	0
01/11/2023	M	12	0	1	0	0	1	1	1	0	0	0	1	0	1	0	0
31/10/2023	F	12	1	1	1	0	0	1	0	1	0	0	1	0	0	0	0
30/08/2024	M	12	1	1	1	0	0	0	1	0	0	1	1	1	0	0	1
10.09.2021	M	12	1	1	1	1	0	0	0	0	0	1	1	0	0	0	0
18/02/2020	F	12	0	1	1	1	1	1	0	0	0	0	1	1	0	0	0
16/09/2021	F	12	1	1	1	1	0	1	0	0	0	0	1	1	0	0	0
07/08/2023	M	12	1	1	1	0	1	0	1	0	0	0	1	1	0	0	0
15/11/2023	F	12	1	1	1	0	1	1	0	0	0	0	1	0	0	0	0
15/04/2022	F	12	1	1	1	1	1	1	0	0	0	0	1	1	0	0	0
20/01/2020	F	13	0	0	0	0	0	0	0	0	0	0	0	0	1	0	0
24/02/2020	F	13	0	0	0	0	0	0	0	0	0	0	0	0	0	0	0
02/10/2020	M	13	0	0	0	0	0	0	0	0	0	0	0	0	0	0	0
05/10/2020	F	13	0	0	0	0	0	0	0	0	0	0	1	1	0	0	0
13/05/2021	F	13	0	0	0	0	0	0	0	0	0	0	1	1	0	0	0
29/07/2021	F	13	0	0	0	0	0	0	0	0	0	0	0	0	1	0	0
14/01/2022	F	13	0	0	0	0	0	0	0	0	0	0	1	0	0	0	1
24/03/2022	F	13	0	0	0	0	0	0	0	0	0	0	1	1	0	0	0
15/04/2022	M	13	0	0	0	0	0	0	0	0	0	0	0	0	0	0	0
31/10/2022	F	13	0	0	0	0	0	0	0	0	0	0	0	0	0	0	0
16/12/2022	M	13	0	0	0	0	0	0	0	0	0	0	0	0	0	0	1
06/03/2023	F	13	0	0	0	0	0	0	0	0	0	0	0	0	0	0	1
27/06/2023	M	13	0	0	0	0	0	0	0	0	0	0	1	0	0	0	0

14/12/2023	F	13	0	0	0	0	0	0	0	0	0	0	1	0	0	0	0
12/01/2024	F	13	0	0	0	0	0	0	0	0	0	0	0	0	0	0	1
14/03/2024	M	13	0	0	0	0	0	0	0	0	0	0	0	0	1	0	0
02/04/2024	M	13	0	0	0	0	0	0	0	0	0	0	0	0	0	0	1
22/04/2024	F	13	0	0	0	0	0	0	0	0	0	0	1	0	0	0	0
03/09/2021	F	13	0	0	0	0	0	0	0	0	0	1	1	0	0	0	0
05.11.2021	F	13	0	0	0	0	0	0	0	0	0	1	0	0	0	0	0
12.11.2021	F	13	0	0	0	0	0	0	0	0	0	1	0	0	0	0	0
20/04/2022	F	13	0	0	0	0	0	0	0	0	1	0	1	0	0	0	1
07/04/2023	M	13	0	0	0	0	0	0	0	0	1	0	1	0	1	0	0
01/09/2020	F	13	0	0	0	0	1	0	0	0	0	0	1	0	1	0	0
16/09/2021	F	13	0	1	0	0	0	0	0	0	0	0	1	0	0	0	0
31/08/2023	M	13	0	0	1	0	0	0	0	0	0	0	1	1	0	0	0
31/10/2023	M	13	0	0	1	0	0	0	0	0	0	0	1	1	0	0	0
26/10/2022	F	13	0	0	0	0	1	0	0	0	0	0	1	0	1	0	0
15/12/2022	M	13	0	0	0	0	1	0	0	0	0	0	1	0	1	0	0
19/05/2023	F	13	0	0	0	0	1	0	0	0	0	0	1	0	0	0	1
06/06/2023	F	13	0	0	0	0	1	0	0	0	0	0	1	0	1	0	0
16/06/2023	F	13	0	0	0	0	1	0	0	0	0	0	1	0	1	0	0
26/07/2023	M	13	0	0	0	0	1	0	0	0	0	0	1	0	1	0	0
31/10/2023	F	13	0	0	0	0	1	0	0	0	0	0	1	0	1	0	0
10/04/2024	F	13	0	0	0	0	1	0	0	0	0	0	1	0	1	0	0
21/05/2024	M	13	0	0	0	0	1	0	0	0	0	0	1	0	1	0	0
23/08/2024	M	13	0	0	0	0	1	0	0	0	0	0	1	0	0	0	0
23/07/2024	M	13	0	0	0	0	1	0	0	1	0	0	1	0	1	0	0
16/03/2020	F	13	0	0	0	0	1	1	0	0	0	0	1	1	0	0	0
16/07/2020	F	13	0	1	0	0	0	0	1	0	0	0	1	1	0	0	0
23/11/2020	F	13	1	0	1	0	0	0	0	0	0	0	1	1	0	0	0
24/02/2023	F	13	0	1	1	0	0	0	0	0	0	0	1	0	1	0	0

09/08/2023	F	13	0	0	1	0	1	0	0	0	0	0	1	0	0	0	0
23/01/2024	F	13	0	1	1	0	0	0	0	0	0	0	1	1	0	0	0
19/03/2024	M	13	0	1	1	0	0	0	0	0	0	0	1	1	0	0	0
03/09/2024	M	13	0	1	1	0	0	0	0	0	0	0	1	1	0	0	0
27/10/2022	M	13	0	1	0	0	0	0	1	0	0	0	1	0	1	0	0
11/05/2023	M	13	0	1	0	0	0	1	0	0	0	0	1	1	1	0	0
14/03/2024	M	13	0	0	0	0	1	1	0	0	0	0	1	0	1	0	0
13/04/2022	F	13	0	0	1	0	1	1	0	0	0	0	1	0	1	0	0
10/06/2022	F	13	0	0	1	1	0	1	0	0	0	0	1	0	0	0	0
23/05/2023	F	13	1	1	1	0	0	0	0	0	0	0	1	1	0	0	0
05/09/2024	M	13	0	1	1	0	1	0	0	0	0	0	1	1	1	0	0
27/09/2024	F	13	0	0	1	0	1	0	1	0	0	0	1	0	0	0	0
07/06/2021	M	13	1	1	0	1	0	1	0	0	0	0	1	1	0	0	0
10/06/2022	M	13	0	1	1	1	1	0	0	0	0	0	1	0	0	0	0
20/03/2024	M	13	0	1	1	1	0	1	0	0	0	0	1	0	0	0	0
05/07/2024	M	13	1	1	1	0	0	0	1	0	0	0	1	0	0	0	0
29/08/2024	M	13	1	1	1	0	1	0	1	0	0	0	1	1	0	0	0
22/07/2020	M	14	0	0	0	0	0	0	0	0	0	0	1	0	0	0	0
10/08/2020	F	14	0	0	0	0	0	0	0	0	0	0	0	0	0	0	1
17/09/2020	F	14	0	0	0	0	0	0	0	0	0	0	0	0	1	0	0
18/02/2021	F	14	0	0	0	0	0	0	0	0	0	0	0	0	0	0	0
15/06/2021	F	14	0	0	0	0	0	0	0	0	0	0	0	0	0	0	1
14/09/2021	F	14	0	0	0	0	0	0	0	0	0	0	1	1	0	0	0
22/11/2021	F	14	0	0	0	0	0	0	0	0	0	0	1	0	0	0	0
12.11.2021	F	14	0	0	0	0	0	0	0	0	0	0	0	0	0	1	0
20.01.2021	F	14	0	0	0	0	0	0	0	0	0	0	1	0	0	0	0
05/01/2022	M	14	0	0	0	0	0	0	0	0	0	0	0	0	0	0	1
10/01/2022	F	14	0	0	0	0	0	0	0	0	0	0	0	0	1	0	0
25/08/2022	M	14	0	0	0	0	0	0	0	0	0	0	0	0	0	0	1

09/09/2022	F	14	0	0	0	0	0	0	0	0	0	0	0	0	0	0	1
28/09/2022	M	14	0	0	0	0	0	0	0	0	0	0	1	0	0	0	0
30/01/2023	M	14	0	0	0	0	0	0	0	0	0	0	0	0	0	0	1
13/06/2023	F	14	0	0	0	0	0	0	0	0	0	0	0	0	0	0	1
29/09/2023	M	14	0	0	0	0	0	0	0	0	0	0	1	0	0	0	0
10/10/2024	F	14	0	0	0	0	0	0	0	0	0	0	0	0	0	0	0
26/11/2024	M	14	0	0	0	0	0	0	0	0	0	0	0	0	0	0	0
25/05/2023	M	14	0	0	0	0	0	0	0	0	0	1	0	0	0	0	0
03/05/2024	F	14	0	0	0	0	0	0	0	0	1	0	0	0	0	0	1
26.11.2021	F	14	0	0	0	0	0	0	0	1	0	1	0	1	0	1	0
20/06/2022	F	14	0	0	0	0	0	0	0	1	1	0	0	0	0	0	0
17/07/2020	M	14	0	1	0	0	0	0	0	0	0	0	1	0	0	0	0
03/09/2020	M	14	0	0	0	0	1	0	0	0	0	0	1	0	1	0	0
08/02/2021	F	14	0	0	0	0	1	0	0	0	0	0	1	0	0	0	1
09/02/2021	M	14	0	0	0	0	1	0	0	0	0	0	1	1	0	0	0
25/11/2021	F	14	0	0	0	1	0	0	0	0	0	0	1	0	0	0	0
08/04/2021	M	14	0	0	1	0	0	0	0	0	0	0	1	0	0	0	0
20/04/2022	M	14	0	0	0	0	1	0	0	0	0	0	1	1	1	0	0
27/06/2022	M	14	0	0	0	0	1	0	0	0	0	0	1	1	0	0	0
20/07/2022	M	14	0	1	0	0	0	0	0	0	0	0	1	0	0	0	0
10/05/2023	F	14	0	0	0	0	0	1	0	0	0	0	1	0	0	0	0
11/10/2023	F	14	0	0	0	0	1	0	0	0	0	0	1	0	1	0	0
21/11/2023	M	14	0	0	0	0	1	0	0	0	0	0	1	0	0	0	0
23/04/2024	M	14	0	0	0	0	1	0	0	0	0	0	1	0	0	0	0
01/10/2024	M	14	0	0	0	0	1	0	0	0	0	0	1	1	0	0	0
29/10/2024	F	14	0	0	0	0	1	0	0	0	0	0	1	0	1	0	0
08/09/2021	M	14	0	0	0	0	1	0	0	0	0	1	1	0	0	1	1
03/09/2020	M	14	0	1	1	0	0	0	0	0	0	0	1	0	0	0	0
03/09/2020	M	14	0	1	1	0	0	0	0	0	0	0	1	0	0	0	0

18/10/2021	M	14	0	0	1	0	1	0	0	0	0	0	1	1	0	0	0
08/03/2022	F	14	0	1	1	0	0	0	0	0	0	0	1	1	0	0	0
13/04/2022	F	14	1	0	1	0	0	0	0	0	0	0	1	0	1	0	0
17/05/2022	F	14	0	0	1	0	0	1	0	0	0	0	1	1	0	0	0
07/02/2024	F	14	0	0	1	0	1	0	0	0	0	0	1	0	0	0	0
14/02/2022	F	14	0	0	0	0	1	0	1	0	0	0	1	0	0	0	0
13/04/2022	F	14	0	0	0	0	1	0	1	0	0	0	1	1	0	0	0
13/05/2024	M	14	0	1	0	0	1	0	0	0	0	0	1	1	0	0	0
01/10/2021	M	14	0	1	0	0	1	0	0	0	0	1	0	1	0	0	0
23/09/2020	M	14	0	1	0	0	1	1	0	0	0	0	1	0	1	0	0
07/03/2022	F	14	1	1	1	0	0	0	0	0	0	0	1	0	1	0	0
23/05/2022	M	14	1	1	1	0	0	0	0	0	0	0	1	1	0	0	0
20/02/2023	M	14	1	0	1	0	0	0	1	0	0	0	1	0	1	0	0
23/09/2021	F	14	0	1	1	1	0	0	0	0	0	1	1	0	0	0	0
16/09/2020	M	14	0	1	1	0	0	1	1	0	0	0	1	0	0	0	0
06/11/2020	F	14	1	1	1	0	1	0	0	0	0	0	1	1	0	0	0
02/11/2023	F	14	1	1	1	0	1	0	0	0	0	0	1	1	1	0	0
22/02/2021	M	14	1	1	1	0	0	0	1	0	1	0	1	0	0	0	0
20/03/2023	F	14	1	1	1	1	1	1	1	0	0	0	1	0	1	0	0
21/09/2020	F	15	0	0	0	0	0	0	0	0	0	0	0	0	0	0	1
22/10/2020	F	15	0	0	0	0	0	0	0	0	0	0	1	0	1	0	0
26/10/2020	M	15	0	0	0	0	0	0	0	0	0	0	0	0	0	0	1
15/12/2020	M	15	0	0	0	0	0	0	0	0	0	0	0	0	0	0	0
01/03/2021	M	15	0	0	0	0	0	0	0	0	0	0	0	0	0	0	0
27/05/2021	M	15	0	0	0	0	0	0	0	0	0	0	1	1	0	0	0
15/09/2021	F	15	0	0	0	0	0	0	0	0	0	0	0	0	0	1	0
27/09/2021	F	15	0	0	0	0	0	0	0	0	0	0	1	0	0	0	1
14/01/2022	F	15	0	0	0	0	0	0	0	0	0	0	0	0	0	0	1
01/02/2022	M	15	0	0	0	0	0	0	0	0	0	0	1	0	1	0	0

23/02/2022	F	15	0	0	0	0	0	0	0	0	0	0	0	0	0	0	0
21/06/2022	M	15	0	0	0	0	0	0	0	0	0	0	0	0	0	0	1
05/07/2022	F	15	0	0	0	0	0	0	0	0	0	0	1	0	0	0	0
08/02/2023	F	15	0	0	0	0	0	0	0	0	0	0	0	0	0	0	1
18/05/2023	F	15	0	0	0	0	0	0	0	0	0	0	0	0	0	0	0
23/08/2023	F	15	0	0	0	0	0	0	0	0	0	0	1	0	0	0	1
25/09/2023	F	15	0	0	0	0	0	0	0	0	0	0	1	0	1	0	0
18/01/2024	F	15	0	0	0	0	0	0	0	0	0	0	0	0	0	0	0
22/05/2024	M	15	0	0	0	0	0	0	0	0	0	0	0	0	0	0	0
30/08/2024	M	15	0	0	0	0	0	0	0	0	0	0	0	0	0	0	0
24/09/2020	M	15	0	0	0	0	0	0	0	1	0	0	1	0	1	0	0
20/08/2024	F	15	0	0	0	0	0	0	0	0	1	0	0	0	0	0	0
06/07/2020	M	15	0	0	0	0	1	0	0	0	0	0	1	0	1	0	0
20/07/2020	M	15	0	1	0	0	0	0	0	0	0	0	1	1	0	0	1
06/08/2020	M	15	0	0	0	0	1	0	0	0	0	0	1	0	1	0	0
17/09/2020	M	15	0	0	0	0	1	0	0	0	0	0	1	0	1	0	0
12/05/2023	F	15	0	0	1	0	0	0	0	0	0	0	1	0	0	0	0
24/03/2022	M	15	0	0	0	0	1	0	0	0	0	0	1	0	1	0	0
21/06/2022	M	15	0	1	0	0	0	0	0	0	0	0	1	1	0	0	0
16/02/2023	M	15	0	0	0	0	1	0	0	0	0	0	0	0	0	0	0
06/09/2023	F	15	0	1	0	0	0	0	0	0	0	0	1	0	0	0	0
13/11/2023	M	15	0	0	0	0	1	0	0	0	0	0	1	0	1	0	0
20/06/2024	M	15	0	1	0	0	0	0	0	0	0	0	1	0	0	0	0
03/09/2024	M	15	0	0	0	0	1	0	0	0	0	0	1	0	1	0	0
17/03/2023	M	15	0	1	0	0	0	0	0	0	1	0	1	0	0	0	1
18/06/2020	M	15	0	1	0	0	1	0	0	0	0	0	1	1	1	0	0
24/08/2021	M	15	0	1	1	0	0	0	0	0	0	0	1	0	0	0	0
17/06/2022	M	15	0	0	1	0	1	0	0	0	0	0	1	0	1	0	0
14/04/2022	M	15	0	1	0	0	1	0	0	0	0	0	1	0	1	0	0

23/08/2022	F	15	0	0	0	0	1	1	0	0	0	0	1	0	1	0	0
16/10/2020	F	15	0	1	0	1	0	0	1	0	0	0	1	1	1	0	0
25/11/2020	M	15	1	0	0	0	1	0	1	0	0	0	1	1	0	0	0
29/06/2023	M	15	0	1	1	0	1	0	0	0	0	0	1	0	0	0	0
06/09/2023	M	15	0	1	1	0	0	0	1	0	0	0	1	0	0	0	0
20/09/2023	M	15	1	0	0	0	1	1	0	0	0	0	1	0	0	0	0
21.05.2021	F	15	0	1	1	1	0	0	0	0	0	1	1	0	0	0	0
28/06/2023	F	15	1	1	1	0	0	1	1	0	0	0	1	0	0	0	1
22/06/2020	F	15	1	1	1	1	1	1	1	0	0	0	1	0	1	0	0
23/01/2020	F	16	0	0	0	0	0	0	0	0	0	0	1	0	0	0	0
29/06/2021	M	16	0	0	0	0	0	0	0	0	0	0	1	1	0	0	1
06/07/2021	F	16	0	0	0	0	0	0	0	0	0	0	0	0	0	0	1
22/07/2021	M	16	0	0	0	0	0	0	0	0	0	0	1	0	0	0	0
23/11/2021	F	16	0	0	0	0	0	0	0	0	0	0	1	0	0	0	0
02/02/2022	F	16	0	0	0	0	0	0	0	0	0	0	0	0	0	0	0
18/02/2022	F	16	0	0	0	0	0	0	0	0	0	0	0	0	0	0	0
05/04/2022	F	16	0	0	0	0	0	0	0	0	0	0	0	0	1	0	1
27/09/2022	F	16	0	0	0	0	0	0	0	0	0	0	0	0	1	0	0
18/11/2022	F	16	0	0	0	0	0	0	0	0	0	0	0	0	0	0	0
21/11/2022	F	16	0	0	0	0	0	0	0	0	0	0	0	0	0	0	0
13/01/2023	F	16	0	0	0	0	0	0	0	0	0	0	0	0	0	0	1
02/02/2023	F	16	0	0	0	0	0	0	0	0	0	0	0	0	0	0	1
11/05/2023	F	16	0	0	0	0	0	0	0	0	0	0	0	0	0	0	1
17/08/2023	F	16	0	0	0	0	0	0	0	0	0	0	0	0	0	0	1
27/02/2024	M	16	0	0	0	0	0	0	0	0	0	0	1	0	0	0	0
09/07/2024	M	16	0	0	0	0	0	0	0	0	0	0	0	0	0	0	0
26/09/2024	F	16	0	0	0	0	0	0	0	0	0	0	0	0	1	0	0
04/09/2020	M	16	0	0	0	0	0	0	0	0	0	1	1	1	0	0	0
15.09.2021	F	16	0	0	0	0	0	0	0	0	0	1	0	0	0	1	0

15/11/2024	M	16	0	0	0	0	0	0	0	0	1	0	0	0	0	0	0
10/02/2020	F	16	0	0	0	0	1	0	0	0	0	0	1	0	1	0	0
16/07/2020	M	16	0	1	0	0	0	0	0	0	0	0	1	0	0	0	0
14/01/2021	M	16	0	0	0	0	0	0	1	0	0	0	1	0	0	0	0
06/09/2021	F	16	0	0	0	0	1	0	0	0	0	0	1	0	0	0	1
06.01.2021	F	16	0	0	0	0	1	0	0	0	0	0	1	0	0	0	0
04/11/2021	F	16	0	0	1	0	0	0	0	0	0	0	1	0	1	0	0
02/02/2023	M	16	0	0	1	0	0	0	0	0	0	0	1	1	0	0	0
31/10/2023	M	16	0	0	1	0	0	0	0	0	0	0	1	1	0	0	0
03/09/2024	M	16	0	0	1	0	0	0	0	0	0	0	1	1	0	0	0
17/05/2022	F	16	0	0	0	0	1	0	0	0	0	0	0	0	0	0	0
21/04/2023	F	16	0	0	0	0	1	0	0	0	0	0	1	0	0	0	0
19/07/2023	M	16	0	1	0	0	0	0	0	0	0	0	1	0	0	0	0
02/08/2023	M	16	0	1	0	0	0	0	0	0	0	0	1	0	0	0	0
17/11/2023	M	16	0	0	0	0	1	0	0	0	0	0	1	0	1	0	0
20/06/2024	F	16	0	0	0	0	1	0	0	0	0	0	1	1	0	0	0
13/10/2021	M	16	0	0	0	0	1	0	0	0	0	1	0	0	0	0	0
22.09.2021	M	16	0	0	1	0	0	0	0	0	0	1	0	0	0	1	0
23/12/2020	M	16	0	0	0	0	1	0	1	0	0	0	1	1	1	1	0
16/10/2020	M	16	0	0	1	0	1	0	0	0	0	0	1	1	1	0	0
13/07/2022	F	16	0	1	1	0	0	0	0	0	0	0	1	0	0	0	0
07/09/2023	M	16	0	1	1	0	0	0	0	0	0	0	1	1	0	0	0
06/12/2022	M	16	0	0	0	0	1	1	0	0	0	0	1	0	1	1	0
29/03/2023	F	16	0	1	0	0	1	0	0	0	0	0	1	0	0	0	1
09/07/2024	F	16	0	1	0	0	0	1	0	0	0	0	1	1	0	0	0
22/08/2024	F	16	0	0	0	0	1	0	1	0	0	0	1	0	0	0	0
14/06/2021	M	16	1	1	0	1	0	0	0	0	0	0	1	1	0	0	0
26/10/2020	M	16	1	0	1	0	0	1	0	0	0	0	1	1	1	0	0
02/11/2022	M	16	0	1	1	0	0	0	1	0	0	0	1	0	1	0	0

02/11/2022	M	16	0	1	1	0	0	0	1	0	0	0	1	0	1	0	0
08/11/2023	M	16	0	1	1	0	1	0	0	0	0	0	1	0	0	0	0
21/01/2022	F	16	0	1	0	0	1	0	1	0	0	0	1	0	0	0	0
20/07/2022	F	16	0	1	0	0	1	1	0	0	0	0	1	1	1	0	0
02/11/2022	F	16	0	1	1	1	0	0	1	0	0	0	1	1	0	0	0
05.05.2021	F	16	0	1	0	0	1	1	1	0	0	1	1	1	0	0	0
14/07/2020	M	16	1	1	1	1	0	0	0	1	0	0	1	1	0	0	0
16/01/2020	F	16	1	1	1	1	1	0	0	0	0	0	1	1	0	0	1
17/01/2023	F	16	1	1	1	1	1	1	0	0	0	1	1	1	0	0	0
09/09/2020	M	17	0	0	0	0	0	0	0	0	0	0	1	0	1	0	0
02/10/2020	M	17	0	0	0	0	0	0	0	0	0	0	0	0	0	0	0
22/02/2021	F	17	0	0	0	0	0	0	0	0	0	0	1	0	0	0	1
31/03/2022	F	17	0	0	0	0	0	0	0	0	0	0	0	0	0	0	1
27/04/2022	F	17	0	0	0	0	0	0	0	0	0	0	1	0	0	0	1
17/06/2022	F	17	0	0	0	0	0	0	0	0	0	0	1	0	0	0	1
21/07/2022	F	17	0	0	0	0	0	0	0	0	0	0	0	0	0	0	1
31/08/2022	F	17	0	0	0	0	0	0	0	0	0	0	0	0	0	0	1
08/09/2022	F	17	0	0	0	0	0	0	0	0	0	0	0	0	0	0	1
02/11/2022	F	17	0	0	0	0	0	0	0	0	0	0	1	0	0	0	0
02/11/2022	F	17	0	0	0	0	0	0	0	0	0	0	1	0	0	0	0
06/08/2023	F	17	0	0	0	0	0	0	0	0	0	0	0	0	0	0	1
25/09/2023	F	17	0	0	0	0	0	0	0	0	0	0	0	0	0	0	0
27/11/2023	F	17	0	0	0	0	0	0	0	0	0	0	1	0	0	0	0
22/05/2024	F	17	0	0	0	0	0	0	0	0	0	0	0	0	0	0	1
23/09/2020	F	17	0	0	0	0	1	0	0	0	0	0	1	0	1	0	0
24/09/2020	F	17	0	0	0	0	1	0	0	0	0	0	1	0	0	0	0
19/10/2020	M	17	0	0	0	0	0	0	1	0	0	0	1	1	1	0	0
16/12/2020	F	17	0	0	0	0	0	1	0	0	0	0	0	0	0	0	0
20/09/2022	F	17	0	0	1	0	0	0	0	0	0	0	1	1	0	0	0

20/09/2022	F	17	0	0	1	0	0	0	0	0	0	0	1	1	0	0	0
02/09/2024	F	17	0	0	1	0	0	0	0	0	0	0	1	1	0	0	0
25/05/2022	M	17	1	0	0	0	0	0	0	0	0	0	1	0	0	0	0
11/01/2023	F	17	0	0	0	0	1	0	0	0	0	0	1	0	0	0	0
27/03/2023	M	17	0	0	0	0	1	0	0	0	0	0	1	0	0	0	0
23/05/2023	M	17	0	0	0	0	1	0	0	0	0	0	1	1	0	0	0
20/11/2023	M	17	0	0	0	0	1	0	0	0	0	0	1	0	0	0	0
16/10/2024	M	17	0	0	0	0	0	1	0	0	0	0	1	0	1	0	0
02/12/2020	M	17	0	0	1	0	1	0	0	0	0	0	1	1	0	0	1
26/09/2022	F	17	0	0	1	0	0	1	0	0	0	0	1	0	0	0	0
11/10/2022	F	17	0	0	1	0	0	1	0	0	0	0	0	0	1	0	1
22/06/2022	F	17	0	1	0	0	1	0	0	0	0	0	1	1	0	0	0
27/06/2023	M	17	0	1	0	0	1	0	0	0	0	0	1	0	0	0	1
28/07/2023	M	17	0	0	0	0	1	1	0	0	0	0	1	0	0	0	0
01/03/2024	M	17	0	0	0	0	0	1	1	0	0	0	1	0	0	0	0
21/05/2024	M	17	0	1	0	0	0	1	0	0	0	0	1	1	1	0	0
01/02/2022	F	17	0	1	1	0	0	0	0	1	0	0	1	0	0	0	0
31/01/2024	F	17	0	0	1	0	0	1	0	1	0	0	1	1	0	0	0
29/04/2022	F	17	0	1	1	0	0	1	0	0	0	0	1	1	0	0	0
10/06/2022	F	17	1	0	1	0	0	1	0	0	0	0	1	1	0	0	0
20/09/2023	F	17	0	1	1	0	1	0	0	0	0	0	1	0	0	0	0
18/06/2024	F	17	1	0	1	0	0	0	1	0	0	0	1	1	0	0	0
10/03/2022	F	17	0	1	0	0	1	1	1	0	0	0	1	0	1	0	1
07/12/2022	M	17	1	1	0	0	1	1	0	0	0	0	1	0	0	0	0
13/08/2020	M	17	1	1	1	0	0	1	1	0	0	0	1	1	0	0	0
20/12/2022	M	17	1	1	1	0	0	1	1	0	0	0	1	1	0	0	1
02/07/2024	M	17	1	1	1	0	1	1	0	0	0	0	1	1	0	0	0
28/08/2024	M	17	0	1	1	1	1	0	1	0	0	0	1	1	0	0	0
29/10/2024	M	17	1	1	1	1	1	0	0	0	0	0	1	1	0	0	0

26/08/2021	F	17	1	1	1	1	1	1	1	0	0	0	1	1	0	0	0
09/01/2020	M	18	0	0	0	0	0	0	0	0	0	0	0	0	0	0	0
17/02/2020	M	18	0	0	0	0	0	0	0	0	0	0	0	0	0	0	0
27/02/2020	F	18	0	0	0	0	0	0	0	0	0	0	1	0	0	0	0
02/03/2020	F	18	0	0	0	0	0	0	0	0	0	0	0	0	1	0	0
11/03/2020	M	18	0	0	0	0	0	0	0	0	0	0	0	0	0	0	0
06/07/2020	M	18	0	0	0	0	0	0	0	0	0	0	0	0	0	0	0
07/10/2020	F	18	0	0	0	0	0	0	0	0	0	0	0	0	0	0	1
12/10/2020	M	18	0	0	0	0	0	0	0	0	0	0	1	0	0	0	0
04/12/2020	M	18	0	0	0	0	0	0	0	0	0	0	1	0	1	0	0
23/08/2021	M	18	0	0	0	0	0	0	0	0	0	0	1	1	0	0	0
07/10/2021	F	18	0	0	0	0	0	0	0	0	0	0	0	0	0	0	0
18/01/2022	M	18	0	0	0	0	0	0	0	0	0	0	0	0	0	0	0
26/05/2022	M	18	0	0	0	0	0	0	0	0	0	0	0	0	0	0	1
11/10/2022	F	18	0	0	0	0	0	0	0	0	0	0	0	0	0	0	1
23/11/2022	M	18	0	0	0	0	0	0	0	0	0	0	1	0	0	0	0
14/02/2023	M	18	0	0	0	0	0	0	0	0	0	0	0	0	0	0	0
08/08/2023	F	18	0	0	0	0	0	0	0	0	0	0	0	0	0	0	1
20/03/2024	M	18	0	0	0	0	0	0	0	0	0	0	0	0	1	0	0
20/08/2024	M	18	0	0	0	0	0	0	0	0	0	0	0	0	0	0	0
28/08/2024	F	18	0	0	0	0	0	0	0	0	0	0	0	0	0	0	1
13/12/2024	F	18	0	0	0	0	0	0	0	0	0	0	0	0	0	0	1
06/01/2022	F	18	0	0	1	0	0	0	0	0	0	0	1	1	1	0	0
05/09/2023	F	18	0	0	1	0	0	0	0	0	0	0	1	1	1	1	0
02/10/2023	F	18	0	0	1	0	0	0	0	0	0	0	1	1	0	0	0
30/09/2022	M	18	0	0	0	0	1	0	0	0	0	0	1	0	0	0	0
04/07/2024	M	18	0	1	0	0	0	0	0	0	0	0	1	1	0	0	0
14/11/2024	M	18	0	0	0	0	1	0	0	0	0	0	1	1	1	0	0
13/05/2021	F	18	0	0	0	0	0	0	1	0	0	1	0	0	1	0	0

25/02/2022	M	18	0	0	1	1	0	0	0	0	0	0	1	1	0	0	0
25/11/2022	M	18	1	0	0	0	1	0	0	0	0	0	1	0	1	0	1
30/09/2021	M	18	0	1	1	0	0	0	0	1	0	0	1	1	0	0	0
24/03/2022	F	18	1	0	0	0	0	0	1	1	0	0	1	1	0	0	0
14/07/2020	F	18	0	1	0	1	0	1	0	0	0	0	1	1	0	0	0
24/09/2020	M	18	0	1	0	0	0	1	1	0	0	0	1	1	0	0	0
31/08/2020	F	18	1	1	1	0	0	0	0	0	0	0	1	1	0	0	0
09/10/2023	F	18	0	1	1	0	1	1	0	0	0	0	1	0	0	0	0
12/01/2022	F	18	0	1	1	0	1	1	1	0	0	0	1	0	1	0	0
21/05/2024	M	18	1	1	1	1	0	1	0	0	0	0	1	1	0	0	0
25/05/2024	F	18	1	1	1	0	1	1	0	0	0	0	1	1	0	0	1
25/02/2020	F	19	0	0	0	0	0	0	0	0	0	0	0	0	0	0	1
07/08/2020	F	19	0	0	0	0	0	0	0	0	0	0	0	0	0	0	0
04/11/2020	F	19	0	0	0	0	0	0	0	0	0	0	1	0	0	0	0
14/12/2021	F	19	0	0	0	0	0	0	0	0	0	0	1	0	1	0	0
15.12.2021	F	19	0	0	0	0	0	0	0	0	0	0	0	0	0	1	0
27/04/2022	M	19	0	0	0	0	0	0	0	0	0	0	0	0	0	0	1
26/07/2022	F	19	0	0	0	0	0	0	0	0	0	0	0	0	0	0	1
14/12/2022	M	19	0	0	0	0	0	0	0	0	0	0	1	0	0	0	0
16/12/2022	M	19	0	0	0	0	0	0	0	0	0	0	0	0	0	0	0
16/12/2022	M	19	0	0	0	0	0	0	0	0	0	0	0	0	0	0	0
21/12/2022	M	19	0	0	0	0	0	0	0	0	0	0	1	0	0	0	0
06/04/2023	F	19	0	0	0	0	0	0	0	0	0	0	0	0	0	0	0
22/05/2023	M	19	0	0	0	0	0	0	0	0	0	0	1	0	0	0	0
16/06/2023	F	19	0	0	0	0	0	0	0	0	0	0	0	0	0	0	1
01/08/2023	F	19	0	0	0	0	0	0	0	0	0	0	1	0	1	0	0
10/10/2023	F	19	0	0	0	0	0	0	0	0	0	0	1	0	0	0	0
06/09/2024	M	19	0	0	0	0	0	0	0	0	0	0	0	0	1	0	0
10/12/2024	M	19	0	0	0	0	0	0	0	0	0	0	0	0	0	0	0

14/09/2021	M	19	0	0	0	0	0	0	1	0	0	0	1	0	1	0	0
20/12/2021	F	19	0	0	0	0	1	0	0	0	0	0	1	0	1	0	0
06/08/2020	M	19	0	0	1	0	0	0	0	0	0	0	1	0	0	0	0
25/11/2022	M	19	0	0	1	0	0	0	0	0	0	0	1	1	0	0	0
26/09/2023	F	19	0	0	1	0	0	0	0	0	0	0	1	0	1	0	0
12/09/2022	F	19	0	0	0	0	1	0	0	0	0	0	1	0	0	0	1
12/10/2023	M	19	0	1	0	0	0	0	0	0	0	0	1	0	0	0	0
12/04/2024	M	19	0	0	0	0	1	0	0	0	0	0	0	1	0	0	1
28.04.2021	M	19	0	0	1	0	0	0	0	0	0	1	0	1	0	0	0
12/04/2022	F	19	0	0	0	0	0	0	1	1	0	0	1	1	0	0	0
09/06/2020	M	19	0	1	0	0	1	0	0	0	0	0	1	0	1	0	0
07/10/2021	F	19	0	1	0	1	0	0	0	0	0	0	1	1	1	0	0
25/05/2024	M	19	0	1	0	0	1	0	0	0	0	0	1	1	0	0	1
01/07/2020	M	19	0	0	1	1	0	0	0	0	0	0	1	1	0	0	0
03/10/2022	F	19	0	0	1	0	1	0	0	0	0	0	1	0	1	0	0
04/11/2022	F	19	0	1	1	0	0	0	0	0	0	0	1	1	0	0	0
27/01/2022	F	19	0	0	0	0	1	1	0	0	0	0	1	0	1	0	0
20/11/2024	F	19	1	0	0	0	0	1	0	0	0	0	1	0	0	0	0
11/03/2020	F	19	1	1	0	0	0	0	0	0	0	1	1	1	0	0	0
15.12.2021	F	19	0	1	0	0	0	0	1	0	0	1	0	1	0	0	0
15/06/2020	F	19	1	1	0	0	0	0	1	0	0	0	1	0	0	0	0
19/06/2020	F	19	0	0	1	0	1	1	0	0	0	0	1	1	0	0	0
20/02/2023	F	19	1	0	0	0	1	0	1	0	0	0	1	0	0	0	0
08/10/2020	F	20	0	0	0	0	0	0	0	0	0	0	1	0	1	0	0
17/11/2020	F	20	0	0	0	0	0	0	0	0	0	0	0	0	0	0	1
26/01/2021	F	20	0	0	0	0	0	0	0	0	0	0	0	0	0	0	0
15/06/2021	F	20	0	0	0	0	0	0	0	0	0	0	0	0	0	0	0
22/02/2023	F	20	0	0	0	0	0	0	0	0	0	0	1	0	0	0	0
02/03/2023	F	20	0	0	0	0	0	0	0	0	0	0	1	0	1	0	0

20/09/2023	F	20	0	0	0	0	0	0	0	0	0	0	0	0	0	0	1
22/05/2024	M	20	0	0	0	0	0	0	0	0	0	0	0	0	0	0	1
01/10/2024	F	20	0	0	0	0	0	0	0	0	0	0	0	0	0	0	1
09/10/2024	F	20	0	0	0	0	0	0	0	0	0	0	0	0	0	0	0
13/11/2024	F	20	0	0	0	0	0	0	0	0	0	0	0	0	0	0	1
12/12/2024	F	20	0	0	0	0	0	0	0	0	0	0	0	0	0	0	0
03.02.2021	F	20	0	0	0	0	0	0	0	0	1	0	0	0	0	0	0
01/09/2022	F	20	0	0	0	0	0	0	0	0	0	1	0	0	0	0	1
22/09/2020	F	20	1	0	0	0	0	0	0	0	0	0	1	1	0	0	0
28/03/2022	M	20	0	0	1	0	0	0	0	0	0	0	1	0	0	0	0
27/05/2022	F	20	1	0	0	0	0	0	0	0	0	0	1	0	0	0	1
16/03/2023	M	20	0	0	0	0	1	0	0	0	0	0	1	0	0	0	0
25/10/2023	F	20	0	1	0	0	0	0	0	0	0	0	1	0	0	0	0
12/07/2022	M	20	0	1	0	0	1	0	0	0	0	0	1	1	0	0	0
05/01/2024	F	20	0	1	0	0	1	0	0	0	0	0	1	1	0	0	0
10/05/2024	M	20	1	1	0	0	0	0	0	0	0	0	1	1	0	0	0
19/06/2024	F	20	0	0	1	1	0	0	0	1	0	0	1	1	0	0	1
03/02/2023	M	20	0	1	1	1	0	0	0	0	0	0	1	0	0	0	0
28/06/2023	M	20	0	1	1	0	0	0	1	0	0	0	1	0	1	0	0
26/07/2023	F	20	0	1	0	0	1	1	0	0	0	0	1	0	1	0	0
21/02/2024	F	20	1	1	0	1	0	0	0	0	0	0	1	1	0	0	0
16/07/2024	F	20	1	0	0	0	1	1	0	0	0	0	1	1	1	0	0
12/04/2023	F	20	1	1	1	0	1	0	0	0	0	0	1	0	1	0	0
06/11/2023	F	20	1	1	1	1	0	0	0	0	0	0	1	1	0	0	0
07/05/2024	F	20	1	0	1	0	1	1	0	0	0	0	1	1	0	0	0
28/08/2024	F	20	1	1	1	0	1	0	0	0	0	0	1	1	1	0	0
09/11/2021	M	20	1	1	1	0	0	1	1	0	0	0	1	0	1	0	0
04/04/2021	F	20	0	1	1	1	1	1	0	1	0	0	1	0	0	0	1
16/02/2023	F	20	1	1	1	1	1	1	1	0	0	0	1	1	0	0	0

17/03/2020	F	21	0	0	0	0	0	0	0	0	0	0	0	0	0	0	0
19/05/2020	F	21	0	0	0	0	0	0	0	0	0	0	1	0	0	0	0
01/02/2021	F	21	0	0	0	0	0	0	0	0	0	0	0	0	1	0	0
16/09/2021	F	21	0	0	0	0	0	0	0	0	0	0	1	0	1	0	0
07/10/2021	M	21	0	0	0	0	0	0	0	0	0	0	0	0	1	0	0
01.09.2021	M	21	0	0	0	0	0	0	0	0	0	0	0	0	0	0	0
17.03.2021	F	21	0	0	0	0	0	0	0	0	0	0	1	1	0	1	1
19.11.2021	F	21	0	0	0	0	0	0	0	0	0	0	0	0	0	0	0
12/04/2022	F	21	0	0	0	0	0	0	0	0	0	0	0	0	0	0	0
20/05/2022	M	21	0	0	0	0	0	0	0	0	0	0	0	0	0	0	0
24/06/2022	F	21	0	0	0	0	0	0	0	0	0	0	0	0	0	0	0
05/09/2022	M	21	0	0	0	0	0	0	0	0	0	0	1	0	1	0	0
27/10/2022	F	21	0	0	0	0	0	0	0	0	0	0	1	0	1	0	0
04/11/2022	F	21	0	0	0	0	0	0	0	0	0	0	0	0	0	0	1
08/02/2023	F	21	0	0	0	0	0	0	0	0	0	0	0	0	0	0	1
26/01/2024	F	21	0	0	0	0	0	0	0	0	0	0	0	0	0	0	1
18/10/2024	F	21	0	0	0	0	0	0	0	0	0	0	0	0	0	0	1
16/10/2020	M	21	0	0	0	0	0	0	0	0	0	1	0	0	0	0	0
08/09/2021	F	21	0	0	0	0	0	0	0	0	0	1	0	1	0	0	0
08/10/2021	F	21	0	0	0	0	0	0	0	0	1	0	0	0	0	0	0
26.05.2021	F	21	0	0	0	0	0	0	0	0	0	1	1	0	0	1	0
10/01/2022	F	21	0	0	0	0	0	0	0	0	1	0	0	0	0	0	0
06/06/2024	M	21	0	0	0	0	0	0	0	0	1	0	1	0	0	0	0
27/01/2020	M	21	0	0	0	0	1	0	0	0	0	0	1	0	1	0	0
28/03/2022	F	21	0	0	1	0	0	0	0	0	0	0	1	0	0	0	0
05/09/2022	F	21	0	0	1	0	0	0	0	0	0	0	1	1	0	0	1
07/12/2022	F	21	0	0	1	0	0	0	0	0	0	0	1	0	0	0	0
28/03/2022	F	21	0	0	0	0	0	1	0	0	0	0	1	0	0	0	0
07/11/2022	F	21	0	0	0	0	1	0	0	0	0	0	1	0	1	0	0

10/03/2020	M	21	1	1	0	0	0	0	0	0	0	0	1	1	0	0	0
13/05/2021	F	21	0	0	0	0	1	1	0	0	0	0	1	0	1	0	0
12/05/2022	M	21	0	0	1	0	1	0	0	0	0	0	1	1	1	0	0
06/09/2023	M	21	0	1	1	0	0	0	0	0	0	0	1	0	0	0	0
05/04/2024	M	21	0	0	1	0	0	1	0	0	0	0	1	0	0	0	0
24/10/2022	M	21	0	1	0	0	1	0	0	0	0	0	1	0	1	0	0
29/04/2024	F	21	0	0	0	0	0	1	1	0	0	0	1	0	0	0	0
18/03/2021	F	21	1	1	0	0	1	0	0	0	0	0	1	1	0	0	1
11/02/2021	M	21	0	0	1	0	1	1	0	0	0	0	1	1	1	0	0
03/10/2022	M	21	0	0	1	0	1	1	0	0	0	0	1	1	0	0	1
18/03/2022	F	21	1	1	0	0	1	0	0	0	0	0	1	1	0	0	0
19/10/2022	F	21	1	1	0	0	1	0	0	0	0	0	1	1	1	0	0
23/08/2023	F	21	1	1	0	1	0	0	0	0	0	0	1	0	0	0	1
03/07/2024	F	21	0	0	0	1	0	1	1	0	0	0	1	0	0	0	0
31/01/2024	F	21	0	1	1	1	0	1	1	0	0	0	1	1	1	0	0
13/01/2020	M	22	0	0	0	0	0	0	0	0	0	0	0	0	0	0	1
04/03/2020	F	22	0	0	0	0	0	0	0	0	0	0	0	0	1	0	0
28/07/2020	F	22	0	0	0	0	0	0	0	0	0	0	0	0	0	0	1
08/09/2020	F	22	0	0	0	0	0	0	0	0	0	0	0	0	0	0	0
23/12/2020	F	22	0	0	0	0	0	0	0	0	0	0	1	0	0	0	0
19/01/2021	F	22	0	0	0	0	0	0	0	0	0	0	0	0	0	0	0
17/05/2021	F	22	0	0	0	0	0	0	0	0	0	0	1	0	0	0	0
07/06/2021	F	22	0	0	0	0	0	0	0	0	0	0	1	1	0	0	0
12.03.2021	F	22	0	0	0	0	0	0	0	0	0	0	0	0	0	1	0
26.03.2021	F	22	0	0	0	0	0	0	0	0	0	0	0	1	0	1	1
27.01.2021	F	22	0	0	0	0	0	0	0	0	0	0	0	0	0	0	1
29.10.2021	F	22	0	0	0	0	0	0	0	0	0	0	0	0	0	1	0
27/06/2022	M	22	0	0	0	0	0	0	0	0	0	0	0	0	0	0	1
22/08/2022	F	22	0	0	0	0	0	0	0	0	0	0	1	1	0	0	0

22/08/2022	F	22	0	0	0	0	0	0	0	0	0	0	1	1	0	0	0
14/10/2022	F	22	0	0	0	0	0	0	0	0	0	0	0	0	0	0	0
10/05/2023	F	22	0	0	0	0	0	0	0	0	0	0	0	0	0	0	1
18/05/2023	F	22	0	0	0	0	0	0	0	0	0	0	0	0	0	0	1
18/09/2023	M	22	0	0	0	0	0	0	0	0	0	0	0	0	0	0	1
08/11/2023	M	22	0	0	0	0	0	0	0	0	0	0	0	0	0	0	1
22/11/2023	F	22	0	0	0	0	0	0	0	0	0	0	0	0	0	0	1
22/05/2024	F	22	0	0	0	0	0	0	0	0	0	0	0	0	1	0	0
02/07/2024	F	22	0	0	0	0	0	0	0	0	0	0	0	0	0	0	1
04.06.2021	F	22	0	0	0	0	0	0	0	0	1	0	0	0	0	0	0
12/10/2022	F	22	0	0	0	0	0	0	0	0	1	0	0	0	0	0	1
14/03/2024	M	22	0	0	0	0	0	0	0	0	0	1	1	0	0	0	0
30/04/2024	F	22	0	0	0	0	0	0	0	0	0	1	1	1	0	0	0
10.02.2021	M	22	0	0	0	0	1	0	0	0	0	0	1	0	0	0	0
19/08/2024	M	22	0	0	1	0	0	0	0	0	0	0	1	1	0	0	0
29/04/2022	F	22	0	0	0	0	0	0	1	0	0	0	1	0	1	0	0
05/10/2022	F	22	0	1	0	0	0	0	0	0	0	0	1	1	0	0	0
06/03/2023	M	22	0	0	0	0	1	0	0	0	0	0	1	0	1	0	0
15/11/2023	F	22	0	0	0	0	1	0	0	0	0	0	1	0	1	0	0
17/01/2024	F	22	0	0	0	0	1	0	0	0	0	0	1	0	0	0	0
15/10/2021	M	22	0	0	0	0	1	0	0	0	0	1	1	0	0	0	0
16/08/2023	F	22	0	0	0	0	1	0	0	1	0	0	1	0	0	0	1
15/11/2024	F	22	0	0	0	0	1	0	0	0	1	0	1	0	1	0	0
11/12/2020	M	22	1	0	0	0	1	0	0	0	0	0	1	0	0	0	0
21/02/2020	F	22	0	1	1	0	0	0	0	0	0	0	1	1	0	0	0
16/09/2020	F	22	0	0	1	1	0	0	0	0	0	0	1	1	0	0	0
18/11/2020	M	22	0	0	1	0	0	1	0	0	0	0	1	0	0	0	0
12/04/2023	M	22	0	0	1	0	1	0	0	0	0	0	1	0	0	0	0
02/07/2024	M	22	0	0	1	0	1	0	0	0	0	0	1	1	0	0	0

02/09/2022	F	22	0	1	0	0	0	1	0	0	0	0	1	1	0	0	0
13/02/2023	M	22	0	0	0	0	1	1	0	0	0	0	1	0	0	0	0
28/05/2020	F	22	0	0	1	1	0	0	0	1	0	0	1	1	1	0	0
16/06/2020	M	22	1	1	0	0	1	0	0	0	0	0	1	1	0	0	0
04/11/2021	F	22	0	1	0	0	0	1	1	0	0	0	1	0	0	0	0
13/10/2023	F	22	0	1	0	0	0	1	1	0	0	0	1	0	0	0	0
22/03/2022	F	22	0	1	1	1	0	0	0	1	0	0	1	1	0	0	0
29.09.2021	F	22	0	1	1	0	0	1	0	0	0	1	1	0	0	0	0
25/09/2020	M	22	0	1	1	1	1	0	0	0	0	0	1	0	1	0	0
16/01/2023	F	22	1	1	1	0	0	1	0	0	0	0	1	1	0	0	0
06/09/2022	F	22	0	1	0	0	1	1	1	0	0	0	1	1	1	0	0
28/05/2024	M	22	1	1	1	1	1	0	0	0	0	0	1	1	0	0	0
03/02/2020	M	23	0	0	0	0	0	0	0	0	0	0	0	0	0	0	0
17/03/2020	F	23	0	0	0	0	0	0	0	0	0	0	1	1	0	0	0
10/08/2020	F	23	0	0	0	0	0	0	0	0	0	0	0	0	0	0	0
10/09/2020	F	23	0	0	0	0	0	0	0	0	0	0	1	1	1	0	0
15/09/2020	F	23	0	0	0	0	0	0	0	0	0	0	0	0	0	0	1
07/01/2021	M	23	0	0	0	0	0	0	0	0	0	0	0	0	1	0	0
23/11/2021	M	23	0	0	0	0	0	0	0	0	0	0	0	0	0	0	0
24.03.2021	M	23	0	0	0	0	0	0	0	0	0	0	1	0	0	0	0
24.03.2021	F	23	0	0	0	0	0	0	0	0	0	0	1	0	0	0	0
28.05.2021	F	23	0	0	0	0	0	0	0	0	0	0	0	0	0	1	0
07/01/2022	F	23	0	0	0	0	0	0	0	0	0	0	0	0	0	0	1
09/02/2022	F	23	0	0	0	0	0	0	0	0	0	0	1	0	0	0	0
23/02/2022	F	23	0	0	0	0	0	0	0	0	0	0	0	0	0	0	1
23/03/2022	F	23	0	0	0	0	0	0	0	0	0	0	1	0	0	0	1
29/06/2022	F	23	0	0	0	0	0	0	0	0	0	0	0	0	0	0	1
12/10/2022	F	23	0	0	0	0	0	0	0	0	0	0	0	0	0	0	1
19/12/2022	F	23	0	0	0	0	0	0	0	0	0	0	0	0	0	0	0

07/04/2023	M	23	0	0	0	0	0	0	0	0	0	0	0	0	0	0	1
18/05/2023	M	23	0	0	0	0	0	0	0	0	0	0	0	0	0	0	1
17/01/2024	F	23	0	0	0	0	0	0	0	0	0	0	0	0	0	0	0
08/03/2024	F	23	0	0	0	0	0	0	0	0	0	0	0	0	0	0	0
29/03/2024	M	23	0	0	0	0	0	0	0	0	0	0	0	0	1	0	0
18/09/2024	F	23	0	0	0	0	0	0	0	0	0	0	0	0	0	0	0
11/10/2024	F	23	0	0	0	0	0	0	0	0	0	0	1	0	1	0	1
19/11/2024	F	23	0	0	0	0	0	0	0	0	0	0	0	0	0	0	0
20/11/2024	F	23	0	0	0	0	0	0	0	0	0	0	0	0	1	0	1
03/12/2024	F	23	0	0	0	0	0	0	0	0	0	0	0	0	0	0	1
18/12/2024	M	23	0	0	0	0	0	0	0	0	0	0	0	0	0	0	1
10.11.2021	M	23	0	0	0	0	0	0	0	0	0	1	0	0	0	0	0
13.10.2021	M	23	0	0	0	0	0	0	0	0	0	1	0	0	0	0	0
17.11.2021	M	23	0	0	0	0	0	0	0	0	0	1	0	1	0	0	0
20/08/2024	M	23	0	0	0	0	0	0	0	0	1	0	1	0	1	0	0
17/02/2020	M	23	0	0	0	0	1	0	0	0	0	0	1	0	0	0	0
08/04/2021	M	23	0	0	0	0	0	0	1	0	0	0	1	0	0	0	0
03/08/2021	M	23	0	0	0	0	1	0	0	0	0	0	1	0	1	0	0
27/09/2021	M	23	0	0	0	0	1	0	0	0	0	0	1	1	0	0	0
10/02/2020	M	23	0	0	1	0	0	0	0	0	0	0	1	1	0	0	0
02/09/2022	F	23	0	0	1	0	0	0	0	0	0	0	1	0	1	0	0
24/10/2022	M	23	0	0	1	0	0	0	0	0	0	0	1	1	0	0	0
15/11/2023	F	23	0	0	1	0	0	0	0	0	0	0	1	0	1	0	1
03/09/2024	F	23	0	0	1	0	0	0	0	0	0	0	1	1	1	0	0
18/03/2022	M	23	0	1	0	0	0	0	0	0	0	0	1	1	0	0	0
29/09/2023	M	23	0	0	0	0	0	0	1	0	0	0	1	0	1	0	0
03/10/2023	F	23	0	1	0	0	0	0	0	0	0	0	1	1	1	0	0
03.09.2021	F	23	0	0	1	0	0	0	0	0	0	1	1	0	0	0	0
17/03/2022	M	23	0	0	0	0	1	0	0	1	0	0	1	0	1	0	0

27.10.2021	F	23	0	1	0	0	0	0	0	1	0	1	0	0	0	0	0
15/10/2020	M	23	1	0	0	0	1	0	0	0	0	0	1	1	1	0	0
02/05/2022	M	23	0	1	1	0	0	0	0	0	0	0	1	1	0	0	0
24/05/2023	F	23	0	1	1	0	0	0	0	0	0	0	1	0	0	0	0
24/07/2023	M	23	0	1	1	0	0	0	0	0	0	0	1	1	0	0	0
08/05/2024	F	23	0	1	1	0	0	0	0	0	0	0	1	0	0	0	0
06/09/2023	M	23	0	0	0	0	1	1	0	0	0	0	1	0	0	0	1
25.08.2021	F	23	0	0	1	0	0	1	0	0	0	1	1	1	1	0	0
02/09/2020	F	23	0	0	1	0	1	1	0	0	0	0	1	0	1	0	0
22/11/2022	M	23	0	0	1	0	1	0	1	0	0	0	1	0	0	0	0
23/10/2024	M	23	0	0	1	0	1	0	1	0	0	0	1	0	0	0	0
10/05/2022	M	23	1	1	0	0	0	1	0	0	0	0	1	1	0	0	0
16/03/2022	F	23	1	0	1	0	1	0	0	1	0	0	1	1	0	0	1
29/10/2020	M	23	1	1	0	0	1	0	1	0	0	0	1	0	1	0	0
20/03/2023	F	23	0	1	1	1	1	0	1	0	0	0	1	1	0	0	0
07/02/2020	F	24	0	0	0	0	0	0	0	0	0	0	0	0	0	0	1
30/06/2020	F	24	0	0	0	0	0	0	0	0	0	0	1	0	1	0	0
03/07/2020	F	24	0	0	0	0	0	0	0	0	0	0	0	0	0	0	1
10/08/2020	M	24	0	0	0	0	0	0	0	0	0	0	1	0	0	0	0
17/05/2021	F	24	0	0	0	0	0	0	0	0	0	0	1	1	0	0	0
08/06/2021	F	24	0	0	0	0	0	0	0	0	0	0	0	0	0	0	1
10/06/2021	M	24	0	0	0	0	0	0	0	0	0	0	1	1	0	0	1
13/10/2021	F	24	0	0	0	0	0	0	0	0	0	0	0	1	0	0	0
18/10/2021	M	24	0	0	0	0	0	0	0	0	0	0	1	1	0	0	0
05.11.2021	F	24	0	0	0	0	0	0	0	0	0	0	0	0	0	1	0
18/03/2022	M	24	0	0	0	0	0	0	0	0	0	0	1	0	0	0	0
31/03/2022	M	24	0	0	0	0	0	0	0	0	0	0	1	0	0	0	0
02/11/2022	F	24	0	0	0	0	0	0	0	0	0	0	1	0	0	0	0
21/11/2022	F	24	0	0	0	0	0	0	0	0	0	0	0	0	0	0	0

11/04/2023	F	24	0	0	0	0	0	0	0	0	0	0	1	0	0	0	0
12/04/2023	F	24	0	0	0	0	0	0	0	0	0	0	1	0	0	0	0
10/08/2023	M	24	0	0	0	0	0	0	0	0	0	0	0	0	0	0	0
10/11/2023	F	24	0	0	0	0	0	0	0	0	0	0	0	0	1	0	0
19/04/2024	F	24	0	0	0	0	0	0	0	0	0	0	0	0	0	0	0
03/09/2024	F	24	0	0	0	0	0	0	0	0	0	0	1	1	0	0	0
17/09/2024	F	24	0	0	0	0	0	0	0	0	0	0	1	0	0	0	0
18/12/2024	M	24	0	0	0	0	0	0	0	0	0	0	0	0	0	0	0
10/09/2020	F	24	0	1	0	0	0	0	0	0	0	0	1	1	1	0	0
21/09/2020	F	24	0	0	0	0	1	0	0	0	0	0	1	0	1	0	0
17.03.2021	F	24	1	0	0	0	0	0	0	0	0	0	1	0	0	0	0
11/02/2022	F	24	0	0	0	0	0	1	0	0	0	0	1	0	1	0	0
02/11/2022	M	24	0	0	0	0	1	0	0	0	0	0	1	0	1	0	0
05/04/2023	F	24	0	0	0	0	1	0	0	0	0	0	1	0	0	0	0
24/04/2023	F	24	0	1	0	0	0	0	0	0	0	0	1	1	1	0	0
14.05.2021	F	24	0	0	0	1	0	0	0	0	0	1	1	0	0	0	0
10.02.2021	M	24	0	0	1	0	0	0	0	1	0	0	1	1	1	0	1
01/08/2024	F	24	0	0	0	0	1	0	0	0	1	0	1	1	1	0	0
16/09/2021	M	24	0	1	1	0	0	0	0	0	0	0	1	1	0	0	0
22/06/2022	M	24	0	1	1	0	0	0	0	0	0	0	1	0	0	0	0
30/04/2024	F	24	0	1	1	0	0	0	0	0	0	0	1	1	0	0	0
25/09/2024	F	24	0	0	1	0	0	1	0	0	0	0	1	0	0	0	0
30/04/2024	F	24	0	1	0	0	0	0	1	0	0	0	1	0	0	0	0
02/07/2024	M	24	0	1	0	0	0	0	1	0	0	0	1	1	0	0	1
28.04.2021	M	24	0	1	0	1	0	0	0	0	0	1	1	0	0	0	0
10/05/2023	M	24	1	1	1	0	0	0	0	0	0	0	1	0	0	0	0
10.02.2021	M	24	0	1	1	0	1	0	0	0	1	0	1	1	0	0	0
08/06/2021	F	24	1	1	1	0	0	0	1	0	0	0	1	1	0	0	0
13/03/2023	M	24	1	1	1	1	0	0	0	0	0	0	1	1	0	0	0

18/09/2023	F	24	0	1	1	0	1	1	0	0	0	0	1	1	1	0	0
21/10/2021	F	24	0	0	1	0	1	1	1	0	1	0	1	1	0	0	0
11/03/2020	M	25	0	0	0	0	0	0	0	0	0	0	0	0	0	0	1
07/07/2020	F	25	0	0	0	0	0	0	0	0	0	0	1	1	0	0	0
14/07/2020	F	25	0	0	0	0	0	0	0	0	0	0	0	0	0	0	0
20/07/2020	F	25	0	0	0	0	0	0	0	0	0	0	0	0	0	0	0
03/09/2020	F	25	0	0	0	0	0	0	0	0	0	0	0	0	0	0	0
25/09/2020	M	25	0	0	0	0	0	0	0	0	0	0	0	0	0	0	0
30/09/2020	F	25	0	0	0	0	0	0	0	0	0	0	1	0	0	0	0
09/11/2020	F	25	0	0	0	0	0	0	0	0	0	0	1	0	1	0	0
18/02/2021	F	25	0	0	0	0	0	0	0	0	0	0	0	0	0	0	0
22/02/2021	F	25	0	0	0	0	0	0	0	0	0	0	0	0	0	0	0
13/05/2021	F	25	0	0	0	0	0	0	0	0	0	0	0	0	0	0	1
10/06/2021	M	25	0	0	0	0	0	0	0	0	0	0	0	0	1	0	0
22/06/2021	M	25	0	0	0	0	0	0	0	0	0	0	1	1	0	0	0
05/07/2021	F	25	0	0	0	0	0	0	0	0	0	0	1	0	0	0	1
15/11/2021	F	25	0	0	0	0	0	0	0	0	0	0	0	0	1	0	0
12.05.2021	F	25	0	0	0	0	0	0	0	0	0	0	0	1	0	0	0
17.03.2021	F	25	0	0	0	0	0	0	0	0	0	0	0	0	1	0	1
25.08.2021	F	25	0	0	0	0	0	0	0	0	0	0	0	0	0	1	0
16/02/2022	F	25	0	0	0	0	0	0	0	0	0	0	2	0	0	0	0
23/02/2022	F	25	0	0	0	0	0	0	0	0	0	0	1	0	0	0	1
08/03/2022	F	25	0	0	0	0	0	0	0	0	0	0	1	1	0	0	0
13/05/2022	F	25	0	0	0	0	0	0	0	0	0	0	0	0	0	0	1
22/06/2022	F	25	0	0	0	0	0	0	0	0	0	0	1	0	0	0	0
07/09/2022	M	25	0	0	0	0	0	0	0	0	0	0	0	0	0	0	0
16/11/2022	M	25	0	0	0	0	0	0	0	0	0	0	1	0	0	0	0
03/02/2023	F	25	0	0	0	0	0	0	0	0	0	0	1	0	0	0	0
01/03/2023	F	25	0	0	0	0	0	0	0	0	0	0	0	0	0	0	1

03/03/2023	F	25	0	0	0	0	0	0	0	0	0	0	0	0	0	0	1
03/03/2023	F	25	0	0	0	0	0	0	0	0	0	0	0	0	0	0	1
29/03/2023	F	25	0	0	0	0	0	0	0	0	0	0	0	0	0	0	1
29/08/2023	F	25	0	0	0	0	0	0	0	0	0	0	1	1	0	0	0
26/09/2023	F	25	0	0	0	0	0	0	0	0	0	0	0	0	0	0	1
27/10/2023	F	25	0	0	0	0	0	0	0	0	0	0	0	0	0	0	1
07/11/2023	F	25	0	0	0	0	0	0	0	0	0	0	0	0	0	0	1
16/11/2023	F	25	0	0	0	0	0	0	0	0	0	0	1	0	0	0	0
02/04/2024	F	25	0	0	0	0	0	0	0	0	0	0	0	0	0	0	0
19/04/2024	M	25	0	0	0	0	0	0	0	0	0	0	0	0	0	0	0
23/04/2024	F	25	0	0	0	0	0	0	0	0	0	0	1	1	0	0	0
18/06/2024	F	25	0	0	0	0	0	0	0	0	0	0	0	0	0	0	0
23/08/2024	F	25	0	0	0	0	0	0	0	0	0	0	0	1	0	0	0
02/10/2024	F	25	0	0	0	0	0	0	0	0	0	0	1	1	0	0	0
30/10/2024	F	25	0	0	0	0	0	0	0	0	0	0	1	0	0	0	0
07/01/2020	F	25	0	0	0	0	0	0	0	0	1	0	1	0	1	0	0
04/10/2021	F	25	0	0	0	0	0	0	0	1	0	0	1	1	0	0	0
28/09/2022	F	25	0	0	0	0	0	0	0	0	1	1	0	0	0	0	0
19/07/2021	F	25	0	0	0	0	1	0	0	0	0	0	1	0	0	0	0
14/09/2021	F	25	0	0	0	0	0	0	1	0	0	0	0	0	0	0	1
13/01/2020	M	25	0	0	1	0	0	0	0	0	0	0	1	1	1	0	0
16/08/2023	M	25	0	0	1	0	0	0	0	0	0	0	1	1	0	0	0
03/09/2024	M	25	0	0	1	0	0	0	0	0	0	0	1	1	0	0	0
21/01/2022	M	25	0	0	0	0	1	0	0	0	0	0	1	0	0	0	0
29/06/2022	M	25	0	0	0	0	1	0	0	0	0	0	1	1	0	0	0
29/06/2022	M	25	0	0	0	0	1	0	0	0	0	0	1	1	0	0	0
22/06/2023	M	25	0	0	0	0	1	0	0	0	0	0	1	0	0	0	1
14/03/2024	F	25	1	0	0	0	0	0	0	0	0	0	1	1	0	0	0
18/12/2024	F	25	0	0	0	0	0	0	1	0	0	0	1	1	0	0	0

26.05.2021	F	25	0	0	0	0	1	0	0	0	0	1	0	1	0	0	0
10/07/2020	M	25	0	1	0	0	1	0	0	0	0	0	1	0	1	0	0
25/11/2020	F	25	0	0	0	0	1	1	0	0	0	0	1	1	0	0	0
13/05/2021	F	25	0	1	0	0	1	0	0	0	0	0	1	1	0	0	0
02/07/2020	M	25	0	1	1	0	0	0	0	0	0	0	1	1	0	0	0
23/09/2020	F	25	0	1	1	0	0	0	0	0	0	0	1	1	0	0	0
02/11/2022	F	25	0	0	0	0	1	1	0	0	0	0	1	0	0	0	0
11/08/2023	F	25	1	1	0	0	0	0	0	0	0	0	1	0	0	0	0
07/10/2021	M	25	0	0	0	0	1	0	1	0	0	1	1	0	0	0	0
29.09.2021	M	25	0	1	1	0	0	0	0	0	0	1	1	0	0	0	0
02/03/2020	M	25	0	1	1	1	0	0	0	0	0	0	1	1	0	0	0
08/12/2020	M	25	0	1	1	0	1	0	0	0	0	0	1	1	0	0	0
18/03/2022	M	25	1	1	1	0	0	0	0	0	0	0	1	1	0	0	0
21/08/2024	M	25	0	1	1	0	1	0	0	0	0	0	1	1	0	0	1
09/07/2024	F	25	1	0	0	1	1	0	0	0	0	0	0	0	0	0	0
26.11.2021	F	25	1	1	0	0	1	0	0	0	0	1	0	1	0	0	0
27/05/2022	F	25	1	1	1	0	1	1	0	0	0	0	1	1	1	0	0
19/02/2020	M	26	0	0	0	0	0	0	0	0	0	0	0	0	1	0	0
26/02/2020	F	26	0	0	0	0	0	0	0	0	0	0	1	0	0	0	0
10/07/2020	M	26	0	0	0	0	0	0	0	0	0	0	1	0	0	0	0
07/08/2020	M	26	0	0	0	0	0	0	0	0	0	0	1	0	0	0	0
02/09/2020	M	26	0	0	0	0	0	0	0	0	0	0	1	1	0	0	0
26/08/2021	F	26	0	0	0	0	0	0	0	0	0	0	1	1	0	0	1
03.11.2021	F	26	0	0	0	0	0	0	0	0	0	0	0	0	0	0	0
13.10.2021	F	26	0	0	0	0	0	0	0	0	0	0	0	0	0	0	0
14/01/2022	M	26	0	0	0	0	0	0	0	0	0	0	0	0	0	0	1
23/03/2022	F	26	0	0	0	0	0	0	0	0	0	0	0	0	0	0	0
25/05/2022	M	26	0	0	0	0	0	0	0	0	0	0	1	1	0	0	0
09/06/2022	F	26	0	0	0	0	0	0	0	0	0	0	0	0	0	0	1

21/06/2022	F	26	0	0	0	0	0	0	0	0	0	0	1	0	0	0	0
26/09/2022	F	26	0	0	0	0	0	0	0	0	0	0	1	1	0	0	0
25/11/2022	M	26	0	0	0	0	0	0	0	0	0	0	1	0	0	0	0
08/02/2023	F	26	0	0	0	0	0	0	0	0	0	0	0	0	0	0	1
29/03/2023	F	26	0	0	0	0	0	0	0	0	0	0	0	0	0	0	1
22/05/2023	F	26	0	0	0	0	0	0	0	0	0	0	0	0	0	0	1
22/05/2023	F	26	0	0	0	0	0	0	0	0	0	0	0	0	0	0	1
26/07/2023	F	26	0	0	0	0	0	0	0	0	0	0	0	0	0	0	1
15/11/2023	M	26	0	0	0	0	0	0	0	0	0	0	0	0	0	0	1
13/03/2024	F	26	0	0	0	0	0	0	0	0	0	0	0	0	0	0	1
02/04/2024	F	26	0	0	0	0	0	0	0	0	0	0	0	0	0	0	0
23/04/2024	F	26	0	0	0	0	0	0	0	0	0	0	1	0	1	0	1
18/06/2024	F	26	0	0	0	0	0	0	0	0	0	0	0	0	0	0	1
18/06/2024	M	26	0	0	0	0	0	0	0	0	0	0	0	0	0	0	0
25/06/2024	F	26	0	0	0	0	0	0	0	0	0	0	0	0	0	0	1
03/07/2024	M	26	0	0	0	0	0	0	0	0	0	0	0	0	0	0	0
16/07/2024	F	26	0	0	0	0	0	0	0	0	0	0	0	0	0	0	0
23/07/2024	F	26	0	0	0	0	0	0	0	0	0	0	0	0	0	0	0
23/10/2024	F	26	0	0	0	0	0	0	0	0	0	0	1	0	1	0	0
13/12/2024	M	26	0	0	0	0	0	0	0	0	0	0	1	1	0	0	0
15/09/2021	F	26	0	0	0	0	0	0	0	0	0	1	1	0	0	0	0
02/12/2021	F	26	0	0	0	0	0	0	0	0	1	0	0	0	0	0	0
17.11.2021	F	26	0	0	0	0	0	0	0	0	0	1	0	0	0	1	0
28/04/2023	M	26	0	0	0	0	0	0	0	0	1	0	0	0	0	0	0
09/09/2020	F	26	0	0	0	1	0	0	0	0	0	0	1	0	1	0	0
14/10/2021	F	26	1	0	0	0	0	0	0	0	0	0	0	0	0	0	1
07/01/2020	M	26	0	0	1	0	0	0	0	0	0	0	1	1	0	0	0
26/10/2020	M	26	0	0	1	0	0	0	0	0	0	0	1	1	0	0	0
17/05/2022	M	26	0	0	1	0	0	0	0	0	0	0	1	1	0	0	0

31/08/2022	M	26	0	0	1	0	0	0	0	0	0	0	1	0	0	0	0
23/10/2024	M	26	0	0	1	0	0	0	0	0	0	0	1	0	0	0	1
21/02/2022	F	26	1	0	0	0	0	0	0	0	0	0	1	1	0	0	1
09/03/2022	M	26	0	0	0	0	1	0	0	0	0	0	1	0	0	0	0
08/04/2022	F	26	0	0	0	0	0	1	0	0	0	0	1	0	0	0	1
10/05/2022	M	26	0	1	0	0	0	0	0	0	0	0	1	1	0	0	0
24/04/2023	M	26	0	1	0	0	0	0	0	0	0	0	1	1	0	0	0
27.08.2021	M	26	0	1	0	0	0	0	0	0	0	1	1	0	0	0	0
12.11.2021	M	26	0	0	1	0	0	0	0	0	0	1	1	0	0	0	0
17/06/2022	M	26	0	0	0	0	1	0	0	1	0	0	1	0	1	0	0
02/08/2023	M	26	0	1	0	0	0	0	0	0	1	0	1	0	1	0	0
23/10/2024	M	26	0	1	0	0	0	0	0	1	0	0	0	0	0	0	0
14/09/2021	F	26	0	0	1	0	0	0	1	0	0	0	1	1	0	0	0
01/02/2022	M	26	0	0	1	1	0	0	0	0	0	0	1	0	0	0	0
15/04/2022	F	26	0	1	1	0	0	0	0	0	0	0	1	1	1	0	0
22/02/2023	F	26	0	0	1	0	0	1	0	0	0	0	1	0	1	0	0
28/06/2023	F	26	0	0	1	0	1	0	0	0	0	0	1	0	0	0	0
03/11/2022	F	26	0	0	0	0	1	1	0	0	0	0	1	1	0	0	1
18/09/2024	M	26	0	1	0	1	0	0	0	0	0	0	1	0	0	0	1
26.05.2021	F	26	1	0	0	0	0	1	0	0	0	1	1	0	0	0	0
09/09/2020	F	26	0	0	1	0	1	1	0	0	0	0	1	0	0	0	0
07/06/2023	M	26	0	1	1	0	0	0	1	0	0	0	1	0	0	0	0
10/09/2021	F	26	0	0	1	0	1	1	1	0	0	1	1	0	0	0	0
27/05/2021	F	26	1	1	1	1	1	0	0	0	0	0	1	1	0	0	1
24.03.2021	F	26	0	1	1	1	0	1	1	0	0	0	1	1	0	0	0
08/01/2020	F	27	0	0	0	0	0	0	0	0	0	0	0	0	0	0	1
08/01/2020	F	27	0	0	0	0	0	0	0	0	0	0	0	0	1	0	1
19/02/2020	F	27	0	0	0	0	0	0	0	0	0	0	0	0	0	0	1
17/03/2020	F	27	0	0	0	0	0	0	0	0	0	0	0	0	1	0	1

06/08/2020	M	27	0	0	0	0	0	0	0	0	0	0	1	0	0	0	0
12/08/2020	F	27	0	0	0	0	0	0	0	0	0	0	0	0	0	0	1
18/09/2020	M	27	0	0	0	0	0	0	0	0	0	0	0	0	0	0	0
25/09/2020	F	27	0	0	0	0	0	0	0	0	0	0	1	0	0	0	0
28/10/2020	F	27	0	0	0	0	0	0	0	0	0	0	0	0	0	0	0
11/01/2021	F	27	0	0	0	0	0	0	0	0	0	0	1	0	0	0	0
08/02/2021	M	27	0	0	0	0	0	0	0	0	0	0	1	0	0	0	0
20/04/2021	F	27	0	0	0	0	0	0	0	0	0	0	0	0	0	0	1
20/04/2021	F	27	0	0	0	0	0	0	0	0	0	0	1	0	0	0	1
20/09/2021	F	27	0	0	0	0	0	0	0	0	0	0	1	0	0	0	0
06/10/2021	F	27	0	0	0	0	0	0	0	0	0	0	0	0	0	1	0
03.02.2021	F	27	0	0	0	0	0	0	0	0	0	0	1	0	0	0	0
25/02/2022	F	27	0	0	0	0	0	0	0	0	0	0	0	0	0	0	0
23/03/2022	F	27	0	0	0	0	0	0	0	0	0	0	0	0	0	0	1
17/05/2022	F	27	0	0	0	0	0	0	0	0	0	0	1	0	0	0	0
25/05/2022	F	27	0	0	0	0	0	0	0	0	0	0	0	0	0	0	1
09/02/2023	F	27	0	0	0	0	0	0	0	0	0	0	0	0	1	0	0
01/03/2023	M	27	0	0	0	0	0	0	0	0	0	0	1	0	0	0	0
07/04/2023	F	27	0	0	0	0	0	0	0	0	0	0	0	0	0	0	1
12/04/2023	F	27	0	0	0	0	0	0	0	0	0	0	1	0	1	0	0
03/06/2023	F	27	0	0	0	0	0	0	0	0	0	0	0	0	0	0	0
20/06/2023	F	27	0	0	0	0	0	0	0	0	0	0	0	0	0	0	1
22/06/2023	F	27	0	0	0	0	0	0	0	0	0	0	0	0	0	0	1
26/06/2023	F	27	0	0	0	0	0	0	0	0	0	0	1	0	0	0	0
15/02/2024	F	27	0	0	0	0	0	0	0	0	0	0	0	0	0	0	1
21/02/2024	F	27	0	0	0	0	0	0	0	0	0	0	0	0	0	0	1
23/02/2024	F	27	0	0	0	0	0	0	0	0	0	0	0	0	0	0	1
06/03/2024	M	27	0	0	0	0	0	0	0	0	0	0	0	0	0	0	1
26/03/2024	F	27	0	0	0	0	0	0	0	0	0	0	0	0	0	0	0

29/03/2024	F	27	0	0	0	0	0	0	0	0	0	0	0	0	0	0	0
11/10/2024	F	27	0	0	0	0	0	0	0	0	0	0	0	0	0	0	0
12/12/2024	M	27	0	0	0	0	0	0	0	0	0	0	0	0	1	0	0
30/01/2020	F	27	0	0	0	0	0	0	0	0	0	1	1	0	1	0	1
15/07/2021	F	27	0	0	0	0	0	0	0	1	0	0	0	0	0	0	1
15.10.2021	M	27	0	0	0	0	0	0	0	0	0	1	0	1	0	0	0
19/04/2023	F	27	0	0	0	0	0	0	0	0	1	0	0	0	0	0	0
27/11/2023	F	27	0	0	0	0	0	0	0	1	0	0	0	0	0	0	1
08/01/2024	M	27	0	0	0	0	0	0	0	1	0	0	0	0	0	0	1
15/03/2024	F	27	0	0	0	0	0	0	0	1	0	0	1	1	0	0	0
05/11/2024	M	27	0	0	0	0	0	0	0	0	0	1	0	0	0	0	1
29/07/2020	F	27	0	0	0	0	0	1	0	0	0	0	1	1	0	0	0
21/09/2020	F	27	0	0	0	0	1	0	0	0	0	0	1	1	0	0	0
24/05/2021	M	27	0	1	0	0	0	0	0	0	0	0	1	1	0	0	0
14/08/2020	F	27	0	0	1	0	0	0	0	0	0	0	1	1	1	0	0
30/09/2020	F	27	0	0	1	0	0	0	0	0	0	0	1	1	0	0	0
16/03/2022	M	27	0	0	1	0	0	0	0	0	0	0	1	1	0	0	0
04/09/2023	M	27	0	0	1	0	0	0	0	0	0	0	1	1	0	0	0
30/10/2024	F	27	0	0	1	0	0	0	0	0	0	0	1	0	0	0	0
13/02/2023	F	27	0	1	0	0	0	0	0	0	0	0	1	1	0	0	0
23/04/2024	M	27	0	1	0	0	0	0	0	0	0	0	1	1	0	0	0
21/05/2024	F	27	0	0	0	1	0	0	0	0	0	0	1	0	1	0	1
14.05.2021	M	27	0	1	0	0	0	0	0	0	0	1	1	0	0	0	0
03.09.2021	F	27	0	0	1	0	0	0	0	0	0	1	1	1	0	0	0
05.11.2021	F	27	0	0	1	0	0	0	0	0	0	1	1	0	0	0	0
15.12.2021	F	27	0	0	1	0	0	0	0	0	0	1	1	0	0	1	1
21/02/2022	M	27	0	0	0	0	1	0	0	0	1	0	1	1	0	0	0
23/03/2021	M	27	0	0	0	0	1	0	0	1	1	0	1	0	1	0	1
02/03/2020	F	27	1	1	0	0	0	1	0	0	0	0	1	1	1	0	0

08/07/2020	M	27	0	1	1	0	0	1	0	0	0	0	1	1	0	0	0
07/10/2020	M	27	1	1	1	0	0	0	0	0	0	0	1	1	1	0	0
10/01/2022	F	27	0	1	1	0	0	1	0	0	0	0	1	1	1	0	0
17/02/2023	F	27	1	1	1	0	0	0	0	0	0	0	1	0	0	0	0
24/11/2023	F	27	1	0	1	0	1	0	0	0	0	0	0	0	0	0	1
23/04/2024	F	27	1	0	1	0	0	1	0	0	0	0	1	1	0	0	0
18/10/2023	F	27	0	1	0	0	1	1	0	0	0	0	1	0	0	0	1
08/10/2021	F	27	0	1	1	0	0	0	1	0	0	1	1	0	0	0	0
24/04/2023	F	27	1	1	1	1	0	0	0	0	0	0	1	1	0	0	0
30/08/2023	F	27	1	1	1	1	0	0	0	0	0	0	1	0	0	0	0
17/02/2023	M	27	1	0	1	0	1	1	0	1	0	0	1	1	0	0	1
04.06.2021	F	27	0	1	1	0	1	1	0	0	0	1	1	0	0	0	0
18/02/2022	F	27	1	1	0	0	1	0	1	1	0	0	1	1	1	0	0
27/05/2021	F	27	1	1	1	0	1	1	0	0	0	0	1	1	1	0	0
19.05.2021	M	27	0	1	1	1	1	0	1	0	0	1	1	0	0	0	0
08/01/2020	F	28	0	0	0	0	0	0	0	0	0	0	0	0	0	0	0
14/02/2020	F	28	0	0	0	0	0	0	0	0	0	0	0	0	0	0	0
26/02/2020	F	28	0	0	0	0	0	0	0	0	0	0	1	0	1	0	0
01/07/2020	F	28	0	0	0	0	0	0	0	0	0	0	0	0	0	0	1
03/07/2020	F	28	0	0	0	0	0	0	0	0	0	0	0	0	0	0	1
29/07/2020	M	28	0	0	0	0	0	0	0	0	0	0	0	0	1	0	0
16/09/2020	M	28	0	0	0	0	0	0	0	0	0	0	1	1	0	0	0
23/09/2020	F	28	0	0	0	0	0	0	0	0	0	0	1	0	0	0	0
05/10/2020	F	28	0	0	0	0	0	0	0	0	0	0	1	0	0	0	0
13/11/2020	M	28	0	0	0	0	0	0	0	0	0	0	1	0	1	0	0
17/12/2020	M	28	0	0	0	0	0	0	0	0	0	0	1	0	0	0	0
22/02/2021	F	28	0	0	0	0	0	0	0	0	0	0	0	0	0	0	0
12/04/2021	M	28	0	0	0	0	0	0	0	0	0	0	1	0	0	0	0
29/06/2021	M	28	0	0	0	0	0	0	0	0	0	0	1	0	0	0	0

04/11/2021	M	28	0	0	0	0	0	0	0	0	0	0	0	0	0	0	1
15.10.2021	F	28	0	0	0	0	0	0	0	0	0	0	0	0	0	1	0
11/02/2022	M	28	0	0	0	0	0	0	0	0	0	0	1	0	0	0	1
13/04/2022	F	28	0	0	0	0	0	0	0	0	0	0	0	0	0	0	1
20/05/2022	F	28	0	0	0	0	0	0	0	0	0	0	0	0	0	0	1
25/05/2022	F	28	0	0	0	0	0	0	0	0	0	0	0	0	0	0	1
12/10/2022	M	28	0	0	0	0	0	0	0	0	0	0	0	0	0	0	1
12/10/2022	M	28	0	0	0	0	0	0	0	0	0	0	0	0	0	0	1
14/10/2022	F	28	0	0	0	0	0	0	0	0	0	0	0	0	0	0	1
06/02/2023	F	28	0	0	0	0	0	0	0	0	0	0	1	0	0	0	1
22/02/2023	F	28	0	0	0	0	0	0	0	0	0	0	1	0	0	0	0
05/04/2023	F	28	0	0	0	0	0	0	0	0	0	0	1	0	1	0	0
31/05/2023	M	28	0	0	0	0	0	0	0	0	0	0	0	0	0	0	1
06/06/2023	M	28	0	0	0	0	0	0	0	0	0	0	0	0	0	0	1
18/08/2023	M	28	0	0	0	0	0	0	0	0	0	0	1	0	0	0	0
08/11/2023	F	28	0	0	0	0	0	0	0	0	0	0	1	0	0	0	0
05/01/2024	F	28	0	0	0	0	0	0	0	0	0	0	1	0	0	0	1
08/01/2024	M	28	0	0	0	0	0	0	0	0	0	0	1	0	0	0	0
17/01/2024	M	28	0	0	0	0	0	0	0	0	0	0	1	0	0	0	0
23/01/2024	F	28	0	0	0	0	0	0	0	0	0	0	0	0	0	0	1
23/02/2024	M	28	0	0	0	0	0	0	0	0	0	0	0	0	0	0	0
02/04/2024	F	28	0	0	0	0	0	0	0	0	0	0	0	0	0	0	0
24/04/2024	F	28	0	0	0	0	0	0	0	0	0	0	0	0	0	0	0
20/05/2024	F	28	0	0	0	0	0	0	0	0	0	0	0	0	0	0	0
06/06/2024	F	28	0	0	0	0	0	0	0	0	0	0	0	0	0	0	1
27/08/2024	F	28	0	0	0	0	0	0	0	0	0	0	0	0	0	0	1
28/08/2024	F	28	0	0	0	0	0	0	0	0	0	0	0	0	0	0	0
06/09/2024	F	28	0	0	0	0	0	0	0	0	0	0	1	1	0	0	0
29/07/2020	M	28	0	0	0	0	0	0	0	0	1	0	0	0	0	0	1

19.11.2021	M	28	0	0	0	0	0	0	0	0	0	1	0	0	0	0	0
28.04.2021	M	28	0	0	0	0	0	0	0	0	0	1	0	0	0	0	0
29/03/2023	F	28	0	0	0	0	0	0	0	1	0	0	0	0	0	0	1
22/01/2020	M	28	0	0	1	0	0	0	0	0	0	0	1	1	1	0	1
16/03/2022	M	28	0	0	1	0	0	0	0	0	0	0	1	1	0	0	0
23/08/2022	F	28	0	0	1	0	0	0	0	0	0	0	1	1	1	0	0
21/09/2022	F	28	0	0	1	0	0	0	0	0	0	0	1	1	0	0	0
07/12/2022	F	28	0	0	1	0	0	0	0	0	0	0	1	0	0	0	0
22/06/2023	M	28	0	0	1	0	0	0	0	0	0	0	1	0	0	0	1
25/08/2023	F	28	0	0	1	0	0	0	0	0	0	0	1	0	0	0	0
06/09/2023	M	28	0	0	1	0	0	0	0	0	0	0	1	0	1	1	0
27/01/2022	M	28	0	0	0	0	1	0	0	0	0	0	0	0	1	0	0
22/02/2022	F	28	0	0	0	0	1	0	0	0	0	0	1	0	0	0	0
25/11/2022	F	28	0	0	0	0	1	0	0	0	0	0	1	0	1	0	0
16/12/2022	M	28	0	1	0	0	0	0	0	0	0	0	1	1	1	0	0
24/05/2023	M	28	0	0	0	0	1	0	0	0	0	0	1	0	0	0	0
31/01/2024	F	28	0	0	0	0	0	1	0	0	0	0	1	1	1	0	0
22/03/2024	F	28	1	0	0	0	0	0	0	0	0	0	1	0	0	0	0
23/07/2024	M	28	0	1	0	0	0	0	0	0	0	0	1	0	0	0	0
10.09.2021	F	28	0	0	1	0	0	0	0	0	0	1	1	0	0	0	0
12.05.2021	F	28	0	0	1	0	0	0	0	0	0	1	1	1	0	0	0
13/07/2020	M	28	0	1	0	0	1	0	0	0	0	0	1	0	1	0	0
14/07/2020	M	28	0	1	0	0	1	0	0	0	0	0	1	1	0	0	0
09.03.2021	F	28	0	0	0	0	0	1	1	0	0	0	1	0	0	0	0
06/05/2021	M	28	0	0	1	0	1	0	0	0	0	0	1	1	0	0	0
23/04/2024	M	28	0	1	1	0	0	0	0	0	0	0	1	0	0	0	0
05/11/2024	F	28	0	0	0	0	1	0	1	0	0	0	1	0	0	0	0
22/09/2021	F	28	1	1	0	0	0	0	0	0	0	1	1	0	0	0	0
12.05.2021	M	28	0	0	0	0	1	0	1	0	0	1	0	1	0	1	1

30/03/2022	F	28	1	1	1	0	0	0	0	0	0	0	1	1	0	0	0
09/06/2022	M	28	0	0	1	0	1	1	0	0	0	0	1	1	0	0	0
21/09/2023	F	28	0	0	1	0	1	1	0	0	0	0	1	1	0	0	0
04/10/2023	M	28	0	1	1	0	0	1	0	0	0	0	1	0	0	0	1
25/10/2023	M	28	1	1	1	0	0	0	0	0	0	0	1	0	1	0	0
04/12/2024	F	28	1	0	1	0	1	0	0	0	0	0	1	1	0	0	1
31/08/2020	F	28	0	1	1	1	1	1	0	0	0	0	1	1	0	0	0
18/03/2022	F	28	0	1	1	1	1	1	0	0	0	0	1	1	0	0	0
20/06/2023	M	28	1	1	1	0	1	0	1	0	0	0	1	1	0	0	0
02/11/2022	F	28	1	1	1	0	1	1	1	0	0	0	1	0	0	0	0
02/11/2022	F	28	1	1	1	0	1	1	1	0	0	0	1	0	0	0	0
08/01/2020	F	29	0	0	0	0	0	0	0	0	0	0	0	0	0	0	1
13/01/2020	M	29	0	0	0	0	0	0	0	0	0	0	0	0	0	0	0
30/01/2020	F	29	0	0	0	0	0	0	0	0	0	0	0	0	0	0	1
18/02/2020	F	29	0	0	0	0	0	0	0	0	0	0	0	0	0	0	1
26/06/2020	M	29	0	0	0	0	0	0	0	0	0	0	1	0	0	0	0
17/09/2020	M	29	0	0	0	0	0	0	0	0	0	0	1	1	0	0	0
21/09/2020	F	29	0	0	0	0	0	0	0	0	0	0	1	1	0	0	0
13/04/2021	F	29	0	0	0	0	0	0	0	0	0	0	1	0	0	0	1
06/07/2021	M	29	0	0	0	0	0	0	0	0	0	0	0	0	0	0	1
30/08/2021	F	29	0	0	0	0	0	0	0	0	0	0	1	0	0	0	0
06/09/2021	F	29	0	0	0	0	0	0	0	0	0	0	0	0	0	0	1
22/11/2021	F	29	0	0	0	0	0	0	0	0	0	0	1	0	1	0	0
02/12/2021	M	29	0	0	0	0	0	0	0	0	0	0	0	0	0	0	1
25/05/2024	M	29	0	0	0	0	0	0	0	0	0	0	1	1	0	0	0
05/01/2022	F	29	0	0	0	0	0	0	0	0	0	0	0	0	0	0	1
19/01/2022	M	29	0	0	0	0	0	0	0	0	0	0	0	0	0	0	1
12/04/2022	M	29	0	0	0	0	0	0	0	0	0	0	0	0	1	0	0
12/04/2022	M	29	0	0	0	0	0	0	0	0	0	0	1	0	1	0	0

16/05/2022	M	29	0	0	0	0	0	0	0	0	0	0	1	0	0	0	0
04/10/2022	F	29	0	0	0	0	0	0	0	0	0	0	1	0	1	0	0
07/12/2022	F	29	0	0	0	0	0	0	0	0	0	0	0	0	1	0	0
14/12/2022	F	29	0	0	0	0	0	0	0	0	0	0	1	0	0	0	1
15/12/2022	F	29	0	0	0	0	0	0	0	0	0	0	0	0	0	0	1
16/12/2022	F	29	0	0	0	0	0	0	0	0	0	0	0	0	0	0	0
01/03/2023	F	29	0	0	0	0	0	0	0	0	0	0	1	0	0	0	0
21/06/2023	F	29	0	0	0	0	0	0	0	0	0	0	1	0	0	0	1
26/06/2023	F	29	0	0	0	0	0	0	0	0	0	0	1	0	0	0	0
11/10/2023	F	29	0	0	0	0	0	0	0	0	0	0	0	0	0	0	1
15/11/2023	F	29	0	0	0	0	0	0	0	0	0	0	0	0	0	0	1
05/01/2024	F	29	0	0	0	0	0	0	0	0	0	0	1	0	0	0	0
24/02/2024	F	29	0	0	0	0	0	0	0	0	0	0	0	0	0	0	1
23/04/2024	F	29	0	0	0	0	0	0	0	0	0	0	0	0	0	0	1
22/05/2024	M	29	0	0	0	0	0	0	0	0	0	0	0	0	0	0	1
24/05/2024	F	29	0	0	0	0	0	0	0	0	0	0	0	0	0	0	0
19/09/2024	F	29	0	0	0	0	0	0	0	0	0	0	1	1	0	0	0
09/10/2024	M	29	0	0	0	0	0	0	0	0	0	0	1	0	0	0	0
30/10/2024	M	29	0	0	0	0	0	0	0	0	0	0	1	0	0	0	0
03/12/2024	F	29	0	0	0	0	0	0	0	0	0	0	0	0	0	0	0
21/09/2021	M	29	0	0	0	0	0	0	0	0	0	1	0	0	0	0	0
12.11.2021	F	29	0	0	0	0	0	0	0	0	0	1	0	1	0	0	0
11/03/2020	F	29	0	0	0	0	1	0	0	0	0	0	1	0	0	0	0
12.02.2021	F	29	1	0	0	0	0	0	0	0	0	0	1	0	0	0	0
06/09/2021	F	29	0	0	1	0	0	0	0	0	0	0	1	1	0	0	0
11/11/2021	M	29	0	0	1	0	0	0	0	0	0	0	1	1	0	0	0
16/11/2022	M	29	0	0	1	0	0	0	0	0	0	0	1	0	1	0	0
16/11/2022	M	29	0	0	1	0	0	0	0	0	0	0	1	0	0	0	0
24/05/2023	F	29	0	0	1	0	0	0	0	0	0	0	1	0	0	0	0

09/05/2024	M	29	0	0	1	0	0	0	0	0	0	0	1	1	0	0	0
01/08/2024	F	29	0	0	1	0	0	0	0	0	0	0	1	1	1	0	0
17/09/2024	M	29	0	0	1	0	0	0	0	0	0	0	1	1	1	0	0
10/06/2022	M	29	0	1	0	0	0	0	0	0	0	0	1	0	0	0	0
18/10/2024	M	29	0	0	0	0	1	0	0	0	0	0	1	1	1	0	0
25/11/2024	F	29	0	0	0	0	1	0	0	0	0	0	1	1	0	0	0
05/10/2021	F	29	0	0	0	0	1	0	0	0	1	0	1	0	1	0	0
07.05.2021	F	29	0	1	0	0	0	0	0	0	0	1	1	0	0	0	0
19/02/2020	M	29	0	0	0	1	0	0	1	0	0	0	1	0	1	1	0
07/10/2021	M	29	1	1	0	0	0	0	0	0	0	0	1	1	0	0	0
08/03/2020	M	29	0	0	1	0	0	0	1	0	0	0	1	1	0	0	0
17/03/2020	M	29	0	0	1	1	0	0	0	0	0	0	1	1	0	0	0
09/02/2022	M	29	0	0	1	0	1	0	0	0	0	0	1	1	0	0	0
28/04/2022	M	29	0	0	1	0	1	0	0	0	0	0	1	0	0	0	0
26/05/2022	M	29	1	0	1	0	0	0	0	0	0	0	1	1	0	0	0
17/02/2023	F	29	0	1	1	0	0	0	0	0	0	0	1	0	0	0	0
02/08/2023	M	29	0	1	1	0	0	0	0	0	0	0	1	1	0	0	0
25/08/2023	M	29	0	1	1	0	0	0	0	0	0	0	1	0	0	0	0
10/07/2024	F	29	0	1	1	0	0	0	0	0	0	0	1	1	1	0	0
03/09/2024	M	29	0	1	1	0	0	0	0	0	0	0	1	1	0	0	0
13/11/2020	F	29	0	0	0	0	1	0	1	0	0	1	1	1	1	0	0
01.09.2021	M	29	0	1	1	0	0	0	0	0	0	1	1	1	0	0	0
04.06.2021	M	29	1	0	1	0	0	0	0	0	0	1	1	0	0	0	0
10/05/2023	F	29	0	1	1	0	1	0	0	0	0	0	1	0	0	0	0
16/05/2023	M	29	0	1	1	0	0	1	0	0	0	0	1	1	1	0	1
09/09/2022	F	29	0	1	0	0	0	1	1	0	0	0	1	1	0	0	0
08/05/2024	M	29	1	1	0	0	0	0	1	0	0	0	1	1	0	0	0
26.05.2021	M	29	1	1	0	1	0	0	0	0	0	1	1	0	0	0	0
28.05.2021	F	29	1	1	0	1	0	0	0	0	0	1	1	0	0	0	0

21/03/2023	F	29	0	1	0	0	1	0	1	0	1	0	1	1	0	0	0
29.01.2021	M	29	0	1	0	1	1	1	0	0	0	0	1	1	0	0	0
27/11/2020	F	29	1	1	1	0	0	1	0	0	0	0	1	0	0	0	0
05.11.2021	M	29	1	1	1	0	0	1	0	0	0	1	1	1	0	0	0
08/01/2020	M	30	0	0	0	0	0	0	0	0	0	0	1	0	0	0	1
29/01/2020	F	30	0	0	0	0	0	0	0	0	0	0	0	0	0	0	1
05/02/2020	M	30	0	0	0	0	0	0	0	0	0	0	0	0	1	0	0
25/02/2020	F	30	0	0	0	0	0	0	0	0	0	0	0	0	0	0	0
26/02/2020	F	30	0	0	0	0	0	0	0	0	0	0	0	0	0	0	1
15/03/2020	M	30	0	0	0	0	0	0	0	0	0	0	1	1	0	0	0
15/06/2020	F	30	0	0	0	0	0	0	0	0	0	0	1	1	1	0	0
22/06/2020	M	30	0	0	0	0	0	0	0	0	0	0	1	0	0	0	0
22/06/2020	F	30	0	0	0	0	0	0	0	0	0	0	0	0	0	0	0
17/07/2020	F	30	0	0	0	0	0	0	0	0	0	0	0	0	0	0	0
12/08/2020	M	30	0	0	0	0	0	0	0	0	0	0	1	1	1	0	0
27/10/2020	M	30	0	0	0	0	0	0	0	0	0	0	1	0	0	0	0
10/11/2020	M	30	0	0	0	0	0	0	0	0	0	0	1	0	1	0	0
01/02/2021	M	30	0	0	0	0	0	0	0	0	0	0	1	0	0	0	0
10/06/2021	M	30	0	0	0	0	0	0	0	0	0	0	1	1	0	0	0
20/07/2021	M	30	0	0	0	0	0	0	0	0	0	0	0	0	0	0	0
26/08/2021	F	30	0	0	0	0	0	0	0	0	0	0	0	0	0	0	1
06/09/2021	M	30	0	0	0	0	0	0	0	0	0	0	0	0	0	0	1
28.05.2021	F	30	0	0	0	0	0	0	0	0	0	0	0	0	0	1	0
02/02/2022	F	30	0	0	0	0	0	0	0	0	0	0	0	0	0	0	1
02/02/2022	F	30	0	0	0	0	0	0	0	0	0	0	1	0	1	0	0
09/02/2022	F	30	0	0	0	0	0	0	0	0	0	0	0	0	0	0	1
24/02/2022	M	30	0	0	0	0	0	0	0	0	0	0	1	1	0	0	0
08/03/2022	F	30	0	0	0	0	0	0	0	0	0	0	0	0	0	0	1
09/03/2022	M	30	0	0	0	0	0	0	0	0	0	0	0	0	0	0	0

21/04/2022	M	30	0	0	0	0	0	0	0	0	0	0	0	0	0	0	1
19/09/2022	F	30	0	0	0	0	0	0	0	0	0	0	1	0	0	0	0
26/10/2022	F	30	0	0	0	0	0	0	0	0	0	0	0	0	0	0	0
04/11/2022	F	30	0	0	0	0	0	0	0	0	0	0	0	0	0	0	1
24/11/2022	M	30	0	0	0	0	0	0	0	0	0	0	1	0	0	0	0
29/11/2022	F	30	0	0	0	0	0	0	0	0	0	0	1	0	0	0	0
01/03/2023	F	30	0	0	0	0	0	0	0	0	0	0	0	0	0	0	1
21/06/2023	M	30	0	0	0	0	0	0	0	0	0	0	1	0	0	0	1
28/06/2023	M	30	0	0	0	0	0	0	0	0	0	0	0	0	0	0	1
28/06/2023	M	30	0	0	0	0	0	0	0	0	0	0	1	0	0	0	0
16/11/2023	F	30	0	0	0	0	0	0	0	0	0	0	1	0	0	0	0
19/02/2024	M	30	0	0	0	0	0	0	0	0	0	0	1	0	1	1	0
01/03/2024	M	30	0	0	0	0	0	0	0	0	0	0	0	0	0	0	0
06/03/2024	M	30	0	0	0	0	0	0	0	0	0	0	0	0	1	0	0
29/03/2024	F	30	0	0	0	0	0	0	0	0	0	0	0	0	0	0	0
02/04/2024	F	30	0	0	0	0	0	0	0	0	0	0	0	0	0	0	0
23/04/2024	M	30	0	0	0	0	0	0	0	0	0	0	0	0	0	0	0
21/08/2024	F	30	0	0	0	0	0	0	0	0	0	0	0	0	0	0	1
28/08/2024	F	30	0	0	0	0	0	0	0	0	0	0	0	0	0	0	0
03/09/2024	F	30	0	0	0	0	0	0	0	0	0	0	1	1	0	0	0
13/12/2024	M	30	0	0	0	0	0	0	0	0	0	0	0	0	0	0	1
30/09/2020	M	30	0	0	0	0	0	0	0	0	0	1	1	0	0	0	0
05/10/2020	F	30	0	0	0	0	0	0	0	0	1	0	1	0	1	0	0
06.10.2021	F	30	0	0	0	0	0	0	0	0	1	0	0	0	0	0	0
19.11.2021	M	30	0	0	0	0	0	0	0	0	0	1	0	1	0	0	0
05/04/2023	F	30	0	0	0	0	0	0	0	1	0	0	0	0	0	0	1
16/11/2023	M	30	0	0	0	0	0	0	0	0	1	0	0	0	0	0	1
04/03/2024	M	30	0	0	0	0	0	0	0	0	1	0	0	0	0	0	1
05/07/2024	F	30	0	0	0	0	0	0	0	0	1	0	0	0	0	0	0

26/02/2020	F	30	0	0	0	0	1	0	0	0	0	0	1	0	1	0	0
26/05/2020	F	30	0	0	0	0	1	0	0	0	0	0	1	0	1	0	0
08/04/2021	M	30	0	1	0	0	0	0	0	0	0	0	1	1	0	0	1
14/06/2021	M	30	0	1	0	0	0	0	0	0	0	0	1	1	0	0	0
07/09/2020	F	30	0	0	1	0	0	0	0	0	0	0	1	1	0	0	0
09/09/2020	M	30	0	0	1	0	0	0	0	0	0	0	0	0	0	0	0
25/08/2022	M	30	0	0	1	0	0	0	0	0	0	0	1	1	1	0	0
27/09/2023	M	30	0	0	1	0	0	0	0	0	0	0	1	0	0	0	0
15/11/2023	F	30	0	0	1	0	0	0	0	0	0	0	1	0	1	0	1
27/09/2024	M	30	0	0	1	0	0	0	0	0	0	0	1	1	0	0	0
19/11/2024	F	30	0	0	1	0	0	0	0	0	0	0	1	1	0	0	0
29/09/2022	F	30	0	0	0	1	0	0	0	0	0	0	1	1	0	0	0
08.09.2021	F	30	0	0	0	0	0	1	0	0	0	1	1	1	0	0	0
26.05.2021	M	30	0	1	0	0	0	0	0	0	0	1	1	0	0	0	0
27/01/2020	F	30	0	0	0	0	1	1	0	0	0	0	1	0	0	0	0
29/09/2020	M	30	0	1	0	0	1	0	0	0	0	0	1	0	1	0	0
20/01/2020	M	30	0	0	1	1	0	0	0	0	0	0	1	1	0	0	0
28/02/2020	M	30	0	0	1	0	1	0	0	0	0	0	1	1	0	0	0
01/11/2023	M	30	0	1	1	0	0	0	0	0	0	0	1	0	1	0	0
31.03.2021	F	30	0	0	1	0	1	0	0	0	0	0	1	1	0	0	0
21/09/2022	F	30	0	1	0	0	1	0	0	0	0	0	1	1	1	0	1
18/03/2022	F	30	0	0	1	0	1	0	0	0	1	0	1	1	1	0	1
11/08/2020	F	30	0	1	1	0	1	0	0	0	0	0	1	1	1	0	0
11/05/2022	M	30	0	0	1	0	1	1	0	0	0	0	1	1	1	0	0
20/05/2022	F	30	0	1	1	0	0	1	0	0	0	0	1	1	0	0	0
22/05/2023	M	30	0	0	1	1	1	0	0	0	0	0	1	1	0	0	0
31/01/2024	F	30	0	1	1	1	0	0	0	0	0	0	1	0	1	0	0
31/07/2024	M	30	0	1	1	0	1	0	0	0	0	0	1	0	1	0	0
18/09/2024	M	30	0	1	1	1	0	0	0	0	0	0	1	0	1	0	0

09/10/2024	F	30	0	1	1	0	1	0	0	0	0	0	1	1	0	0	0
13/11/2024	M	30	0	0	1	0	0	1	1	0	0	0	1	1	0	0	0
13/04/2022	M	30	1	1	0	0	1	0	0	0	0	0	1	1	0	0	0
22/08/2024	F	30	0	1	0	0	1	1	0	0	1	0	0	1	1	2	0
03/08/2020	M	30	1	1	1	0	0	1	0	0	0	0	1	1	1	0	0
20/09/2022	M	30	1	1	1	1	0	0	0	0	0	0	1	1	0	0	0
19/04/2024	M	30	0	0	1	0	1	1	1	0	0	0	1	0	1	0	0
29/08/2024	F	30	0	1	1	1	1	0	0	0	0	0	1	0	0	0	0
04/04/2022	F	30	1	1	0	0	1	1	0	0	0	0	1	1	0	0	0
21/12/2020	M	30	1	1	1	0	1	1	0	0	0	0	1	1	0	0	0
22/01/2020	F	31	0	0	0	0	0	0	0	0	0	0	1	0	0	0	0
10/02/2020	M	31	0	0	0	0	0	0	0	0	0	0	0	0	0	0	0
21/02/2020	F	31	0	0	0	0	0	0	0	0	0	0	1	0	0	0	1
02/03/2020	M	31	0	0	0	0	0	0	0	0	0	0	0	0	0	0	1
02/09/2020	M	31	0	0	0	0	0	0	0	0	0	0	1	0	1	0	0
02/09/2020	F	31	0	0	0	0	0	0	0	0	0	0	1	0	1	0	1
24/09/2020	F	31	0	0	0	0	0	0	0	0	0	0	1	0	0	0	0
11/11/2020	F	31	0	0	0	0	0	0	0	0	0	0	1	0	0	0	0
19/01/2021	M	31	0	0	0	0	0	0	0	0	0	0	0	0	0	0	1
25/01/2021	F	31	0	0	0	0	0	0	0	0	0	0	0	0	0	0	0
11/05/2021	F	31	0	0	0	0	0	0	0	0	0	0	0	0	0	0	1
21/09/2021	M	31	0	0	0	0	0	0	0	0	0	0	1	0	0	0	0
24.03.2021	F	31	0	0	0	0	0	0	0	0	0	0	1	0	0	0	0
26.05.2021	F	31	0	0	0	0	0	0	0	0	0	0	0	0	0	0	1
27.10.2021	F	31	0	0	0	0	0	0	0	0	0	0	0	0	0	0	0
06/01/2022	M	31	0	0	0	0	0	0	0	0	0	0	0	0	0	0	0
10/01/2022	M	31	0	0	0	0	0	0	0	0	0	0	0	0	0	0	0
12/01/2022	F	31	0	0	0	0	0	0	0	0	0	0	1	0	1	0	0
13/01/2022	F	31	0	0	0	0	0	0	0	0	0	0	0	0	0	0	0

24/05/2022	F	31	0	0	0	0	0	0	0	0	0	0	0	0	0	0	1
27/09/2022	F	31	0	0	0	0	0	0	0	0	0	0	1	1	0	0	0
14/10/2022	F	31	0	0	0	0	0	0	0	0	0	0	0	0	0	0	1
21/11/2022	F	31	0	0	0	0	0	0	0	0	0	0	0	0	0	0	1
11/01/2023	F	31	0	0	0	0	0	0	0	0	0	0	0	0	0	0	1
09/02/2023	F	31	0	0	0	0	0	0	0	0	0	0	0	0	1	0	0
01/03/2023	M	31	0	0	0	0	0	0	0	0	0	0	0	0	0	0	1
22/03/2023	F	31	0	0	0	0	0	0	0	0	0	0	1	0	0	0	0
29/03/2023	M	31	0	0	0	0	0	0	0	0	0	0	1	0	0	0	0
29/05/2023	F	31	0	0	0	0	0	0	0	0	0	0	1	0	0	0	0
27/06/2023	M	31	0	0	0	0	0	0	0	0	0	0	0	0	0	0	1
29/06/2023	F	31	0	0	0	0	0	0	0	0	0	0	0	0	0	0	1
04/08/2023	M	31	0	0	0	0	0	0	0	0	0	0	0	0	0	0	1
09/08/2023	F	31	0	0	0	0	0	0	0	0	0	0	0	0	0	0	1
14/02/2024	F	31	0	0	0	0	0	0	0	0	0	0	0	0	0	0	0
06/03/2024	F	31	0	0	0	0	0	0	0	0	0	0	1	1	0	0	0
22/03/2024	M	31	0	0	0	0	0	0	0	0	0	0	0	0	0	0	0
29/08/2024	F	31	0	0	0	0	0	0	0	0	0	0	1	1	0	0	0
04/09/2024	F	31	0	0	0	0	0	0	0	0	0	0	0	0	0	0	1
22/10/2024	M	31	0	0	0	0	0	0	0	0	0	0	0	0	0	0	1
29.10.2021	F	31	0	0	0	0	0	0	0	0	0	1	1	1	0	0	0
27/09/2024	M	31	0	0	0	0	0	0	0	0	0	1	1	0	0	0	0
14/09/2020	F	31	0	0	0	0	0	0	1	0	0	0	0	0	1	0	0
16/11/2020	M	31	0	0	0	0	0	0	1	0	0	0	1	0	1	0	0
07/01/2020	F	31	0	0	1	0	0	0	0	0	0	0	1	1	0	0	0
26/02/2020	M	31	0	0	1	0	0	0	0	0	0	0	1	1	0	0	0
27/07/2020	M	31	0	0	1	0	0	0	0	0	0	0	1	0	0	0	0
16/09/2020	F	31	0	0	1	0	0	0	0	0	0	0	1	0	0	0	0
05/07/2022	M	31	0	0	1	0	0	0	0	0	0	0	1	0	0	0	0

16/07/2022	M	31	0	0	1	0	0	0	0	0	0	0	1	0	1	0	0
01/08/2022	F	31	0	0	1	0	0	0	0	0	0	0	1	1	0	0	1
12/09/2022	M	31	0	0	1	0	0	0	0	0	0	0	1	1	1	0	0
27/01/2023	F	31	0	0	1	0	0	0	0	0	0	0	1	0	0	0	0
04/09/2024	F	31	0	0	1	0	0	0	0	0	0	0	1	1	0	0	0
04/09/2024	M	31	0	0	1	0	0	0	0	0	0	0	1	1	0	0	0
02/09/2022	F	31	0	0	0	0	0	1	0	0	0	0	1	1	0	0	0
27/02/2023	M	31	0	0	0	0	1	0	0	0	0	0	1	0	0	0	1
23/05/2023	M	31	0	0	0	0	1	0	0	0	0	0	1	1	0	0	0
23/08/2023	F	31	0	0	0	0	0	1	0	0	0	0	1	0	0	0	0
07.05.2021	M	31	0	1	0	0	0	0	0	0	0	1	1	0	0	0	0
15.12.2021	M	31	0	0	0	0	1	0	0	0	0	1	0	1	0	0	0
22.10.2021	F	31	0	0	1	0	0	0	0	0	0	1	1	0	0	0	0
25.08.2021	F	31	0	0	1	0	0	0	0	0	0	1	1	0	0	0	0
09/12/2020	M	31	0	0	0	0	1	0	1	0	0	0	1	0	1	0	0
01/11/2021	F	31	0	0	0	0	0	1	1	0	0	0	1	0	1	0	0
06/01/2020	M	31	0	1	1	0	0	0	0	0	0	0	1	0	0	0	0
27/02/2020	F	31	0	1	1	0	0	0	0	0	0	0	1	1	1	0	0
11/03/2020	M	31	0	0	1	0	0	1	0	0	0	0	1	1	0	0	0
22/09/2020	M	31	0	0	1	1	0	0	0	0	0	0	1	1	0	0	0
22/10/2021	M	31	0	0	1	0	0	1	0	0	0	0	1	1	1	0	0
29/03/2023	M	31	1	0	1	0	0	0	0	0	0	0	1	0	0	0	0
13/06/2023	M	31	0	0	1	0	1	0	0	0	0	0	1	1	0	0	0
29/08/2023	F	31	0	0	1	0	1	0	0	0	0	0	1	1	1	0	0
01/07/2024	M	31	0	0	1	1	0	0	0	0	0	0	1	0	0	0	0
28/06/2023	M	31	0	1	0	0	0	1	0	0	0	0	1	0	0	0	0
24/05/2024	M	31	0	0	0	1	1	0	0	0	0	0	1	1	0	0	0
08.09.2021	F	31	0	0	1	0	0	1	0	0	0	1	1	0	0	0	0
22/09/2021	M	31	0	1	0	0	1	1	0	0	0	0	0	1	0	0	0

31/08/2020	F	31	0	1	1	0	0	0	1	0	0	0	1	1	0	0	0
15/02/2023	F	31	0	1	1	0	0	1	0	0	0	0	1	0	0	0	0
09/08/2023	F	31	0	0	1	1	0	1	0	0	0	0	1	0	0	0	1
24/09/2024	M	31	1	1	1	0	0	0	0	0	0	0	1	1	0	0	0
23/10/2020	F	31	1	1	1	1	0	0	0	0	0	0	1	0	0	0	0
20/02/2024	F	31	1	1	1	1	0	0	0	0	0	0	1	1	0	0	0
22/10/2024	M	31	0	0	1	0	1	1	1	0	0	0	1	1	1	0	0
29/06/2023	M	31	1	1	1	0	1	1	1	0	0	0	1	0	0	0	0
05.03.2021	F	31	1	1	1	1	1	1	0	0	0	0	1	1	0	0	0
07/02/2020	F	32	0	0	0	0	0	0	0	0	0	0	0	0	0	0	1
11/02/2020	M	32	0	0	0	0	0	0	0	0	0	0	0	0	0	0	1
24/09/2020	F	32	0	0	0	0	0	0	0	0	0	0	1	0	0	0	0
20/11/2020	F	32	0	0	0	0	0	0	0	0	0	0	0	0	0	0	1
02/12/2020	F	32	0	0	0	0	0	0	0	0	0	0	0	0	0	0	1
09/02/2021	F	32	0	0	0	0	0	0	0	0	0	0	0	0	0	0	1
20/05/2021	F	32	0	0	0	0	0	0	0	0	0	0	0	0	0	0	1
08/09/2021	F	32	0	0	0	0	0	0	0	0	0	0	0	0	0	1	0
16/09/2021	M	32	0	0	0	0	0	0	0	0	0	0	1	1	0	0	0
29/09/2021	F	32	0	0	0	0	0	0	0	0	0	0	0	0	0	0	0
25/10/2021	F	32	0	0	0	0	0	0	0	0	0	0	0	0	0	0	1
17.02.2021	M	32	0	0	0	0	0	0	0	0	0	0	0	0	1	0	1
20.01.2021	F	32	0	0	0	0	0	0	0	0	0	0	1	0	0	0	0
24.02.2021	F	32	0	0	0	0	0	0	0	0	0	0	0	0	0	0	0
29.10.2021	F	32	0	0	0	0	0	0	0	0	0	0	0	0	0	1	0
29.10.2021	F	32	0	0	0	0	0	0	0	0	0	0	0	0	0	0	0
18/01/2022	F	32	0	0	0	0	0	0	0	0	0	0	1	0	0	0	0
19/01/2022	F	32	0	0	0	0	0	0	0	0	0	0	0	0	0	0	1
23/03/2022	M	32	0	0	0	0	0	0	0	0	0	0	1	1	0	0	0
25/05/2022	M	32	0	0	0	0	0	0	0	0	0	0	1	1	0	0	0

26/07/2022	M	32	0	0	0	0	0	0	0	0	0	0	0	0	0	0	0
12/09/2022	M	32	0	0	0	0	0	0	0	0	0	0	1	1	0	0	0
22/11/2022	F	32	0	0	0	0	0	0	0	0	0	0	0	0	0	0	1
24/02/2023	F	32	0	0	0	0	0	0	0	0	0	0	1	0	0	0	1
19/04/2023	M	32	0	0	0	0	0	0	0	0	0	0	0	0	0	0	1
05/12/2023	F	32	0	0	0	0	0	0	0	0	0	0	1	0	0	0	0
13/02/2024	F	32	0	0	0	0	0	0	0	0	0	0	0	0	1	0	0
06/03/2024	F	32	0	0	0	0	0	0	0	0	0	0	0	0	0	0	0
15/03/2024	F	32	0	0	0	0	0	0	0	0	0	0	0	0	0	0	0
26/04/2024	F	32	0	0	0	0	0	0	0	0	0	0	0	0	1	0	0
11/10/2024	F	32	0	0	0	0	0	0	0	0	0	0	0	0	0	0	1
29/11/2021	F	32	0	0	0	0	0	0	0	1	0	0	0	0	0	0	0
09.06.2021	M	32	0	0	0	0	0	0	0	0	0	1	0	1	0	0	0
10.09.2021	M	32	0	0	0	0	0	0	0	0	0	1	1	0	0	0	0
19.05.2021	F	32	0	0	0	0	0	0	0	0	0	1	0	0	0	0	0
04/02/2022	M	32	0	0	0	0	0	0	0	0	0	1	0	0	0	0	1
20/06/2022	F	32	0	0	0	0	0	0	0	1	0	0	0	0	0	0	0
12/05/2023	F	32	0	0	0	0	0	0	0	0	1	0	1	0	1	0	0
16/05/2023	F	32	0	0	0	0	0	0	0	0	1	0	0	0	0	0	1
09/04/2024	M	32	0	0	0	0	0	0	0	0	1	0	0	0	0	0	0
07.04.2021	F	32	0	0	0	0	0	0	0	1	0	1	1	0	0	1	1
08/07/2020	M	32	0	0	0	1	0	0	0	0	0	0	1	1	0	0	0
21/10/2020	M	32	0	0	0	0	1	0	0	0	0	0	1	0	1	0	0
14/12/2020	M	32	0	0	0	0	1	0	0	0	0	0	1	0	1	0	0
20/04/2021	F	32	0	0	0	0	1	0	0	0	0	0	0	0	0	0	1
17.03.2021	F	32	0	0	0	0	1	0	0	0	0	0	0	0	1	0	1
28/09/2020	F	32	0	0	1	0	0	0	0	0	0	0	1	1	0	0	0
09/09/2021	M	32	0	0	1	0	0	0	0	0	0	0	1	1	0	0	0
02/02/2022	M	32	0	0	1	0	0	0	0	0	0	0	1	0	0	0	0

01/03/2022	M	32	0	0	1	0	0	0	0	0	0	0	1	1	0	0	0
27/05/2022	M	32	0	0	1	0	0	0	0	0	0	0	1	1	0	0	0
30/08/2022	F	32	0	0	1	0	0	0	0	0	0	0	1	0	0	0	0
29/11/2022	F	32	0	0	1	0	0	0	0	0	0	0	1	0	0	0	1
03/03/2023	M	32	0	0	1	0	0	0	0	0	0	0	1	0	0	0	0
15/05/2023	M	32	0	0	1	0	0	0	0	0	0	0	1	1	0	0	0
06/10/2023	M	32	0	0	1	0	0	0	0	0	0	0	1	1	0	0	0
01/08/2024	F	32	0	0	1	0	0	0	0	0	0	0	1	1	1	0	0
02/08/2024	F	32	0	0	1	0	0	0	0	0	0	0	1	1	0	0	0
21/08/2024	M	32	0	0	1	0	0	0	0	0	0	0	1	1	0	0	0
21/08/2024	F	32	0	0	1	0	0	0	0	0	0	0	1	0	0	0	0
30/09/2024	M	32	0	0	1	0	0	0	0	0	0	0	1	1	0	0	0
13/11/2024	F	32	0	0	1	0	0	0	0	0	0	0	1	1	0	0	0
29/04/2022	F	32	0	1	0	0	0	0	0	0	0	0	1	1	0	0	0
29/04/2022	M	32	0	0	0	0	1	0	0	0	0	0	1	0	1	0	0
27/06/2022	M	32	0	1	0	0	0	0	0	0	0	0	1	1	1	0	0
20/07/2022	M	32	0	1	0	0	0	0	0	0	0	0	1	1	0	0	0
28/09/2022	F	32	0	0	0	0	0	0	1	0	0	0	1	0	1	0	0
08/05/2024	F	32	0	0	0	0	1	0	0	0	0	0	1	0	0	0	0
27/08/2024	M	32	0	1	0	0	0	0	0	0	0	0	1	1	0	0	0
19.11.2021	M	32	0	0	0	0	1	0	0	0	0	1	0	0	0	1	0
17.12.2021	F	32	0	0	1	0	0	0	0	0	0	1	0	0	0	0	0
22/02/2022	M	32	0	0	0	0	1	0	0	0	1	0	1	1	0	0	0
18/11/2022	M	32	0	0	0	0	0	0	1	1	0	0	1	0	0	0	0
05/06/2020	M	32	0	0	1	0	0	1	0	0	0	0	1	1	0	0	0
17/07/2020	M	32	0	0	1	0	1	0	0	0	0	0	1	1	0	0	0
25/02/2021	M	32	0	0	1	0	0	1	0	0	0	0	1	1	0	0	0
20/12/2021	F	32	0	0	1	0	0	1	0	0	0	0	1	1	0	0	0
25/02/2022	M	32	0	0	1	0	1	0	0	0	0	0	1	1	0	0	0

17/02/2023	F	32	0	0	1	1	0	0	0	0	0	0	1	0	0	0	0
11/03/2021	F	32	1	1	0	0	0	1	0	0	0	0	1	1	0	0	0
11/01/2021	M	32	0	1	1	0	1	0	0	0	0	0	1	0	0	0	1
04/10/2021	M	32	0	1	1	1	0	0	0	0	0	0	1	0	0	0	0
07/03/2022	M	32	0	0	1	0	1	0	1	0	0	0	1	0	1	0	0
13/07/2022	M	32	0	1	1	0	0	1	0	0	0	0	1	1	0	0	0
03/11/2023	M	32	0	0	1	0	1	1	0	0	0	0	1	0	1	0	0
20/03/2024	F	32	1	1	1	0	0	0	0	0	0	0	1	0	0	0	0
20/06/2022	M	32	1	0	1	0	0	1	0	1	0	0	0	0	0	0	0
18/09/2020	F	32	1	1	1	1	0	0	0	0	0	0	1	1	1	0	0
21/07/2022	F	32	0	1	1	1	0	1	0	0	0	0	1	1	1	0	0
15/05/2023	F	32	1	1	1	0	0	1	0	0	0	0	1	1	0	0	0
15/11/2024	M	32	1	1	1	1	0	0	0	0	0	0	1	1	0	0	0
21/03/2023	M	32	1	1	1	1	1	0	0	0	0	0	1	1	0	0	0
28/06/2023	M	32	1	1	1	0	1	1	0	0	0	0	1	0	0	0	0
27/08/2024	F	32	1	1	1	0	1	1	0	0	0	0	1	1	0	0	0
20.01.2021	F	32	0	1	1	0	1	1	1	0	0	0	1	1	0	0	0
27/01/2020	F	33	0	0	0	0	0	0	0	0	0	0	1	1	0	0	1
05/08/2020	F	33	0	0	0	0	0	0	0	0	0	0	1	1	0	0	0
15/09/2020	F	33	0	0	0	0	0	0	0	0	0	0	1	0	0	0	0
30/10/2020	F	33	0	0	0	0	0	0	0	0	0	0	1	1	0	0	0
04/11/2020	F	33	0	0	0	0	0	0	0	0	0	0	0	0	1	0	0
19/11/2020	M	33	0	0	0	0	0	0	0	0	0	0	0	0	1	0	0
07/01/2021	M	33	0	0	0	0	0	0	0	0	0	0	0	0	0	0	1
11/03/2021	M	33	0	0	0	0	0	0	0	0	0	0	0	0	0	0	1
25/03/2021	F	33	0	0	0	0	0	0	0	0	0	0	0	0	0	0	1
10/05/2021	M	33	0	0	0	0	0	0	0	0	0	0	0	0	1	1	0
12.11.2021	F	33	0	0	0	0	0	0	0	0	0	0	0	1	0	0	0
15.01.2021	M	33	0	0	0	0	0	0	0	0	0	0	0	0	1	0	0

25/01/2022	M	33	0	0	0	0	0	0	0	0	0	0	0	0	0	0	1
28/01/2022	F	33	0	0	0	0	0	0	0	0	0	0	1	0	0	0	1
10/02/2022	F	33	0	0	0	0	0	0	0	0	0	0	1	0	0	0	0
11/07/2022	M	33	0	0	0	0	0	0	0	0	0	0	0	0	0	0	1
13/07/2022	M	33	0	0	0	0	0	0	0	0	0	0	1	1	0	0	0
13/07/2022	F	33	0	0	0	0	0	0	0	0	0	0	1	0	0	0	0
20/07/2022	F	33	0	0	0	0	0	0	0	0	0	0	1	1	0	0	0
09/09/2022	F	33	0	0	0	0	0	0	0	0	0	0	1	0	0	0	0
19/09/2022	F	33	0	0	0	0	0	0	0	0	0	0	1	0	1	0	1
19/09/2022	M	33	0	0	0	0	0	0	0	0	0	0	1	1	0	0	0
24/10/2022	F	33	0	0	0	0	0	0	0	0	0	0	0	0	0	0	0
23/11/2022	M	33	0	0	0	0	0	0	0	0	0	0	0	0	0	0	0
02/03/2023	F	33	0	0	0	0	0	0	0	0	0	0	0	0	1	0	0
03/04/2023	F	33	0	0	0	0	0	0	0	0	0	0	1	0	1	0	0
07/04/2023	M	33	0	0	0	0	0	0	0	0	0	0	1	0	0	0	0
31/05/2023	F	33	0	0	0	0	0	0	0	0	0	0	1	0	1	0	0
28/07/2023	F	33	0	0	0	0	0	0	0	0	0	0	0	0	0	0	1
23/08/2023	F	33	0	0	0	0	0	0	0	0	0	0	0	0	0	0	1
23/08/2023	F	33	0	0	0	0	0	0	0	0	0	0	0	0	0	0	1
30/08/2023	F	33	0	0	0	0	0	0	0	0	0	0	1	0	0	0	0
14/12/2023	F	33	0	0	0	0	0	0	0	0	0	0	0	0	0	0	1
09/02/2024	M	33	0	0	0	0	0	0	0	0	0	0	1	0	0	0	0
30/04/2024	M	33	0	0	0	0	0	0	0	0	0	0	0	0	0	0	0
14/05/2024	F	33	0	0	0	0	0	0	0	0	0	0	0	0	0	0	1
01/07/2024	F	33	0	0	0	0	0	0	0	0	0	0	0	0	0	0	1
02/08/2024	F	33	0	0	0	0	0	0	0	0	0	0	1	0	0	0	0
25/09/2024	F	33	0	0	0	0	0	0	0	0	0	0	1	0	1	0	0
29/10/2024	M	33	0	0	0	0	0	0	0	0	0	0	1	0	1	0	0
14/11/2024	F	33	0	0	0	0	0	0	0	0	0	0	0	0	0	0	0

05/12/2024	F	33	0	0	0	0	0	0	0	0	0	0	0	0	0	0	1
10/12/2024	F	33	0	0	0	0	0	0	0	0	0	0	0	0	1	0	1
23/08/2025	F	33	0	0	0	0	0	0	0	0	0	0	0	0	0	0	1
18/07/2022	F	33	0	0	0	0	0	0	0	1	0	0	0	0	0	0	1
28/09/2022	F	33	0	0	0	0	0	0	0	1	0	0	0	0	0	0	0
09/02/2024	F	33	0	0	0	0	0	0	0	0	1	0	0	0	0	0	0
13/11/2024	M	33	0	0	0	0	0	0	0	0	1	0	0	0	0	0	0
04/12/2024	M	33	0	0	0	0	0	0	0	0	0	1	0	0	0	0	1
12/03/2020	M	33	0	1	0	0	0	0	0	0	0	0	1	0	1	0	0
22/02/2021	M	33	0	0	0	0	1	0	0	0	0	0	1	1	1	0	0
30/08/2021	F	33	0	0	0	0	1	0	0	0	0	0	1	1	1	0	0
05/08/2020	M	33	0	0	1	0	0	0	0	0	0	0	1	1	0	0	0
26/09/2022	M	33	0	0	1	0	0	0	0	0	0	0	1	1	0	0	0
09/08/2023	M	33	0	0	1	0	0	0	0	0	0	0	1	0	0	0	0
06/09/2023	F	33	0	0	1	0	0	0	0	0	0	0	1	0	0	0	0
12/01/2024	F	33	0	0	1	0	0	0	0	0	0	0	1	1	0	0	0
25/09/2024	M	33	0	0	1	0	0	0	0	0	0	0	1	0	0	0	0
07/10/2024	M	33	0	0	1	0	0	0	0	0	0	0	1	1	0	0	0
09/10/2024	F	33	0	0	1	0	0	0	0	0	0	0	1	0	0	0	0
10/10/2024	M	33	0	0	1	0	0	0	0	0	0	0	1	1	0	0	0
06/11/2024	F	33	0	0	1	0	0	0	0	0	0	0	1	0	0	0	0
05.03.2021	F	33	0	0	1	0	0	0	0	0	0	0	1	0	0	0	0
23/08/2022	M	33	0	0	0	0	1	0	0	0	0	0	0	0	0	0	1
04/01/2023	F	33	0	0	0	0	1	0	0	0	0	0	1	0	0	0	0
06/03/2023	F	33	0	0	0	0	0	1	0	0	0	0	1	0	0	0	1
12/04/2023	M	33	0	0	0	0	1	0	0	0	0	0	1	0	1	0	0
12/04/2024	F	33	0	0	0	0	1	0	0	0	0	0	1	1	0	0	0
28/05/2024	F	33	1	0	0	0	0	0	0	0	0	0	1	1	0	0	0
28/05/2024	M	33	0	0	0	0	1	0	0	0	0	0	1	0	0	0	0

04/11/2020	F	33	1	0	0	0	0	0	0	0	0	1	1	0	0	0	0
17.12.2021	M	33	0	0	0	0	1	0	0	0	0	1	0	0	0	0	0
06/03/2022	F	33	0	0	1	0	0	0	0	0	1	0	1	0	0	0	1
01/04/2024	M	33	0	0	1	0	0	0	0	0	0	1	1	0	0	0	0
11/08/2020	M	33	1	0	1	0	0	0	0	0	0	0	1	1	0	0	0
03/12/2020	M	33	0	0	1	1	0	0	0	0	0	0	1	1	0	0	0
11/01/2022	F	33	0	0	1	0	1	0	0	0	0	0	1	0	0	0	0
04/04/2022	F	33	0	1	1	0	0	0	0	0	0	0	1	0	0	0	0
20/11/2024	M	33	1	0	1	0	0	0	0	0	0	0	1	0	0	0	1
19/12/2024	F	33	0	0	1	0	1	0	0	0	0	0	1	0	1	0	0
16/05/2022	M	33	1	1	0	0	0	0	0	0	0	0	1	1	0	0	0
19/04/2023	F	33	0	0	1	1	0	0	0	0	1	0	1	0	0	0	0
15/03/2021	M	33	0	1	1	1	0	0	0	0	0	0	1	1	1	0	0
08/06/2021	M	33	0	1	1	1	0	0	0	0	0	0	1	1	0	0	0
12/01/2022	M	33	0	1	1	0	1	0	0	0	0	0	1	0	1	0	0
01/03/2023	F	33	0	1	1	0	0	1	0	0	0	0	1	0	0	0	0
13/03/2023	F	33	0	0	1	0	0	1	1	0	0	0	1	0	0	0	0
27/08/2024	M	33	0	1	1	0	1	0	0	0	0	0	1	0	0	0	0
22/08/2022	F	33	1	1	0	0	1	0	0	0	0	0	1	1	0	0	0
21.12.2021	M	33	0	1	1	1	0	0	0	0	0	1	0	0	0	0	0
15/12/2022	F	33	0	1	1	0	0	0	1	1	1	0	1	1	1	0	0
09/03/2022	F	33	1	1	1	0	0	1	0	0	0	0	1	1	0	0	0
27/10/2023	M	33	1	1	1	0	0	1	0	0	0	0	1	0	1	0	0
15/01/2020	F	34	0	0	0	0	0	0	0	0	0	0	1	0	0	0	1
02/03/2020	F	34	0	0	0	0	0	0	0	0	0	0	0	0	0	0	1
02/03/2020	M	34	0	0	0	0	0	0	0	0	0	0	1	1	0	0	0
17/06/2020	F	34	0	0	0	0	0	0	0	0	0	0	0	0	0	0	1
22/09/2020	M	34	0	0	0	0	0	0	0	0	0	0	1	1	0	0	0
04/11/2020	F	34	0	0	0	0	0	0	0	0	0	0	0	0	0	0	1

23/11/2020	F	34	0	0	0	0	0	0	0	0	0	0	1	0	1	0	0
20/05/2021	F	34	0	0	0	0	0	0	0	0	0	0	0	0	0	0	1
07/06/2021	M	34	0	0	0	0	0	0	0	0	0	0	0	0	0	0	1
29/09/2021	F	34	0	0	0	0	0	0	0	0	0	0	0	0	0	0	0
18/10/2021	F	34	0	0	0	0	0	0	0	0	0	0	0	0	0	0	1
12.05.2021	F	34	0	0	0	0	0	0	0	0	0	0	0	0	0	1	0
21.05.2021	F	34	0	0	0	0	0	0	0	0	0	0	0	1	0	0	0
24.11.2021	M	34	0	0	0	0	0	0	0	0	0	0	0	0	0	0	0
26.02.2021	F	34	0	0	0	0	0	0	0	0	0	0	0	0	0	1	0
31.03.2021	F	34	0	0	0	0	0	0	0	0	0	0	0	0	1	0	1
11/01/2022	F	34	0	0	0	0	0	0	0	0	0	0	0	0	0	0	0
18/02/2022	M	34	0	0	0	0	0	0	0	0	0	0	1	0	0	0	0
21/03/2022	M	34	0	0	0	0	0	0	0	0	0	0	1	0	1	0	0
21/03/2022	F	34	0	0	0	0	0	0	0	0	0	0	1	0	0	0	0
25/05/2022	F	34	0	0	0	0	0	0	0	0	0	0	0	0	0	0	0
05/09/2022	M	34	0	0	0	0	0	0	0	0	0	0	1	1	0	0	0
29/09/2022	M	34	0	0	0	0	0	0	0	0	0	0	0	0	0	0	1
17/10/2022	F	34	0	0	0	0	0	0	0	0	0	0	0	0	0	0	1
08/02/2023	F	34	0	0	0	0	0	0	0	0	0	0	0	0	0	0	1
19/04/2023	F	34	0	0	0	0	0	0	0	0	0	0	0	0	0	0	1
10/05/2023	F	34	0	0	0	0	0	0	0	0	0	0	0	0	0	0	1
17/08/2023	F	34	0	0	0	0	0	0	0	0	0	0	0	0	0	0	0
27/09/2023	F	34	0	0	0	0	0	0	0	0	0	0	1	0	0	0	0
11/12/2023	F	34	0	0	0	0	0	0	0	0	0	0	0	0	0	0	0
19/01/2024	F	34	0	0	0	0	0	0	0	0	0	0	1	0	0	0	0
23/02/2024	F	34	0	0	0	0	0	0	0	0	0	0	1	0	0	0	0
29/03/2024	M	34	0	0	0	0	0	0	0	0	0	0	0	0	0	0	1
21/05/2024	F	34	0	0	0	0	0	0	0	0	0	0	0	0	0	0	1
02/07/2024	F	34	0	0	0	0	0	0	0	0	0	0	0	0	0	0	1

10/07/2024	F	34	0	0	0	0	0	0	0	0	0	0	0	0	0	0	1
23/07/2024	F	34	0	0	0	0	0	0	0	0	0	0	0	0	0	0	1
27/09/2024	M	34	0	0	0	0	0	0	0	0	0	0	0	0	0	0	0
07/10/2024	M	34	0	0	0	0	0	0	0	0	0	0	0	1	0	0	0
03/12/2024	F	34	0	0	0	0	0	0	0	0	0	0	0	0	0	0	0
24/04/2021	F	34	0	0	0	0	0	0	0	0	0	1	0	0	0	0	1
07/10/2021	F	34	0	0	0	0	0	0	0	0	1	0	0	0	0	0	0
25/10/2021	F	34	0	0	0	0	0	0	0	0	1	0	0	0	0	0	0
14/12/2021	F	34	0	0	0	0	0	0	0	0	1	0	1	1	0	0	0
21.12.2021	M	34	0	0	0	0	0	0	0	0	1	0	0	0	0	0	0
27/03/2023	M	34	0	0	0	0	0	0	0	0	1	0	0	0	0	0	0
18/10/2023	F	34	0	0	0	0	0	0	0	0	1	0	0	0	0	0	0
02/04/2024	F	34	0	0	0	0	0	0	0	0	1	0	0	0	0	0	0
17/06/2020	F	34	0	0	0	0	1	0	0	0	0	0	1	1	0	0	0
06/05/2021	F	34	0	0	0	0	1	0	0	0	0	0	0	0	0	0	0
23/08/2021	F	34	0	0	0	0	0	0	1	0	0	0	1	0	0	0	0
26.11.2021	F	34	0	0	0	0	1	0	0	0	0	0	1	0	0	0	0
12/02/2020	M	34	0	0	1	0	0	0	0	0	0	0	1	1	0	0	0
24/09/2020	M	34	0	0	1	0	0	0	0	0	0	0	1	1	0	0	0
18/03/2021	F	34	0	0	1	0	0	0	0	0	0	0	0	0	1	0	0
30/08/2022	F	34	0	0	1	0	0	0	0	0	0	0	1	1	0	0	0
04/09/2023	M	34	0	0	1	0	0	0	0	0	0	0	1	1	0	0	0
19/09/2023	F	34	0	0	1	0	0	0	0	0	0	0	1	1	0	0	0
23/05/2024	M	34	0	0	1	0	0	0	0	0	0	0	1	1	0	0	0
31/05/2022	F	34	0	0	0	0	1	0	0	0	0	0	1	0	0	0	0
28/03/2023	F	34	0	0	0	0	1	0	0	0	0	0	1	0	1	0	0
16/01/2024	M	34	0	0	0	0	1	0	0	0	0	0	1	1	0	0	0
04/03/2024	M	34	0	1	0	0	0	0	0	0	0	0	1	1	1	0	0
06/03/2024	M	34	0	0	0	0	1	0	0	0	0	0	1	0	1	0	0

15.12.2021	F	34	0	0	1	0	0	0	0	0	0	1	0	0	0	0	0
05/02/2020	F	34	0	0	1	0	1	0	0	0	0	0	1	1	0	0	0
05/02/2020	F	34	0	0	1	0	0	1	0	0	0	0	1	1	0	0	0
09/03/2021	M	34	0	0	1	0	1	0	0	0	0	0	1	1	1	1	0
13/05/2021	F	34	0	0	1	1	0	0	0	0	0	0	1	0	0	0	0
26/01/2022	M	34	1	0	1	0	0	0	0	0	0	0	1	1	0	0	0
08/06/2022	M	34	0	1	1	0	0	0	0	0	0	0	1	1	0	0	0
21/07/2022	F	34	0	0	1	0	0	1	0	0	0	0	1	1	0	0	0
18/08/2023	M	34	0	0	1	0	1	0	0	0	0	0	1	0	0	0	0
31/08/2023	M	34	0	0	1	0	1	0	0	0	0	0	1	1	0	0	0
01/11/2023	F	34	0	0	1	0	1	0	0	0	0	0	1	0	0	0	1
19/01/2024	M	34	0	0	1	0	0	1	0	0	0	0	1	1	0	0	0
06/09/2024	F	34	0	0	1	0	1	0	0	0	0	0	1	1	1	0	0
06/09/2024	M	34	0	0	1	1	0	0	0	0	0	0	1	1	1	0	0
01/11/2023	F	34	0	1	0	0	1	0	0	0	0	0	1	0	0	0	0
02/03/2020	F	34	0	1	1	0	0	0	0	0	1	0	1	1	1	0	0
15.12.2021	M	34	0	0	1	0	1	0	0	0	0	1	0	1	0	0	0
27.08.2021	M	34	0	0	1	1	0	0	0	0	0	1	1	0	0	0	0
01/07/2020	F	34	0	1	1	0	0	1	0	0	0	0	1	1	0	0	1
23/03/2022	M	34	0	1	1	0	1	0	0	0	0	0	1	1	0	0	0
26/10/2022	M	34	1	1	1	0	0	0	0	0	0	0	1	1	1	0	0
08/02/2023	F	34	0	0	1	1	0	1	0	0	0	0	1	0	0	0	0
15/03/2023	M	34	1	1	1	0	0	0	0	0	0	0	1	0	0	0	0
09.03.2021	M	34	0	1	1	1	0	0	0	0	0	0	1	1	0	0	0
10.03.2021	F	34	0	1	1	1	0	0	0	0	0	0	1	0	1	0	1
06/10/2023	F	34	0	0	0	0	1	1	1	0	0	0	1	0	0	0	0
31/08/2020	M	34	1	1	1	0	1	0	0	0	0	0	1	0	0	0	0
25/08/2022	F	34	1	1	1	0	0	0	1	0	0	0	1	1	1	0	0
13/10/2023	F	34	1	1	1	0	1	0	0	0	0	0	1	0	0	0	0

19/03/2024	F	34	0	1	1	1	0	1	0	0	0	1	1	1	0	0	0
16/09/2020	M	34	1	1	1	1	1	1	1	0	0	0	1	1	0	0	0
13/02/2020	M	35	0	0	0	0	0	0	0	0	0	0	1	1	0	0	0
17/02/2020	M	35	0	0	0	0	0	0	0	0	0	0	1	0	0	0	0
19/05/2020	F	35	0	0	0	0	0	0	0	0	0	0	0	0	0	0	1
28/08/2020	M	35	0	0	0	0	0	0	0	0	0	0	1	1	0	0	0
12/11/2020	M	35	0	0	0	0	0	0	0	0	0	0	0	0	0	0	1
03/12/2020	M	35	0	0	0	0	0	0	0	0	0	0	0	0	1	0	0
04/04/2021	F	35	0	0	0	0	0	0	0	0	0	0	0	0	0	0	1
14/09/2021	M	35	0	0	0	0	0	0	0	0	0	0	1	1	0	0	0
09.06.2021	F	35	0	0	0	0	0	0	0	0	0	0	0	0	0	1	0
15.12.2021	M	35	0	0	0	0	0	0	0	0	0	0	0	1	0	1	0
30/03/2022	M	35	0	0	0	0	0	0	0	0	0	0	1	1	0	0	0
13/04/2022	F	35	0	0	0	0	0	0	0	0	0	0	0	0	1	0	0
13/04/2022	M	35	0	0	0	0	0	0	0	0	0	0	0	0	0	0	0
20/04/2022	M	35	0	0	0	0	0	0	0	0	0	0	0	0	0	0	1
26/04/2022	F	35	0	0	0	0	0	0	0	0	0	0	1	0	0	0	0
13/05/2022	M	35	0	0	0	0	0	0	0	0	0	0	1	1	0	0	0
08/06/2022	F	35	0	0	0	0	0	0	0	0	0	0	1	0	1	0	0
22/06/2022	F	35	0	0	0	0	0	0	0	0	0	0	0	0	0	0	1
27/06/2022	F	35	0	0	0	0	0	0	0	0	0	0	0	0	0	0	1
18/07/2022	F	35	0	0	0	0	0	0	0	0	0	0	1	1	0	0	0
19/09/2022	M	35	0	0	0	0	0	0	0	0	0	0	0	0	1	0	0
24/10/2022	F	35	0	0	0	0	0	0	0	0	0	0	0	0	0	0	1
04/11/2022	M	35	0	0	0	0	0	0	0	0	0	0	0	0	1	0	0
28/04/2023	F	35	0	0	0	0	0	0	0	0	0	0	1	0	0	0	0
30/05/2023	M	35	0	0	0	0	0	0	0	0	0	0	1	0	0	0	0
02/08/2023	F	35	0	0	0	0	0	0	0	0	0	0	0	0	0	0	1
16/08/2023	F	35	0	0	0	0	0	0	0	0	0	0	0	0	0	0	1

02/10/2023	F	35	0	0	0	0	0	0	0	0	0	0	0	0	0	0	1
03/11/2023	M	35	0	0	0	0	0	0	0	0	0	0	0	0	1	0	0
10/11/2023	F	35	0	0	0	0	0	0	0	0	0	0	1	0	0	0	0
10/01/2024	M	35	0	0	0	0	0	0	0	0	0	0	0	0	0	0	1
09/02/2024	F	35	0	0	0	0	0	0	0	0	0	0	0	0	0	0	0
28/02/2024	F	35	0	0	0	0	0	0	0	0	0	0	0	0	0	0	1
24/04/2024	M	35	0	0	0	0	0	0	0	0	0	0	1	0	1	1	0
27/08/2024	F	35	0	0	0	0	0	0	0	0	0	0	0	0	0	0	0
18/09/2024	M	35	0	0	0	0	0	0	0	0	0	0	0	0	0	0	1
25/10/2024	F	35	0	0	0	0	0	0	0	0	0	0	0	0	0	0	0
05/08/2021	F	35	0	0	0	0	0	0	0	0	1	0	0	0	0	0	1
01/09/2021	F	35	0	0	0	0	0	0	0	0	0	1	1	0	0	0	0
25/10/2021	F	35	0	0	0	0	0	0	0	0	1	0	0	0	0	0	0
07/09/2022	F	35	0	0	0	0	0	0	0	1	0	0	1	1	0	0	0
30/08/2023	F	35	0	0	0	0	0	0	0	0	0	1	0	0	1	0	0
22/03/2021	F	35	0	0	0	0	1	0	0	0	0	0	1	0	1	0	1
29/03/2021	F	35	0	0	0	0	0	1	0	0	0	0	1	1	0	0	0
29/07/2020	M	35	0	0	1	0	0	0	0	0	0	0	1	1	1	0	0
23/08/2022	M	35	0	0	1	0	0	0	0	0	0	0	1	1	1	0	0
17/02/2023	F	35	0	0	1	0	0	0	0	0	0	0	1	0	0	0	1
30/08/2023	F	35	0	0	1	0	0	0	0	0	0	0	1	0	1	0	0
02/10/2023	M	35	0	0	1	0	0	0	0	0	0	0	1	0	0	0	0
15/05/2024	F	35	0	0	1	0	0	0	0	0	0	0	1	1	0	0	0
01/08/2024	F	35	0	0	1	0	0	0	0	0	0	0	0	0	0	0	0
27.01.2021	M	35	0	0	1	0	0	0	0	0	0	0	1	1	1	0	0
11/01/2022	M	35	0	0	0	0	1	0	0	0	0	0	1	0	0	0	0
19/01/2022	F	35	0	0	0	0	1	0	0	0	0	0	1	0	0	0	0
06/06/2023	F	35	1	0	0	0	0	0	0	0	0	0	1	1	0	0	0
13/09/2021	F	35	0	0	1	0	0	0	0	0	1	0	1	1	0	0	0

31/08/2022	M	35	0	0	1	0	0	0	0	1	0	0	1	1	1	0	0
07.04.2021	F	35	0	0	1	0	0	0	0	0	0	1	1	0	0	0	0
17.12.2021	M	35	0	0	1	0	0	0	0	0	0	1	0	0	0	1	0
15/01/2020	F	35	0	1	0	0	1	0	0	0	0	0	1	1	1	0	1
07/09/2020	F	35	1	1	0	0	0	0	0	0	0	0	1	0	1	0	0
10/05/2021	M	35	0	0	0	0	1	1	0	0	0	0	1	0	1	0	0
09/01/2020	F	35	0	0	1	0	1	0	0	0	0	0	1	1	0	0	0
08/03/2022	F	35	0	0	1	1	0	0	0	0	0	0	1	0	0	0	1
10/06/2022	M	35	0	0	1	0	0	1	0	0	0	0	1	1	0	0	0
16/06/2022	F	35	0	0	1	0	1	0	0	0	0	0	1	1	1	0	0
21/04/2023	M	35	1	0	1	0	0	0	0	0	0	0	1	0	0	0	1
08/05/2023	M	35	1	0	1	0	0	0	0	0	0	0	1	1	0	0	0
16/06/2023	M	35	0	1	1	0	0	0	0	0	0	0	1	0	0	0	0
24/11/2023	F	35	0	0	1	0	1	0	0	0	0	0	1	0	1	1	0
15.01.2021	M	35	0	0	1	1	0	0	0	0	0	0	1	1	0	0	0
28.05.2021	F	35	0	0	1	0	1	0	0	0	0	1	0	1	0	0	0
31/08/2020	F	35	0	1	1	0	1	0	0	0	0	0	1	1	1	0	0
22/09/2020	M	35	1	1	1	0	0	0	0	0	0	0	1	0	0	0	0
29/10/2020	F	35	0	0	1	0	1	0	1	0	0	0	1	1	0	0	0
26/06/2023	M	35	0	1	1	0	0	1	0	0	0	0	1	1	1	0	0
28/08/2023	F	35	0	1	1	0	1	0	0	0	0	0	1	1	0	0	0
04/03/2024	M	35	0	1	1	0	0	1	0	0	0	0	1	1	1	0	0
25/05/2024	F	35	1	1	1	0	0	0	0	0	0	0	1	0	0	0	0
30/07/2024	F	35	0	0	1	0	1	1	0	0	0	0	1	1	0	0	0
06.01.2021	M	35	0	0	1	1	0	0	1	0	0	0	1	1	0	0	0
14/12/2021	F	35	1	1	0	0	1	0	0	0	0	1	0	0	1	0	0
28/07/2020	F	35	0	1	1	0	1	1	0	0	0	0	1	1	1	0	0
18/08/2023	F	35	0	1	1	0	1	1	0	0	0	0	1	0	0	0	0
31/07/2024	F	35	0	1	1	0	1	1	0	0	0	0	1	1	0	0	0

12/08/2020	M	35	0	1	1	1	1	0	0	1	0	0	1	0	0	0	0
22.10.2021	M	35	0	1	1	0	1	1	0	0	0	1	1	0	0	0	0
13/05/2022	F	35	1	1	1	1	0	0	1	0	0	0	1	1	0	0	0
04/10/2022	M	35	1	1	1	1	0	0	1	0	0	0	1	1	1	0	0
29/07/2020	F	35	1	1	1	1	1	1	0	0	0	0	1	1	0	0	0
13/01/2020	M	36	0	0	0	0	0	0	0	0	0	0	0	0	1	0	0
09/06/2020	F	36	0	0	0	0	0	0	0	0	0	0	0	0	0	0	0
15/07/2020	F	36	0	0	0	0	0	0	0	0	0	0	1	0	0	0	0
07/08/2020	M	36	0	0	0	0	0	0	0	0	0	0	1	0	0	0	0
22/10/2020	M	36	0	0	0	0	0	0	0	0	0	0	1	1	1	0	0
16/12/2020	M	36	1	1	0	0	1	0	0	0	0	0	1	1	0	0	0
16/01/2021	M	36	0	0	0	0	0	0	0	0	0	0	0	0	0	0	0
30/03/2021	M	36	0	0	0	0	0	0	0	0	0	0	0	0	1	0	0
08/04/2021	F	36	0	0	0	0	0	0	0	0	0	0	0	0	0	0	1
10/06/2021	M	36	0	0	0	0	0	0	0	0	0	0	1	1	0	0	0
17/06/2021	F	36	0	0	0	0	0	0	0	0	0	0	0	0	1	0	0
01/07/2021	F	36	0	0	0	0	0	0	0	0	0	0	1	0	0	0	0
31/08/2021	F	36	0	0	0	0	0	0	0	0	0	0	0	0	1	0	1
03.09.2021	F	36	0	0	0	0	0	0	0	0	0	0	0	0	0	1	0
21.12.2021	M	36	0	0	0	0	0	0	0	0	0	0	0	0	0	0	0
28.05.2021	M	36	0	0	0	0	0	0	0	0	0	0	0	0	0	1	0
25/02/2022	M	36	0	0	0	0	0	0	0	0	0	0	0	0	0	0	0
12/04/2022	F	36	0	0	0	0	0	0	0	0	0	0	1	1	1	0	1
13/05/2022	F	36	0	0	0	0	0	0	0	0	0	0	1	1	0	0	0
08/06/2022	F	36	0	0	0	0	0	0	0	0	0	0	0	0	0	0	1
03/11/2022	M	36	0	0	0	0	0	0	0	0	0	0	0	0	0	0	1
06/01/2023	F	36	0	0	0	0	0	0	0	0	0	0	0	0	0	0	1
10/01/2023	F	36	0	0	0	0	0	0	0	0	0	0	0	0	1	0	0
16/01/2023	M	36	0	0	0	0	0	0	0	0	0	0	0	0	0	0	1

27/01/2023	M	36	0	0	0	0	0	0	0	0	0	0	1	0	0	0	0
02/08/2023	M	36	0	0	0	0	0	0	0	0	0	0	1	0	0	0	0
16/01/2024	F	36	0	0	0	0	0	0	0	0	0	0	1	0	1	0	0
17/01/2024	F	36	0	0	0	0	0	0	0	0	0	0	0	0	1	0	0
14/02/2024	M	36	0	0	0	0	0	0	0	0	0	0	0	0	0	0	1
16/02/2024	F	36	0	0	0	0	0	0	0	0	0	0	0	0	0	0	0
23/02/2024	F	36	0	0	0	0	0	0	0	0	0	0	1	1	0	0	0
08/05/2024	F	36	0	0	0	0	0	0	0	0	0	0	0	0	0	0	0
02/07/2024	F	36	0	0	0	0	0	0	0	0	0	0	1	0	0	0	1
28/08/2024	M	36	0	0	0	0	0	0	0	0	0	0	0	0	0	0	1
02/12/2021	M	36	0	0	0	0	0	0	0	0	1	0	0	0	0	0	0
10.03.2021	F	36	0	0	0	0	0	0	0	0	1	0	0	0	0	0	0
22.10.2021	F	36	0	0	0	0	0	0	0	0	0	1	0	0	0	0	0
24/06/2022	F	36	0	0	0	0	0	0	0	1	0	0	0	0	0	0	0
20/09/2022	F	36	0	0	0	0	0	0	0	0	1	0	0	0	0	0	1
18/11/2020	F	36	0	0	0	0	1	0	0	0	0	0	1	0	1	0	0
17/06/2021	F	36	0	0	0	0	0	1	0	0	0	0	1	0	0	0	0
16/11/2021	F	36	0	0	0	0	1	0	0	0	0	0	1	1	0	0	0
11/08/2020	M	36	0	0	1	0	0	0	0	0	0	0	1	0	0	0	0
15/05/2021	M	36	0	0	1	0	0	0	0	0	0	0	1	1	0	0	0
01/09/2022	M	36	0	0	1	0	0	0	0	0	0	0	1	1	0	0	0
02/11/2022	F	36	0	0	1	0	0	0	0	0	0	0	1	0	0	0	1
30/08/2023	F	36	0	0	1	0	0	0	0	0	0	0	1	0	1	1	0
11/10/2023	F	36	0	0	1	0	0	0	0	0	0	0	1	0	0	0	0
09/02/2024	F	36	0	0	1	0	0	0	0	0	0	0	1	0	0	0	0
18/06/2024	F	36	0	0	1	0	0	0	0	0	0	0	1	0	0	0	0
04/09/2024	M	36	0	0	1	0	0	0	0	0	0	0	1	1	0	0	0
22/10/2024	F	36	0	0	1	0	0	0	0	0	0	0	1	1	0	0	0
18/03/2022	M	36	0	0	0	0	1	0	0	0	0	0	1	1	0	0	0

30/05/2023	F	36	0	1	0	0	0	0	0	0	0	0	1	1	0	0	0
24/08/2023	F	36	0	0	0	0	1	0	0	0	0	0	1	0	0	0	0
18/06/2024	M	36	0	1	0	0	0	0	0	0	0	0	1	0	0	0	0
11/11/2020	F	36	0	0	0	0	1	0	0	1	0	0	1	0	1	0	0
26/08/2021	F	36	0	0	1	0	0	0	0	0	1	0	1	0	1	0	1
06.10.2021	M	36	0	0	1	0	0	0	0	0	0	1	1	0	0	0	0
13/10/2020	M	36	1	1	0	0	0	0	0	0	0	0	1	0	1	0	0
11/06/2020	F	36	0	0	1	0	0	1	0	0	0	0	1	0	0	0	0
17/09/2020	M	36	0	0	1	0	0	1	0	0	0	0	1	1	0	0	0
28/10/2020	M	36	0	0	1	0	1	0	0	0	0	0	1	1	1	0	0
11/11/2021	M	36	1	0	1	0	0	0	0	0	0	0	1	1	1	0	0
30/08/2022	M	36	0	1	1	0	0	0	0	0	0	0	1	1	0	0	0
16/12/2022	F	36	0	0	1	0	0	1	0	0	0	0	1	0	1	0	0
25/08/2023	F	36	0	1	1	0	0	0	0	0	0	0	1	0	0	0	0
08/04/2024	F	36	1	1	0	0	0	0	0	0	0	0	1	0	0	0	0
17.03.2021	F	36	1	1	0	0	0	0	0	0	1	0	1	1	0	0	0
27.10.2021	M	36	0	0	0	0	1	1	0	0	0	1	0	1	0	0	0
24/09/2020	F	36	1	0	0	0	1	1	0	0	0	0	1	1	1	0	0
03/02/2021	M	36	1	1	0	0	1	0	0	0	0	0	1	1	0	0	0
17/02/2020	M	36	0	1	1	0	0	1	0	0	0	0	1	1	0	0	1
25/05/2022	M	36	0	1	1	1	0	0	0	0	0	0	1	0	0	0	0
04/10/2022	F	36	0	1	1	0	1	0	0	0	0	0	1	1	0	0	0
11/10/2022	F	36	1	1	1	0	0	0	0	0	0	0	1	0	1	0	0
21/03/2023	M	36	1	1	1	0	0	0	0	0	0	0	1	1	0	0	0
20/06/2023	F	36	1	1	1	0	0	0	0	0	0	0	1	1	0	0	0
11/08/2023	M	36	1	1	1	0	0	0	0	0	0	0	1	0	1	0	0
16/08/2023	F	36	1	1	1	0	0	0	0	0	0	0	1	0	0	0	0
26.11.2021	M	36	0	0	1	0	1	0	1	0	0	1	0	0	0	0	0
20/05/2021	F	36	0	1	1	0	1	1	0	0	0	0	1	1	1	0	0

14/06/2021	M	36	1	1	1	1	0	0	0	0	0	0	1	1	1	0	0
21/09/2022	M	36	1	1	1	0	0	1	0	0	0	0	1	1	0	0	0
15/11/2023	M	36	0	1	1	1	1	0	0	0	0	0	1	0	0	0	0
12/04/2024	F	36	0	1	1	0	1	1	0	0	0	0	1	1	1	0	1
07.04.2021	M	36	0	1	1	0	1	1	0	0	0	1	1	0	1	0	0
26.05.2021	F	36	1	0	1	1	1	0	0	0	0	1	1	1	0	0	0
02/08/2021	F	36	0	1	1	1	1	1	0	0	0	0	1	1	0	0	0
17.02.2021	F	36	1	1	1	0	0	1	1	0	0	0	1	1	0	0	0
22.09.2021	F	36	0	1	1	1	1	1	1	0	0	1	1	1	0	0	0
06/01/2020	M	37	0	0	0	0	0	0	0	0	0	0	0	0	0	0	1
27/02/2020	M	37	0	0	0	0	0	0	0	0	0	0	1	0	0	0	0
12/03/2020	M	37	0	0	0	0	0	0	0	0	0	0	0	0	0	0	1
17/03/2020	M	37	0	0	0	0	0	0	0	0	0	0	1	0	0	0	0
18/11/2020	F	37	0	0	0	0	0	0	0	0	0	0	1	0	1	0	0
12/01/2021	F	37	0	0	0	0	0	0	0	0	0	0	0	0	0	0	1
22/02/2021	M	37	0	0	0	0	0	0	0	0	0	0	0	0	0	0	0
29/03/2021	F	37	0	0	0	0	0	0	0	0	0	0	0	0	0	0	0
12/04/2021	F	37	0	0	0	0	0	0	0	0	0	0	0	0	0	0	1
25/05/2021	M	37	0	0	0	0	0	0	0	0	0	0	1	1	0	0	1
10/06/2021	M	37	0	0	0	0	0	0	0	0	0	0	0	0	0	0	0
11/11/2021	F	37	0	0	0	0	0	0	0	0	0	0	0	0	0	0	0
18/11/2021	M	37	0	0	0	0	0	0	0	0	0	0	0	0	0	0	0
08.12.2021	F	37	0	0	0	0	0	0	0	0	0	0	0	0	0	0	0
17.02.2021	M	37	0	0	0	0	0	0	0	0	0	0	0	0	0	0	0
18/01/2022	M	37	0	0	0	0	0	0	0	0	0	0	1	0	0	0	0
28/01/2022	F	37	0	0	0	0	0	0	0	0	0	0	0	0	0	0	0
06/03/2022	M	37	0	0	0	0	0	0	0	0	0	0	1	0	0	0	0
09/03/2022	F	37	0	0	0	0	0	0	0	0	0	0	0	1	0	0	0
30/03/2022	F	37	0	0	0	0	0	0	0	0	0	0	1	0	1	0	0

20/04/2022	M	37	0	0	0	0	0	0	0	0	0	0	0	0	0	0	0
19/07/2022	F	37	0	0	0	0	0	0	0	0	0	0	0	0	0	0	0
26/07/2022	F	37	0	0	0	0	0	0	0	0	0	0	0	0	0	0	1
31/08/2022	M	37	0	0	0	0	0	0	0	0	0	0	0	0	0	0	1
07/12/2022	M	37	0	0	0	0	0	0	0	0	0	0	0	0	0	0	0
21/12/2022	F	37	0	0	0	0	0	0	0	0	0	0	0	0	0	0	1
22/02/2023	F	37	0	0	0	0	0	0	0	0	0	0	0	0	0	0	1
23/02/2023	M	37	0	0	0	0	0	0	0	0	0	0	0	0	0	0	0
23/02/2023	F	37	0	0	0	0	0	0	0	0	0	0	0	0	0	0	1
06/04/2023	F	37	0	0	0	0	0	0	0	0	0	0	0	0	0	0	0
12/04/2023	F	37	0	0	0	0	0	0	0	0	0	0	1	0	1	0	0
31/05/2023	F	37	0	0	0	0	0	0	0	0	0	0	0	0	1	0	0
15/06/2023	F	37	0	0	0	0	0	0	0	0	0	0	0	0	0	0	0
13/04/2024	F	37	0	0	0	0	0	0	0	0	0	0	0	0	1	0	0
23/04/2024	F	37	0	0	0	0	0	0	0	0	0	0	0	0	0	0	1
26/04/2024	M	37	0	0	0	0	0	0	0	0	0	0	0	0	1	1	0
22/10/2024	M	37	0	0	0	0	0	0	0	0	0	0	1	0	0	0	0
25/10/2024	F	37	0	0	0	0	0	0	0	0	0	0	1	0	1	0	1
27.10.2021	F	37	0	0	0	0	0	0	0	0	0	1	1	0	0	0	0
06/11/2024	F	37	0	0	0	0	0	0	0	1	0	0	0	0	0	0	0
11/03/2021	F	37	0	0	0	0	0	0	0	1	0	1	0	0	0	0	0
10/12/2020	M	37	1	0	0	0	0	0	0	0	0	0	1	0	0	0	0
20/09/2021	M	37	0	0	1	0	0	0	0	0	0	0	1	0	0	0	1
21/09/2021	M	37	0	0	1	0	0	0	0	0	0	0	1	0	0	0	0
07/01/2022	F	37	0	0	1	0	0	0	0	0	0	0	1	0	0	0	0
14/01/2022	F	37	0	0	1	0	0	0	0	0	0	0	1	1	1	0	0
21/09/2022	M	37	0	0	1	0	0	0	0	0	0	0	1	1	0	0	0
05/10/2022	M	37	0	0	1	0	0	0	0	0	0	0	1	1	0	0	0
12/10/2022	F	37	0	0	1	0	0	0	0	0	0	0	1	1	0	0	0

14/02/2023	F	37	0	0	1	0	0	0	0	0	0	0	1	1	0	0	0
09/03/2023	F	37	0	0	1	0	0	0	0	0	0	0	1	1	0	0	0
18/04/2023	M	37	0	0	1	0	0	0	0	0	0	0	1	0	0	0	0
18/08/2023	F	37	0	0	1	0	0	0	0	0	0	0	1	0	0	0	0
31/08/2023	F	37	0	0	1	0	0	0	0	0	0	0	1	1	1	1	0
08/10/2024	M	37	0	0	1	0	0	0	0	0	0	0	1	0	0	0	0
05/01/2022	M	37	0	0	0	0	1	0	0	0	0	0	1	0	1	0	0
24/02/2022	F	37	0	0	0	0	1	0	0	0	0	0	1	1	1	0	0
20/04/2022	F	37	0	0	0	0	1	0	0	0	0	0	1	0	0	0	1
10/05/2022	M	37	1	0	0	0	0	0	0	0	0	0	1	1	1	0	0
26/01/2024	F	37	0	0	0	0	1	0	0	0	0	0	1	0	1	1	0
30/07/2024	M	37	0	0	0	0	1	0	0	0	0	0	1	0	0	0	0
15.12.2021	F	37	0	0	1	0	0	0	0	0	0	1	0	1	0	0	0
04/07/2024	F	37	0	0	0	0	1	0	0	0	1	0	0	0	1	1	0
04/02/2020	M	37	0	0	0	1	1	0	0	0	0	0	1	1	0	0	0
30/09/2020	F	37	0	0	1	0	1	0	0	0	0	0	1	1	0	0	0
26/08/2021	M	37	1	0	1	0	0	0	0	0	0	0	1	1	0	0	0
09/02/2022	M	37	0	0	1	0	1	0	0	0	0	0	1	0	0	0	0
20/04/2022	M	37	0	1	1	0	0	0	0	0	0	0	1	1	0	0	0
29/04/2022	F	37	0	0	1	0	1	0	0	0	0	0	1	0	1	0	1
05/10/2022	M	37	0	1	1	0	0	0	0	0	0	0	1	1	0	0	0
25/10/2023	M	37	0	0	1	0	0	0	1	0	0	0	1	0	0	0	0
12/04/2024	M	37	0	1	1	0	0	0	0	0	0	0	1	1	0	0	0
23/08/2024	M	37	0	1	1	0	0	0	0	0	0	0	1	1	0	0	0
03/09/2024	F	37	1	0	1	0	0	0	0	0	0	0	1	1	0	0	0
11/02/2022	M	37	0	1	0	0	1	0	0	0	0	0	1	1	0	0	0
19/07/2023	M	37	0	1	0	0	0	1	0	0	0	0	1	0	0	0	1
23/04/2024	M	37	1	1	0	0	0	0	0	0	0	0	1	0	0	0	0
30/08/2024	F	37	1	1	0	0	0	0	0	0	0	1	0	0	1	0	1

15/07/2020	M	37	1	1	1	0	0	0	0	0	0	0	0	0	1	0	1
12/11/2020	F	37	0	0	1	1	1	0	0	0	0	0	1	1	1	0	0
22/11/2021	F	37	0	1	1	0	1	0	0	0	0	0	1	1	1	0	0
23/03/2022	M	37	0	0	1	0	1	1	0	0	0	0	1	1	1	0	0
10/05/2022	F	37	0	0	1	0	1	1	0	0	0	0	1	0	1	0	1
16/05/2022	M	37	0	1	1	0	0	1	0	0	0	0	1	1	1	0	0
07/06/2022	F	37	0	1	1	0	1	0	0	0	0	0	1	1	1	0	0
31/10/2022	F	37	0	1	1	1	0	0	0	0	0	0	1	0	0	0	0
27/09/2021	F	37	1	1	0	0	0	0	1	0	1	0	1	1	1	0	1
29/09/2021	M	37	1	0	1	0	0	1	0	0	0	1	1	0	0	0	0
18/09/2020	F	37	0	0	1	1	1	1	0	0	0	0	1	1	0	0	0
13/09/2021	M	37	1	0	1	1	1	0	0	0	0	0	1	1	0	0	0
07/01/2022	F	37	1	1	1	1	0	0	0	0	0	0	1	1	1	0	0
10/01/2022	M	37	1	1	1	1	0	0	0	0	0	0	1	1	0	0	0
05/10/2022	M	37	0	0	1	1	1	0	1	0	0	0	1	0	0	0	0
18/11/2022	M	37	0	1	1	0	1	1	0	0	0	0	1	1	1	0	0
12/04/2023	M	37	0	1	1	1	1	0	0	0	0	0	1	0	0	0	0
31/05/2023	M	37	1	1	1	1	0	0	0	0	0	0	1	0	1	0	0
02/02/2022	F	37	1	1	0	1	1	0	0	0	0	0	1	1	1	0	0
05/02/2020	F	38	0	0	0	0	0	0	0	0	0	0	1	1	0	0	0
11/02/2020	F	38	0	0	0	0	0	0	0	0	0	0	1	0	1	0	0
26/02/2020	F	38	0	0	0	0	0	0	0	0	0	0	0	0	1	0	1
10/06/2020	F	38	0	0	0	0	0	0	0	0	0	0	1	0	0	0	0
09/07/2020	F	38	0	0	0	0	0	0	0	0	0	0	1	0	0	0	0
31/08/2020	F	38	0	0	0	0	0	0	0	0	0	0	0	0	1	0	0
15/09/2020	M	38	0	0	0	0	0	0	0	0	0	0	1	1	0	0	0
11/01/2021	F	38	0	0	0	0	0	0	0	0	0	0	0	0	1	0	0
08/04/2021	M	38	0	0	0	0	0	0	0	0	0	0	0	0	0	0	1
27/09/2021	M	38	0	0	0	0	0	0	0	0	0	0	1	1	0	0	0

24.11.2021	F	38	0	0	0	0	0	0	0	0	0	0	0	0	0	1	0
12/01/2022	F	38	0	0	0	0	0	0	0	0	0	0	0	0	0	0	0
04/02/2022	F	38	0	0	0	0	0	0	0	0	0	0	0	0	0	0	0
06/03/2022	M	38	0	0	0	0	0	0	0	0	0	0	0	0	1	0	0
28/09/2022	F	38	0	0	0	0	0	0	0	0	0	0	0	0	0	0	1
30/09/2022	M	38	0	0	0	0	0	0	0	0	0	0	0	0	0	0	1
30/09/2022	M	38	0	0	0	0	0	0	0	0	0	0	0	0	0	0	1
03/11/2022	F	38	0	0	0	0	0	0	0	0	0	0	0	0	0	0	0
07/12/2022	M	38	0	0	0	0	0	0	0	0	0	0	1	1	0	0	0
06/01/2023	F	38	0	0	0	0	0	0	0	0	0	0	0	0	1	0	0
05/04/2023	M	38	0	0	0	0	0	0	0	0	0	0	0	0	0	0	1
28/04/2023	F	38	0	0	0	0	0	0	0	0	0	0	0	0	0	0	1
24/10/2023	F	38	0	0	0	0	0	0	0	0	0	0	0	0	0	0	0
15/01/2024	F	38	0	0	0	0	0	0	0	0	0	0	0	0	0	0	1
20/03/2024	F	38	0	0	0	0	0	0	0	0	0	0	0	0	0	0	0
28/03/2024	F	38	0	0	0	0	0	0	0	0	0	0	1	1	0	0	0
29/03/2024	F	38	0	0	0	0	0	0	0	0	0	0	0	0	0	0	0
29/03/2024	F	38	0	0	0	0	0	0	0	0	0	0	0	0	0	0	0
10/07/2024	M	38	0	0	0	0	0	0	0	0	0	0	0	0	0	0	1
28/08/2024	M	38	0	0	0	0	0	0	0	0	0	0	1	0	0	0	0
17/09/2024	F	38	0	0	0	0	0	0	0	0	0	0	0	1	0	0	1
18/09/2024	F	38	0	0	0	0	0	0	0	0	0	0	0	0	0	0	0
26/11/2024	F	38	0	0	0	0	0	0	0	0	0	0	0	0	0	0	0
03/12/2024	F	38	0	0	0	0	0	0	0	0	0	0	0	0	0	0	1
03/09/2021	M	38	0	0	0	0	0	0	0	0	0	1	1	0	0	0	0
05.11.2021	F	38	0	0	0	0	0	0	0	0	0	1	0	0	0	0	0
20.10.2021	M	38	0	0	0	0	0	0	0	0	0	1	1	0	0	0	0
31/01/2020	F	38	0	0	0	0	1	0	0	0	0	0	1	0	1	0	0
27/07/2020	M	38	0	0	1	0	0	0	0	0	0	0	1	1	1	0	0

08/09/2020	M	38	0	0	1	0	0	0	0	0	0	0	1	1	0	0	0
14/12/2021	F	38	0	0	1	0	0	0	0	0	0	0	1	1	0	0	0
08/03/2022	M	38	0	0	1	0	0	0	0	0	0	0	1	1	0	0	0
06/09/2022	F	38	0	0	1	0	0	0	0	0	0	0	1	1	1	1	0
24/11/2022	F	38	0	0	1	0	0	0	0	0	0	0	1	1	0	0	0
11/08/2023	M	38	0	0	1	0	0	0	0	0	0	0	1	0	0	0	0
23/08/2023	M	38	0	0	1	0	0	0	0	0	0	0	1	0	0	0	0
23/08/2023	F	38	0	0	1	0	0	0	0	0	0	0	1	0	0	0	0
19/09/2023	F	38	0	0	1	0	0	0	0	0	0	0	1	1	1	0	0
20/09/2023	F	38	0	0	1	0	0	0	0	0	0	0	1	0	1	0	0
08/11/2023	M	38	0	0	1	0	0	0	0	0	0	0	1	0	0	0	0
08/02/2024	M	38	0	0	1	0	0	0	0	0	0	0	1	1	0	0	0
16/02/2024	M	38	0	0	1	0	0	0	0	0	0	0	1	1	0	0	1
21/02/2024	M	38	0	0	1	0	0	0	0	0	0	0	1	1	0	0	0
01/07/2024	M	38	0	0	1	0	0	0	0	0	0	0	1	1	0	1	1
19/01/2022	F	38	0	1	0	0	0	0	0	0	0	0	0	0	0	0	0
28/04/2022	M	38	0	0	0	0	1	0	0	0	0	0	1	0	1	0	1
29/03/2023	F	38	0	0	0	0	0	1	0	0	0	0	1	0	1	0	0
16/06/2023	M	38	1	0	0	0	0	0	0	0	0	0	1	0	0	0	1
30/04/2024	F	38	1	0	0	0	0	0	0	0	0	0	0	0	1	0	0
04/11/2024	F	38	0	0	0	0	1	0	0	0	0	0	1	0	1	0	0
15/10/2021	M	38	0	0	1	0	0	0	0	0	0	1	0	0	0	1	0
03.12.2021	M	38	0	0	1	0	0	0	0	0	0	1	1	1	0	0	0
14/12/2022	F	38	0	0	0	0	1	0	0	0	0	1	0	0	0	0	1
20/05/2021	F	38	0	0	0	1	1	0	0	0	0	0	1	1	1	0	0
22/01/2020	M	38	0	1	1	0	0	0	0	0	0	0	1	0	0	0	1
01/09/2020	F	38	0	0	1	0	0	1	0	0	0	0	1	1	0	0	0
09/09/2020	M	38	0	0	1	0	0	1	0	0	0	0	1	1	0	0	0
14/11/2022	F	38	0	1	1	0	0	0	0	0	0	0	1	0	1	0	0

24/04/2023	M	38	0	1	1	0	0	0	0	0	0	0	1	1	0	0	0
17.02.2021	F	38	0	0	1	1	0	0	0	0	0	0	1	1	0	0	0
05/10/2023	M	38	0	0	0	0	1	0	1	0	0	0	1	0	0	0	1
02/04/2024	F	38	1	0	0	0	1	0	0	0	0	0	1	0	1	0	0
17/01/2020	M	38	0	0	0	0	1	1	1	0	0	0	1	0	1	0	0
09/10/2020	F	38	1	1	0	1	0	0	0	0	0	0	1	0	1	0	0
17/02/2020	M	38	0	1	1	0	1	0	0	0	0	0	1	1	1	0	0
09/07/2020	M	38	0	1	1	0	1	0	0	0	0	0	1	1	0	0	0
02/12/2020	F	38	0	1	1	0	1	0	0	0	0	0	1	1	1	0	0
04/10/2021	F	38	0	0	1	0	1	1	0	0	0	0	1	0	1	0	0
21/02/2022	F	38	0	1	1	0	1	0	0	0	0	0	1	1	1	0	0
09/09/2022	F	38	1	1	1	0	0	0	0	0	0	0	1	1	1	0	0
02/07/2024	M	38	1	1	1	0	0	0	0	0	0	0	1	1	0	0	0
23/10/2024	M	38	1	1	1	0	0	0	0	0	0	0	1	1	0	0	0
16/08/2023	F	38	1	1	1	0	0	0	0	1	0	0	1	0	1	0	0
10/06/2020	F	38	0	1	1	0	1	1	0	0	0	0	1	1	0	0	0
08/01/2020	F	39	0	0	0	0	0	0	0	0	0	0	0	0	0	0	1
12/03/2020	M	39	0	0	0	0	0	0	0	0	0	0	1	0	0	0	0
17/06/2020	F	39	0	0	0	0	0	0	0	0	0	0	0	0	0	0	0
22/02/2021	F	39	0	0	0	0	0	0	0	0	0	0	1	0	0	0	0
22/03/2021	M	39	0	0	0	0	0	0	0	0	0	0	0	0	0	0	1
26/08/2021	M	39	0	0	0	0	0	0	0	0	0	0	0	0	0	0	1
30/08/2021	F	39	0	0	0	0	0	0	0	0	0	0	0	0	0	0	0
10/09/2021	F	39	0	0	0	0	0	0	0	0	0	0	0	0	0	1	0
21/10/2021	F	39	0	0	0	0	0	0	0	0	0	0	1	1	0	0	0
24.02.2021	F	39	0	0	0	0	0	0	0	0	0	0	1	0	0	0	0
12/01/2022	M	39	0	0	0	0	0	0	0	0	0	0	0	0	0	0	0
25/01/2022	M	39	0	0	0	0	0	0	0	0	0	0	0	0	1	0	0
28/01/2022	M	39	0	0	0	0	0	0	0	0	0	0	0	0	0	0	0

07/02/2022	M	39	0	0	0	0	0	0	0	0	0	0	0	0	0	0	1
11/03/2022	F	39	0	0	0	0	0	0	0	0	0	0	1	0	0	0	0
31/03/2022	F	39	0	0	0	0	0	0	0	0	0	0	0	0	0	0	0
22/06/2022	F	39	0	0	0	0	0	0	0	0	0	0	1	0	0	0	0
27/06/2022	M	39	0	0	0	0	0	0	0	0	0	0	1	0	0	0	0
31/08/2022	M	39	0	0	0	0	0	0	0	0	0	0	1	0	1	0	0
02/11/2022	F	39	0	0	0	0	0	0	0	0	0	0	1	0	1	0	0
08/11/2022	F	39	0	0	0	0	0	0	0	0	0	0	1	0	0	0	0
25/08/2023	F	39	0	0	0	0	0	0	0	0	0	0	1	0	0	0	0
05/09/2023	M	39	0	0	0	0	0	0	0	0	0	0	0	0	0	0	1
13/10/2023	M	39	0	0	0	0	0	0	0	0	0	0	0	0	1	0	0
01/03/2024	F	39	0	0	0	0	0	0	0	0	0	0	1	1	0	0	0
06/03/2024	F	39	0	0	0	0	0	0	0	0	0	0	0	0	0	0	0
22/03/2024	M	39	0	0	0	0	0	0	0	0	0	0	0	0	0	0	0
02/04/2024	M	39	0	0	0	0	0	0	0	0	0	0	0	0	0	0	1
12/04/2024	M	39	0	0	0	0	0	0	0	0	0	0	0	0	0	0	1
25/06/2024	F	39	0	0	0	0	0	0	0	0	0	0	0	0	0	0	1
05/07/2024	F	39	0	0	0	0	0	0	0	0	0	0	0	0	0	0	0
29/11/2024	F	39	0	0	0	0	0	0	0	0	0	0	0	0	0	0	0
14/12/2022	F	39	0	0	0	0	0	0	0	0	1	0	0	0	0	0	0
24.03.2021	M	39	0	0	0	0	1	0	0	0	0	0	1	0	0	0	1
16/06/2020	F	39	0	0	1	0	0	0	0	0	0	0	1	1	0	0	0
27/07/2020	M	39	0	0	1	0	0	0	0	0	0	0	1	1	1	0	0
12/08/2020	F	39	0	0	1	0	0	0	0	0	0	0	1	0	1	0	0
31/08/2020	M	39	0	0	1	0	0	0	0	0	0	0	1	1	0	0	0
05/10/2022	F	39	0	0	1	0	0	0	0	0	0	0	1	0	0	0	0
25/09/2023	M	39	0	0	1	0	0	0	0	0	0	0	1	1	0	0	0
05/10/2023	F	39	0	0	1	0	0	0	0	0	0	0	1	1	1	0	0
03/11/2023	F	39	0	0	1	0	0	0	0	0	0	0	1	0	0	0	0

27/02/2024	F	39	0	0	1	0	0	0	0	0	0	0	1	1	0	0	0
06/03/2022	M	39	1	0	0	0	0	0	0	0	0	0	1	1	0	0	0
15/02/2023	F	39	0	1	0	0	0	0	0	0	0	0	1	0	1	0	0
27/09/2023	F	39	0	0	0	0	1	0	0	0	0	0	1	0	1	0	0
28.05.2021	F	39	0	0	1	0	0	0	0	0	0	1	0	0	0	0	0
07/02/2024	F	39	0	0	0	0	1	0	0	1	1	0	0	0	1	0	0
09/06/2020	F	39	0	0	1	0	1	0	0	0	0	0	1	1	0	0	0
08/10/2020	M	39	0	0	1	0	0	1	0	0	0	0	1	0	1	0	0
05/08/2021	M	39	0	0	1	1	0	0	0	0	0	0	1	1	1	0	0
13/09/2021	F	39	0	0	1	0	1	0	0	0	0	0	1	1	0	0	0
24/05/2022	F	39	0	0	1	0	0	1	0	0	0	0	1	1	0	0	0
26/07/2023	M	39	0	0	1	1	0	0	0	0	0	0	1	0	0	0	0
12/04/2024	M	39	0	0	1	0	0	1	0	0	0	0	1	1	0	0	0
30/04/2024	M	39	0	0	1	1	0	0	0	0	0	0	1	1	1	0	0
20/12/2024	F	39	0	0	1	0	1	0	0	0	0	0	1	1	0	0	0
08/02/2023	F	39	0	0	0	0	1	0	1	0	0	0	1	0	0	0	1
31/08/2023	M	39	1	1	0	0	0	0	0	0	0	0	1	1	0	0	0
07/09/2021	F	39	1	0	1	0	0	0	0	1	0	0	1	1	0	0	0
14/01/2020	F	39	1	1	0	0	0	1	0	0	0	0	1	1	1	0	0
20/11/2020	M	39	1	1	1	0	0	0	0	0	0	0	1	1	0	0	0
15/02/2021	F	39	0	0	1	0	1	1	0	0	0	0	1	0	1	0	0
31/08/2021	M	39	0	0	1	1	0	1	0	0	0	0	1	0	0	0	0
19/07/2023	M	39	1	0	1	0	0	1	0	0	0	0	1	0	1	0	0
03.12.2021	F	39	0	1	1	1	0	0	0	0	0	1	1	0	0	0	0
13/04/2021	M	39	1	1	0	0	1	1	0	0	0	0	1	0	0	0	1
17/02/2020	F	39	0	0	1	1	1	0	1	0	0	0	1	0	1	0	0
24/07/2023	M	39	0	1	1	1	1	0	0	0	0	0	1	1	0	0	0
07/12/2023	M	39	1	1	1	1	0	0	0	0	0	0	1	1	1	0	0
10/05/2023	M	39	1	1	1	1	0	1	0	0	0	0	1	0	0	0	0

28.04.2021	M	39	1	1	1	1	0	1	0	0	0	1	0	0	0	1	0
02/07/2020	F	39	1	1	1	1	1	1	0	0	0	0	1	1	0	0	0
04/03/2020	M	40	0	0	0	0	0	0	0	0	0	0	0	0	0	0	1
28/05/2020	M	40	0	0	0	0	0	0	0	0	0	0	1	0	0	0	0
26/06/2020	F	40	0	0	0	0	0	0	0	0	0	0	0	0	0	0	1
16/09/2020	F	40	0	0	0	0	0	0	0	0	0	0	1	1	0	0	0
14/10/2020	M	40	0	0	0	0	0	0	0	0	0	0	0	0	0	0	0
27/10/2020	M	40	0	0	0	0	0	0	0	0	0	0	1	0	0	0	0
27/10/2020	M	40	0	0	0	0	0	0	0	0	0	0	0	0	0	0	0
25/11/2020	M	40	0	0	0	0	0	0	0	0	0	0	1	0	0	0	0
02/02/2021	F	40	0	0	0	0	0	0	0	0	0	0	1	0	0	0	0
11/02/2021	F	40	0	0	0	0	0	0	0	0	0	0	0	0	0	0	1
13/07/2021	F	40	0	0	0	0	0	0	0	0	0	0	1	1	1	0	0
26/07/2021	F	40	0	0	0	0	0	0	0	0	0	0	0	0	0	0	1
26/08/2021	M	40	0	0	0	0	0	0	0	0	0	0	0	0	0	0	0
13/09/2021	F	40	0	0	0	0	0	0	0	0	0	0	0	0	0	0	0
20/10/2021	F	40	0	0	0	0	0	0	0	0	0	0	1	0	0	0	0
03.02.2021	M	40	0	0	0	0	0	0	0	0	0	0	1	0	1	0	0
03.11.2021	F	40	0	0	0	0	0	0	0	0	0	0	0	0	0	0	0
08.01.2021	M	40	0	0	0	0	0	0	0	0	0	0	1	0	0	0	0
10.09.2021	F	40	0	0	0	0	0	0	0	0	0	0	0	0	0	0	0
27.01.2021	F	40	0	0	0	0	0	0	0	0	0	0	0	0	0	0	1
14/01/2022	M	40	0	0	0	0	0	0	0	0	0	0	1	0	0	0	0
20/01/2022	M	40	0	0	0	0	0	0	0	0	0	0	1	0	0	0	0
01/02/2022	M	40	0	0	0	0	0	0	0	0	0	0	1	0	0	0	0
16/02/2022	M	40	0	0	0	0	0	0	0	0	0	0	0	0	0	0	1
12/04/2022	M	40	0	0	0	0	0	0	0	0	0	0	1	1	0	0	0
15/04/2022	M	40	0	0	0	0	0	0	0	0	0	0	1	1	0	0	0
26/05/2022	F	40	0	0	0	0	0	0	0	0	0	0	1	0	0	0	1

08/06/2022	F	40	0	0	0	0	0	0	0	0	0	0	0	0	0	0	1
18/07/2022	F	40	0	0	0	0	0	0	0	0	0	0	0	0	0	0	1
01/08/2022	F	40	0	0	0	0	0	0	0	0	0	0	1	0	0	0	0
25/10/2022	F	40	0	0	0	0	0	0	0	0	0	0	0	0	0	0	1
26/10/2022	F	40	0	0	0	0	0	0	0	0	0	0	0	0	0	0	0
28/03/2023	M	40	0	0	0	0	0	0	0	0	0	0	0	0	0	0	0
12/05/2023	F	40	0	0	0	0	0	0	0	0	0	0	0	0	0	0	1
12/05/2023	F	40	0	0	0	0	0	0	0	0	0	0	1	0	1	0	0
11/08/2023	F	40	0	0	0	0	0	0	0	0	0	0	1	0	0	0	1
13/10/2023	F	40	0	0	0	0	0	0	0	0	0	0	1	0	0	0	1
21/02/2024	M	40	0	0	0	0	0	0	0	0	0	0	0	0	0	0	1
29/03/2024	F	40	0	0	0	0	0	0	0	0	0	0	0	0	0	0	0
12/04/2024	F	40	0	0	0	0	0	0	0	0	0	0	0	0	0	0	0
25/06/2024	M	40	0	0	0	0	0	0	0	0	0	0	0	0	0	0	1
23/10/2024	F	40	0	0	0	0	0	0	0	0	0	0	0	0	1	0	0
05/11/2024	M	40	0	0	0	0	0	0	0	0	0	0	1	0	1	0	0
04/12/2024	F	40	0	0	0	0	0	0	0	0	0	0	0	0	0	0	1
03.03.2021	F	40	0	0	0	0	0	0	0	0	1	0	0	0	0	0	0
15.09.2021	M	40	0	0	0	0	0	0	0	0	0	1	1	0	0	0	0
31/08/2020	M	40	0	0	0	0	0	0	1	0	0	0	1	0	1	0	0
22/06/2021	M	40	0	0	0	0	0	0	1	0	0	0	1	0	0	0	0
24.03.2021	M	40	0	0	0	0	0	0	1	0	0	0	1	0	0	0	0
17/06/2020	M	40	0	0	1	0	0	0	0	0	0	0	1	1	0	0	0
09/07/2020	M	40	0	0	1	0	0	0	0	0	0	0	1	1	1	0	0
08/09/2020	M	40	0	0	1	0	0	0	0	0	0	0	1	1	1	0	0
20/05/2021	M	40	0	0	1	0	0	0	0	0	0	0	1	1	1	0	0
04/09/2023	M	40	0	0	1	0	0	0	0	0	0	0	1	1	0	0	0
24/11/2023	M	40	0	0	1	0	0	0	0	0	0	0	1	1	0	0	0
21/09/2023	M	40	0	1	0	0	0	0	0	0	0	0	0	0	1	0	0

08/05/2024	F	40	1	0	0	0	0	0	0	0	0	0	1	1	0	0	1
09/05/2024	M	40	0	0	0	0	1	0	0	0	0	0	1	0	0	0	0
22/09/2021	M	40	0	0	1	0	0	0	0	0	0	1	1	0	0	0	0
27.10.2021	F	40	0	0	1	0	0	0	0	0	0	1	1	0	0	0	0
30/10/2024	M	40	0	0	1	0	0	0	0	0	1	1	1	1	0	0	1
17/06/2020	M	40	1	0	0	0	1	0	0	0	0	0	1	1	0	0	0
20/10/2021	F	40	0	1	0	0	1	0	0	0	0	0	1	0	0	0	1
10/08/2020	M	40	0	0	1	1	0	0	0	0	0	0	1	1	0	0	0
17/05/2021	M	40	0	0	1	0	0	1	0	0	0	0	1	1	1	0	0
02/11/2022	M	40	0	0	1	0	1	0	0	0	0	0	1	0	0	0	0
07/12/2022	M	40	0	1	1	0	0	0	0	0	0	0	1	0	0	0	0
13/10/2023	M	40	1	0	1	0	0	0	0	0	0	0	1	0	0	0	0
12/04/2024	F	40	1	0	1	0	0	0	0	0	0	0	1	1	0	0	0
29/10/2024	F	40	0	0	1	1	0	0	0	0	0	0	1	1	0	0	0
04/01/2023	M	40	0	0	0	0	1	1	0	0	0	0	1	0	0	0	1
06/01/2023	M	40	0	1	0	0	1	0	0	0	0	0	1	0	0	0	0
26/07/2023	F	40	1	1	0	0	0	0	0	0	0	0	1	0	1	0	0
15.09.2021	F	40	0	0	0	0	1	1	0	0	0	1	1	0	0	0	0
08/09/2021	M	40	0	0	1	1	0	0	0	0	0	1	1	0	0	0	0
10/06/2021	F	40	0	0	1	0	1	0	0	0	1	1	1	0	0	0	0
12/04/2024	F	40	1	1	1	0	0	0	0	0	0	0	1	1	0	0	0
18/09/2024	M	40	0	0	1	1	1	0	0	0	0	0	1	1	1	0	0
16/07/2024	F	40	1	1	0	0	0	1	0	0	0	0	1	0	0	0	1
27/09/2023	F	40	0	1	1	0	0	1	1	0	0	0	1	0	0	0	0
04/10/2022	F	40	1	0	0	0	1	1	1	0	0	0	1	0	1	0	0
03.11.2021	M	40	0	1	1	1	1	0	0	0	0	1	1	1	0	0	0
21.12.2021	M	40	0	1	1	1	0	1	1	0	0	1	0	0	0	0	0
17/12/2020	F	40	1	1	1	1	1	1	1	0	0	0	1	1	1	0	0
08/01/2020	F	41	0	0	0	0	0	0	0	0	0	0	1	0	0	0	0

04/02/2020	M	41	0	0	0	0	0	0	0	0	0	0	1	1	0	0	0
14/02/2020	M	41	0	0	0	0	0	0	0	0	0	0	0	0	0	0	0
02/06/2020	M	41	0	0	0	0	0	0	0	0	0	0	0	0	1	0	0
03/07/2020	F	41	0	0	0	0	0	0	0	0	0	0	0	0	0	0	1
07/07/2020	F	41	0	0	0	0	0	0	0	0	0	0	1	0	0	0	1
07/12/2020	F	41	0	0	0	0	0	0	0	0	0	0	0	0	0	0	1
25/05/2021	F	41	0	0	0	0	0	0	0	0	0	0	1	1	0	0	0
06/09/2021	F	41	0	0	0	0	0	0	0	0	0	0	1	1	0	0	0
20/09/2021	F	41	0	0	0	0	0	0	0	0	0	0	0	0	0	0	0
20/12/2021	F	41	0	0	0	0	0	0	0	0	0	0	1	0	0	0	0
31.03.2021	M	41	0	0	0	0	0	0	0	0	0	0	0	0	0	0	0
06/01/2022	F	41	0	0	0	0	0	0	0	0	0	0	1	0	1	0	0
26/01/2022	M	41	0	0	0	0	0	0	0	0	0	0	0	0	0	0	0
08/03/2022	F	41	0	0	0	0	0	0	0	0	0	0	0	0	0	0	1
09/03/2022	M	41	0	0	0	0	0	0	0	0	0	0	0	0	0	0	0
17/05/2022	F	41	0	0	0	0	0	0	0	0	0	0	1	0	1	0	0
05/09/2022	F	41	0	0	0	0	0	0	0	0	0	0	0	0	0	0	0
19/09/2022	F	41	0	0	0	0	0	0	0	0	0	0	0	**0**	0	0	1
30/09/2022	F	41	0	0	0	0	0	0	0	0	0	0	1	0	0	0	0
14/10/2022	M	41	0	0	0	0	0	0	0	0	0	0	0	0	1	0	0
16/01/2023	M	41	0	0	0	0	0	0	0	0	0	0	0	0	1	0	0
03/03/2023	F	41	0	0	0	0	0	0	0	0	0	0	1	0	0	0	1
05/04/2023	F	41	0	0	0	0	0	0	0	0	0	0	1	0	1	0	0
09/05/2023	M	41	0	0	0	0	0	0	0	0	0	0	1	0	0	0	0
30/05/2023	M	41	0	0	0	0	0	0	0	0	0	0	1	0	0	0	0
16/08/2023	F	41	0	0	0	0	0	0	0	0	0	0	1	0	0	0	1
18/08/2023	F	41	0	0	0	0	0	0	0	0	0	0	1	0	1	0	0
12/12/2023	F	41	0	0	0	0	0	0	0	0	0	0	0	0	0	0	1
10/01/2024	M	41	0	0	0	0	0	0	0	0	0	0	1	0	0	0	0

08/04/2024	M	41	0	0	0	0	0	0	0	0	0	0	0	0	0	0	0
24/04/2024	F	41	0	0	0	0	0	0	0	0	0	0	0	0	0	0	0
09/05/2024	F	41	0	0	0	0	0	0	0	0	0	0	0	0	0	0	0
20/05/2024	M	41	0	0	0	0	0	0	0	0	0	0	1	0	0	0	0
22/05/2024	M	41	0	0	0	0	0	0	0	0	0	0	0	0	0	0	1
19/06/2024	M	41	0	0	0	0	0	0	0	0	0	0	1	0	0	0	0
10/07/2024	F	41	0	0	0	0	0	0	0	0	0	0	0	0	0	0	1
03/12/2024	M	41	0	0	0	0	0	0	0	0	0	0	0	0	0	0	0
17/12/2024	M	41	0	0	0	0	0	0	0	0	0	0	0	0	0	0	1
14/12/2021	F	41	0	0	0	0	0	0	0	0	1	0	0	0	1	0	1
03.09.2021	M	41	0	0	0	0	0	0	0	0	0	1	1	0	0	0	0
21.12.2021	M	41	0	0	0	0	0	0	0	0	0	1	0	0	0	0	0
27.10.2021	F	41	0	0	0	0	0	0	0	0	0	1	0	0	0	1	0
28.04.2021	F	41	0	0	0	0	0	0	0	0	1	0	0	0	0	1	0
08/08/2023	F	41	0	0	0	0	0	0	0	0	0	1	1	0	0	0	0
14/01/2020	F	41	0	0	1	0	0	0	0	0	0	0	1	1	1	0	0
22/01/2020	F	41	0	0	1	0	0	0	0	0	0	0	1	1	1	0	0
05/02/2020	M	41	0	0	1	0	0	0	0	0	0	0	1	1	0	0	0
04/04/2021	M	41	0	0	1	0	0	0	0	0	0	0	0	0	0	0	1
06/05/2021	M	41	0	0	1	0	0	0	0	0	0	0	1	1	1	0	0
31/08/2022	M	41	0	0	1	0	0	0	0	0	0	0	1	0	0	0	0
17/02/2023	M	41	0	0	1	0	0	0	0	0	0	0	1	0	0	0	1
17/08/2023	M	41	0	0	1	0	0	0	0	0	0	0	1	1	0	0	0
18/09/2023	M	41	0	0	1	0	0	0	0	0	0	0	1	1	0	0	0
09/01/2024	M	41	0	0	1	0	0	0	0	0	0	0	1	1	0	0	0
06.01.2021	F	41	0	0	1	0	0	0	0	0	0	0	1	0	1	0	0
17.02.2021	M	41	0	0	1	0	0	0	0	0	0	0	1	1	0	0	0
14/01/2020	M	41	0	0	1	0	0	1	0	0	0	0	1	0	0	0	0
13/09/2021	M	41	0	0	1	0	1	0	0	0	0	0	1	1	0	0	0

27/04/2022	F	41	0	1	1	0	0	0	0	0	0	0	1	1	0	0	0
20/11/2024	M	41	0	1	1	0	0	0	0	0	0	0	1	1	1	0	0
30/05/2022	M	41	0	1	0	0	1	0	0	0	0	0	1	1	0	0	0
13/11/2023	F	41	0	1	0	0	1	0	0	0	0	0	1	1	1	0	0
08/10/2024	M	41	0	1	1	0	0	0	0	1	0	0	1	1	1	0	0
17.12.2021	M	41	0	0	1	0	1	0	0	0	0	1	1	1	0	0	0
21.05.2021	M	41	0	0	1	0	1	0	0	0	0	1	0	0	0	0	0
22.09.2021	F	41	0	0	1	0	0	1	0	0	0	1	1	1	0	0	0
17/02/2020	M	41	0	1	1	0	1	0	0	0	0	0	1	0	0	0	0
14/06/2021	F	41	0	1	1	0	0	0	1	0	0	0	1	1	0	0	0
13/09/2021	M	41	0	0	1	1	1	0	0	0	0	0	1	1	0	0	0
30/08/2022	F	41	0	1	1	0	1	0	0	0	0	0	1	1	0	0	0
16/09/2022	M	41	0	0	1	1	1	0	0	0	0	0	1	0	1	0	0
27/06/2024	F	41	1	1	1	0	0	0	0	0	0	0	1	1	0	0	0
03/07/2024	F	41	1	1	1	0	0	0	0	0	0	0	1	1	0	0	0
23/05/2022	F	41	1	0	0	0	1	1	0	0	0	0	1	0	0	0	1
26/07/2023	M	41	0	1	0	0	1	1	0	0	0	0	1	0	0	0	0
03/02/2021	M	41	1	1	0	1	0	0	0	0	1	0	1	0	1	0	0
28/07/2023	F	41	0	1	1	0	0	1	0	1	0	0	1	0	0	0	1
08.09.2021	M	41	0	0	1	0	1	1	0	0	0	1	0	0	0	0	0
04/02/2020	M	41	0	1	0	0	1	1	1	0	0	0	1	0	1	0	0
13/10/2021	M	41	1	1	1	1	1	1	0	0	0	1	1	1	0	0	0
23/01/2020	F	42	0	0	0	0	0	0	0	0	0	0	0	0	1	0	1
13/02/2020	M	42	0	0	0	0	0	0	0	0	0	0	0	0	0	0	0
13/02/2020	F	42	0	0	0	0	0	0	0	0	0	0	1	0	0	0	1
26/02/2020	M	42	0	0	0	0	0	0	0	0	0	0	1	0	0	0	0
23/03/2020	M	42	0	0	0	0	0	0	0	0	0	0	1	0	0	0	0
18/06/2020	F	42	0	0	0	0	0	0	0	0	0	0	1	0	1	0	0
07/07/2020	F	42	0	0	0	0	0	0	0	0	0	0	1	1	1	0	0

13/07/2020	F	42	0	0	0	0	0	0	0	0	0	0	0	0	0	0	1
16/07/2020	F	42	0	0	0	0	0	0	0	0	0	0	1	1	0	0	0
16/11/2020	F	42	0	0	0	0	0	0	0	0	0	0	0	0	1	0	0
26/11/2020	M	42	0	0	0	0	0	0	0	0	0	0	1	0	0	0	0
25/03/2021	F	42	0	0	0	0	0	0	0	0	0	0	1	0	0	0	0
12/04/2021	F	42	0	0	0	0	0	0	0	0	0	0	0	0	0	0	1
20/07/2021	F	42	0	0	0	0	0	0	0	0	0	0	0	0	1	0	0
23/08/2021	M	42	0	0	0	0	0	0	0	0	0	0	1	0	0	0	0
02/11/2021	F	42	0	0	0	0	0	0	0	0	0	0	0	0	0	0	1
05/01/2022	F	42	0	0	0	0	0	0	0	0	0	0	1	0	0	0	0
07/01/2022	F	42	0	0	0	0	0	0	0	0	0	0	0	0	0	0	1
25/03/2022	M	42	0	0	0	0	0	0	0	0	0	0	0	0	0	0	1
06/06/2022	F	42	0	0	0	0	0	0	0	0	0	0	0	0	0	0	1
27/09/2022	M	42	0	0	0	0	0	0	0	0	0	0	0	0	0	0	0
26/10/2022	F	42	0	0	0	0	0	0	0	0	0	0	0	0	0	0	0
23/02/2023	F	42	0	0	0	0	0	0	0	0	0	0	0	0	0	0	0
31/05/2023	M	42	0	0	0	0	0	0	0	0	0	0	1	0	1	0	0
28/06/2023	F	42	0	0	0	0	0	0	0	0	0	0	1	0	0	0	0
08/08/2023	F	42	0	0	0	0	0	0	0	0	0	0	1	0	0	0	0
11/08/2023	F	42	0	0	0	0	0	0	0	0	0	0	0	0	0	0	1
25/10/2023	F	42	0	0	0	0	0	0	0	0	0	0	0	0	0	0	1
06/11/2023	F	42	0	0	0	0	0	0	0	0	0	0	0	0	0	0	0
13/03/2024	F	42	0	0	0	0	0	0	0	0	0	0	0	0	0	0	0
19/04/2024	M	42	0	0	0	0	0	0	0	0	0	0	0	0	0	0	1
07/05/2024	F	42	0	0	0	0	0	0	0	0	0	0	0	0	0	0	1
17/07/2024	F	42	0	0	0	0	0	0	0	0	0	0	0	0	0	0	1
31/07/2024	F	42	0	0	0	0	0	0	0	0	0	0	0	0	1	0	0
16/10/2024	M	42	0	0	0	0	0	0	0	0	0	0	0	0	0	0	1
29/10/2024	M	42	0	0	0	0	0	0	0	0	0	0	0	0	0	0	1

13/11/2024	F	42	0	0	0	0	0	0	0	0	0	0	0	0	0	0	1
20/01/2020	M	42	0	0	0	0	0	0	0	0	1	0	0	0	0	0	0
31/08/2020	F	42	0	0	0	0	0	0	0	1	0	0	0	0	0	0	0
08/03/2021	F	42	0	0	0	0	0	0	0	0	1	0	0	0	0	0	0
07.04.2021	M	42	0	0	0	0	0	0	0	0	0	1	0	0	0	1	0
09/09/2022	F	42	0	0	0	0	0	0	0	1	0	0	1	1	0	0	0
24/06/2022	F	42	0	0	0	0	0	0	0	0	1	1	0	0	0	0	1
21/02/2020	M	42	0	0	0	0	0	0	1	0	0	0	1	0	0	0	0
14/12/2021	F	42	0	0	0	0	1	0	0	0	0	0	1	0	0	0	0
03/03/2020	M	42	0	0	1	0	0	0	0	0	0	0	1	1	1	0	0
21/09/2020	F	42	0	0	1	0	0	0	0	0	0	0	1	1	0	0	0
20/07/2022	F	42	0	0	1	0	0	0	0	0	0	0	1	0	0	0	0
25/08/2022	M	42	0	0	1	0	0	0	0	0	0	0	1	1	0	0	0
30/08/2022	M	42	0	0	1	0	0	0	0	0	0	0	1	0	0	0	0
02/08/2023	F	42	0	0	1	0	0	0	0	0	0	0	1	0	0	0	0
07/09/2023	M	42	0	0	1	0	0	0	0	0	0	0	1	0	0	0	0
27/09/2023	F	42	0	0	1	0	0	0	0	0	0	0	1	0	0	0	0
16/11/2023	M	42	0	0	1	0	0	0	0	0	0	0	1	1	0	0	0
21/08/2024	F	42	0	0	1	0	0	0	0	0	0	0	1	1	0	0	0
24.02.2021	M	42	0	0	1	0	0	0	0	0	0	0	1	0	1	0	1
24/06/2022	F	42	0	0	0	0	0	0	1	0	0	0	0	0	0	0	0
29/06/2022	F	42	0	0	0	0	0	0	1	0	0	0	1	0	0	0	0
27/10/2023	F	42	0	1	0	0	0	0	0	0	0	0	1	0	0	0	0
11/12/2023	M	42	0	0	0	0	1	0	0	0	0	0	0	0	1	0	1
29/09/2021	M	42	0	0	1	0	0	0	0	0	0	1	1	0	0	0	0
19.11.2021	F	42	0	0	1	0	0	0	0	0	0	1	0	1	0	0	0
08/05/2023	F	42	0	0	0	0	0	1	0	0	1	0	1	0	0	0	1
25/11/2021	F	42	1	1	0	0	0	0	0	0	0	0	0	1	1	0	1
29/06/2020	F	42	0	0	1	0	1	0	0	0	0	0	1	1	0	0	0

29/04/2021	M	42	0	0	1	0	1	0	0	0	0	0	1	1	0	0	0
02/09/2022	M	42	0	1	1	0	0	0	0	0	0	0	1	0	0	0	0
01/03/2022	F	42	0	0	0	0	0	1	1	0	0	0	1	0	0	0	1
09.06.2021	M	42	0	1	0	0	1	0	0	0	1	1	0	0	0	0	1
10/10/2024	F	42	1	0	1	0	0	0	0	0	1	1	1	1	1	0	0
04/02/2020	M	42	1	1	0	0	1	0	0	0	0	0	1	1	0	0	0
29/11/2021	F	42	0	1	0	0	1	1	0	0	0	0	1	0	1	0	0
26/06/2023	M	42	0	1	1	0	0	0	1	0	0	0	1	0	1	0	1
18/09/2024	F	42	0	1	1	1	0	0	0	0	0	0	1	1	0	0	0
18/09/2024	F	42	1	0	1	1	0	0	0	0	0	0	1	0	0	0	0
14/07/2020	F	42	1	0	0	1	1	0	0	0	0	1	1	1	0	0	0
28/07/2023	M	42	1	1	1	1	0	0	1	0	0	0	1	0	0	0	0
02/10/2024	M	42	1	1	1	1	0	1	0	1	0	0	1	1	0	0	0
09/01/2020	M	43	0	0	0	0	0	0	0	0	0	0	0	0	0	0	0
13/01/2020	F	43	0	0	0	0	0	0	0	0	0	0	1	0	0	0	0
28/01/2020	F	43	0	0	0	0	0	0	0	0	0	0	0	0	0	0	0
12/02/2020	F	43	0	0	0	0	0	0	0	0	0	0	0	1	0	0	0
02/03/2020	F	43	0	0	0	0	0	0	0	0	0	0	1	0	0	0	0
01/07/2020	F	43	0	0	0	0	0	0	0	0	0	0	0	0	0	0	0
15/07/2020	F	43	0	0	0	0	0	0	0	0	0	0	0	0	0	0	0
20/07/2020	M	43	0	0	0	0	0	0	0	0	0	0	0	0	0	0	1
06/08/2020	F	43	0	0	0	0	0	0	0	0	0	0	0	0	0	0	1
11/08/2020	F	43	0	0	0	0	0	0	0	0	0	0	0	0	1	0	0
18/09/2020	F	43	0	0	0	0	0	0	0	0	0	0	1	0	0	0	0
09/10/2020	F	43	0	0	0	0	0	0	0	0	0	0	0	0	0	0	0
30/10/2020	F	43	0	0	0	0	0	0	0	0	0	0	0	0	0	0	0
10/12/2020	F	43	0	0	0	0	0	0	0	0	0	0	1	0	1	0	0
25/01/2021	F	43	0	0	0	0	0	0	0	0	0	0	0	0	0	0	1
26/01/2021	F	43	0	0	0	0	0	0	0	0	0	0	0	0	0	0	1

19/07/2021	M	43	0	0	0	0	0	0	0	0	0	0	0	0	0	0	1
26/07/2021	F	43	0	0	0	0	0	0	0	0	0	0	0	0	1	0	0
28/10/2021	F	43	0	0	0	0	0	0	0	0	0	0	0	0	0	0	0
01.09.2021	F	43	0	0	0	0	0	0	0	0	0	0	0	0	0	1	0
03.02.2021	F	43	0	0	0	0	0	0	0	0	0	0	1	0	0	0	0
10.11.2021	F	43	0	0	0	0	0	0	0	0	0	0	0	0	0	1	0
21.05.2021	F	43	0	0	0	0	0	0	0	0	0	0	0	0	0	1	0
26.05.2021	F	43	0	0	0	0	0	0	0	0	0	0	0	0	0	1	0
21/01/2022	M	43	0	0	0	0	0	0	0	0	0	0	0	0	0	0	0
20/04/2022	M	43	0	0	0	0	0	0	0	0	0	0	0	0	0	0	0
24/02/2023	F	43	0	0	0	0	0	0	0	0	0	0	1	0	1	0	1
07/06/2023	F	43	0	0	0	0	0	0	0	0	0	0	0	0	0	0	1
13/10/2023	M	43	0	0	0	0	0	0	0	0	0	0	1	0	0	0	0
24/10/2023	F	43	0	0	0	0	0	0	0	0	0	0	0	0	1	0	0
01/04/2024	F	43	0	0	0	0	0	0	0	0	0	0	0	0	0	0	0
21/05/2024	F	43	0	0	0	0	0	0	0	0	0	0	0	0	1	0	0
09/07/2024	F	43	0	0	0	0	0	0	0	0	0	0	0	0	0	0	1
18/09/2024	F	43	0	0	0	0	0	0	0	0	0	0	0	0	0	0	1
08/10/2024	F	43	0	0	0	0	0	0	0	0	0	0	0	0	0	0	0
30/10/2024	M	43	0	0	0	0	0	0	0	0	0	0	0	0	1	0	0
03/12/2024	F	43	0	0	0	0	0	0	0	0	0	0	0	0	1	1	0
17/09/2020	M	43	0	0	0	0	0	0	0	0	0	1	0	0	0	0	0
04.06.2021	F	43	0	0	0	0	0	0	0	0	1	0	0	0	0	0	0
01/02/2022	M	43	0	0	0	0	0	0	0	0	1	0	0	0	0	0	1
08/05/2023	F	43	0	0	0	0	0	0	0	0	0	1	0	0	0	0	1
26/05/2023	F	43	0	0	0	0	0	0	0	0	1	0	0	0	0	0	1
07/05/2024	F	43	0	0	0	0	0	0	0	0	1	0	0	0	0	0	0
23/01/2020	M	43	0	0	0	0	1	0	0	0	0	0	1	0	0	0	0
01/02/2021	M	43	0	0	0	0	1	0	0	0	0	0	0	0	0	0	1

24/05/2021	F	43	0	0	0	0	1	0	0	0	0	0	1	0	0	0	0
15/07/2021	F	43	0	0	0	0	1	0	0	0	0	0	1	0	0	0	1
16/09/2021	F	43	0	0	0	0	0	1	0	0	0	0	1	1	1	0	0
17.03.2021	M	43	1	0	0	0	0	0	0	0	0	0	1	1	0	0	0
01/07/2020	M	43	0	0	1	0	0	0	0	0	0	0	1	1	0	0	1
17/07/2020	M	43	0	0	1	0	0	0	0	0	0	0	1	1	0	0	0
12/08/2020	F	43	0	0	1	0	0	0	0	0	0	0	1	0	0	0	1
18/07/2022	M	43	0	0	1	0	0	0	0	0	0	0	1	1	1	0	0
12/10/2022	M	43	0	0	1	0	0	0	0	0	0	0	1	1	0	0	0
08/02/2023	F	43	0	0	1	0	0	0	0	0	0	0	1	0	0	0	0
20/03/2023	F	43	0	0	1	0	0	0	0	0	0	0	1	1	1	0	0
24/08/2023	F	43	0	0	1	0	0	0	0	0	0	0	1	1	1	0	0
07/02/2024	F	43	0	0	1	0	0	0	0	0	0	0	1	1	0	0	1
26/06/2024	F	43	0	0	1	0	0	0	0	0	0	0	1	1	0	0	0
26/10/2022	M	43	0	0	0	0	0	0	1	0	0	0	1	0	0	0	0
15/12/2022	F	43	0	0	0	0	1	0	0	0	0	0	1	0	1	0	0
26.05.2021	M	43	0	1	0	0	0	0	0	0	0	1	1	0	0	0	0
12/08/2020	M	43	0	0	0	1	0	1	0	0	0	0	1	1	0	0	0
18/02/2021	F	43	0	0	0	0	1	0	1	0	0	0	1	0	0	0	0
08/09/2020	M	43	1	0	1	0	0	0	0	0	0	0	1	1	0	0	0
20/06/2023	F	43	0	1	1	0	0	0	0	0	0	0	1	0	1	0	0
26/03/2024	F	43	0	0	1	0	0	1	0	0	0	0	0	0	0	0	0
09/04/2024	F	43	0	1	1	0	0	0	0	0	0	0	1	1	0	0	0
06/06/2024	M	43	0	0	1	0	1	0	0	0	0	0	1	1	0	0	0
05/04/2023	F	43	0	0	0	0	1	1	0	0	0	0	0	0	0	0	1
30/07/2024	F	43	0	1	0	0	0	1	0	0	0	0	1	1	0	0	0
29/07/2020	M	43	0	1	0	0	1	0	1	0	0	0	1	0	0	0	0
19/05/2020	F	43	0	0	1	1	1	0	0	0	0	0	1	0	1	0	0
09/03/2021	M	43	0	1	1	0	1	0	0	0	0	0	1	1	1	0	0

09/02/2022	M	43	0	1	1	0	1	0	0	0	0	0	1	1	0	0	0
19/09/2022	F	43	1	1	1	0	0	0	0	0	0	0	1	1	0	0	0
31/05/2023	M	43	1	1	1	0	0	0	0	0	0	0	1	0	0	0	0
31/08/2023	F	43	0	1	1	0	1	0	0	0	0	0	1	1	0	0	0
14/06/2023	F	43	1	0	0	0	1	0	1	0	0	0	1	0	1	0	0
04/10/2021	M	43	1	1	1	1	0	0	0	0	0	0	1	1	0	0	1
08/06/2022	F	43	0	1	1	0	1	1	0	0	0	0	0	0	0	0	1
16/01/2020	F	44	0	0	0	0	0	0	0	0	0	0	0	0	1	0	0
12/02/2020	F	44	0	0	0	0	0	0	0	0	0	0	0	0	0	0	1
12/02/2020	M	44	0	0	0	0	0	0	0	0	0	0	1	0	0	0	1
19/06/2020	M	44	0	0	0	0	0	0	0	0	0	0	0	0	0	0	0
01/07/2020	M	44	0	0	0	0	0	0	0	0	0	0	1	0	0	0	0
10/07/2020	F	44	0	0	0	0	0	0	0	0	0	0	0	0	0	0	1
14/01/2021	F	44	0	0	0	0	0	0	0	0	0	0	0	0	1	1	0
09/03/2021	F	44	0	0	0	0	0	0	0	0	0	0	0	0	0	0	0
21/09/2021	F	44	0	0	0	0	0	0	0	0	0	0	1	1	1	0	0
03.02.2021	F	44	0	0	0	0	0	0	0	0	0	0	0	0	1	0	1
07.04.2021	F	44	0	0	0	0	0	0	0	0	0	0	0	1	0	1	0
07/01/2022	F	44	0	0	0	0	0	0	0	0	0	0	0	0	0	0	1
12/01/2022	F	44	0	0	0	0	0	0	0	0	0	0	0	0	0	0	0
14/01/2022	M	44	0	0	0	0	0	0	0	0	0	0	0	0	0	0	1
18/01/2022	F	44	0	0	0	0	0	0	0	0	0	0	1	0	1	0	0
21/01/2022	F	44	0	0	0	0	0	0	0	0	0	0	0	0	0	0	1
16/02/2022	F	44	0	0	0	0	0	0	0	0	0	0	0	0	1	0	1
01/03/2022	F	44	0	0	0	0	0	0	0	0	0	0	1	0	0	0	0
27/04/2022	F	44	0	0	0	0	0	0	0	0	0	0	0	0	0	0	1
20/06/2022	F	44	0	0	0	0	0	0	0	0	0	0	0	0	0	0	1
22/06/2022	M	44	0	0	0	0	0	0	0	0	0	0	1	1	0	0	0
05/07/2022	F	44	0	0	0	0	0	0	0	0	0	0	0	0	0	0	0

26/07/2022	F	44	0	0	0	0	0	0	0	0	0	0	0	0	0	0	1
10/10/2022	F	44	0	0	0	0	0	0	0	0	0	0	0	0	1	0	0
24/10/2022	F	44	0	0	0	0	0	0	0	0	0	0	0	0	1	0	1
17/05/2023	F	44	0	0	0	0	0	0	0	0	0	0	0	0	0	0	1
21/08/2023	M	44	0	0	0	0	0	0	0	0	0	0	0	0	1	0	1
15/11/2023	F	44	0	0	0	0	0	0	0	0	0	0	0	0	0	0	1
17/11/2023	F	44	0	0	0	0	0	0	0	0	0	0	1	0	0	0	0
22/11/2023	F	44	0	0	0	0	0	0	0	0	0	0	0	0	0	0	1
01/03/2024	F	44	0	0	0	0	0	0	0	0	0	0	0	0	0	0	0
07/05/2024	M	44	0	0	0	0	0	0	0	0	0	0	0	0	0	0	1
03/07/2024	M	44	0	0	0	0	0	0	0	0	0	0	0	0	0	0	0
20/08/2024	F	44	0	0	0	0	0	0	0	0	0	0	0	0	0	0	0
07/10/2024	F	44	0	0	0	0	0	0	0	0	0	0	0	0	0	0	0
04/12/2024	M	44	0	0	0	0	0	0	0	0	0	0	1	1	0	0	0
20/12/2024	F	44	0	0	0	0	0	0	0	0	0	0	0	0	0	0	0
22/09/2021	M	44	0	0	0	0	0	0	0	0	0	1	1	0	0	0	0
01/10/2021	F	44	0	0	0	0	0	0	0	0	0	1	1	0	0	0	0
13/10/2021	M	44	0	0	0	0	0	0	0	0	0	1	0	0	0	0	0
02/12/2021	F	44	0	0	0	0	0	0	0	0	1	0	0	0	0	0	0
13.10.2021	F	44	0	0	0	0	0	0	0	0	0	1	0	0	0	0	0
28.04.2021	F	44	0	0	0	0	0	0	0	0	0	1	0	0	0	0	0
13/07/2022	F	44	0	0	0	0	0	0	0	0	1	0	0	0	0	0	0
02/05/2023	M	44	0	0	0	0	0	0	0	0	1	0	0	0	0	0	1
23/01/2024	M	44	0	0	0	0	0	0	0	0	1	0	0	0	0	0	1
01/02/2024	F	44	0	0	0	0	0	0	0	0	1	0	0	0	0	0	0
07/07/2020	F	44	0	0	1	0	0	0	0	0	0	0	1	1	1	0	0
21/01/2021	F	44	0	0	1	0	0	0	0	0	0	0	1	1	1	0	0
28/08/2022	M	44	0	0	1	0	0	0	0	0	0	0	1	0	0	0	0
05/09/2022	M	44	0	0	1	0	0	0	0	0	0	0	1	1	1	0	0

06/09/2022	M	44	0	0	1	0	0	0	0	0	0	0	1	1	0	0	0
24/10/2022	F	44	0	0	1	0	0	0	0	0	0	0	1	1	1	0	0
05/09/2024	F	44	0	0	1	0	0	0	0	0	0	0	1	1	1	0	0
26/08/2024	M	44	0	0	0	0	1	0	0	0	0	0	1	1	0	0	0
24.02.2021	F	44	0	1	0	0	0	1	0	0	0	0	1	1	0	0	0
11/03/2020	M	44	0	0	1	1	0	0	0	0	0	0	1	0	1	0	0
13/08/2020	F	44	1	0	1	0	0	0	0	0	0	0	1	1	0	0	1
01/08/2023	M	44	0	0	1	1	0	0	0	0	0	0	1	1	0	0	0
09/10/2024	M	44	0	0	1	0	1	0	0	0	0	0	1	0	0	0	0
15/04/2022	M	44	1	1	0	0	0	0	0	0	0	0	1	1	1	0	0
28/08/2023	F	44	0	0	0	0	1	0	1	0	0	0	1	1	1	0	0
29/06/2020	M	44	1	1	0	0	1	0	0	0	0	0	1	1	0	0	0
13/04/2021	M	44	1	1	1	0	0	0	0	0	0	0	1	1	0	0	0
14/06/2021	M	44	0	1	1	0	1	0	0	0	0	0	1	1	0	0	0
23/07/2024	F	44	0	1	1	0	1	0	0	0	0	0	1	0	0	0	1
14/01/2020	M	44	1	1	1	1	0	0	0	0	0	0	1	1	0	0	0
06/06/2023	M	44	0	1	1	0	1	1	0	0	0	0	1	0	1	0	0
28/09/2022	M	44	1	1	1	0	1	0	1	0	0	0	1	1	1	1	0
29/10/2020	M	44	1	1	1	1	1	1	1	0	0	0	1	1	1	0	0
27/02/2020	F	45	0	0	0	0	0	0	0	0	0	0	1	0	1	0	0
03/03/2020	M	45	0	0	0	0	0	0	0	0	0	0	0	0	1	0	0
04/03/2020	F	45	0	0	0	0	0	0	0	0	0	0	0	0	0	0	1
09/07/2020	F	45	0	0	0	0	0	0	0	0	0	0	0	0	1	0	0
22/07/2020	F	45	0	0	0	0	0	0	0	0	0	0	1	0	0	0	0
04/08/2020	F	45	0	0	0	0	0	0	0	0	0	0	1	0	0	0	0
15/09/2020	F	45	0	0	0	0	0	0	0	0	0	0	1	1	0	0	0
17/09/2020	F	45	0	0	0	0	0	0	0	0	0	0	1	0	1	0	0
02/11/2020	M	45	0	0	0	0	0	0	0	0	0	0	1	0	1	0	0
03/12/2020	F	45	0	0	0	0	0	0	0	0	0	0	0	0	0	0	1

20/09/2021	M	45	0	0	0	0	0	0	0	0	0	0	1	1	0	0	0
20/09/2021	M	45	0	0	0	0	0	0	0	0	0	0	1	1	0	0	0
07/10/2021	F	45	0	0	0	0	0	0	0	0	0	0	0	0	0	0	0
16/11/2021	F	45	0	0	0	0	0	0	0	0	0	0	0	0	0	0	0
03.02.2021	M	45	0	0	0	0	0	0	0	0	0	0	0	0	0	0	1
03.12.2021	F	45	0	0	0	0	0	0	0	0	0	0	1	0	0	0	0
08/02/2022	M	45	0	0	0	0	0	0	0	0	0	0	0	0	0	0	1
06/06/2022	M	45	0	0	0	0	0	0	0	0	0	0	0	0	0	0	1
25/08/2022	M	45	0	0	0	0	0	0	0	0	0	0	1	0	0	0	0
12/01/2023	M	45	0	0	0	0	0	0	0	0	0	0	0	0	0	0	1
12/04/2023	M	45	0	0	0	0	0	0	0	0	0	0	0	0	0	0	1
18/05/2023	F	45	0	0	0	0	0	0	0	0	0	0	0	0	1	0	0
24/07/2023	F	45	0	0	0	0	0	0	0	0	0	0	0	0	0	0	1
29/09/2023	F	45	0	0	0	0	0	0	0	0	0	0	1	0	0	0	0
06/10/2023	F	45	0	0	0	0	0	0	0	0	0	0	1	0	1	0	0
08/11/2023	F	45	0	0	0	0	0	0	0	0	0	0	0	0	0	0	1
10/04/2024	F	45	0	0	0	0	0	0	0	0	0	0	0	0	0	0	1
20/06/2024	F	45	0	0	0	0	0	0	0	0	0	0	0	0	0	0	0
23/07/2024	F	45	0	0	0	0	0	0	0	0	0	0	0	0	1	0	0
30/08/2024	M	45	0	0	0	0	0	0	0	0	0	0	0	0	0	0	0
28/10/2024	F	45	0	0	0	0	0	0	0	0	0	0	0	0	0	0	1
22/06/2021	M	45	0	0	0	0	0	0	0	1	0	0	0	0	0	0	1
15/07/2021	F	45	0	0	0	0	0	0	0	1	0	0	0	0	0	0	1
25/10/2021	F	45	0	0	0	0	0	0	0	0	1	0	0	0	0	0	1
04.06.2021	F	45	0	0	0	0	0	0	0	0	0	1	1	0	0	0	0
07.05.2021	F	45	0	0	0	0	0	0	0	0	0	1	0	1	0	0	0
14.05.2021	M	45	0	0	0	0	0	0	0	0	0	1	0	0	0	0	0
17.12.2021	F	45	0	0	0	0	0	0	0	0	0	1	0	1	0	0	0
10/06/2022	M	45	0	0	0	0	0	0	0	1	0	0	1	0	0	0	0

20/01/2020	F	45	0	0	0	1	0	0	0	0	0	0	1	0	0	0	0
12/02/2020	M	45	0	0	1	0	0	0	0	0	0	0	1	1	1	0	0
27/02/2020	M	45	0	0	1	0	0	0	0	0	0	0	1	0	0	0	0
22/02/2023	M	45	0	0	1	0	0	0	0	0	0	0	1	0	0	0	0
07/06/2023	M	45	0	0	1	0	0	0	0	0	0	0	1	0	1	0	0
08/08/2023	F	45	0	0	1	0	0	0	0	0	0	0	1	1	0	0	0
24/08/2023	M	45	0	0	1	0	0	0	0	0	0	0	1	1	0	0	0
04/10/2023	M	45	0	0	1	0	0	0	0	0	0	0	1	0	1	0	0
06/03/2024	M	45	0	0	1	0	0	0	0	0	0	0	1	1	1	0	0
11/10/2024	M	45	0	0	1	0	0	0	0	0	0	0	1	1	0	0	0
28/04/2022	M	45	1	0	0	0	0	0	0	0	0	0	1	1	1	0	0
27/09/2022	M	45	1	0	0	0	0	0	0	0	0	0	1	1	0	0	0
08/03/2023	F	45	0	0	0	0	1	0	0	0	0	0	1	0	1	0	0
03.12.2021	F	45	0	0	0	0	1	0	0	0	0	1	0	0	0	0	0
29/09/2021	F	45	0	0	1	0	0	0	0	0	0	1	1	0	0	0	0
02/08/2023	M	45	0	0	1	0	0	0	0	1	0	1	1	1	0	0	0
31/08/2021	F	45	0	0	0	0	1	0	1	0	0	0	1	0	1	0	0
25/09/2020	M	45	0	0	1	0	1	0	0	0	0	0	1	0	1	1	0
11/11/2020	F	45	0	0	1	0	1	0	0	0	0	0	1	1	1	0	0
09/09/2022	M	45	0	0	1	0	0	1	0	0	0	0	1	1	0	0	0
15/05/2023	M	45	0	1	1	0	0	0	0	0	0	0	1	0	0	0	0
03/04/2023	M	45	0	0	0	0	1	0	1	0	0	0	1	1	0	0	0
22/01/2024	F	45	0	1	0	0	1	0	0	0	0	0	1	1	0	0	0
09/03/2020	M	45	0	1	1	0	1	0	0	0	0	0	1	0	1	0	0
06/10/2023	F	45	0	0	1	0	1	0	1	0	0	0	1	0	1	1	0
26/01/2024	M	45	0	1	1	0	0	1	0	0	0	0	1	1	0	0	0
22/11/2021	F	45	0	0	1	1	1	0	0	1	0	0	1	1	1	0	0
03.09.2021	M	45	1	1	1	0	0	0	0	0	0	1	1	0	0	0	0
18/06/2020	F	45	1	1	1	0	0	1	0	0	0	0	1	1	1	0	0

05/02/2020	F	46	0	0	0	0	0	0	0	0	0	0	1	0	1	0	0
26/02/2020	M	46	0	0	0	0	0	0	0	0	0	0	1	0	0	0	0
10/07/2020	F	46	0	0	0	0	0	0	0	0	0	0	0	0	0	0	1
31/07/2020	F	46	0	0	0	0	0	0	0	0	0	0	0	1	0	0	0
02/10/2020	M	46	0	0	0	0	0	0	0	0	0	0	0	0	0	0	1
16/12/2020	M	46	0	0	0	0	0	0	0	0	0	0	0	0	0	0	0
22/03/2021	F	46	0	0	0	0	0	0	0	0	0	0	0	0	0	0	0
27/09/2021	M	46	0	0	0	0	0	0	0	0	0	0	1	1	0	0	0
04/11/2021	F	46	0	0	0	0	0	0	0	0	0	0	1	0	0	0	0
10.09.2021	M	46	0	0	0	0	0	0	0	0	0	0	0	0	0	1	0
26.03.2021	F	46	0	0	0	0	0	0	0	0	0	0	0	0	0	1	1
27.10.2021	M	46	0	0	0	0	0	0	0	0	0	0	0	0	0	1	0
26/01/2022	M	46	0	0	0	0	0	0	0	0	0	0	0	0	0	0	0
07/02/2022	F	46	0	0	0	0	0	0	0	0	0	0	1	0	1	0	0
07/03/2022	F	46	0	0	0	0	0	0	0	0	0	0	0	0	0	0	0
08/03/2022	M	46	0	0	0	0	0	0	0	0	0	0	0	0	0	0	1
26/04/2022	F	46	0	0	0	0	0	0	0	0	0	0	0	0	0	0	1
08/11/2022	M	46	0	0	0	0	0	0	0	0	0	0	0	0	1	0	0
08/11/2022	M	46	0	0	0	0	0	0	0	0	0	0	0	0	1	0	0
22/11/2022	F	46	0	0	0	0	0	0	0	0	0	0	0	0	0	0	1
16/03/2023	F	46	0	0	0	0	0	0	0	0	0	0	0	0	1	0	0
10/04/2023	M	46	0	0	0	0	0	0	0	0	0	0	0	0	0	0	0
13/06/2023	F	46	0	0	0	0	0	0	0	0	0	0	0	0	0	0	1
24/11/2023	F	46	0	0	0	0	0	0	0	0	0	0	0	0	0	0	1
01/03/2024	F	46	0	0	0	0	0	0	0	0	0	0	0	0	0	0	1
07/05/2024	M	46	0	0	0	0	0	0	0	0	0	0	0	0	0	0	0
14/05/2024	F	46	0	0	0	0	0	0	0	0	0	0	0	0	0	0	1
20/06/2024	F	46	0	0	0	0	0	0	0	0	0	0	0	0	0	0	0
01/07/2024	F	46	0	0	0	0	0	0	0	0	0	0	1	0	0	0	0

30/07/2024	F	46	0	0	0	0	0	0	0	0	0	0	0	0	0	0	0
20/08/2024	F	46	0	0	0	0	0	0	0	0	0	0	1	0	0	0	0
03/09/2024	M	46	0	0	0	0	0	0	0	0	0	0	0	0	0	0	1
06/11/2024	M	46	0	0	0	0	0	0	0	0	0	0	1	0	0	0	0
05.11.2021	F	46	0	0	0	0	0	0	0	0	0	1	0	0	0	0	0
17.11.2021	F	46	0	0	0	0	0	0	0	0	0	1	0	1	0	0	0
27/09/2022	M	46	0	0	0	0	0	0	0	0	0	1	0	0	0	0	0
16/02/2023	F	46	0	0	0	0	0	0	0	0	1	0	1	0	1	0	0
03/11/2023	F	46	0	0	0	0	0	0	0	0	0	1	0	0	0	0	0
01/10/2024	F	46	0	0	0	0	0	0	0	0	1	0	0	0	0	0	1
23/11/2020	F	46	0	0	0	0	1	0	0	0	0	0	1	1	1	0	0
07/02/2020	F	46	0	0	1	0	0	0	0	0	0	0	1	0	0	0	1
17/12/2020	F	46	0	0	1	0	0	0	0	0	0	0	1	1	0	0	0
10/05/2022	F	46	0	0	1	0	0	0	0	0	0	0	1	0	1	0	1
02/08/2022	M	46	0	0	1	0	0	0	0	0	0	0	1	1	1	0	0
05/09/2022	F	46	0	0	1	0	0	0	0	0	0	0	1	1	0	0	0
18/04/2023	F	46	0	0	1	0	0	0	0	0	0	0	1	0	0	0	0
30/08/2023	M	46	0	0	1	0	0	0	0	0	0	0	1	0	1	1	0
19/09/2023	F	46	0	0	1	0	0	0	0	0	0	0	1	1	1	0	0
09/05/2024	M	46	0	0	1	0	0	0	0	0	0	0	1	1	1	0	0
31/07/2024	F	46	0	0	1	0	0	0	0	0	0	0	1	1	0	0	0
09/10/2024	F	46	0	0	1	0	0	0	0	0	0	0	1	1	0	0	0
06/11/2024	M	46	0	0	1	0	0	0	0	0	0	0	1	1	0	0	0
14/11/2024	M	46	0	0	1	0	0	0	0	0	0	0	1	0	1	0	0
13/12/2024	F	46	0	0	1	0	0	0	0	0	0	0	1	1	0	0	0
11/04/2023	F	46	0	0	0	0	1	0	0	0	0	0	1	0	1	0	0
28/04/2023	F	46	0	0	0	0	1	0	0	0	0	0	1	0	0	0	0
17/05/2023	M	46	1	0	0	0	0	0	0	0	0	0	1	0	0	0	0
06/10/2023	M	46	0	0	0	0	1	0	0	0	0	0	1	0	0	0	0

19/04/2024	F	46	0	1	0	0	0	0	0	0	0	0	1	0	0	0	0
25.08.2021	F	46	0	0	1	0	0	0	0	0	0	1	1	1	0	0	0
16/11/2022	F	46	0	1	1	0	0	0	0	0	0	0	0	0	0	0	0
05/04/2023	M	46	0	0	1	0	1	0	0	0	0	0	1	0	1	0	0
06/03/2024	M	46	0	0	1	0	0	1	0	0	0	0	1	0	1	0	0
25/08/2022	M	46	0	1	0	0	0	0	1	0	0	0	1	0	1	0	0
05/03/2024	F	46	0	0	0	0	1	1	0	0	0	0	1	0	0	0	0
21.12.2021	F	46	0	0	0	0	1	1	0	0	0	1	0	1	0	0	0
14/10/2020	F	46	0	1	1	0	0	1	0	0	0	0	1	1	0	0	0
11/01/2023	M	46	0	1	1	0	1	0	0	0	0	0	1	0	0	0	0
04/12/2023	F	46	0	1	1	0	1	0	0	0	0	0	0	0	0	0	1
22/05/2024	M	46	1	1	0	0	0	0	1	1	0	0	1	1	1	0	0
14/12/2021	M	46	1	1	0	0	1	1	0	0	0	0	0	0	1	0	0
13/01/2020	M	47	0	0	0	0	0	0	0	0	0	0	0	0	0	0	0
09/10/2020	F	47	0	0	0	0	0	0	0	0	0	0	0	0	0	0	1
18/05/2021	F	47	0	0	0	0	0	0	0	0	0	0	1	0	0	0	0
21/10/2021	M	47	0	0	0	0	0	0	0	0	0	0	0	0	0	0	1
23/11/2021	F	47	0	0	0	0	0	0	0	0	0	0	1	1	0	0	1
10.09.2021	F	47	0	0	0	0	0	0	0	0	0	0	0	0	0	1	0
18/03/2022	F	47	0	0	0	0	0	0	0	0	0	0	0	0	0	0	0
13/05/2022	F	47	0	0	0	0	0	0	0	0	0	0	1	0	0	0	0
21/06/2022	F	47	0	0	0	0	0	0	0	0	0	0	1	0	0	0	0
06/09/2022	F	47	0	0	0	0	0	0	0	0	0	0	0	0	0	0	1
07/09/2022	F	47	0	0	0	0	0	0	0	0	0	0	0	0	0	0	0
08/09/2022	M	47	0	0	0	0	0	0	0	0	0	0	0	0	1	0	0
24/10/2022	F	47	0	0	0	0	0	0	0	0	0	0	0	0	0	0	0
21/03/2023	M	47	0	0	0	0	0	0	0	0	0	0	1	0	0	0	1
28/04/2023	F	47	0	0	0	0	0	0	0	0	0	0	1	0	0	0	0
30/05/2023	F	47	0	0	0	0	0	0	0	0	0	0	0	0	0	0	1

03/10/2023	M	47	0	0	0	0	0	0	0	0	0	0	0	0	1	0	0
27/11/2023	M	47	0	0	0	0	0	0	0	0	0	0	0	0	0	0	1
20/02/2024	F	47	0	0	0	0	0	0	0	0	0	0	1	1	0	0	0
11/03/2024	F	47	0	0	0	0	0	0	0	0	0	0	0	0	0	0	0
14/03/2024	F	47	0	0	0	0	0	0	0	0	0	0	1	0	1	0	0
26/03/2024	M	47	0	0	0	0	0	0	0	0	0	0	0	0	0	0	0
29/03/2024	F	47	0	0	0	0	0	0	0	0	0	0	0	0	0	0	1
12/04/2024	F	47	0	0	0	0	0	0	0	0	0	0	0	0	0	0	0
24/04/2024	F	47	0	0	0	0	0	0	0	0	0	0	0	0	0	0	0
06/06/2024	M	47	0	0	0	0	0	0	0	0	0	0	1	0	0	0	1
04/07/2024	F	47	0	0	0	0	0	0	0	0	0	0	1	0	1	0	0
19/07/2024	M	47	0	0	0	0	0	0	0	0	0	0	0	0	0	0	1
30/07/2024	F	47	0	0	0	0	0	0	0	0	0	0	0	0	0	0	1
21/08/2024	M	47	0	0	0	0	0	0	0	0	0	0	0	0	0	0	1
29/11/2024	F	47	0	0	0	0	0	0	0	0	0	0	0	0	0	0	1
24/09/2020	M	47	0	0	0	0	0	0	0	0	0	1	0	0	0	0	0
08/10/2021	F	47	0	0	0	0	0	0	0	0	0	1	1	0	0	1	0
28/11/2023	F	47	0	0	0	0	0	0	0	1	0	0	0	0	0	0	1
02/07/2024	F	47	0	0	0	0	0	0	0	0	1	0	0	0	0	0	0
03/07/2024	M	47	0	0	0	0	0	0	0	0	1	1	0	0	0	0	0
22/07/2020	F	47	0	1	0	0	0	0	0	0	0	0	1	1	0	0	0
15/02/2023	M	47	0	0	1	0	0	0	0	0	0	0	1	0	1	0	0
24/08/2023	M	47	0	0	1	0	0	0	0	0	0	0	1	1	1	0	0
04/09/2024	M	47	0	0	1	0	0	0	0	0	0	0	1	1	0	0	0
21/11/2023	F	47	1	0	0	0	0	0	0	0	0	0	1	1	1	0	0
29/10/2024	F	47	0	0	0	0	1	0	0	0	0	0	1	1	1	0	0
15/09/2021	F	47	0	0	1	0	0	0	0	0	0	1	1	0	0	0	0
01/03/2023	M	47	0	0	1	0	0	0	0	0	0	1	1	0	1	0	0
26/10/2021	F	47	0	1	0	0	0	0	1	0	0	0	1	0	0	0	0

22/07/2020	M	47	0	0	1	1	0	0	0	0	0	0	1	1	0	0	1
10/06/2021	M	47	0	1	1	0	0	0	0	0	0	0	1	0	0	0	0
07/01/2022	F	47	0	0	1	0	0	1	0	0	0	0	1	0	1	0	0
16/05/2022	F	47	0	0	1	0	0	1	0	0	0	0	1	1	1	0	0
20/05/2022	M	47	0	1	1	0	0	0	0	0	0	0	1	1	0	0	0
02/09/2024	F	47	1	0	1	0	0	0	0	0	0	0	1	1	0	0	0
26/11/2024	F	47	0	1	1	0	0	0	0	0	0	0	1	1	0	0	1
17/03/2023	F	47	1	1	0	0	0	0	0	0	0	0	1	0	0	0	0
30/04/2024	F	47	1	0	0	0	1	0	0	0	0	0	1	1	0	0	0
03.09.2021	M	47	0	0	1	1	0	0	0	0	0	1	1	0	0	0	0
12.11.2021	F	47	0	0	1	0	0	1	0	0	0	1	0	1	0	0	0
10.02.2021	F	47	1	1	0	0	1	0	0	0	0	0	1	1	0	0	0
25/07/2023	F	47	0	1	1	1	0	0	0	0	0	0	1	0	0	0	0
08/05/2024	F	47	0	0	1	0	0	1	1	0	0	0	1	1	1	0	1
10/02/2022	F	47	0	1	0	0	1	1	0	0	0	0	0	0	1	0	1
23/03/2020	M	47	1	1	1	1	0	0	0	0	0	0	1	0	1	0	1
11/01/2022	M	47	1	1	1	1	0	0	0	0	0	0	1	1	0	0	0
14/09/2020	F	48	0	0	0	0	0	0	0	0	0	0	1	0	1	0	0
21/09/2020	M	48	0	0	0	0	0	0	0	0	0	0	1	1	0	0	0
09/10/2020	M	48	0	0	0	0	0	0	0	0	0	0	0	0	0	0	0
12/10/2020	F	48	0	0	0	0	0	0	0	0	0	0	0	0	1	0	0
04/12/2020	F	48	0	0	0	0	0	0	0	0	0	0	1	0	1	0	0
29/04/2021	F	48	0	0	0	0	0	0	0	0	0	0	0	0	0	0	1
07/10/2021	M	48	0	0	0	0	0	0	0	0	0	0	0	0	0	0	0
20/12/2021	F	48	0	0	0	0	0	0	0	0	0	0	1	0	0	0	0
03/11/2023	F	48	0	0	0	0	0	0	0	0	0	0	0	0	0	0	1
21.05.2021	M	48	0	0	0	0	0	0	0	0	0	0	0	0	0	1	0
06/01/2022	F	48	0	0	0	0	0	0	0	0	0	0	0	0	1	0	0
14/01/2022	M	48	0	0	0	0	0	0	0	0	0	0	0	0	0	0	1

28/04/2022	F	48	0	0	0	0	0	0	0	0	0	0	0	0	1	0	0
14/10/2022	F	48	0	0	0	0	0	0	0	0	0	0	0	0	0	0	0
19/10/2022	F	48	0	0	0	0	0	0	0	0	0	0	0	0	0	0	0
14/06/2023	F	48	0	0	0	0	0	0	0	0	0	0	0	0	0	0	1
09/08/2023	F	48	0	0	0	0	0	0	0	0	0	0	1	0	0	0	0
20/09/2023	F	48	0	0	0	0	0	0	0	0	0	0	0	0	0	0	1
20/03/2024	F	48	0	0	0	0	0	0	0	0	0	0	0	0	0	0	1
10/07/2024	F	48	0	0	0	0	0	0	0	0	0	0	0	0	0	0	1
10/07/2024	M	48	0	0	0	0	0	0	0	0	0	0	0	0	0	0	0
11/09/2024	F	48	0	0	0	0	0	0	0	0	0	0	0	0	0	0	1
03/09/2021	M	48	0	0	0	0	0	0	0	0	0	1	0	0	0	0	0
07/12/2022	M	48	0	0	0	0	0	0	0	0	0	1	0	0	0	0	1
11/03/2020	F	48	0	1	0	0	0	0	0	0	0	0	1	0	1	0	0
09/03/2020	F	48	0	0	1	0	0	0	0	0	0	0	0	1	1	0	0
02/10/2020	F	48	0	0	1	0	0	0	0	0	0	0	1	1	1	0	0
07/06/2021	F	48	0	0	1	0	0	0	0	0	0	0	1	1	0	0	0
14/03/2022	F	48	0	0	1	0	0	0	0	0	0	0	1	0	0	0	0
16/12/2022	F	48	0	0	1	0	0	0	0	0	0	0	1	0	0	0	0
21/06/2023	F	48	0	0	1	0	0	0	0	0	0	0	1	0	0	0	1
25/09/2023	M	48	0	0	0	0	0	0	1	0	0	0	1	0	1	0	0
01/04/2021	F	48	0	0	0	0	0	0	1	0	0	1	1	1	0	0	0
26/01/2021	M	48	0	0	0	1	0	0	1	0	0	0	1	0	1	0	0
08/10/2020	M	48	1	0	1	0	0	0	0	0	0	0	1	1	1	0	0
30/09/2024	F	48	0	0	1	0	1	0	0	0	0	0	1	1	1	0	0
16/12/2022	M	48	0	0	1	1	0	1	0	0	0	0	1	0	0	0	0
22/02/2023	F	48	1	1	1	0	0	0	0	0	0	0	1	0	1	0	1
16/06/2023	F	48	0	0	1	0	1	0	1	0	0	0	1	0	1	0	0
17/11/2023	M	48	1	1	1	0	0	0	0	0	0	0	1	1	0	0	0
25/10/2022	F	48	1	1	0	0	1	0	0	0	0	0	1	0	0	0	1

17/09/2020	F	48	0	1	1	0	1	0	1	0	0	0	1	1	0	0	0
19/04/2023	F	48	1	1	1	0	1	0	0	0	0	0	1	0	1	0	0
21.05.2021	M	48	1	1	1	0	0	1	0	0	0	1	1	0	0	0	0
12/10/2022	F	48	0	1	1	1	0	1	1	0	0	0	1	1	0	0	0
13/05/2022	F	48	1	1	1	0	1	1	1	0	0	0	1	1	0	0	0
07/02/2020	F	49	0	0	0	0	0	0	0	0	0	0	0	0	0	0	0
02/07/2020	M	49	0	0	0	0	0	0	0	0	0	0	0	0	0	0	0
22/07/2020	F	49	0	0	0	0	0	0	0	0	0	0	1	0	1	0	0
29/09/2020	F	49	0	0	0	0	0	0	0	0	0	0	1	0	1	0	0
09/11/2020	M	49	0	0	0	0	0	0	0	0	0	0	1	0	0	0	0
28/01/2021	M	49	0	0	0	0	0	0	0	0	0	0	0	0	0	0	0
13/09/2021	F	49	0	0	0	0	0	0	0	0	0	0	0	0	0	0	1
24.03.2021	F	49	0	0	0	0	0	0	0	0	0	0	1	0	0	0	0
10/02/2022	F	49	0	0	0	0	0	0	0	0	0	0	0	0	0	0	0
21/04/2022	M	49	0	0	0	0	0	0	0	0	0	0	0	0	0	0	0
21/11/2022	F	49	0	0	0	0	0	0	0	0	0	0	0	0	0	0	1
12/04/2023	F	49	0	0	0	0	0	0	0	0	0	0	1	0	0	0	0
10/08/2023	F	49	0	0	0	0	0	0	0	0	0	0	1	0	0	0	0
02/10/2023	F	49	0	0	0	0	0	0	0	0	0	0	1	0	0	0	0
24/11/2023	F	49	0	0	0	0	0	0	0	0	0	0	0	0	0	0	1
15/03/2024	F	49	0	0	0	0	0	0	0	0	0	0	0	0	0	0	0
20/03/2024	F	49	0	0	0	0	0	0	0	0	0	0	0	0	1	0	0
16/05/2024	F	49	0	0	0	0	0	0	0	0	0	0	0	0	0	0	0
16/05/2024	F	49	0	0	0	0	0	0	0	0	0	0	1	0	0	0	0
09/07/2024	F	49	0	0	0	0	0	0	0	0	0	0	0	0	0	0	1
31/07/2024	F	49	0	0	0	0	0	0	0	0	0	0	0	0	0	0	1
30/08/2024	F	49	0	0	0	0	0	0	0	0	0	0	1	0	0	0	0
16/10/2024	F	49	0	0	0	0	0	0	0	0	0	0	0	0	0	0	0
25.08.2021	M	49	0	0	0	0	0	0	0	0	0	1	1	0	0	1	0

06/01/2022	F	49	0	0	0	0	0	0	0	0	1	0	0	0	1	0	0
31/05/2023	F	49	0	0	0	0	0	0	0	0	1	0	0	0	0	0	1
31/05/2023	F	49	0	0	0	0	0	0	0	1	0	0	1	0	0	0	0
09/10/2023	M	49	0	0	0	0	0	0	0	0	1	0	0	0	0	0	1
06/06/2024	M	49	0	0	0	0	0	0	0	0	1	0	0	0	0	0	0
27.01.2021	F	49	0	0	0	0	1	0	0	0	0	0	1	1	1	0	0
15/07/2021	M	49	0	0	1	0	0	0	0	0	0	0	1	1	0	0	0
29/08/2023	M	49	0	0	1	0	0	0	0	0	0	0	1	1	1	0	0
04/09/2024	M	49	0	0	1	0	0	0	0	0	0	0	1	1	1	0	0
24/11/2023	F	49	0	0	0	0	1	0	0	0	0	0	0	1	1	0	0
17/09/2020	M	49	0	0	0	0	1	0	0	0	1	0	0	1	0	0	0
27/10/2023	F	49	0	0	1	0	0	0	0	1	0	0	1	0	1	0	1
30/08/2024	F	49	0	0	1	0	0	0	0	0	0	1	1	1	0	0	0
19.05.2021	M	49	0	0	1	0	0	0	0	0	0	1	1	0	0	0	0
19.05.2021	F	49	0	0	1	0	0	0	0	0	0	1	1	0	0	0	0
19/10/2021	M	49	0	1	1	0	0	0	0	0	0	0	1	1	0	0	1
20/05/2022	F	49	0	1	0	0	1	0	0	0	0	0	1	1	0	0	0
19/06/2024	M	49	0	1	1	0	0	0	0	1	0	0	1	1	0	0	1
23/10/2024	F	49	0	1	1	0	0	1	0	0	0	0	1	0	1	0	0
29/10/2020	F	50	0	0	0	0	0	0	0	0	0	0	0	0	1	0	0
03/12/2020	F	50	0	0	0	0	0	0	0	0	0	0	1	0	0	0	0
09/03/2021	M	50	0	0	0	0	0	0	0	0	0	0	1	0	1	0	0
06/05/2021	F	50	0	0	0	0	0	0	0	0	0	0	1	1	0	0	0
05/07/2021	F	50	0	0	0	0	0	0	0	0	0	0	1	0	1	0	0
05/08/2021	F	50	0	0	0	0	0	0	0	0	0	0	1	0	0	0	0
26/10/2021	M	50	0	0	0	0	0	0	0	0	0	0	1	1	1	0	0
25/11/2021	F	50	0	0	0	0	0	0	0	0	0	0	1	1	0	0	0
14/12/2021	M	50	0	0	0	0	0	0	0	0	0	0	0	0	1	0	0
17.03.2021	F	50	0	0	0	0	0	0	0	0	0	0	1	0	0	0	0

20.01.2021	F	50	0	0	0	0	0	0	0	0	0	0	0	0	0	0	1
27/04/2022	M	50	0	0	0	0	0	0	0	0	0	0	0	0	0	0	1
11/05/2022	F	50	0	0	0	0	0	0	0	0	0	0	0	0	0	0	1
10/05/2023	F	50	0	0	0	0	0	0	0	0	0	0	0	0	0	0	1
02/04/2024	F	50	0	0	0	0	0	0	0	0	0	0	0	0	0	0	1
22/04/2024	F	50	0	0	0	0	0	0	0	0	0	0	0	0	1	1	0
26/04/2024	F	50	0	0	0	0	0	0	0	0	0	0	0	0	0	0	1
14/05/2024	F	50	0	0	0	0	0	0	0	0	0	0	0	0	0	0	0
21/05/2024	M	50	0	0	0	0	0	0	0	0	0	0	0	0	0	0	1
21/05/2024	F	50	0	0	0	0	0	0	0	0	0	0	0	0	0	0	0
06/06/2024	F	50	0	0	0	0	0	0	0	0	0	0	0	0	0	0	0
25/06/2024	F	50	0	0	0	0	0	0	0	0	0	0	0	0	0	0	1
15/10/2024	M	50	0	0	0	0	0	0	0	0	0	0	0	0	0	0	0
15/03/2023	F	50	0	0	0	0	0	0	0	1	0	0	0	0	0	0	1
20/04/2023	F	50	0	0	0	0	0	0	0	0	1	0	0	0	0	0	0
19/09/2024	F	50	0	0	0	0	0	0	0	0	1	0	0	0	0	0	0
15/11/2024	F	50	0	0	0	0	0	0	0	0	1	0	0	0	0	0	1
31/05/2023	M	50	0	0	0	0	0	0	0	1	1	0	0	0	0	0	0
08/07/2020	M	50	0	0	0	0	1	0	0	0	0	0	1	1	1	0	0
02/05/2022	M	50	0	0	1	0	0	0	0	0	0	0	0	0	0	0	1
07/09/2023	M	50	0	0	1	0	0	0	0	0	0	0	1	0	1	0	0
13/11/2024	M	50	0	1	0	0	0	0	0	0	0	0	1	0	1	0	0
04/09/2024	M	50	0	0	0	1	1	0	0	0	0	0	1	1	1	0	0
20.10.2021	F	50	0	1	1	0	0	0	0	0	0	1	1	0	0	0	0
21/02/2024	F	50	0	1	1	1	0	0	0	0	0	0	1	1	1	0	0
15/02/2024	M	50	1	0	0	0	1	1	0	0	0	0	1	0	1	0	0
29/08/2023	F	50	1	1	1	0	0	0	0	0	1	0	1	1	0	0	0
13/01/2020	M	51	0	0	0	0	0	0	0	0	0	0	0	0	0	0	1
03/02/2020	F	51	0	0	0	0	0	0	0	0	0	0	1	0	0	0	0

02/03/2020	M	51	0	0	0	0	0	0	0	0	0	0	1	0	0	0	1
11/09/2020	F	51	0	0	0	0	0	0	0	0	0	0	0	0	0	0	0
22/12/2020	M	51	0	0	0	0	0	0	0	0	0	0	0	0	1	0	0
22/03/2021	M	51	0	0	0	0	0	0	0	0	0	0	1	0	1	0	1
20/07/2021	F	51	0	0	0	0	0	0	0	0	0	0	0	0	0	0	0
23/08/2021	F	51	0	0	0	0	0	0	0	0	0	0	0	0	0	0	1
24.02.2021	F	51	0	0	0	0	0	0	0	0	0	0	0	0	1	1	1
27.08.2021	M	51	0	0	0	0	0	0	0	0	0	0	0	0	0	1	0
23/02/2022	F	51	0	0	0	0	0	0	0	0	0	0	0	0	0	0	1
23/02/2022	F	51	0	0	0	0	0	0	0	0	0	0	1	0	0	0	1
28/03/2022	M	51	0	0	0	0	0	0	0	0	0	0	0	0	0	0	0
12/04/2022	F	51	0	0	0	0	0	0	0	0	0	0	0	0	0	0	0
13/05/2022	F	51	0	0	0	0	0	0	0	0	0	0	0	0	0	0	1
18/07/2022	M	51	0	0	0	0	0	0	0	0	0	0	0	0	0	0	0
18/07/2022	M	51	0	0	0	0	0	0	0	0	0	0	0	0	0	0	1
28/07/2022	F	51	0	0	0	0	0	0	0	0	0	0	1	0	0	0	0
02/08/2022	F	51	0	0	0	0	0	0	0	0	0	0	0	0	0	0	0
07/10/2022	F	51	0	0	0	0	0	0	0	0	0	0	0	0	0	0	1
26/10/2022	F	51	0	0	0	0	0	0	0	0	0	0	1	0	1	0	0
19/12/2022	F	51	0	0	0	0	0	0	0	0	0	0	0	0	0	0	0
05/04/2023	F	51	0	0	0	0	0	0	0	0	0	0	1	0	0	0	0
05/04/2023	F	51	0	0	0	0	0	0	0	0	0	0	1	0	0	0	0
26/05/2023	F	51	0	0	0	0	0	0	0	0	0	0	0	0	0	0	1
25/03/2024	F	51	0	0	0	0	0	0	0	0	0	0	0	0	1	0	0
18/09/2024	F	51	0	0	0	0	0	0	0	0	0	0	1	1	1	0	0
04/03/2020	F	51	0	0	0	0	0	0	0	0	1	0	0	0	0	0	0
27.01.2021	F	51	0	0	0	0	0	0	0	0	1	0	0	0	0	0	0
28/06/2022	F	51	0	0	0	0	0	0	0	0	1	0	0	0	0	0	0
25/09/2023	F	51	0	0	0	0	0	0	0	0	1	0	0	0	0	0	0

27.01.2021	M	51	0	0	1	0	0	0	0	0	0	0	1	1	0	0	0
29/09/2023	F	51	1	0	0	0	0	0	0	0	0	0	1	0	0	0	0
15/09/2021	M	51	0	0	1	0	0	0	0	0	0	1	1	0	0	1	1
29/08/2024	M	51	0	1	1	0	0	0	0	0	0	0	1	0	0	0	0
03/02/2020	M	51	1	0	0	1	1	0	0	0	0	0	0	0	0	0	1
07/10/2021	M	51	0	0	1	1	0	1	0	0	0	0	1	1	0	0	0
15/03/2023	F	51	1	0	1	1	0	0	0	0	0	0	1	0	1	0	0
07/12/2021	F	51	1	1	1	0	0	0	0	1	0	0	1	1	0	0	0
07/05/2024	F	51	0	1	1	0	1	1	0	0	0	0	1	1	1	0	0
30/01/2020	F	52	0	0	0	0	0	0	0	0	0	0	0	0	0	0	1
24/02/2020	F	52	0	0	0	0	0	0	0	0	0	0	0	0	0	0	1
25/02/2020	M	52	0	0	0	0	0	0	0	0	0	0	0	0	0	0	1
14/08/2020	F	52	0	0	0	0	0	0	0	0	0	0	0	0	0	0	1
09/10/2020	F	52	0	0	0	0	0	0	0	0	0	0	1	0	0	0	0
28/10/2020	M	52	0	0	0	0	0	0	0	0	0	0	0	0	0	0	0
12/04/2021	F	52	0	0	0	0	0	0	0	0	0	0	0	0	0	0	1
20/07/2021	F	52	0	0	0	0	0	0	0	0	0	0	1	0	0	0	0
25/11/2021	F	52	0	0	0	0	0	0	0	0	0	0	1	0	0	0	0
20.10.2021	F	52	0	0	0	0	0	0	0	0	0	0	0	0	0	1	0
25.08.2021	F	52	0	0	0	0	0	0	0	0	0	0	0	0	0	1	0
07/02/2022	F	52	0	0	0	0	0	0	0	0	0	0	1	0	1	0	0
14/02/2022	F	52	0	0	0	0	0	0	0	0	0	0	0	0	0	0	0
30/03/2022	F	52	0	0	0	0	0	0	0	0	0	0	0	0	0	0	1
14/04/2022	F	52	0	0	0	0	0	0	0	0	0	0	0	0	0	0	0
15/04/2022	F	52	0	0	0	0	0	0	0	0	0	0	1	0	0	0	0
02/05/2022	M	52	0	0	0	0	0	0	0	0	0	0	0	0	0	0	0
20/05/2022	F	52	0	0	0	0	0	0	0	0	0	0	0	0	0	0	1
20/05/2022	F	52	0	0	0	0	0	0	0	0	0	0	0	0	0	0	1
07/06/2022	M	52	0	0	0	0	0	0	0	0	0	0	1	1	0	0	0

06/09/2022	F	52	0	0	0	0	0	0	0	0	0	0	0	0	0	0	0
30/09/2022	F	52	0	0	0	0	0	0	0	0	0	0	0	0	0	0	0
10/10/2022	F	52	0	0	0	0	0	0	0	0	0	0	0	0	0	0	1
09/03/2023	F	52	0	0	0	0	0	0	0	0	0	0	0	0	0	0	1
25/08/2023	F	52	0	0	0	0	0	0	0	0	0	0	1	0	1	0	0
15/12/2023	M	52	0	0	0	0	0	0	0	0	0	0	0	0	0	0	0
17/01/2024	F	52	0	0	0	0	0	0	0	0	0	0	0	0	0	0	1
16/02/2024	F	52	0	0	0	0	0	0	0	0	0	0	1	0	0	0	0
27/06/2024	F	52	0	0	0	0	0	0	0	0	0	0	1	0	1	0	0
06/10/2024	F	52	0	0	0	0	0	0	0	0	0	0	0	0	0	0	1
07/01/2020	F	52	0	0	0	0	0	0	0	0	1	0	0	0	0	0	0
21/02/2020	F	52	0	0	0	0	0	0	0	0	1	0	0	0	0	0	1
19/07/2021	F	52	0	0	0	0	0	0	0	0	1	0	0	0	0	0	1
08/11/2021	F	52	0	0	0	0	0	0	0	0	1	0	1	1	0	0	0
08.09.2021	F	52	0	0	0	0	0	0	0	0	0	1	1	0	0	0	0
07/09/2020	F	52	0	0	0	0	0	1	0	0	0	0	1	1	0	0	0
14/01/2021	F	52	0	1	0	0	0	0	0	0	0	0	0	0	1	0	1
07/02/2020	F	52	0	0	1	0	0	0	0	0	0	0	1	1	1	0	1
19/05/2020	F	52	0	0	1	0	0	0	0	0	0	0	1	0	0	0	0
11/08/2020	F	52	0	0	1	0	0	0	0	0	0	0	1	1	0	0	0
07/01/2021	M	52	0	0	1	0	0	0	0	0	0	0	1	1	1	0	0
24/05/2022	M	52	0	0	1	0	0	0	0	0	0	0	1	1	0	0	0
10/02/2022	F	52	0	1	0	0	0	0	0	0	0	0	1	0	1	0	0
10/04/2023	M	52	0	0	0	0	1	0	0	0	0	0	1	0	0	0	0
09/07/2024	F	52	0	0	0	0	0	0	1	0	0	0	1	0	1	0	0
09/11/2021	F	52	0	0	1	0	1	0	0	0	0	0	1	1	1	0	0
02/02/2023	M	52	0	1	0	0	1	0	0	0	0	0	1	1	0	0	0
12/05/2023	F	52	1	0	0	0	0	0	1	0	0	0	1	0	0	0	0
02/08/2024	M	52	0	0	0	0	1	1	0	0	0	0	1	1	0	0	0

16/11/2023	F	52	1	0	0	0	0	1	0	0	1	0	0	0	0	0	1
17/01/2024	F	52	0	1	1	0	1	0	0	0	0	0	1	1	1	0	0
23/04/2024	F	52	1	1	1	0	0	0	0	0	0	0	1	1	0	0	0
29/08/2024	M	52	0	1	1	0	0	1	0	0	0	0	1	1	0	0	0
23/02/2023	M	52	1	0	0	0	1	0	1	0	1	0	1	0	1	0	0
08/11/2023	F	52	0	0	1	0	1	1	1	0	0	0	1	0	1	0	0
15/06/2020	F	53	0	0	0	0	0	0	0	0	0	0	1	1	0	0	0
08/07/2021	M	53	0	0	0	0	0	0	0	0	0	0	0	0	0	0	0
05.03.2021	M	53	0	0	0	0	0	0	0	0	0	0	0	0	0	1	1
25/02/2022	F	53	0	0	0	0	0	0	0	0	0	0	0	0	0	0	0
13/05/2022	F	53	0	0	0	0	0	0	0	0	0	0	1	0	1	0	0
20/05/2022	F	53	0	0	0	0	0	0	0	0	0	0	1	0	0	0	1
16/06/2022	F	53	0	0	0	0	0	0	0	0	0	0	0	0	1	0	0
19/04/2023	F	53	0	0	0	0	0	0	0	0	0	0	1	0	1	0	1
16/06/2023	F	53	0	0	0	0	0	0	0	0	0	0	0	0	1	0	0
07/08/2023	F	53	0	0	0	0	0	0	0	0	0	0	0	0	0	0	0
06/10/2023	F	53	0	0	0	0	0	0	0	0	0	0	0	0	1	0	0
07/02/2024	F	53	0	0	0	0	0	0	0	0	0	0	0	0	0	0	1
08/02/2024	M	53	0	0	0	0	0	0	0	0	0	0	0	0	0	0	1
28/02/2024	M	53	0	0	0	0	0	0	0	0	0	0	0	0	1	0	0
13/05/2024	M	53	0	0	0	0	0	0	0	0	0	0	0	0	0	0	1
28/05/2024	M	53	0	0	0	0	0	0	0	0	0	0	0	0	0	0	0
19/07/2021	M	53	0	0	0	0	0	0	0	0	0	1	0	0	0	0	0
17.11.2021	M	53	0	0	0	0	0	0	0	0	0	1	0	0	0	0	0
26.02.2021	F	53	0	0	0	0	0	0	0	0	0	1	0	0	0	0	0
12/01/2022	F	53	0	0	0	0	0	0	0	1	0	0	0	0	0	0	0
06/02/2023	M	53	0	0	0	0	0	0	0	0	1	0	0	0	0	0	1
27/03/2023	M	53	0	0	0	0	0	0	0	1	0	0	1	1	0	0	0
15/04/2021	M	53	0	0	0	0	0	0	1	0	0	0	1	0	0	0	0

24.03.2021	F	53	0	0	0	0	1	0	0	0	0	0	1	0	1	0	1
29/03/2021	F	53	0	0	1	0	0	0	0	0	0	0	1	1	0	0	0
23/03/2022	F	53	0	0	1	0	0	0	0	0	0	0	1	0	0	0	0
17/03/2022	F	53	0	0	0	0	1	0	0	0	0	0	1	1	0	0	0
06/10/2023	M	53	0	1	0	0	0	0	0	0	0	0	1	0	1	0	0
10.09.2021	F	53	0	0	1	0	0	0	0	0	0	1	1	0	0	0	0
01/09/2022	M	53	0	1	1	0	0	0	0	0	0	0	1	1	0	0	1
29/09/2022	F	53	0	0	1	0	1	0	0	0	0	0	1	1	1	0	0
21/03/2022	F	53	1	0	0	0	0	0	1	0	0	0	1	0	1	0	0
25/10/2023	M	53	1	1	0	0	0	0	0	0	0	0	1	0	1	0	0
22/09/2021	F	53	0	1	1	0	0	0	0	0	0	1	1	0	0	0	0
09/09/2021	F	53	1	1	1	0	0	0	0	0	0	0	1	1	0	0	0
07/01/2020	M	54	0	0	0	0	0	0	0	0	0	0	0	0	0	0	1
30/01/2020	M	54	0	0	0	0	0	0	0	0	0	0	0	1	0	0	0
22/06/2020	M	54	0	0	0	0	0	0	0	0	0	0	1	0	0	0	0
27/07/2020	F	54	0	0	0	0	0	0	0	0	0	0	1	0	0	0	0
29/07/2020	F	54	0	0	0	0	0	0	0	0	0	0	0	0	0	0	0
05/10/2020	M	54	0	0	0	0	0	0	0	0	0	0	1	0	0	0	0
27/11/2020	F	54	0	0	0	0	0	0	0	0	0	0	1	0	0	0	0
19/01/2021	M	54	0	0	0	0	0	0	0	0	0	0	0	1	1	0	0
01/02/2021	F	54	0	0	0	0	0	0	0	0	0	0	0	0	0	0	1
05/08/2021	F	54	0	0	0	0	0	0	0	0	0	0	1	0	1	0	0
14/12/2021	F	54	0	0	0	0	0	0	0	0	0	0	0	0	0	0	0
17.03.2021	F	54	0	0	0	0	0	0	0	0	0	0	0	0	1	0	1
04/02/2022	F	54	0	0	0	0	0	0	0	0	0	0	0	0	1	0	0
17/02/2022	F	54	0	0	0	0	0	0	0	0	0	0	0	0	0	0	1
18/03/2022	F	54	0	0	0	0	0	0	0	0	0	0	0	0	0	0	1
25/08/2022	F	54	0	0	0	0	0	0	0	0	0	0	0	0	0	0	1
29/09/2022	M	54	0	0	0	0	0	0	0	0	0	0	0	0	1	0	0

17/10/2022	F	54	0	0	0	0	0	0	0	0	0	0	0	0	1	0	0
08/02/2023	F	54	0	0	0	0	0	0	0	0	0	0	0	0	0	0	1
09/02/2023	F	54	0	0	0	0	0	0	0	0	0	0	0	0	0	0	1
22/02/2023	M	54	0	0	0	0	0	0	0	0	0	0	0	0	0	0	1
27/02/2023	F	54	0	0	0	0	0	0	0	0	0	0	0	0	0	0	0
26/05/2023	F	54	0	0	0	0	0	0	0	0	0	0	0	0	0	0	1
17/08/2023	M	54	0	0	0	0	0	0	0	0	0	0	0	0	0	0	1
12/10/2023	M	54	0	0	0	0	0	0	0	0	0	0	1	0	0	0	0
21/02/2024	F	54	0	0	0	0	0	0	0	0	0	0	0	0	0	0	0
20/03/2024	F	54	0	0	0	0	0	0	0	0	0	0	0	0	0	0	1
25/03/2024	F	54	0	0	0	0	0	0	0	0	0	0	1	0	0	0	0
30/04/2024	M	54	0	0	0	0	0	0	0	0	0	0	0	0	0	0	0
28/05/2024	F	54	0	0	0	0	0	0	0	0	0	0	1	0	0	0	0
20/06/2024	F	54	0	0	0	0	0	0	0	0	0	0	0	0	0	0	1
01/07/2024	F	54	0	0	0	0	0	0	0	0	0	0	0	0	0	0	0
02/07/2024	F	54	0	0	0	0	0	0	0	0	0	0	0	0	0	0	0
15/10/2024	F	54	0	0	0	0	0	0	0	0	0	0	0	0	0	0	0
16/10/2024	M	54	0	0	0	0	0	0	0	0	0	0	0	0	0	0	0
30/10/2024	F	54	0	0	0	0	0	0	0	0	0	0	0	0	0	0	1
17/09/2020	M	54	0	0	0	0	0	0	0	0	1	0	0	0	0	0	0
26/07/2021	F	54	0	0	0	0	0	0	0	0	1	0	0	0	0	0	0
18/07/2022	M	54	0	0	0	0	0	0	0	0	0	1	0	0	0	0	1
13/12/2022	F	54	0	0	0	0	0	0	0	0	1	0	0	0	1	0	0
10/05/2023	F	54	0	0	0	0	0	0	0	1	0	0	0	0	0	0	1
12/05/2023	F	54	0	0	0	0	0	0	0	0	1	0	0	0	0	0	1
18/11/2020	F	54	0	0	0	0	1	0	0	0	0	0	1	0	0	0	0
12/04/2023	F	54	0	0	1	0	0	0	0	0	0	0	1	0	1	0	0
03/09/2024	M	54	0	0	1	0	0	0	0	0	0	0	1	1	0	0	1
09/04/2024	M	54	0	0	0	0	1	0	0	0	0	0	1	0	0	0	0

23/10/2024	F	54	0	0	0	0	1	0	0	0	0	0	1	0	0	0	0
08/10/2021	F	54	0	0	0	0	0	0	1	0	0	1	1	1	0	0	1
26.11.2021	M	54	0	0	1	0	0	0	0	0	1	1	0	1	0	0	0
17/02/2022	F	54	1	0	1	0	0	0	0	0	0	0	1	1	0	0	0
24/08/2023	M	54	0	0	1	1	0	0	0	0	0	0	1	1	0	0	0
18/09/2023	M	54	0	0	1	1	1	0	0	0	0	0	1	1	0	0	1
31/08/2020	M	55	0	0	0	0	0	0	0	0	0	0	1	1	0	0	0
15/09/2020	M	55	0	0	0	0	0	0	0	0	0	0	1	1	0	0	0
23/12/2020	F	55	0	0	0	0	0	0	0	0	0	0	0	0	0	0	0
26/10/2021	F	55	0	0	0	0	0	0	0	0	0	0	1	0	0	0	1
02/11/2021	F	55	0	0	0	0	0	0	0	0	0	0	0	0	1	0	0
02/02/2022	M	55	0	0	0	0	0	0	0	0	0	0	0	0	0	0	0
26/07/2022	M	55	0	0	0	0	0	0	0	0	0	0	0	0	0	0	0
25/08/2022	M	55	0	0	0	0	0	0	0	0	0	0	1	0	0	0	1
31/08/2022	F	55	0	0	0	0	0	0	0	0	0	0	0	0	0	0	1
12/09/2022	F	55	0	0	0	0	0	0	0	0	0	0	0	0	0	0	0
01/11/2022	F	55	0	0	0	0	0	0	0	0	0	0	0	0	0	0	0
18/11/2022	F	55	0	0	0	0	0	0	0	0	0	0	0	0	0	0	1
16/01/2023	F	55	0	0	0	0	0	0	0	0	0	0	0	0	1	0	1
07/03/2023	M	55	0	0	0	0	0	0	0	0	0	0	1	0	1	0	0
06/04/2023	F	55	0	0	0	0	0	0	0	0	0	0	0	0	0	0	1
11/04/2023	F	55	0	0	0	0	0	0	0	0	0	0	0	0	0	0	0
30/05/2023	F	55	0	0	0	0	0	0	0	0	0	0	0	0	1	0	0
16/08/2023	M	55	0	0	0	0	0	0	0	0	0	0	0	0	0	0	1
19/09/2023	M	55	0	0	0	0	0	0	0	0	0	0	0	0	0	0	1
23/02/2024	F	55	0	0	0	0	0	0	0	0	0	0	1	0	0	0	0
23/08/2024	M	55	0	0	0	0	0	0	0	0	0	0	1	0	1	0	0
26/09/2024	F	55	0	0	0	0	0	0	0	0	0	0	1	0	1	0	0
16/10/2024	M	55	0	0	0	0	0	0	0	0	0	0	0	0	0	0	1

04/12/2024	M	55	0	0	0	0	0	0	0	0	0	0	0	0	0	0	0
25/10/2021	F	55	0	0	0	0	0	0	0	0	1	0	0	0	0	0	0
21/07/2022	F	55	0	0	0	0	0	0	0	0	1	0	0	0	0	0	0
02/08/2023	F	55	0	0	1	0	0	0	0	0	0	0	1	0	0	0	1
12/07/2021	M	55	0	0	1	0	0	0	0	1	0	0	0	0	0	0	1
18/07/2022	F	55	0	0	0	0	1	0	0	0	0	1	0	0	1	0	1
19/05/2023	F	55	0	0	1	0	0	0	0	1	1	0	1	0	1	0	1
19.11.2021	F	55	0	1	1	0	0	0	0	0	0	1	1	0	0	0	0
18/11/2020	F	55	1	1	1	0	0	0	0	0	0	0	1	1	0	0	0
24/05/2022	M	55	1	1	1	0	1	0	0	0	0	0	1	1	0	1	0
22/03/2023	M	55	1	1	1	1	0	0	0	0	0	0	1	0	0	0	1
09/11/2020	F	56	0	0	0	0	0	0	0	0	0	0	1	0	0	0	1
16/11/2020	F	56	0	0	0	0	0	0	0	0	0	0	0	0	1	0	0
21.12.2021	F	56	0	0	0	0	0	0	0	0	0	0	0	0	0	0	0
11/01/2022	F	56	0	0	0	0	0	0	0	0	0	0	0	1	0	0	0
02/02/2022	M	56	0	0	0	0	0	0	0	0	0	0	0	0	0	0	0
28/03/2022	F	56	0	0	0	0	0	0	0	0	0	0	0	0	0	0	0
28/06/2023	F	56	0	0	0	0	0	0	0	0	0	0	1	0	0	0	0
25/09/2023	F	56	0	0	0	0	0	0	0	0	0	0	0	0	0	0	1
26/01/2024	F	56	0	0	0	0	0	0	0	0	0	0	0	0	0	0	0
14/02/2024	F	56	0	0	0	0	0	0	0	0	0	0	0	0	0	0	0
14/05/2024	F	56	0	0	0	0	0	0	0	0	0	0	0	0	0	0	0
19/09/2024	F	56	0	0	0	0	0	0	0	0	0	0	0	0	0	0	0
29/11/2021	F	56	0	0	0	0	0	0	0	1	1	0	0	0	0	0	0
26/10/2021	F	56	0	0	0	0	1	0	0	0	0	0	1	0	0	0	1
16/07/2024	M	56	0	0	1	0	0	0	0	0	0	1	1	1	0	0	0
18/03/2022	M	56	0	0	1	0	1	0	0	0	0	0	1	0	1	0	0
05/09/2024	M	56	0	1	1	0	0	1	0	0	0	0	1	1	1	1	0
07/02/2024	M	56	1	1	0	0	1	0	1	0	0	0	1	0	0	0	1

31/08/2020	F	57	0	0	0	0	0	0	0	0	0	0	0	0	0	0	0
15/09/2020	M	57	0	0	0	0	0	0	0	0	0	0	1	1	0	0	0
11/02/2021	F	57	0	0	0	0	0	0	0	0	0	0	0	0	0	0	0
12/04/2021	M	57	0	0	0	0	0	0	0	0	0	0	0	0	0	0	0
20/07/2021	M	57	0	0	0	0	0	0	0	0	0	0	0	0	1	0	0
24/08/2021	F	57	0	0	0	0	0	0	0	0	0	0	0	0	0	0	1
25/10/2021	F	57	0	0	0	0	0	0	0	0	0	0	1	0	0	0	0
26/10/2021	F	57	0	0	0	0	0	0	0	0	0	0	0	0	0	0	0
15.09.2021	M	57	0	0	0	0	0	0	0	0	0	0	0	0	0	1	0
27.10.2021	F	57	0	0	0	0	0	0	0	0	0	0	0	0	0	0	0
31.01.2021	F	57	0	0	0	0	0	0	0	0	0	0	0	0	1	0	1
03/03/2023	F	57	0	0	0	0	0	0	0	0	0	0	1	0	0	0	1
06/04/2023	F	57	0	0	0	0	0	0	0	0	0	0	0	0	0	0	0
27/07/2023	F	57	0	0	0	0	0	0	0	0	0	0	1	0	1	0	1
04/09/2023	F	57	0	0	0	0	0	0	0	0	0	0	0	0	1	0	1
07/11/2023	F	57	0	0	0	0	0	0	0	0	0	0	1	0	0	0	0
27/11/2023	F	57	0	0	0	0	0	0	0	0	0	0	0	0	0	0	1
25/06/2024	M	57	0	0	0	0	0	0	0	0	0	0	0	0	0	0	1
03/07/2024	F	57	0	0	0	0	0	0	0	0	0	0	0	0	0	0	0
17/07/2024	M	57	0	0	0	0	0	0	0	0	0	0	1	0	0	0	0
31/07/2024	F	57	0	0	0	0	0	0	0	0	0	0	0	0	1	0	0
23/08/2024	F	57	0	0	0	0	0	0	0	0	0	0	0	0	0	0	0
06/09/2024	F	57	0	0	0	0	0	0	0	0	0	0	0	0	1	0	0
17/12/2024	F	57	0	0	0	0	0	0	0	0	0	0	0	0	1	0	0
08/07/2021	F	57	0	0	0	0	0	0	0	0	1	0	0	0	0	0	1
27/06/2024	F	57	0	0	0	0	0	0	0	0	1	0	0	0	0	0	0
20/01/2020	M	57	0	0	1	0	0	0	1	0	0	0	1	1	0	0	0
07/01/2021	F	57	0	0	1	0	0	1	0	0	0	0	1	1	1	0	0
11/01/2023	M	57	0	1	1	0	0	0	0	0	0	0	1	0	0	0	0

25/10/2022	F	57	0	0	0	0	0	1	1	0	0	0	1	0	1	0	0
14/10/2022	F	57	1	1	1	0	0	0	0	0	0	0	1	0	1	0	0
30/05/2023	F	57	0	0	1	0	1	1	0	0	0	0	1	0	1	0	0
04/12/2024	F	57	1	1	1	0	0	1	1	0	0	0	1	1	1	0	0
15/01/2020	F	58	0	0	0	0	0	0	0	0	0	0	0	0	1	0	0
17/03/2020	F	58	0	0	0	0	0	0	0	0	0	0	0	0	0	0	1
28/10/2020	F	58	0	0	0	0	0	0	0	0	0	0	0	0	0	0	0
03.02.2021	M	58	0	0	0	0	0	0	0	0	0	0	0	0	0	0	0
26.02.2021	M	58	0	0	0	0	0	0	0	0	0	0	0	0	0	1	0
06/01/2022	F	58	0	0	0	0	0	0	0	0	0	0	0	0	0	0	0
07/02/2022	M	58	0	0	0	0	0	0	0	0	0	0	1	0	1	0	0
07/03/2022	F	58	0	0	0	0	0	0	0	0	0	0	1	0	1	0	0
14/12/2022	F	58	0	0	0	0	0	0	0	0	0	0	0	0	0	0	1
14/02/2023	F	58	0	0	0	0	0	0	0	0	0	0	0	0	0	0	0
08/03/2023	F	58	0	0	0	0	0	0	0	0	0	0	0	0	0	0	1
20/09/2023	F	58	0	0	0	0	0	0	0	0	0	0	1	0	0	0	0
23/02/2024	M	58	0	0	0	0	0	0	0	0	0	0	0	0	0	0	0
14/03/2024	M	58	0	0	0	0	0	0	0	0	0	0	0	0	0	0	0
10/04/2024	M	58	0	0	0	0	0	0	0	0	0	0	0	0	0	0	1
21/05/2024	M	58	0	0	0	0	0	0	0	0	0	0	0	0	0	0	0
22/05/2024	F	58	0	0	0	0	0	0	0	0	0	0	0	0	0	0	1
09/02/2022	M	58	0	0	1	0	0	0	0	0	0	0	1	1	0	0	0
24/11/2023	F	58	0	0	1	0	0	0	0	0	0	0	1	1	1	0	0
22/03/2024	F	58	0	0	0	0	0	1	0	0	0	0	1	0	0	0	0
25/09/2023	F	58	0	0	1	0	0	0	0	1	0	0	1	1	0	0	1
15/10/2020	F	58	1	0	1	0	0	0	0	0	0	0	1	1	0	0	0
08/10/2020	F	58	1	1	1	0	0	0	0	0	0	0	1	1	0	0	0
17.02.2021	F	58	0	0	1	1	1	1	0	0	0	0	1	1	1	0	1
02/02/2021	F	59	0	0	0	0	0	0	0	0	0	0	0	0	0	0	1

25/05/2024	F	59	0	0	0	0	0	0	0	0	0	0	0	0	0	0	1
08/03/2022	F	59	0	0	0	0	0	0	0	0	0	0	0	0	0	0	1
22/06/2022	M	59	0	0	0	0	0	0	0	0	0	0	0	0	0	0	0
27/10/2022	M	59	0	0	0	0	0	0	0	0	0	0	0	0	0	0	0
27/10/2022	F	59	0	0	0	0	0	0	0	0	0	0	0	0	0	0	0
28/05/2024	M	59	0	0	0	0	0	0	0	0	0	0	1	0	1	0	0
28/05/2024	F	59	0	0	0	0	0	0	0	0	0	0	1	1	1	0	0
11/11/2024	F	59	0	0	0	0	0	0	0	0	0	0	1	0	0	0	0
31/08/2021	F	59	0	0	0	0	0	0	0	0	1	0	0	0	0	0	0
27.10.2021	F	59	0	0	0	0	0	0	0	0	0	1	0	1	0	0	0
29.01.2021	F	59	0	0	0	0	0	0	0	0	1	0	1	0	0	0	0
10/01/2022	M	59	0	0	0	0	0	0	0	1	0	0	1	0	1	0	1
16/01/2020	M	59	0	1	0	0	0	0	0	0	0	0	1	1	0	0	0
21/11/2022	F	59	0	0	1	0	0	0	0	0	0	0	1	0	0	0	0
08/05/2024	F	59	0	0	1	0	0	0	0	0	0	0	1	0	0	0	0
14/11/2024	M	59	0	0	1	0	0	0	0	0	0	0	1	1	1	0	0
22/11/2022	F	59	0	0	0	0	0	1	0	0	0	0	1	0	0	0	1
01/10/2021	M	59	0	1	0	0	1	0	0	0	0	0	1	0	0	0	0
16/01/2023	M	59	1	0	0	0	0	0	1	0	0	0	1	0	1	0	0
19/02/2020	F	60	0	0	0	0	0	0	0	0	0	0	1	1	0	0	0
12/03/2020	F	60	0	0	0	0	0	0	0	0	0	0	0	0	0	0	0
14/04/2021	F	60	0	0	0	0	0	0	0	0	0	0	0	0	0	0	1
08/02/2024	F	60	0	0	0	0	0	0	0	0	0	0	0	0	0	0	1
01/03/2024	F	60	0	0	0	0	0	0	0	0	0	0	0	0	1	0	0
19/03/2024	M	60	0	0	0	0	0	0	0	0	0	0	0	0	0	0	1
28/10/2024	F	60	0	0	0	0	0	0	0	0	0	0	0	0	0	0	0
13/02/2020	F	60	0	0	0	0	0	0	0	0	1	0	0	0	0	0	0
28/06/2021	F	60	0	0	0	0	0	0	0	0	1	0	1	0	0	0	0
22/05/2024	M	60	0	0	0	0	0	0	0	0	0	1	0	0	0	0	1

18/02/2020	M	60	0	0	1	0	0	0	0	0	0	0	1	0	0	0	0
30/06/2020	M	60	0	0	1	0	0	0	0	0	0	0	1	1	1	0	0
05/09/2022	F	60	0	0	1	0	0	0	1	0	0	0	1	0	1	0	0
21.05.2021	F	60	0	1	1	1	0	0	0	0	0	1	0	1	0	0	0
16/12/2022	F	60	0	1	1	0	1	1	1	0	0	0	1	0	0	0	0
13/01/2020	F	61	0	0	0	0	0	0	0	0	0	0	0	0	1	0	0
13/07/2020	F	61	0	0	0	0	0	0	0	0	0	0	0	0	0	0	1
20/07/2020	M	61	0	0	0	0	0	0	0	0	0	0	0	0	0	0	0
27/04/2021	M	61	0	0	0	0	0	0	0	0	0	0	0	0	0	0	0
12.02.2021	M	61	0	0	0	0	0	0	0	0	0	0	0	0	1	0	1
06/01/2023	F	61	0	0	0	0	0	0	0	0	0	0	0	0	0	0	1
24/04/2023	M	61	0	0	0	0	0	0	0	0	0	0	0	0	0	0	0
29/06/2020	M	61	0	0	0	0	0	0	0	1	0	0	0	0	0	0	1
10/02/2020	F	61	0	0	0	0	0	0	0	0	1	1	0	0	0	0	0
08/09/2020	M	61	1	0	0	0	0	0	0	0	0	0	1	1	1	0	0
07/02/2022	F	61	0	0	1	0	0	0	0	0	0	0	1	0	1	0	0
19/10/2022	F	61	0	1	0	0	0	0	0	0	0	0	0	0	1	0	0
01/04/2021	F	61	0	0	0	0	1	0	1	0	0	1	0	0	0	0	0
05/08/2020	M	62	0	0	0	0	0	0	0	0	0	0	0	0	0	0	1
02/09/2020	F	62	0	0	0	0	0	0	0	0	0	0	0	0	0	0	0
18/01/2021	M	62	0	0	0	0	0	0	0	0	0	0	0	0	0	0	1
25/03/2021	F	62	0	0	0	0	0	0	0	0	0	0	1	1	0	0	0
27/04/2021	F	62	0	0	0	0	0	0	0	0	0	0	0	0	0	0	0
08/09/2021	F	62	0	0	0	0	0	0	0	0	0	0	0	0	0	1	0
09/09/2021	M	62	0	0	0	0	0	0	0	0	0	0	0	0	0	0	0
07/10/2021	M	62	0	0	0	0	0	0	0	0	0	0	1	1	0	0	0
29.10.2021	F	62	0	0	0	0	0	0	0	0	0	0	0	0	0	1	0
01/03/2022	F	62	0	0	0	0	0	0	0	0	0	0	0	0	0	0	0
21/03/2022	M	62	0	0	0	0	0	0	0	0	0	0	0	0	0	0	1

16/05/2022	M	62	0	0	0	0	0	0	0	0	0	0	1	0	0	0	1
22/08/2022	F	62	0	0	0	0	0	0	0	0	0	0	0	0	0	0	1
28/09/2022	F	62	0	0	0	0	0	0	0	0	0	0	0	0	1	0	0
05/10/2022	F	62	0	0	0	0	0	0	0	0	0	0	1	0	0	0	0
26/10/2022	F	62	0	0	0	0	0	0	0	0	0	0	0	0	0	0	0
27/10/2022	F	62	0	0	0	0	0	0	0	0	0	0	0	0	0	0	0
14/12/2022	M	62	0	0	0	0	0	0	0	0	0	0	0	0	0	0	1
25/05/2023	F	62	0	0	0	0	0	0	0	0	0	0	0	0	0	0	1
06/06/2023	M	62	0	0	0	0	0	0	0	0	0	0	0	0	0	0	1
26/07/2023	F	62	0	0	0	0	0	0	0	0	0	0	1	0	0	0	0
18/09/2023	F	62	0	0	0	0	0	0	0	0	0	0	0	0	0	0	0
28/11/2023	F	62	0	0	0	0	0	0	0	0	0	0	0	0	1	0	0
13/03/2024	F	62	0	0	0	0	0	0	0	0	0	0	0	0	1	0	0
28/08/2020	M	62	0	0	0	0	0	0	0	0	1	0	1	1	0	0	0
05/10/2021	M	62	0	0	0	0	0	0	0	0	1	0	0	0	0	0	0
17.11.2021	F	62	0	0	0	0	0	0	0	0	0	1	0	1	0	0	0
17/01/2024	M	62	0	0	0	0	0	0	0	0	0	1	0	0	0	0	0
20/05/2024	F	62	0	0	0	0	0	0	0	0	1	0	0	0	0	0	0
13/08/2020	M	62	0	0	1	0	0	0	0	0	0	0	1	1	0	0	0
23/08/2023	M	62	0	0	1	0	0	0	0	0	0	0	1	0	0	0	0
29/06/2023	F	62	0	0	1	0	0	0	0	0	0	1	1	0	1	0	1
30/09/2022	F	62	0	1	1	0	0	0	0	0	0	0	1	0	1	0	0
12/10/2023	M	62	0	0	0	0	1	0	1	0	0	0	1	1	1	0	0
19.05.2021	M	62	0	1	0	0	1	0	0	0	0	1	1	0	0	0	0
03/08/2023	F	62	0	1	1	1	0	0	0	0	0	0	1	0	1	0	0
28/01/2020	F	63	0	0	0	0	0	0	0	0	0	0	0	0	0	0	0
21/02/2020	F	63	0	0	0	0	0	0	0	0	0	0	0	0	0	0	1
05/10/2020	F	63	0	0	0	0	0	0	0	0	0	0	0	0	1	0	0
14/10/2020	F	63	0	0	0	0	0	0	0	0	0	0	0	0	0	0	0

04/02/2021	F	63	0	0	0	0	0	0	0	0	0	0	0	0	0	0	0
23.04.2021	F	63	0	0	0	0	0	0	0	0	0	0	0	1	0	1	0
27/04/2022	M	63	0	0	0	0	0	0	0	0	0	0	0	1	0	0	0
10/05/2022	F	63	0	0	0	0	0	0	0	0	0	0	0	0	0	0	0
09/06/2022	F	63	0	0	0	0	0	0	0	0	0	0	1	0	1	0	0
23/08/2022	M	63	0	0	0	0	0	0	0	0	0	0	0	0	0	0	1
25/11/2022	F	63	0	0	0	0	0	0	0	0	0	0	0	0	0	0	0
25/11/2022	M	63	0	0	0	0	0	0	0	0	0	0	0	0	0	0	0
24/11/2023	F	63	0	0	0	0	0	0	0	0	0	0	0	0	0	0	1
08/02/2024	M	63	0	0	0	0	0	0	0	0	0	0	0	0	0	0	1
13/02/2020	F	63	0	0	0	0	0	0	0	0	1	0	1	0	0	0	0
14/11/2024	F	63	0	0	0	0	0	0	0	0	1	0	0	0	0	0	0
06/08/2020	M	63	0	1	0	0	0	0	0	0	0	0	1	0	0	0	0
04/09/2020	M	63	0	0	0	0	1	0	0	0	0	0	1	0	0	0	0
27/09/2023	F	63	0	1	0	0	0	0	0	0	0	0	1	0	1	0	0
02/11/2020	F	63	1	1	0	0	0	0	0	0	0	0	1	0	1	0	0
04/10/2023	F	63	1	0	1	0	0	0	1	0	0	0	1	0	1	1	1
17/02/2020	M	63	1	1	1	0	1	0	0	0	0	0	1	1	0	0	1
27/01/2020	F	64	0	0	0	0	0	0	0	0	0	0	1	1	1	0	1
14/02/2020	F	64	0	0	0	0	0	0	0	0	0	0	0	0	0	0	1
30/06/2020	F	64	0	0	0	0	0	0	0	0	0	0	0	0	0	0	0
02/02/2021	F	64	0	0	0	0	0	0	0	0	0	0	0	0	0	0	1
11/03/2021	F	64	0	0	0	0	0	0	0	0	0	0	0	0	0	0	0
13/05/2021	M	64	0	0	0	0	0	0	0	0	0	0	0	0	1	1	0
06/09/2021	M	64	0	0	0	0	0	0	0	0	0	0	1	0	1	0	0
15/09/2021	F	64	0	0	0	0	0	0	0	0	0	0	0	0	0	1	0
12.02.2021	M	64	0	0	0	0	0	0	0	0	0	0	1	0	1	0	1
23.06.2021	M	64	0	0	0	0	0	0	0	0	0	0	0	0	0	0	0
21/03/2022	M	64	0	0	0	0	0	0	0	0	0	0	0	0	0	0	0

20/06/2022	M	64	0	0	0	0	0	0	0	0	0	0	0	0	0	0	0
30/09/2022	M	64	0	0	0	0	0	0	0	0	0	0	1	0	0	0	0
24/10/2022	F	64	0	0	0	0	0	0	0	0	0	0	0	0	1	0	0
19/04/2023	F	64	0	0	0	0	0	0	0	0	0	0	0	0	1	0	0
06/10/2023	F	64	0	0	0	0	0	0	0	0	0	0	0	0	0	0	1
07/11/2023	F	64	0	0	0	0	0	0	0	0	0	0	1	0	1	0	0
17/11/2023	F	64	0	0	0	0	0	0	0	0	0	0	0	0	1	0	0
12/06/2024	M	64	0	0	0	0	0	0	0	0	0	0	0	0	0	0	1
27/09/2024	F	64	0	0	0	0	0	0	0	0	0	0	0	0	0	0	0
13/10/2021	M	64	0	0	0	0	0	0	0	0	0	1	0	1	0	0	0
02/03/2021	F	64	0	0	0	0	0	0	0	0	1	1	1	1	0	0	0
17/08/2020	M	64	0	0	1	0	0	0	0	0	0	0	1	1	0	0	0
14/09/2021	F	64	0	0	1	0	0	0	0	0	0	0	1	1	0	0	0
19/01/2021	F	64	0	0	0	0	0	0	1	0	1	0	1	0	0	0	0
04/02/2021	F	64	0	0	1	1	1	0	1	0	0	1	1	1	1	0	0
11/02/2020	M	65	0	0	0	0	0	0	0	0	0	0	1	0	0	0	0
14/09/2020	F	65	0	0	0	0	0	0	0	0	0	0	0	0	0	0	1
15/09/2020	F	65	0	0	0	0	0	0	0	0	0	0	1	1	0	0	0
16/09/2021	M	65	0	0	0	0	0	0	0	0	0	0	1	0	1	0	0
25/10/2021	F	65	0	0	0	0	0	0	0	0	0	0	0	0	1	0	1
14.05.2021	M	65	0	0	0	0	0	0	0	0	0	0	0	0	0	1	0
19/01/2022	F	65	0	0	0	0	0	0	0	0	0	0	0	0	0	0	1
29/03/2022	F	65	0	0	0	0	0	0	0	0	0	0	0	0	0	0	0
12/05/2022	M	65	0	0	0	0	0	0	0	0	0	0	0	0	0	0	0
25/11/2022	F	65	0	0	0	0	0	0	0	0	0	0	0	0	0	0	1
15/03/2023	M	65	0	0	0	0	0	0	0	0	0	0	0	0	0	0	1
06/04/2023	M	65	0	0	0	0	0	0	0	0	0	0	0	0	0	0	0
10/04/2023	M	65	0	0	0	0	0	0	0	0	0	0	0	0	0	0	0
09/08/2023	M	65	0	0	0	0	0	0	0	0	0	0	1	0	1	0	0

24/11/2023	F	65	0	0	0	0	0	0	0	0	0	0	0	0	0	0	1
15/02/2024	M	65	0	0	0	0	0	0	0	0	0	0	0	0	1	0	0
01/03/2024	F	65	0	0	0	0	0	0	0	0	0	0	1	0	0	0	1
28/03/2024	F	65	0	0	0	0	0	0	0	0	0	0	1	0	0	0	1
03/07/2024	M	65	0	0	0	0	0	0	0	0	0	0	1	0	0	0	0
20/04/2021	F	65	0	0	0	0	0	0	0	0	1	0	0	0	0	0	1
22/06/2020	F	65	0	0	1	0	0	0	0	0	0	0	1	1	0	0	0
26/05/2023	F	65	0	0	1	0	0	0	0	0	0	0	1	0	0	0	0
13/06/2023	F	65	0	0	0	0	0	0	1	0	0	0	1	0	1	0	0
13/10/2023	F	65	0	0	0	0	1	0	0	0	0	0	1	0	0	0	1
26/04/2024	F	65	0	0	0	0	1	0	0	0	0	0	0	0	1	0	0
29/01/2020	F	65	0	0	0	0	1	0	1	0	0	0	1	1	0	0	0
15/03/2021	F	65	0	0	0	0	1	1	0	0	1	0	1	0	1	0	0
04/02/2021	M	65	1	0	1	0	0	0	0	1	0	1	0	0	0	0	1
22/11/2022	M	65	0	0	1	0	1	1	0	0	0	0	1	1	1	0	0
11/03/2020	F	66	0	0	0	0	0	0	0	0	0	0	0	0	0	0	0
15/09/2020	F	66	0	0	0	0	0	0	0	0	0	0	1	1	0	0	0
15/09/2020	F	66	0	0	0	0	0	0	0	0	0	0	0	0	0	0	0
10/12/2020	F	66	0	0	0	0	0	0	0	0	0	0	0	0	0	0	1
04/10/2021	M	66	0	0	0	0	0	0	0	0	0	0	0	0	0	0	1
12.11.2021	F	66	0	0	0	0	0	0	0	0	0	0	0	0	0	1	0
28/03/2022	F	66	0	0	0	0	0	0	0	0	0	0	1	0	0	0	0
23/08/2022	M	66	0	0	0	0	0	0	0	0	0	0	0	0	0	0	1
18/09/2023	M	66	0	0	0	0	0	0	0	0	0	0	1	0	0	0	0
24/10/2023	F	66	0	0	0	0	0	0	0	0	0	0	0	0	0	0	1
25/06/2024	F	66	0	0	0	0	0	0	0	0	0	0	0	0	0	0	0
26/06/2024	F	66	0	0	0	0	0	0	0	0	0	0	0	0	0	0	1
02/10/2024	F	66	0	0	0	0	0	0	0	0	0	0	0	0	0	0	0
15/04/2021	F	66	0	0	0	0	0	0	0	0	1	0	0	0	0	0	1

26/03/2024	F	66	0	0	0	0	0	0	0	0	1	0	1	0	0	0	0
15.01.2021	F	66	0	0	0	0	1	0	0	0	0	0	0	0	1	0	0
01/08/2024	M	66	0	1	0	0	0	0	0	0	1	0	0	0	0	0	0
15/05/2023	M	66	0	0	1	1	0	0	0	0	0	0	1	0	1	0	0
31/07/2024	M	66	0	0	1	0	0	0	1	0	0	0	1	0	0	0	0
19/11/2024	M	66	1	0	1	0	0	1	0	0	0	0	1	1	0	0	0
27/02/2020	F	67	0	0	0	0	0	0	0	0	0	0	0	0	0	0	0
24/08/2021	F	67	0	0	0	0	0	0	0	0	0	0	0	0	0	0	1
28/10/2021	F	67	0	0	0	0	0	0	0	0	0	0	0	0	0	0	0
23/02/2022	F	67	0	0	0	0	0	0	0	0	0	0	0	0	0	0	0
27/04/2022	F	67	0	0	0	0	0	0	0	0	0	0	0	0	0	0	1
26/09/2022	F	67	0	0	0	0	0	0	0	0	0	0	0	0	0	0	0
01/03/2023	F	67	0	0	0	0	0	0	0	0	0	0	1	0	0	0	0
26/02/2024	M	67	0	0	0	0	0	0	0	0	0	0	0	0	0	0	0
23/07/2024	F	67	0	0	0	0	0	0	0	0	0	0	1	0	0	0	0
17/12/2024	F	67	0	0	0	0	0	0	0	0	0	0	0	0	0	0	1
27/05/2021	M	67	0	0	0	0	0	0	0	0	1	0	0	0	0	0	0
15/09/2021	M	67	0	0	0	0	0	0	0	0	0	1	1	0	0	0	0
08/10/2021	F	67	0	0	0	0	0	0	0	0	1	1	1	0	0	0	0
31/05/2023	M	67	0	1	0	0	0	0	0	0	0	0	1	0	1	0	0
07/08/2024	F	67	0	1	0	0	0	0	0	0	0	0	1	0	0	0	1
06/06/2024	M	67	0	0	1	1	0	0	0	0	0	0	1	1	0	0	0
16/02/2022	F	67	0	0	0	0	1	1	0	0	1	0	1	0	1	0	0
07/01/2021	F	67	1	1	1	1	1	0	0	0	0	0	1	1	0	0	0
14/02/2020	F	68	0	0	0	0	0	0	0	0	0	0	0	0	0	0	1
09/03/2020	F	68	0	0	0	0	0	0	0	0	0	0	1	0	1	0	0
29/09/2021	F	68	0	0	0	0	0	0	0	0	0	0	0	1	0	0	0
04/10/2021	M	68	0	0	0	0	0	0	0	0	0	0	0	0	0	0	0
26/10/2021	F	68	0	0	0	0	0	0	0	0	0	0	0	0	0	0	1

10/04/2023	F	68	0	0	0	0	0	0	0	0	0	0	0	0	0	0	0
25/06/2024	F	68	0	0	0	0	0	0	0	0	0	0	0	0	0	0	1
24/09/2024	F	68	0	0	0	0	0	0	0	0	0	0	1	0	1	0	0
15/11/2024	F	68	0	0	0	0	0	0	0	0	0	0	0	0	0	0	1
21/01/2021	F	68	0	0	0	0	0	0	0	0	1	0	1	0	1	0	0
27.10.2021	M	68	0	0	0	0	0	0	0	0	0	1	0	0	0	0	0
23/08/2023	F	68	0	0	0	0	0	0	0	0	1	0	0	0	1	0	1
05/12/2023	F	68	0	0	0	0	0	0	0	0	1	0	0	0	0	0	1
12/01/2022	F	68	0	0	0	0	0	0	0	1	1	0	0	0	0	0	0
26/07/2022	F	68	0	0	1	0	0	0	0	0	0	0	1	1	0	0	0
30/08/2024	M	68	0	0	1	0	0	0	0	0	0	0	1	1	0	0	0
22/11/2021	F	68	0	1	0	0	1	0	0	0	0	0	1	0	0	0	1
27/06/2023	F	68	0	0	0	0	1	1	0	0	0	0	0	0	0	0	1
26/06/2023	F	68	0	1	0	1	0	0	1	0	0	0	1	0	1	0	0
17/02/2020	F	69	0	0	0	0	0	0	0	0	0	0	0	0	1	0	0
29/09/2020	F	69	0	0	0	0	0	0	0	0	0	0	1	0	0	0	0
08/03/2021	F	69	0	0	0	0	0	0	0	0	0	0	0	0	1	0	0
28.04.2021	M	69	0	0	0	0	0	0	0	0	0	0	0	0	0	1	0
16/05/2022	M	69	0	0	0	0	0	0	0	0	0	0	0	0	0	0	0
11/08/2023	M	69	0	0	0	0	0	0	0	0	0	0	0	0	1	0	0
12/10/2023	F	69	0	0	0	0	0	0	0	0	0	0	0	0	0	0	1
27/10/2023	F	69	0	0	0	0	0	0	0	0	0	0	0	0	0	0	1
30/04/2024	F	69	0	0	0	0	0	0	0	0	0	0	0	0	0	0	0
02/07/2024	F	69	0	0	0	0	0	0	0	0	0	0	0	0	0	0	1
23/07/2024	F	69	0	0	0	0	0	0	0	0	0	0	0	0	0	0	0
24/09/2024	F	69	0	0	0	0	0	0	0	0	0	0	1	0	1	0	0
15/11/2024	F	69	0	0	0	0	0	0	0	0	0	0	0	0	0	0	0
17/10/2023	F	69	0	0	1	0	0	0	0	0	0	0	1	1	0	0	0
20/01/2020	F	70	0	0	0	0	0	0	0	0	0	0	1	0	1	0	0

20/01/2020	F	70	0	0	0	0	0	0	0	0	0	0	0	0	0	0	1
05/06/2020	F	70	0	0	0	0	0	0	0	0	0	0	0	0	0	0	1
29/09/2020	M	70	0	0	0	0	0	0	0	0	0	0	1	0	1	0	0
16/10/2020	F	70	0	0	0	0	0	0	0	0	0	0	0	0	1	0	0
29/06/2021	F	70	0	0	0	0	0	0	0	0	0	0	0	0	0	0	0
31/08/2021	M	70	0	0	0	0	0	0	0	0	0	0	0	0	0	0	1
08.09.2021	F	70	0	0	0	0	0	0	0	0	0	0	0	0	0	1	0
14.05.2021	F	70	0	0	0	0	0	0	0	0	0	0	0	0	0	1	0
23/02/2022	F	70	0	0	0	0	0	0	0	0	0	0	0	0	0	0	0
24/10/2022	F	70	0	0	0	0	0	0	0	0	0	0	0	0	0	0	1
08/03/2023	F	70	0	0	0	0	0	0	0	0	0	0	1	0	0	0	0
02/05/2023	F	70	0	0	0	0	0	0	0	0	0	0	0	0	0	0	0
26/05/2023	F	70	0	0	0	0	0	0	0	0	0	0	1	0	0	0	0
02/11/2023	F	70	0	0	0	0	0	0	0	0	0	0	0	0	0	0	1
07/02/2024	M	70	0	0	0	0	0	0	0	0	0	0	0	0	0	0	1
13/03/2024	M	70	0	0	0	0	0	0	0	0	0	0	0	0	0	0	1
02/08/2024	F	70	0	0	0	0	0	0	0	0	0	0	0	0	0	0	1
19.05.2021	F	70	0	0	0	0	0	0	0	0	1	0	0	0	0	0	0
05/10/2020	F	70	1	0	0	0	0	0	0	0	0	0	1	0	0	0	0
25.08.2021	F	70	0	0	0	0	0	0	1	0	0	0	0	1	0	0	0
19/10/2020	F	71	0	0	0	0	0	0	0	0	0	0	1	0	1	0	0
10.02.2021	M	71	0	0	0	0	0	0	0	0	0	0	0	0	0	0	1
11/08/2023	F	71	0	0	0	0	0	0	0	0	0	0	1	0	0	0	0
01/02/2024	F	71	0	0	0	0	0	0	0	0	0	0	0	0	0	0	0
08/07/2024	F	71	0	0	0	0	0	0	0	0	0	0	1	0	0	0	0
10/10/2024	F	71	0	0	0	0	0	0	0	0	0	0	0	0	0	0	1
14.05.2021	F	71	0	0	0	0	0	0	0	0	0	1	0	0	0	0	1
03/07/2020	M	71	0	0	0	1	0	0	0	0	0	0	1	1	0	0	0
01/02/2021	F	71	0	1	0	0	1	0	0	0	0	0	1	0	0	0	0

13/01/2020	F	72	0	0	0	0	0	0	0	0	0	0	1	0	1	0	0
21/01/2022	F	72	0	0	0	0	0	0	0	0	0	0	0	0	0	0	0
28/09/2022	F	72	0	0	0	0	0	0	0	0	0	0	0	1	1	0	0
29/06/2023	F	72	0	0	0	0	0	0	0	0	0	0	0	0	0	0	1
01/03/2024	F	72	0	0	0	0	0	0	0	0	0	0	1	0	1	0	0
21/05/2024	F	72	0	0	0	0	0	0	0	0	0	0	0	0	1	0	1
02/05/2022	M	73	0	0	0	0	0	0	0	0	0	0	0	0	1	0	1
02/05/2022	F	73	0	0	0	0	0	0	0	0	0	0	0	0	0	0	0
21/09/2023	F	73	0	0	0	0	0	0	0	0	0	0	1	0	1	0	0
20/11/2024	F	73	0	0	0	0	0	0	0	0	0	0	1	0	1	0	0
12/12/2024	F	73	0	0	0	0	0	0	0	0	0	0	0	0	0	0	0
02/08/2023	M	73	0	0	0	0	0	0	0	0	1	0	0	0	0	0	1
09/09/2020	F	73	0	0	0	0	1	0	0	0	0	0	1	0	1	0	0
12/03/2020	F	74	0	0	0	0	0	0	0	0	0	0	0	0	1	0	0
06/11/2020	M	74	0	0	0	0	0	0	0	0	0	0	0	0	0	0	1
03.09.2021	M	74	0	0	0	0	0	0	0	0	0	0	0	0	0	1	0
15/11/2022	F	74	0	0	0	0	0	0	0	0	0	0	0	0	0	0	1
20.01.2021	F	74	0	0	0	0	0	1	0	0	0	0	1	0	0	0	0
18/02/2022	F	74	0	0	0	0	1	0	0	0	0	0	1	0	1	0	1
25/08/2022	F	74	0	1	0	0	0	0	0	0	0	0	1	0	1	0	0
28/04/2023	M	74	0	1	0	0	0	0	0	0	0	0	1	0	0	0	0
26/08/2021	F	75	0	0	0	0	0	0	0	0	0	0	0	0	0	0	0
10.02.2021	F	75	0	0	0	0	0	0	0	0	0	0	0	0	1	0	1
07/03/2022	F	75	0	0	0	0	0	0	0	0	0	0	0	0	0	0	0
01/09/2022	F	75	0	0	0	0	0	0	0	0	0	0	0	0	1	0	0
01/04/2021	F	75	0	0	0	0	0	0	0	0	1	0	0	0	0	0	0
30/08/2022	M	75	0	0	1	0	0	0	0	0	0	0	1	1	1	1	0
29/05/2023	F	75	0	1	0	0	0	0	0	0	0	0	1	0	0	0	0
19.05.2021	F	76	0	0	0	0	0	0	0	0	0	0	0	0	0	1	0

06/03/2022	F	76	0	0	0	0	0	0	0	0	0	0	1	0	1	0	0
21/03/2023	M	76	0	0	0	0	0	0	0	1	0	0	1	0	0	0	0
29/07/2021	F	77	0	0	0	0	0	0	0	0	0	0	0	0	0	0	0
01/03/2022	M	77	0	0	0	0	0	0	0	0	0	0	0	0	0	0	1
15/11/2023	F	77	0	0	0	0	0	0	0	0	0	0	0	0	0	0	1
08/11/2023	F	77	0	1	1	0	0	0	0	0	0	0	1	0	0	0	1
13/01/2022	F	78	0	0	0	0	0	0	0	0	0	0	0	0	0	0	0
03/04/2023	F	78	0	0	0	0	0	0	0	0	0	0	1	0	0	0	0
31/01/2020	F	79	0	0	0	0	0	0	0	0	0	0	1	0	1	0	0
22/07/2020	F	79	0	0	0	0	0	0	0	0	0	0	0	0	0	0	0
09/06/2022	F	79	1	0	0	0	0	0	0	0	0	0	1	0	1	0	0
26/07/2022	F	80	0	0	0	0	0	0	0	0	0	0	0	0	0	0	1
24/05/2022	F	81	0	0	0	0	0	0	0	0	0	0	0	0	0	0	1
25/10/2024	F	81	0	0	0	0	0	0	0	0	0	0	0	0	1	0	0
22/01/2024	F	82	0	0	0	0	0	0	0	0	0	0	0	0	0	0	1
06/03/2023	M	83	0	0	0	0	0	0	0	0	1	0	0	0	0	0	1
25/01/2022	M	84	0	0	0	0	0	0	0	0	0	0	1	0	0	0	0
26/10/2021	F	85	0	0	0	0	0	0	0	0	0	0	0	0	0	0	1
29/06/2023	F	85	0	0	0	0	0	0	0	0	0	0	0	0	0	0	1
24/02/2020	F	85	0	0	0	0	0	0	0	0	1	0	0	0	0	0	1
07/12/2022	F	86	0	0	0	0	0	0	0	0	0	0	0	0	0	0	0
17.12.2021	F	86	0	0	0	0	0	0	0	0	0	1	0	0	0	1	0
15/06/2020	F	86	0	1	0	0	0	1	0	0	0	0	1	0	0	0	1
10.02.2021	F	87	0	0	0	0	0	0	0	0	0	0	1	0	1	0	0
26.11.2021	M	87	0	0	0	0	0	0	0	0	0	0	0	0	0	0	0
31/05/2022	F	90	0	0	0	0	0	0	0	0	1	0	0	0	0	0	1
26/04/2021	F	92	0	0	1	0	1	0	0	0	0	0	1	0	0	0	0
19/01/2022	M	0	0	0	0	0	0	0	0	0	0	0	0	0	0	0	1
03/09/2020	F	30	0	0	0	0	0	0	0	0	0	0	0	0	0	0	0
